# High astrovirus diversity in an endemic bat species suggests multiple spillovers from synanthropic rodents and birds

**DOI:** 10.1128/jvi.01357-24

**Published:** 2025-01-22

**Authors:** Rachel Leong, Axel O. G. Hoarau, Victoria Carcauzon, Marie Köster, Muriel Dietrich, Pablo Tortosa, Camille Lebarbenchon

**Affiliations:** 1 UMR Processus Infectieux en Milieu Insulaire Tropical, Université de La Réunion, Inserm, CNRS, IRD, Saint Denis de La Réunion, France; Emory University School of Medicine, Atlanta, Georgia, USA

**Keywords:** Reunion Island, *Mormopterus francoismoutoui*, Southwestern Indian Ocean, ecology, conservation, *Astroviridae*

## Abstract

Beyond the role of bats as natural host reservoirs of infectious agents, the impact of viral spillover from other animal species to bats has been neglected. Given the limited virus-host specificity of astroviruses (AstVs) and their propensity for cross-species transmission, we hypothesized that AstVs could be transmitted within animal communities (rodents, birds, and bats) and that native endemic bats may be exposed to viruses hosted by other species. We investigated the presence of AstV RNA in 3,796 biological samples collected in Reunion Island from *Mormopterus francoismoutoui* (*N* = 3421), an endemic free-tailed bat species, and also from small terrestrial mammals and birds: *Rattus rattus* (*N* = 146), *Rattus norvegicus* (*N* = 74), *Mus musculus domesticus* (*N* = 36), *Suncus murinus* (*N* = 99), and *Columba livia* (*N* = 20). We found significant differences in AstV prevalence between species (ranging from 1% ± 1.97% in *S. murinus* to 75% ± 18.9% in *C. livia*) as well as variation of pairwise identity of AstV sequences among host species, with a high diversity in *M. francoismoutoui* (mean pairwise identity: 53.2% ± 11.2%). Phylogenetic analyses further revealed that AstVs detected in bats did not cluster in a single clade and were genetically related to AstVs found in birds, rodents, pigs, dogs, cats, and reptiles. Our study suggests that bats may be exposed to viruses associated to synanthropic and alien species. Cascade effects of virus spillover toward endemic and endangered bat species need to be fully assessed.

**IMPORTANCE:**

Epidemiological consequences of cross-species transmission of zoonotic viruses are mostly considered from a health and economic perspective. Virus spillovers resulting from human-introduced species are much less considered, although they may have major consequences on the conservation of endemic and endangered bat species, in particular in an island context. Based on astrovirus detection and sequencing in an endemic bat species and five non-native species on a tropical island, we identified multiple and repeated viral introductions from synanthropic rodents and birds to bats, rather than the opposite. Such findings call for more investigations in these isolated and vulnerable ecosystems to better understand and mitigate the risks associated with pathogen spillovers.

## INTRODUCTION

Viral spillover is driven by a large set of factors ranging from environmental conditions to host population structure and dynamics (1). Epidemiological consequences of cross-species transmission of zoonotic viruses have mostly been considered for wildlife and livestock and primarily from a health and economic perspective. However, viral spillovers as a result of alien (i.e., non-native) species introduction are much less considered (2). Specifically in an island context, alien species introduction can pose a significant risk of pathogen spillover to native fauna (3). This has been investigated, for example, in birds (4), squirrels (5), amphibians, and pinnipeds, but never in bats, which are mostly viewed as a source of pathogenic viruses. Such introductions may, however, impact island bat populations and the transmission dynamics of their associated viruses (2). This calls for more investigation in these isolated and vulnerable ecosystems to better understand and mitigate the risks associated with pathogen spillovers.

The evolutionary history of astroviruses (AstVs; Family *Astroviridae*) shows a high propensity for cross-species transmission (6), suggesting limited host specificity and spillover potential to humans (7). *Avastrovirus* and *Mamastrovirus* affect numerous avian and mammalian species, respectively (8). Human AstVs are responsible for diarrhea, particularly in young children (7), and can also lead to infections of the central nervous system (9). Astroviruses have also been identified in numerous livestock, including chickens, ducks, pigs, and cows (8), and in wild animals, such as bats (10) and birds (11). This limited virus-host specificity thus highlights the need to assess AstV diversity and evolution at the ecosystem scale to identify cross-species transmission events and their epidemiological consequences.

Rodents and bats, the two most species-rich mammalian orders, are associated with most non-human AstV detections (6). The high genetic diversity of AstVs detected in these two mammalian groups demonstrates their ability to host a wide range of genetically diverse AstVs (6). However, not enough is known regarding the transmission routes or drivers of infection in these vertebrates. As AstV infections in bats can be highly seasonal, the period of sample collection and tested species could affect detection rates (10, 12, 13). Poor body conditions and co-infections with other viruses could also increase AstV infections in bats (14, 15).

Astroviruses have been reported in a large diversity of mammalian species in the islands of the Southwestern Indian Ocean. In Madagascar, multiple AstVs have been described not only among children (16) but also in 13 bat species (17, 18) and rodents (19). Astroviruses have also been reported in bat guano on Reunion Island (13), a 2,500 km² oceanic island located 700 km East of Madagascar. These AstVs were genetically related to viruses found in other vertebrate hosts (e.g., rats, dogs, reptiles [13]), rather than to bat AstVs previously detected in the Southwestern Indian Ocean (12, 17). However, the study was based on an environmental sampling of dry guano, and the origin of the detected viruses could therefore not be formally linked to the bat host species.

Reunion Island’s wild mammal community is mostly composed of five terrestrial species, all introduced following human colonization 350 years ago (*Rattus rattus*, *Rattus norvegicus*, *Mus musculus domesticus*, *Suncus murinus*, and *Tenrec ecaudatus*; 20), and three native bat species (*Mormopterus francoismoutoui*, *Taphozus mauritianus*, *Pteropus niger*). The Reunion free-tailed bat (*M. francoismoutoui*; Molossidae) is the island’s only endemic mammal, and the most abundant and widespread bat, roosting in large monospecific colonies in natural caves and urban spaces such as buildings, bridges, and houses (21, 22). The species share habitats with other mammals such as *R. rattus* and *M. musculus*, and birds such as *Columba livia* (in rooftops, bridges, etc). Such habitat sharing could favor AstV spillover between these hosts. The lack of phylogenetic structuring of AstVs previously detected in guano samples from the Reunion free-tailed bats may further suggest viral transmission from introduced mammals to the endemic bat species, rather than the opposite (13).

In this study, we focused on virus spillovers between the community of introduced and native vertebrates on Reunion Island. Because of limited virus-host specificity and a propensity for cross-species transmission at an ecosystem scale (6), we hypothesized that AstVs could easily be transmitted between different host species and investigate whether endemic bats could be infected with AstVs shed by alien species rather than reservoirs of bat-borne AstVs.

## MATERIALS AND METHODS

### Sample origin and nucleic acid extraction

We used intestine samples from terrestrial mammals that were collected in Reunion Island in 2012 and 2013, as part of previous investigations on fleas and *Leptospira* diversity (*n* = 355; Fig. S1; 20, 23). In total, we tested 146 *R*. *rattus*, 74 *R*. *norvegicus*, 36 *M*. *musculus domesticus,* and 99 *S*. *murinus* samples. For each sample, we grounded approximately 20 mg of the intestine in 500 µL of phosphate-buffered saline solution with the help of a Tissue Lyser (QIAGEN, Hilden, Germany) and two 3 mm stainless steel beads for 2 × 1 min at 30 Hz. We added 200 µL of shredded material to 180 µL of VXL lysis buffer. We performed RNA extraction with the IndiSpin QIAcube HT Pathogen Kit (QIAGEN, Hilden, Germany).

We opportunistically collected 20 fresh pigeon feces at a *M. francoismoutoui* roost located on a bridge in November 2021. We collected samples on the ground and around pigeon nests with sterile rayon-tipped applicators (Puritan, Guilford, ME, USA). We placed swabs in 1.5 mL of viral transport media (17). We extracted RNA from 140 µL of the sample’s supernatant and did the elution in 60 µL of AVE buffer, with the QIAamp Viral RNA mini kit (QIAGEN, Valencia, CA, USA). We conducted cDNA synthesis and AstV PCR for other samples (24).

For bats, we used fresh feces from two *M. francoismoutoui* maternity colonies that were collected between 2016 and 2020 (*N* = 3421; 24, 25) and previously tested for the presence of AstV RdRp gene (24).

### Molecular detection of astroviruses

We conducted reverse transcription with 10 µL of RNA (17). We tested cDNAs for the presence of the gene encoding the AstVs RNA-dependent RNA polymerase (RdRp) using a semi-nested PCR (26). We performed PCR with the GoTaq G2 Hot Start Green Master Mix (Promega, Madison, WI, USA) in an Applied Biosystems 2720 Thermocycler. We performed electrophoresis using 2% agarose gels stained with 2% Gelred (Biotium, Hayward, CA, USA). We submitted all PCR products of the expected size for direct Sanger sequencing (Genoscreen, Lille, France).

### Statistical and phylogenetic analysis

We performed χ^2^ tests to test the effect of the host species on the probability of successful AstV detection by PCR. As the sampling size was large for rodent species, we further tested the effects of the sex and sampling locations (*R. norvegicus*, *R. rattus,* and *M. musculus*). We conducted all statistical analyses with R 3.4.4 (27).

We performed phylogenetic analysis on partial RdRp gene sequences obtained in this study and 162 AstV sequences previously detected in a large diversity of bat species (in particular from Madagascar and Mozambique), as well as in other vertebrate hosts (e.g., rodents, avian, human, and livestock). We aligned the sequences with Clustal Omega 1.2.3. We performed a maximum likelihood (ML) analysis with PhyML 3.1 (28), an evolutionary model selected with Model Generator 0.85 (29), and 1,000 bootstraps. We conducted a second analysis at the scale of Reunion Island, including only sequences obtained in this study, to investigate the level of genetic variation within host species and spillover patterns between species. We applied Kruskal-Wallis test to compare the pairwise identity of AstV sequences within each host species.

## RESULTS

### Astrovirus prevalence

**Fig 1:**
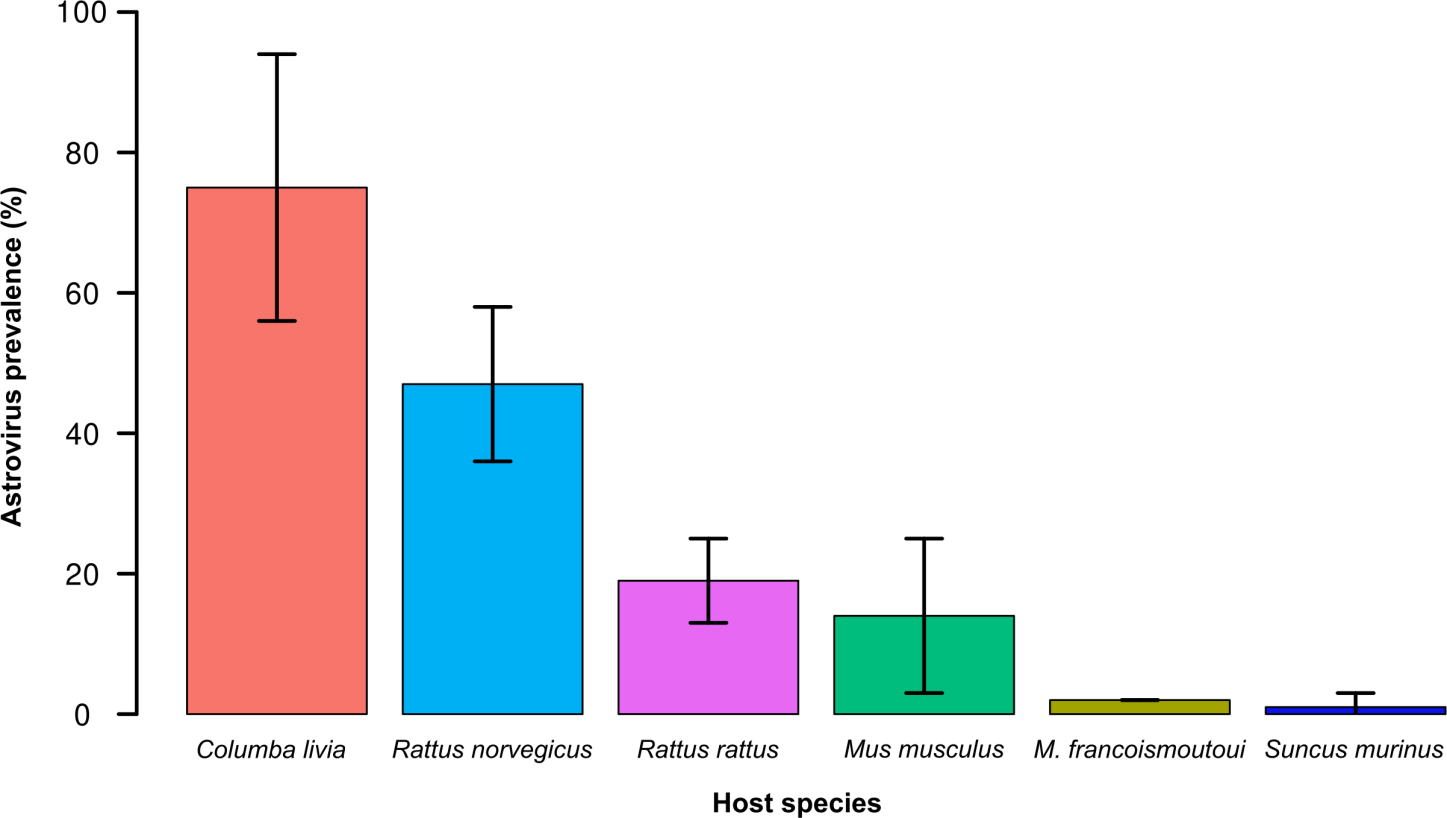
Astrovirus prevalence (± 95% CI) in six species in Reunion Island.

We detected AstVs in 3.7% of the tested samples (141 of 3,796 samples tested positive by PCR and were confirmed by sequencing). There was a significant difference in the prevalence of AstV PCR-positive animals between species (χ² = 317; df = 5; *P* < 0.001; Fig. 1). Prevalence was higher in pigeons (*C. livia*, 75% ± 18.9%) than in rats (*R. norvegicus*, 47.3% ± 11.4%; and *R. rattus*, 19.2% ± 6.4%), mice (*M. musculus*, 13.9% ± 11.3%), bats (*M. francoismoutoui*, 1.7% ± 0.4%), and shrews (*S. murinus*, 1% ± 1.97%).

**Fig 2:**
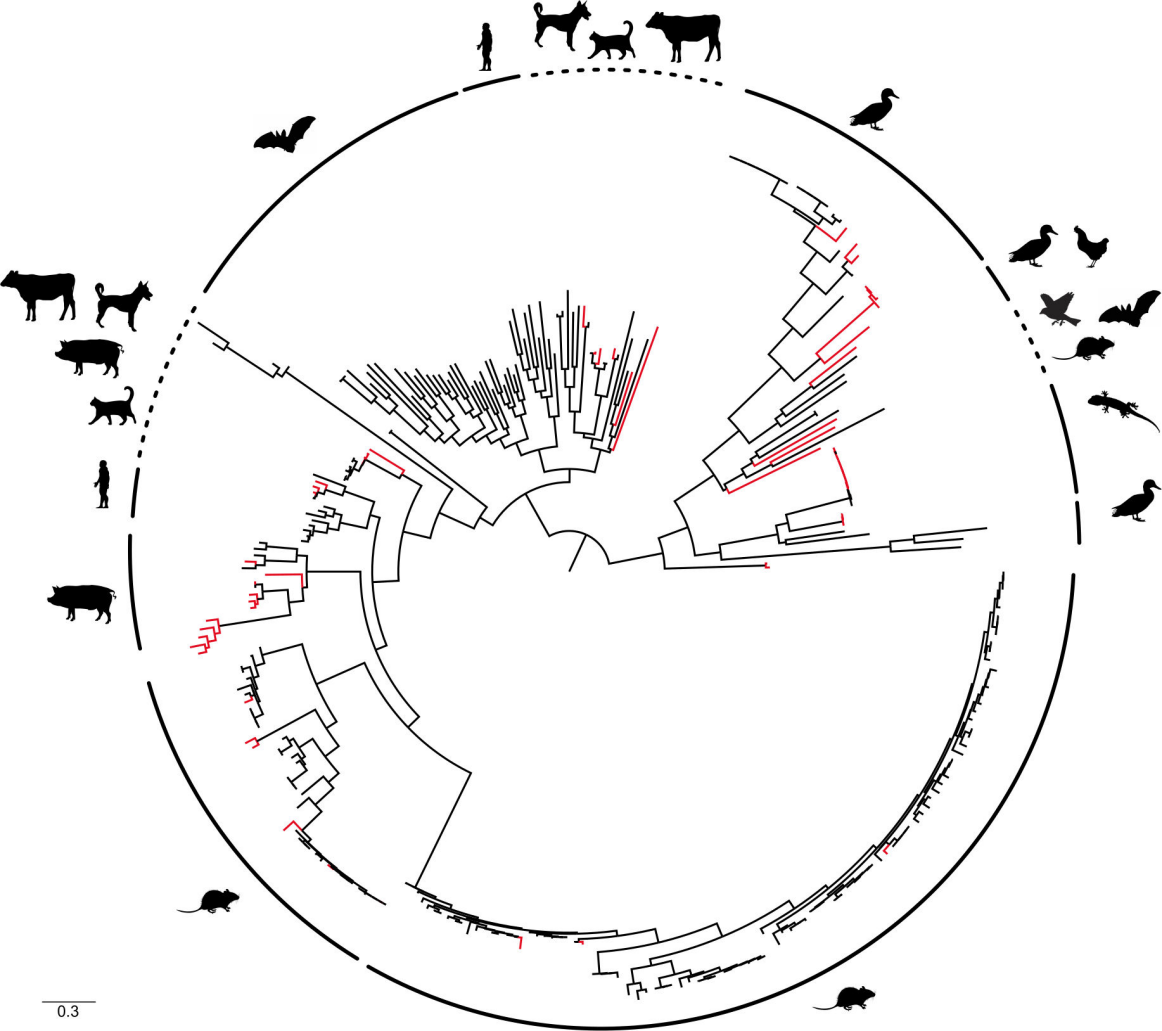
Maximum likelihood consensus tree derived from 303 Astrovirus (AstV) RNA-dependent RNA-polymerase partial nucleotide sequences (387 bp). Red branches indicate AstVs detected in the Reunion free-tailed bat (*Mormopterus francoismoutoui*). Further detail is available in Fig. S2.

Among rodents (rats and mice), there was also a significant difference in AstV prevalence between species (χ² = 22 ; df = 2; *P* < 0.001). We detected a significant effect of the sampling location on AstV prevalence for *R. rattus* (χ² = 47; df = 19; *P* < 0.001; Fig. 2) but not for *R. norvegicus* (χ² = 19; df = 13; *P* = 0.11) and *M. musculus* (χ² =14; df = 11; *P* = 0.23). This variation may, however, reflect the limited and unbalanced sample size between locations (Table S1). No significant difference in AstV prevalence was found between male and female rodents (χ² = 1.3 ; df = 1; *P* = 0.26).

### Genetic diversity and phylogenetic relatedness of bat AstVs

The phylogenetic analysis revealed that AstVs detected in Reunion free-tailed bats were not monophyletic and did not cluster in a single bat-specific AstVs clade (Fig. 2; Fig. S2). Indeed, none of the 57 AstVs sequences obtained from Reunion free-tailed bats clustered with bat AstVs, including the bat species located in Madagascar and Mozambique. Sequences were scattered across several genetic groups associated with different animal species: they were genetically related to AstVs found in not only birds (*Avastrovirus* types 1 and 2) but also rodents, pigs, dogs, cats (*Mamastrovirus*), and reptiles.

For non-bat host species, AstV sequences clustered with viruses previously detected in identical species or taxonomic groups. Indeed, AstVs detected in pigeons were phylogenetically related to Avian astrovirus (type 2), and most AstVs sequences from *R. rattus* and *R norvegicus* clustered with AstVs from other rodent species and locations (Fig. S2).

**Fig 3:**
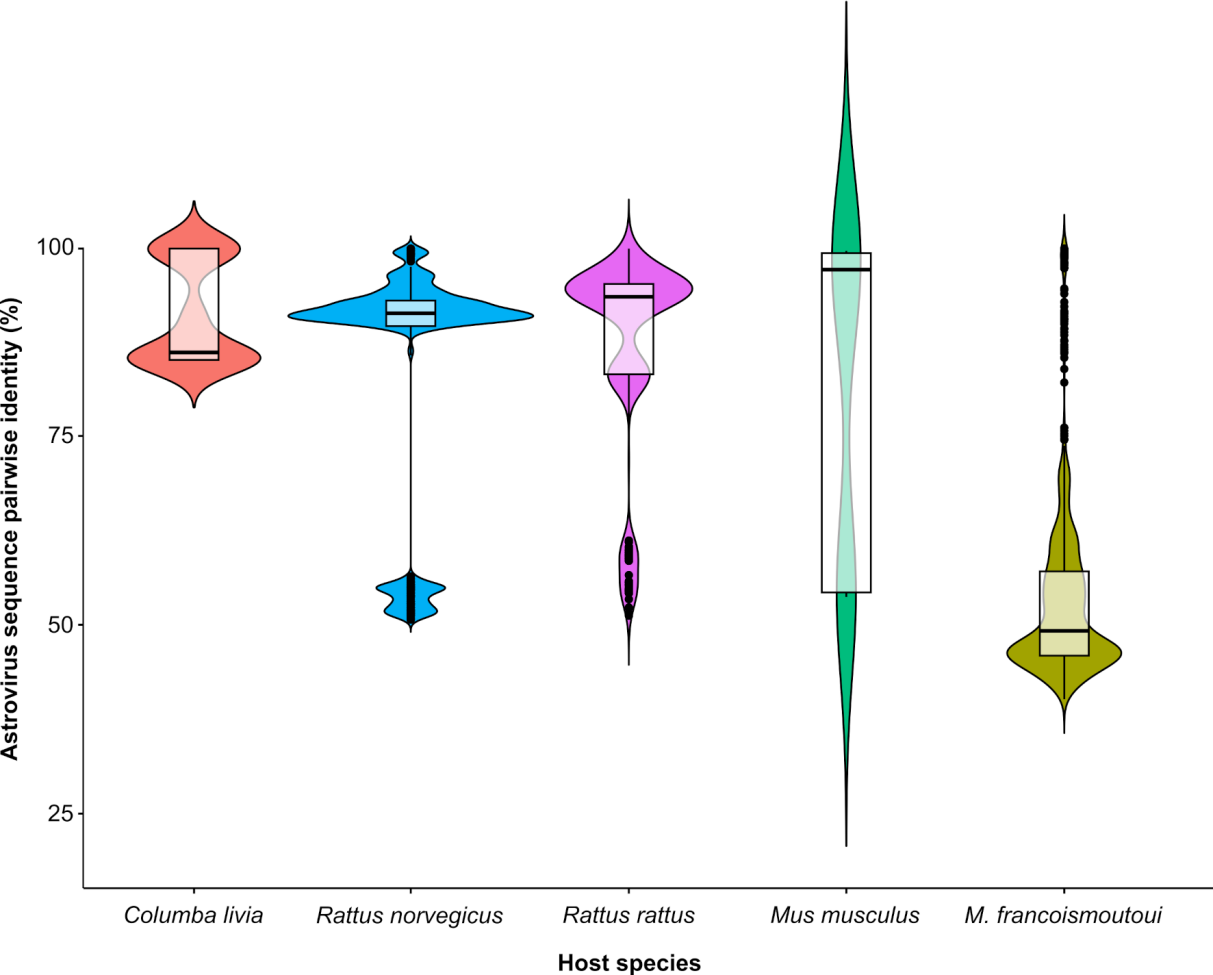
Astrovirus pairwise sequence identity within host species in Reunion Island.

**Fig 4:**
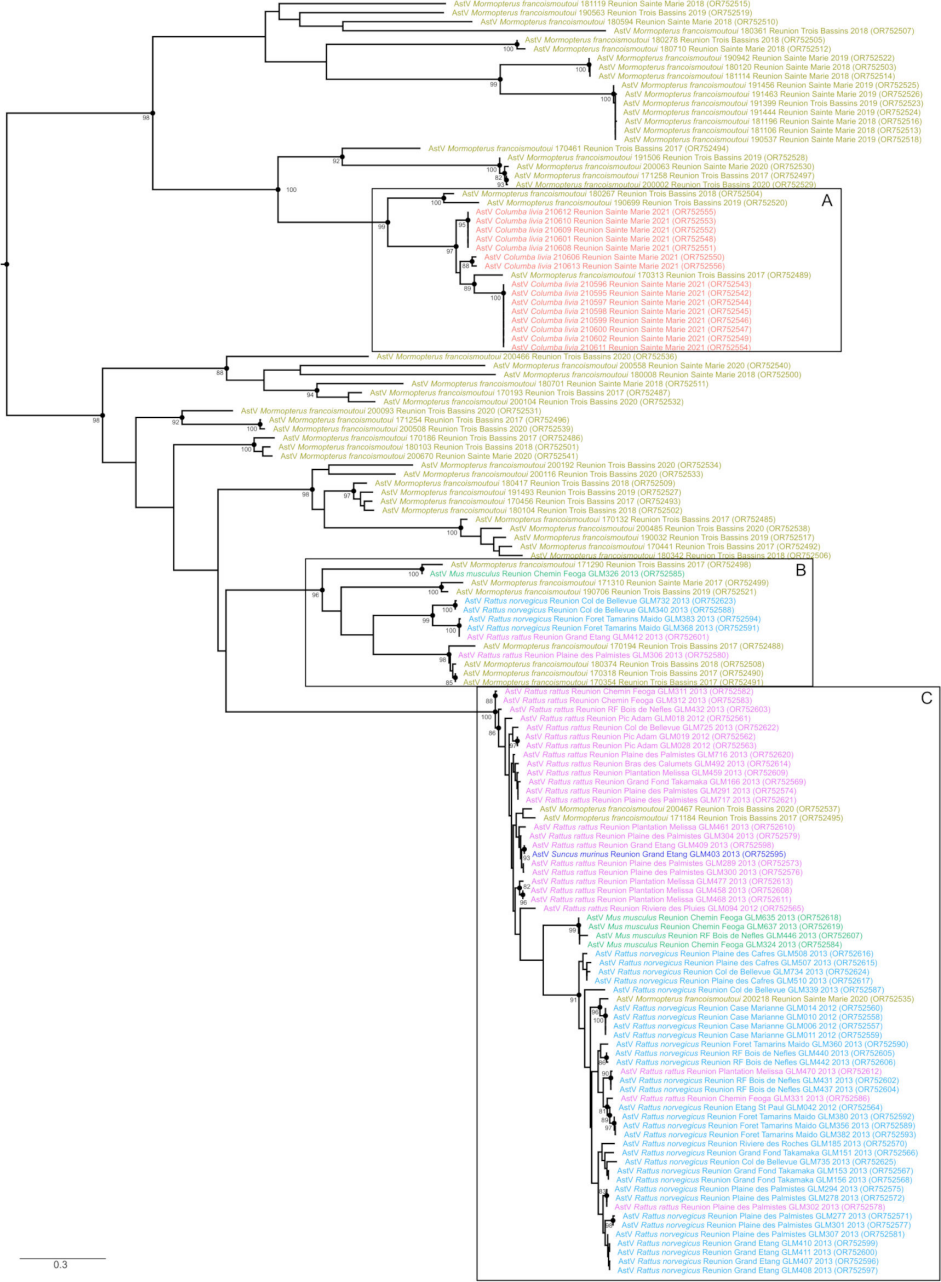
Maximum likelihood consensus tree derived from 141 Astrovirus (AstV) RNA-dependent RNA-polymerase partial nucleotide sequences (387 bp) from Reunion Island. The tree was generated with the general time reversible plus gamma (α = 0.88) and a proportion of invariant sites (I = 0.13) evolutionary model. Bootstrap values are reported when higher than 80. Taxa names were color-coded according to host species (as in Fig. 1 and 3).

Pairwise identity of AstV sequences was significantly different among host species (Kruskal-wallis test: *P* < 0.001), with a particularly high diversity hosted by Reunion free-tailed bats (mean pairwise identity: 53.2% ± 11.2%; Fig. 3). The second phylogenetic analysis, performed at the scale of Reunion Island only, further confirmed that AstVs detected from Reunion free-tailed bats were highly diverse and closely related to AstV detected in birds and rodents (Fig. 4).

Indeed, a highly supported clade including sequences obtained from *M. francoimoutoui* and *C. livia* suggests *Avastrovirus* type 2 transmission from pigeons to bats (Clade A in Fig. 4; Fig. S2). A second clade including sequences from *R. norvegicus*, *R. rattus*, *M. musculus*, and *M. francoimoutoui* may reflect AstV circulation in rodents and transmission to *M. francoimoutoui* (Clade B in Fig. 4; Fig. S2). A third and highly supported group of sequences (Clade C in Fig. 4; Fig. S2) could support virus transmission not only from *R. rattus* to *M. francoimoutoui* and to *S. murinus* but also from *R. norvegicus* to *M. francoimoutoui and* to *R. rattus*. Finally, other *M. francoimoutoui* sequences did not cluster in a single and monophyletic group, suggesting limited specificity between AstVs and this endemic bat species (Fig. 4). Instead, this may suggest viral introduction from a large diversity of mammalian and avian species (Fig. 2; Fig. S2).

## DISCUSSION

In this study, we focused on virus spillover between introduced mammals and birds, and an endemic bat species, on a highly isolated tropical island. Our hypothesis that AstVs can be transmitted between bats and other vertebrate species sharing the same habitat stems from a limited virus-host specificity and a propensity for cross-species transmission at an ecosystem scale (6). Although many bat species are considered natural reservoir hosts for AstVs worldwide, our results suggest multiple and repeated viral introductions from synanthropic rodents and birds to Reunion free-tailed bats.

High genetic diversity of bat AstVs has been described globally (e.g., 10) and, in the Western Indian Ocean, previously reported in Madagascar and Mozambique (12, 17, 18). Overall, this diversity reflects the circulation of not only several bat species-specific AstVs but also of multiple strains within a single bat species (10). Although the level of host specificity varies between studies, most bat AstVs cluster in a distinct and monophyletic clade among the *Mamastrovirus* phylogenetic tree (6), supporting an evolutionary divergence of bat AstVs. Contrary to these previous results on bats, the high genetic diversity we report in this study, together with the lack of monophyly and clustering with any other bat AstVs, suggests low specificity of AstVs in Reunion free-tailed bats. Interestingly, AstVs have not been reported in other Molossid bat species so far, except in *Mops condylurus* in Mozambique, and in *Chaerephon leucogaster* and *Mops leucostigma* in Madagascar (Fig. S2; 12, 18). These AstVs were phylogenetically related to neither other bat AstVs reported worldwide nor the ones we have detected in this study. These findings question the role of Molossid bats as natural reservoirs of AstVs and call for further investigations on this particular bat taxonomic group. Finally, sequencing of the capsid gene may provide more precise information not only on the potential of cross-species transmission but also on the recombination processes and AstV adaptation to their hosts. However, because of the challenge associated with virus isolation and sequencing, genomic data remain limited and impede current understanding of AstV evolutionary history in wild hosts.

Prevalence of bats shedding AstVs in feces was particularly low in Reunion free-tailed bats (<2%) compared with detection rates usually reported in most bat species (10). Although AstV prevalence varies between bat species, transmission may also be highly seasonal and therefore precludes comparison between transversal studies. For instance, a study conducted in a maternity colony of *Myotis myotis* reported detection rates of up to 51%, with a seasonal increase in AstV shedding in the bat population (30). Another investigation conducted in Zimbabwe also highlighted that the increase in AstV shedding coincided with the presence of juvenile bats (4–6 months old; 31). Viral transmission in bats has been shown to be highly dynamic not only for AstVs but also for many other infectious agents (30, 32–35). Shedding pulses are strongly associated with the demographic structure of bat populations and herd immunity. Compared with the major shedding variation of coronavirus, paramyxovirus, and *Leptospira* bacteria detected in the Reunion free-tailed bat (24, 25, 36), the limited AstV shedding variation, for 4 consecutive years, suggests that AstV transmission may not hold in this bat species. Rather, bat AstV infection could result from repeated exposures to viruses maintained in the environment (e.g., bat guano and water) or in other hosts sharing the same habitats, such as rats and pigeons but also feral dogs (*Canis lupus familiaris*), zebu (*Bos taurus indicus*), house sparrow (*Passer domesticus*), and common myna (*Acridotheres tristis*; 13). Indeed, the absence of phylogenetic clustering of most AstVs detected in Reunion free-tailed bats could indicate a larger AstV diversity and host range than the one we focused on in this study, in Reunion Island.

Virus spillover from synanthropic rodents and birds further implies that rats, mice, and pigeons were introduced to Reunion Island with their associated AstVs. The limited AstV genetic diversity and strong host specificity, in particular for *R. norvegicus*, *R. rattus*, *M. musculus,* and *C. livia,* supports such a scenario. To date, the effects of these introductions on bat population health have not been considered. Indeed, although bats are recognized as natural reservoirs for bat-AstVs, the consequences of bat infections with rodent-associated AstVs or *Avastroviruses* remain unknown. Given the pathogenic potential of several AstVs in humans and other mammals (e.g., mice, mink) and in poultry, the physiological and behavioral effects of rodent and avian AstV in bats need to be fully considered.

Reducing spillover risk is recognized as a key prevention strategy to limit human pandemics (37), and ecological countermeasures have been proposed to prevent the emergence of infectious diseases (38). Beyond the role of bats as natural host reservoirs of infectious agents, the impact of invasive alien species on the transmission of infectious agents to bats has been neglected. Our study demonstrates that bats may not only be a source of viruses with pandemic potentials but that they may be exposed to viruses associated with non-native and synanthropic species. In a context of global change and biodiversity loss, cascade effects of virus spillover from these hosts to endemic and endangered bat species need to be fully assessed.

## Data Availability

Data are available in supplemental Table S1 and in GenBank (accession numbers OR752485 to OR752625).

## SUPPLEMENTAL MATERIAL

Figure S1 jvi.01357-24-s0001.pdf Distribution of small mammal sampling sites.

Figure S2 jvi.01357-24-s0002.pdf Maximum likelihood consensus tree derived from 303 Astrovirus (AstV) RNA-dependent RNA polymerase partial nucleotide sequences (387 bp).

Supplemental legends jvi.01357-24-s0003.docx Legends for supplemental figures and table.

Table S1 jvi.01357-24-s0004.pdf Sample information and accession numbers of sequences generated in this study.

ASM does not own the copyrights to Supplemental Material that may be linked to, or accessed through, an article. The authors have granted ASM a non-exclusive, world-wide license to publish the Supplemental Material files. Please contact the corresponding author directly for reuse.
