# Supplementary figures and images for: High astrovirus diversity in an endemic bat species suggests multiple spillovers from synanthropic rodents and birds
Source: J Virol. 2025 Jan 22;99(2):e01357-24. doi: 10.1128/jvi.01357-24 (PMC11853114; doi:10.1128/jvi.01357-24)

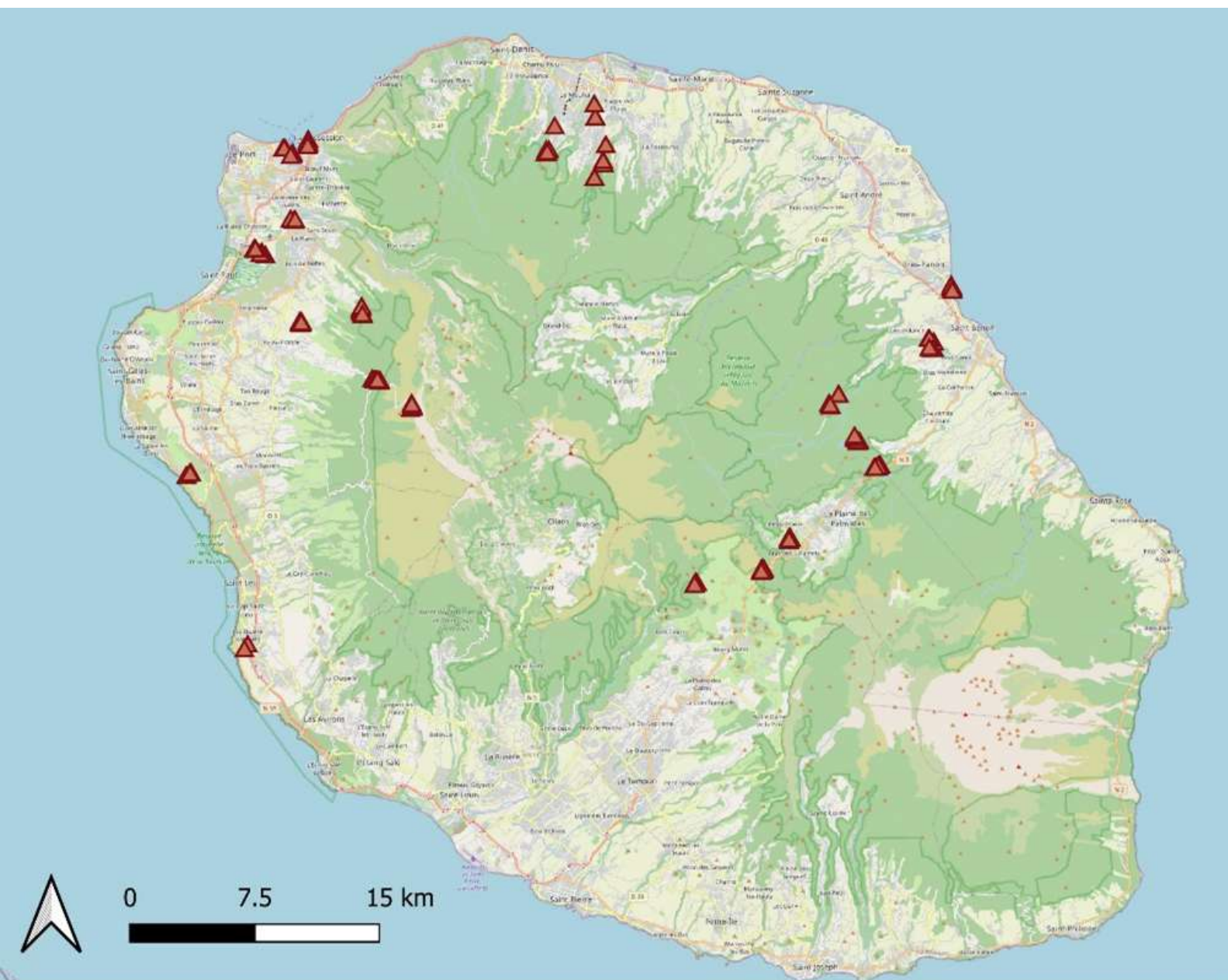

Supplement: Figure S1 — Distribution of small mammal sampling sites. [file jvi.01357-24-s0001.pdf]

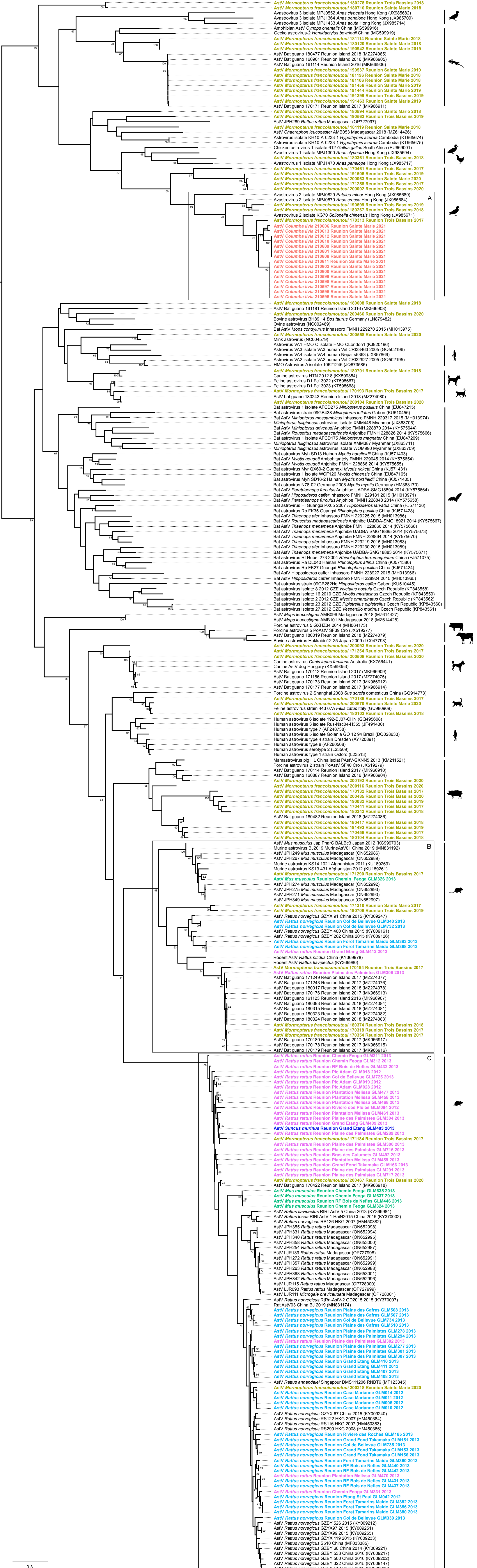

Supplement: Figure S2 — Maximum likelihood consensus tree derived from 303 Astrovirus (AstV) RNA-dependent RNA polymerase partial nucleotide sequences (387 bp). [file jvi.01357-24-s0002.pdf]
