# Supplementary material for: High astrovirus diversity in an endemic bat species suggests multiple spillovers from synanthropic rodents and birds
Source: J Virol. 2025 Jan 22;99(2):e01357-24. doi: 10.1128/jvi.01357-24 (PMC11853114; doi:10.1128/jvi.01357-24)
Supplement: Supplemental legends — Legends for supplemental figures and table. [file jvi.01357-24-s0003.docx]

**Supplementary files**

**Supplementary Figure 1.** Distribution of small mammal sampling sites.

**Supplementary Figure 2.** Maximum likelihood consensus tree derived from 303 Astrovirus (AstV) RNA-dependent RNA-polymerase partial nucleotide sequences (387 bp). The tree was generated with the transversion plus gamma (α = 0.84) and a proportion of invariant sites (I = 0.12) evolutionary model. Bootstrap values are reported when higher than 60. Taxa names corresponding to sequences generated in this study are indicated in bold and were color-coded according to host species.

**Supplementary Table 1.** Sample information and accession numbers of sequences generated in this study.
