# Supplementary material for: High astrovirus diversity in an endemic bat species suggests multiple spillovers from synanthropic rodents and birds
Source: J Virol. 2025 Jan 22;99(2):e01357-24. doi: 10.1128/jvi.01357-24 (PMC11853114; doi:10.1128/jvi.01357-24)
Supplement: Table S1 — Sample information and accession numbers of sequences generated in this study. [file jvi.01357-24-s0004.pdf]

| Sample | *Species* | Location | Sex | Astrovirus PCR | Sequence Accession Number |
|---|---|---|---|---|---|
| **21_0594** | *Columba livia* | OASteMarie | NA | 0 | NA |
| **21_0595** | *Columba livia* | OASteMarie | NA | 1 | OR752542 |
| **21_0596** | *Columba livia* | OASteMarie | NA | 1 | OR752543 |
| **21_0597** | *Columba livia* | OASteMarie | NA | 1 | OR752544 |
| **21_0598** | *Columba livia* | OASteMarie | NA | 1 | OR752545 |
| **21_0599** | *Columba livia* | OASteMarie | NA | 1 | OR752546 |
| **21_0600** | *Columba livia* | OASteMarie | NA | 1 | OR752547 |
| **21_0601** | *Columba livia* | OASteMarie | NA | 1 | OR752548 |
| **21_0602** | *Columba livia* | OASteMarie | NA | 1 | OR752549 |
| **21_0603** | *Columba livia* | OASteMarie | NA | 0 | NA |
| **21_0604** | *Columba livia* | OASteMarie | NA | 0 | NA |
| **21_0605** | *Columba livia* | OASteMarie | NA | 0 | NA |
| **21_0606** | *Columba livia* | OASteMarie | NA | 1 | OR752550 |
| **21_0607** | *Columba livia* | OASteMarie | NA | 0 | NA |
| **21_0608** | *Columba livia* | OASteMarie | NA | 1 | OR752551 |
| **21_0609** | *Columba livia* | OASteMarie | NA | 1 | OR752552 |
| **21_0610** | *Columba livia* | OASteMarie | NA | 1 | OR752553 |
| **21_0611** | *Columba livia* | OASteMarie | NA | 1 | OR752554 |
| **21_0612** | *Columba livia* | OASteMarie | NA | 1 | OR752555 |
| **21_0613** | *Columba livia* | OASteMarie | NA | 1 | OR752556 |
| **16_921** | *Mormopterus francoismoutoui* | Trois bassins | NA | 0 | NA |
| **16_922** | *Mormopterus francoismoutoui* | Trois bassins | NA | 0 | NA |
| **16_923** | *Mormopterus francoismoutoui* | Trois bassins | NA | 0 | NA |
| **16_924** | *Mormopterus francoismoutoui* | Trois bassins | NA | 0 | NA |
| **16_925** | *Mormopterus francoismoutoui* | Trois bassins | NA | 0 | NA |
| **16_926** | *Mormopterus francoismoutoui* | Trois bassins | NA | 0 | NA |
| **16_927** | *Mormopterus francoismoutoui* | Trois bassins | NA | 0 | NA |
| **16_928** | *Mormopterus francoismoutoui* | Trois bassins | NA | 0 | NA |
| **16_929** | *Mormopterus francoismoutoui* | Trois bassins | NA | 0 | NA |
| **16_930** | *Mormopterus francoismoutoui* | Trois bassins | NA | 0 | NA |
| **16_931** | *Mormopterus francoismoutoui* | Trois bassins | NA | 0 | NA |
| **16_932** | *Mormopterus francoismoutoui* | Trois bassins | NA | 0 | NA |
| **16_943** | *Mormopterus francoismoutoui* | Trois bassins | NA | 0 | NA |
| **16_944** | *Mormopterus francoismoutoui* | Trois bassins | NA | 0 | NA |
| **16_945** | *Mormopterus francoismoutoui* | Trois bassins | NA | 0 | NA |
| **16_946** | *Mormopterus francoismoutoui* | Trois bassins | NA | 0 | NA |
| **16_947** | *Mormopterus francoismoutoui* | Trois bassins | NA | 0 | NA |
| **16_948** | *Mormopterus francoismoutoui* | Trois bassins | NA | 0 | NA |
| **16_949** | *Mormopterus francoismoutoui* | Trois bassins | NA | 0 | NA |
| **16_950** | *Mormopterus francoismoutoui* | Trois bassins | NA | 0 | NA |
| **16_951** | *Mormopterus francoismoutoui* | Trois bassins | NA | 0 | NA |
| **16_952** | *Mormopterus francoismoutoui* | Trois bassins | NA | 0 | NA |
| **16_953** | *Mormopterus francoismoutoui* | Trois bassins | NA | 0 | NA |
| **16_954** | *Mormopterus francoismoutoui* | Trois bassins | NA | 0 | NA |
| **16_955** | *Mormopterus francoismoutoui* | Trois bassins | NA | 0 | NA |
| **16_956** | *Mormopterus francoismoutoui* | Trois bassins | NA | 0 | NA |
| **16_957** | *Mormopterus francoismoutoui* | Trois bassins | NA | 0 | NA |
| **16_958** | *Mormopterus francoismoutoui* | Trois bassins | NA | 0 | NA |
| **16_959** | *Mormopterus francoismoutoui* | Trois bassins | NA | 0 | NA |
| **16_960** | *Mormopterus francoismoutoui* | Trois bassins | NA | 0 | NA |
| **16_961** | *Mormopterus francoismoutoui* | Trois bassins | NA | 0 | NA |
| **16_962** | *Mormopterus francoismoutoui* | Trois bassins | NA | 0 | NA |
| **16_963** | *Mormopterus francoismoutoui* | Trois bassins | NA | 0 | NA |
| **16_964** | *Mormopterus francoismoutoui* | Trois bassins | NA | 0 | NA |
| **16_965** | *Mormopterus francoismoutoui* | Trois bassins | NA | 0 | NA |
| **16_966** | *Mormopterus francoismoutoui* | Trois bassins | NA | 0 | NA |
| **16_967** | *Mormopterus francoismoutoui* | Trois bassins | NA | 0 | NA |
| **16_968** | *Mormopterus francoismoutoui* | Trois bassins | NA | 0 | NA |
| **16_969** | *Mormopterus francoismoutoui* | Trois bassins | NA | 0 | NA |
| **16_970** | *Mormopterus francoismoutoui* | Trois bassins | NA | 0 | NA |
| **16_971** | *Mormopterus francoismoutoui* | Trois bassins | NA | 0 | NA |
| **16_972** | *Mormopterus francoismoutoui* | Trois bassins | NA | 0 | NA |
| **16_973** | *Mormopterus francoismoutoui* | Trois bassins | NA | 0 | NA |
| **16_974** | *Mormopterus francoismoutoui* | Trois bassins | NA | 0 | NA |
| **16_975** | *Mormopterus francoismoutoui* | Trois bassins | NA | 0 | NA |
| **16_976** | *Mormopterus francoismoutoui* | Trois bassins | NA | 0 | NA |
| **16_977** | *Mormopterus francoismoutoui* | Trois bassins | NA | 0 | NA |
| **16_978** | *Mormopterus francoismoutoui* | Trois bassins | NA | 0 | NA |
| **16_979** | *Mormopterus francoismoutoui* | Trois bassins | NA | 0 | NA |
| **16_980** | *Mormopterus francoismoutoui* | Trois bassins | NA | 0 | NA |
| **16_981** | *Mormopterus francoismoutoui* | Trois bassins | NA | 0 | NA |

| | | | | | |
|---|---|---|---|---|---|
| **16_992** | *Mormopterus francoismoutoui* | Trois bassins | NA | 0 | NA |
| **16_993** | *Mormopterus francoismoutoui* | Trois bassins | NA | 0 | NA |
| **16_994** | *Mormopterus francoismoutoui* | Trois bassins | NA | 0 | NA |
| **16_995** | *Mormopterus francoismoutoui* | Trois bassins | NA | 0 | NA |
| **16_996** | *Mormopterus francoismoutoui* | Trois bassins | NA | 0 | NA |
| **16_997** | *Mormopterus francoismoutoui* | Trois bassins | NA | 0 | NA |
| **16_998** | *Mormopterus francoismoutoui* | Trois bassins | NA | 0 | NA |
| **16_999** | *Mormopterus francoismoutoui* | Trois bassins | NA | 0 | NA |
| **16_1000** | *Mormopterus francoismoutoui* | Trois bassins | NA | 0 | NA |
| **16_1001** | *Mormopterus francoismoutoui* | Trois bassins | NA | 0 | NA |
| **16_1002** | *Mormopterus francoismoutoui* | Trois bassins | NA | 0 | NA |
| **16_1003** | *Mormopterus francoismoutoui* | Trois bassins | NA | 0 | NA |
| **16_1004** | *Mormopterus francoismoutoui* | Trois bassins | NA | 0 | NA |
| **16_1005** | *Mormopterus francoismoutoui* | Trois bassins | NA | 0 | NA |
| **16_1006** | *Mormopterus francoismoutoui* | Trois bassins | NA | 0 | NA |
| **16_1007** | *Mormopterus francoismoutoui* | Trois bassins | NA | 0 | NA |
| **16_1008** | *Mormopterus francoismoutoui* | Trois bassins | NA | 0 | NA |
| **16_1009** | *Mormopterus francoismoutoui* | Trois bassins | NA | 0 | NA |
| **16_1010** | *Mormopterus francoismoutoui* | Trois bassins | NA | 0 | NA |
| **16_1011** | *Mormopterus francoismoutoui* | Trois bassins | NA | 0 | NA |
| **16_1012** | *Mormopterus francoismoutoui* | Trois bassins | NA | 0 | NA |
| **16_1013** | *Mormopterus francoismoutoui* | Trois bassins | NA | 0 | NA |
| **16_1014** | *Mormopterus francoismoutoui* | Trois bassins | NA | 0 | NA |
| **16_1015** | *Mormopterus francoismoutoui* | Trois bassins | NA | 0 | NA |
| **16_1016** | *Mormopterus francoismoutoui* | Trois bassins | NA | 0 | NA |
| **16_1017** | *Mormopterus francoismoutoui* | Trois bassins | NA | 0 | NA |
| **16_1018** | *Mormopterus francoismoutoui* | Trois bassins | NA | 0 | NA |
| **16_1019** | *Mormopterus francoismoutoui* | Trois bassins | NA | 0 | NA |
| **16_1020** | *Mormopterus francoismoutoui* | Trois bassins | NA | 0 | NA |
| **16_1021** | *Mormopterus francoismoutoui* | Trois bassins | NA | 0 | NA |
| **16_1022** | *Mormopterus francoismoutoui* | Trois bassins | NA | 0 | NA |
| **16_1023** | *Mormopterus francoismoutoui* | Trois bassins | NA | 0 | NA |
| **16_1024** | *Mormopterus francoismoutoui* | Trois bassins | NA | 0 | NA |
| **16_1025** | *Mormopterus francoismoutoui* | Trois bassins | NA | 0 | NA |
| **16_1026** | *Mormopterus francoismoutoui* | Trois bassins | NA | 0 | NA |
| **16_1027** | *Mormopterus francoismoutoui* | Trois bassins | NA | 0 | NA |
| **16_1028** | *Mormopterus francoismoutoui* | Trois bassins | NA | 0 | NA |
| **16_1029** | *Mormopterus francoismoutoui* | Trois bassins | NA | 0 | NA |
| **16_1030** | *Mormopterus francoismoutoui* | Trois bassins | NA | 0 | NA |
| **16_1031** | *Mormopterus francoismoutoui* | Trois bassins | NA | 0 | NA |
| **16_1032** | *Mormopterus francoismoutoui* | Trois bassins | NA | 0 | NA |
| **16_1033** | *Mormopterus francoismoutoui* | Trois bassins | NA | 0 | NA |
| **16_1034** | *Mormopterus francoismoutoui* | Trois bassins | NA | 0 | NA |
| **16_1035** | *Mormopterus francoismoutoui* | Trois bassins | NA | 0 | NA |
| **16_1036** | *Mormopterus francoismoutoui* | Trois bassins | NA | 0 | NA |
| **16_1037** | *Mormopterus francoismoutoui* | Trois bassins | NA | 0 | NA |
| **16_1038** | *Mormopterus francoismoutoui* | Trois bassins | NA | 0 | NA |
| **16_1039** | *Mormopterus francoismoutoui* | Trois bassins | NA | 0 | NA |
| **16_1040** | *Mormopterus francoismoutoui* | Trois bassins | NA | 0 | NA |
| **16_1041** | *Mormopterus francoismoutoui* | Trois bassins | NA | 0 | NA |
| **16_1124** | *Mormopterus francoismoutoui* | Trois bassins | NA | 0 | NA |
| **16_1125** | *Mormopterus francoismoutoui* | Trois bassins | NA | 0 | NA |
| **16_1126** | *Mormopterus francoismoutoui* | Trois bassins | NA | 0 | NA |
| **16_1127** | *Mormopterus francoismoutoui* | Trois bassins | NA | 0 | NA |
| **16_1128** | *Mormopterus francoismoutoui* | Trois bassins | NA | 0 | NA |
| **16_1129** | *Mormopterus francoismoutoui* | Trois bassins | NA | 0 | NA |
| **16_1130** | *Mormopterus francoismoutoui* | Trois bassins | NA | 0 | NA |
| **16_1131** | *Mormopterus francoismoutoui* | Trois bassins | NA | 0 | NA |
| **16_1132** | *Mormopterus francoismoutoui* | Trois bassins | NA | 0 | NA |
| **16_1133** | *Mormopterus francoismoutoui* | Trois bassins | NA | 0 | NA |
| **16_1134** | *Mormopterus francoismoutoui* | Trois bassins | NA | 0 | NA |
| **16_1135** | *Mormopterus francoismoutoui* | Trois bassins | NA | 0 | NA |
| **16_1136** | *Mormopterus francoismoutoui* | Trois bassins | NA | 0 | NA |
| **16_1137** | *Mormopterus francoismoutoui* | Trois bassins | NA | 0 | NA |
| **16_1138** | *Mormopterus francoismoutoui* | Trois bassins | NA | 0 | NA |
| **16_1139** | *Mormopterus francoismoutoui* | Trois bassins | NA | 0 | NA |
| **16_1140** | *Mormopterus francoismoutoui* | Trois bassins | NA | 0 | NA |
| **16_1141** | *Mormopterus francoismoutoui* | Trois bassins | NA | 0 | NA |
| **16_1142** | *Mormopterus francoismoutoui* | Trois bassins | NA | 0 | NA |
| **16_1143** | *Mormopterus francoismoutoui* | Trois bassins | NA | 0 | NA |
| **16_1144** | *Mormopterus francoismoutoui* | Trois bassins | NA | 0 | NA |
| **16_1145** | *Mormopterus francoismoutoui* | Trois bassins | NA | 0 | NA |

| | | | | | |
|---|---|---|---|---|---|
| **16_1146** | *Mormopterus francoismoutoui* | Trois bassins | NA | 0 | NA |
| **16_1147** | *Mormopterus francoismoutoui* | Trois bassins | NA | 0 | NA |
| **16_1148** | *Mormopterus francoismoutoui* | Trois bassins | NA | 0 | NA |
| **16_1149** | *Mormopterus francoismoutoui* | Trois bassins | NA | 0 | NA |
| **16_1150** | *Mormopterus francoismoutoui* | Trois bassins | NA | 0 | NA |
| **16_1151** | *Mormopterus francoismoutoui* | Trois bassins | NA | 0 | NA |
| **16_1152** | *Mormopterus francoismoutoui* | Trois bassins | NA | 0 | NA |
| **16_1153** | *Mormopterus francoismoutoui* | Trois bassins | NA | 0 | NA |
| **16_1154** | *Mormopterus francoismoutoui* | Trois bassins | NA | 0 | NA |
| **16_1155** | *Mormopterus francoismoutoui* | Trois bassins | NA | 0 | NA |
| **16_1156** | *Mormopterus francoismoutoui* | Trois bassins | NA | 0 | NA |
| **16_1157** | *Mormopterus francoismoutoui* | Trois bassins | NA | 0 | NA |
| **16_1158** | *Mormopterus francoismoutoui* | Trois bassins | NA | 0 | NA |
| **16_1159** | *Mormopterus francoismoutoui* | Trois bassins | NA | 0 | NA |
| **16_1160** | *Mormopterus francoismoutoui* | Trois bassins | NA | 0 | NA |
| **16_1161** | *Mormopterus francoismoutoui* | Trois bassins | NA | 0 | NA |
| **16_1162** | *Mormopterus francoismoutoui* | Trois bassins | NA | 0 | NA |
| **16_1163** | *Mormopterus francoismoutoui* | Trois bassins | NA | 0 | NA |
| **16_1191** | *Mormopterus francoismoutoui* | Trois bassins | NA | 0 | NA |
| **16_1192** | *Mormopterus francoismoutoui* | Trois bassins | NA | 0 | NA |
| **16_1193** | *Mormopterus francoismoutoui* | Trois bassins | NA | 0 | NA |
| **16_1194** | *Mormopterus francoismoutoui* | Trois bassins | NA | 0 | NA |
| **16_1195** | *Mormopterus francoismoutoui* | Trois bassins | NA | 0 | NA |
| **16_1196** | *Mormopterus francoismoutoui* | Trois bassins | NA | 0 | NA |
| **16_1197** | *Mormopterus francoismoutoui* | Trois bassins | NA | 0 | NA |
| **16_1198** | *Mormopterus francoismoutoui* | Trois bassins | NA | 0 | NA |
| **16_1199** | *Mormopterus francoismoutoui* | Trois bassins | NA | 0 | NA |
| **16_1200** | *Mormopterus francoismoutoui* | Trois bassins | NA | 0 | NA |
| **16_1201** | *Mormopterus francoismoutoui* | Trois bassins | NA | 0 | NA |
| **16_1202** | *Mormopterus francoismoutoui* | Trois bassins | NA | 0 | NA |
| **16_1203** | *Mormopterus francoismoutoui* | Trois bassins | NA | 0 | NA |
| **16_1204** | *Mormopterus francoismoutoui* | Trois bassins | NA | 0 | NA |
| **16_1205** | *Mormopterus francoismoutoui* | Trois bassins | NA | 0 | NA |
| **16_1206** | *Mormopterus francoismoutoui* | Trois bassins | NA | 0 | NA |
| **16_1207** | *Mormopterus francoismoutoui* | Trois bassins | NA | 0 | NA |
| **16_1208** | *Mormopterus francoismoutoui* | Trois bassins | NA | 0 | NA |
| **16_1209** | *Mormopterus francoismoutoui* | Trois bassins | NA | 0 | NA |
| **16_1210** | *Mormopterus francoismoutoui* | Trois bassins | NA | 0 | NA |
| **16_1211** | *Mormopterus francoismoutoui* | Trois bassins | NA | 0 | NA |
| **16_1212** | *Mormopterus francoismoutoui* | Trois bassins | NA | 0 | NA |
| **16_1213** | *Mormopterus francoismoutoui* | Trois bassins | NA | 0 | NA |
| **16_1214** | *Mormopterus francoismoutoui* | Trois bassins | NA | 0 | NA |
| **16_1215** | *Mormopterus francoismoutoui* | Trois bassins | NA | 0 | NA |
| **16_1216** | *Mormopterus francoismoutoui* | Trois bassins | NA | 0 | NA |
| **16_1217** | *Mormopterus francoismoutoui* | Trois bassins | NA | 0 | NA |
| **16_1218** | *Mormopterus francoismoutoui* | Trois bassins | NA | 0 | NA |
| **16_1219** | *Mormopterus francoismoutoui* | Trois bassins | NA | 0 | NA |
| **16_1220** | *Mormopterus francoismoutoui* | Trois bassins | NA | 0 | NA |
| **16_1221** | *Mormopterus francoismoutoui* | Trois bassins | NA | 0 | NA |
| **16_1222** | *Mormopterus francoismoutoui* | Trois bassins | NA | 0 | NA |
| **16_1223** | *Mormopterus francoismoutoui* | Trois bassins | NA | 0 | NA |
| **16_1224** | *Mormopterus francoismoutoui* | Trois bassins | NA | 0 | NA |
| **16_1225** | *Mormopterus francoismoutoui* | Trois bassins | NA | 0 | NA |
| **16_1226** | *Mormopterus francoismoutoui* | Trois bassins | NA | 0 | NA |
| **16_1227** | *Mormopterus francoismoutoui* | Trois bassins | NA | 0 | NA |
| **16_1228** | *Mormopterus francoismoutoui* | Trois bassins | NA | 0 | NA |
| **16_1229** | *Mormopterus francoismoutoui* | Trois bassins | NA | 0 | NA |
| **16_1230** | *Mormopterus francoismoutoui* | Trois bassins | NA | 0 | NA |
| **16_1231** | *Mormopterus francoismoutoui* | Trois bassins | NA | 0 | NA |
| **16_1232** | *Mormopterus francoismoutoui* | Trois bassins | NA | 0 | NA |
| **16_1233** | *Mormopterus francoismoutoui* | Trois bassins | NA | 0 | NA |
| **16_1234** | *Mormopterus francoismoutoui* | Trois bassins | NA | 0 | NA |
| **16_1235** | *Mormopterus francoismoutoui* | Trois bassins | NA | 0 | NA |
| **16_1236** | *Mormopterus francoismoutoui* | Trois bassins | NA | 0 | NA |
| **16_1237** | *Mormopterus francoismoutoui* | Trois bassins | NA | 0 | NA |
| **16_1238** | *Mormopterus francoismoutoui* | Trois bassins | NA | 0 | NA |
| **16_1239** | *Mormopterus francoismoutoui* | Trois bassins | NA | 0 | NA |
| **16_1240** | *Mormopterus francoismoutoui* | Trois bassins | NA | 0 | NA |
| **17_0011** | *Mormopterus francoismoutoui* | Trois bassins | NA | 0 | NA |
| **17_0012** | *Mormopterus francoismoutoui* | Trois bassins | NA | 0 | NA |
| **17_0013** | *Mormopterus francoismoutoui* | Trois bassins | NA | 0 | NA |
| **17_0014** | *Mormopterus francoismoutoui* | Trois bassins | NA | 0 | NA |

| | | | | | |
|---|---|---|---|---|---|
| **17_0015** | *Mormopterus francoismoutoui* | Trois bassins | NA | 0 | NA |
| **17_0016** | *Mormopterus francoismoutoui* | Trois bassins | NA | 0 | NA |
| **17_0017** | *Mormopterus francoismoutoui* | Trois bassins | NA | 0 | NA |
| **17_0018** | *Mormopterus francoismoutoui* | Trois bassins | NA | 0 | NA |
| **17_0019** | *Mormopterus francoismoutoui* | Trois bassins | NA | 0 | NA |
| **17_0020** | *Mormopterus francoismoutoui* | Trois bassins | NA | 0 | NA |
| **17_0021** | *Mormopterus francoismoutoui* | Trois bassins | NA | 0 | NA |
| **17_0022** | *Mormopterus francoismoutoui* | Trois bassins | NA | 0 | NA |
| **17_0023** | *Mormopterus francoismoutoui* | Trois bassins | NA | 0 | NA |
| **17_0024** | *Mormopterus francoismoutoui* | Trois bassins | NA | 0 | NA |
| **17_0025** | *Mormopterus francoismoutoui* | Trois bassins | NA | 0 | NA |
| **17_0026** | *Mormopterus francoismoutoui* | Trois bassins | NA | 0 | NA |
| **17_0027** | *Mormopterus francoismoutoui* | Trois bassins | NA | 0 | NA |
| **17_0028** | *Mormopterus francoismoutoui* | Trois bassins | NA | 0 | NA |
| **17_0029** | *Mormopterus francoismoutoui* | Trois bassins | NA | 0 | NA |
| **17_0030** | *Mormopterus francoismoutoui* | Trois bassins | NA | 0 | NA |
| **17_0031** | *Mormopterus francoismoutoui* | Trois bassins | NA | 0 | NA |
| **17_0032** | *Mormopterus francoismoutoui* | Trois bassins | NA | 0 | NA |
| **17_0033** | *Mormopterus francoismoutoui* | Trois bassins | NA | 0 | NA |
| **17_0034** | *Mormopterus francoismoutoui* | Trois bassins | NA | 0 | NA |
| **17_0035** | *Mormopterus francoismoutoui* | Trois bassins | NA | 0 | NA |
| **17_0036** | *Mormopterus francoismoutoui* | Trois bassins | NA | 0 | NA |
| **17_0037** | *Mormopterus francoismoutoui* | Trois bassins | NA | 0 | NA |
| **17_0038** | *Mormopterus francoismoutoui* | Trois bassins | NA | 0 | NA |
| **17_0039** | *Mormopterus francoismoutoui* | Trois bassins | NA | 0 | NA |
| **17_0040** | *Mormopterus francoismoutoui* | Trois bassins | NA | 0 | NA |
| **17_0041** | *Mormopterus francoismoutoui* | Trois bassins | NA | 0 | NA |
| **17_0042** | *Mormopterus francoismoutoui* | Trois bassins | NA | 0 | NA |
| **17_0043** | *Mormopterus francoismoutoui* | Trois bassins | NA | 0 | NA |
| **17_0044** | *Mormopterus francoismoutoui* | Trois bassins | NA | 0 | NA |
| **17_0045** | *Mormopterus francoismoutoui* | Trois bassins | NA | 0 | NA |
| **17_0046** | *Mormopterus francoismoutoui* | Trois bassins | NA | 0 | NA |
| **17_0047** | *Mormopterus francoismoutoui* | Trois bassins | NA | 0 | NA |
| **17_0048** | *Mormopterus francoismoutoui* | Trois bassins | NA | 0 | NA |
| **17_0049** | *Mormopterus francoismoutoui* | Trois bassins | NA | 0 | NA |
| **17_0050** | *Mormopterus francoismoutoui* | Trois bassins | NA | 0 | NA |
| **17_0061** | *Mormopterus francoismoutoui* | Trois bassins | NA | 0 | NA |
| **17_0062** | *Mormopterus francoismoutoui* | Trois bassins | NA | 0 | NA |
| **17_0063** | *Mormopterus francoismoutoui* | Trois bassins | NA | 0 | NA |
| **17_0064** | *Mormopterus francoismoutoui* | Trois bassins | NA | 0 | NA |
| **17_0065** | *Mormopterus francoismoutoui* | Trois bassins | NA | 0 | NA |
| **17_0066** | *Mormopterus francoismoutoui* | Trois bassins | NA | 0 | NA |
| **17_0067** | *Mormopterus francoismoutoui* | Trois bassins | NA | 0 | NA |
| **17_0068** | *Mormopterus francoismoutoui* | Trois bassins | NA | 0 | NA |
| **17_0069** | *Mormopterus francoismoutoui* | Trois bassins | NA | 0 | NA |
| **17_0070** | *Mormopterus francoismoutoui* | Trois bassins | NA | 0 | NA |
| **17_0071** | *Mormopterus francoismoutoui* | Trois bassins | NA | 0 | NA |
| **17_0072** | *Mormopterus francoismoutoui* | Trois bassins | NA | 0 | NA |
| **17_0073** | *Mormopterus francoismoutoui* | Trois bassins | NA | 0 | NA |
| **17_0074** | *Mormopterus francoismoutoui* | Trois bassins | NA | 0 | NA |
| **17_0075** | *Mormopterus francoismoutoui* | Trois bassins | NA | 0 | NA |
| **17_0076** | *Mormopterus francoismoutoui* | Trois bassins | NA | 0 | NA |
| **17_0077** | *Mormopterus francoismoutoui* | Trois bassins | NA | 0 | NA |
| **17_0078** | *Mormopterus francoismoutoui* | Trois bassins | NA | 0 | NA |
| **17_0079** | *Mormopterus francoismoutoui* | Trois bassins | NA | 0 | NA |
| **17_0080** | *Mormopterus francoismoutoui* | Trois bassins | NA | 0 | NA |
| **17_0081** | *Mormopterus francoismoutoui* | Trois bassins | NA | 0 | NA |
| **17_0082** | *Mormopterus francoismoutoui* | Trois bassins | NA | 0 | NA |
| **17_0083** | *Mormopterus francoismoutoui* | Trois bassins | NA | 0 | NA |
| **17_0084** | *Mormopterus francoismoutoui* | Trois bassins | NA | 0 | NA |
| **17_0085** | *Mormopterus francoismoutoui* | Trois bassins | NA | 0 | NA |
| **17_0086** | *Mormopterus francoismoutoui* | Trois bassins | NA | 0 | NA |
| **17_0087** | *Mormopterus francoismoutoui* | Trois bassins | NA | 0 | NA |
| **17_0088** | *Mormopterus francoismoutoui* | Trois bassins | NA | 0 | NA |
| **17_0089** | *Mormopterus francoismoutoui* | Trois bassins | NA | 0 | NA |
| **17_0090** | *Mormopterus francoismoutoui* | Trois bassins | NA | 0 | NA |
| **17_0091** | *Mormopterus francoismoutoui* | Trois bassins | NA | 0 | NA |
| **17_0092** | *Mormopterus francoismoutoui* | Trois bassins | NA | 0 | NA |
| **17_0093** | *Mormopterus francoismoutoui* | Trois bassins | NA | 0 | NA |
| **17_0094** | *Mormopterus francoismoutoui* | Trois bassins | NA | 0 | NA |
| **17_0095** | *Mormopterus francoismoutoui* | Trois bassins | NA | 0 | NA |
| **17_0096** | *Mormopterus francoismoutoui* | Trois bassins | NA | 0 | NA |

| | | | | | |
|---|---|---|---|---|---|
| **17_0097** | *Mormopterus francoismoutoui* | Trois bassins | NA | 0 | NA |
| **17_0098** | *Mormopterus francoismoutoui* | Trois bassins | NA | 0 | NA |
| **17_0099** | *Mormopterus francoismoutoui* | Trois bassins | NA | 0 | NA |
| **17_0100** | *Mormopterus francoismoutoui* | Trois bassins | NA | 0 | NA |
| **17_0101** | *Mormopterus francoismoutoui* | Trois bassins | NA | 0 | NA |
| **17_0102** | *Mormopterus francoismoutoui* | Trois bassins | NA | 0 | NA |
| **17_0103** | *Mormopterus francoismoutoui* | Trois bassins | NA | 0 | NA |
| **17_0104** | *Mormopterus francoismoutoui* | Trois bassins | NA | 0 | NA |
| **17_0105** | *Mormopterus francoismoutoui* | Trois bassins | NA | 0 | NA |
| **17_0106** | *Mormopterus francoismoutoui* | Trois bassins | NA | 0 | NA |
| **17_0107** | *Mormopterus francoismoutoui* | Trois bassins | NA | 0 | NA |
| **17_0108** | *Mormopterus francoismoutoui* | Trois bassins | NA | 0 | NA |
| **17_0109** | *Mormopterus francoismoutoui* | Trois bassins | NA | 0 | NA |
| **17_0110** | *Mormopterus francoismoutoui* | Trois bassins | NA | 0 | NA |
| **17_0121** | *Mormopterus francoismoutoui* | Trois bassins | NA | 0 | NA |
| **17_0122** | *Mormopterus francoismoutoui* | Trois bassins | NA | 0 | NA |
| **17_0123** | *Mormopterus francoismoutoui* | Trois bassins | NA | 0 | NA |
| **17_0124** | *Mormopterus francoismoutoui* | Trois bassins | NA | 0 | NA |
| **17_0125** | *Mormopterus francoismoutoui* | Trois bassins | NA | 0 | NA |
| **17_0126** | *Mormopterus francoismoutoui* | Trois bassins | NA | 0 | NA |
| **17_0127** | *Mormopterus francoismoutoui* | Trois bassins | NA | 0 | NA |
| **17_0128** | *Mormopterus francoismoutoui* | Trois bassins | NA | 0 | NA |
| **17_0129** | *Mormopterus francoismoutoui* | Trois bassins | NA | 0 | NA |
| **17_0130** | *Mormopterus francoismoutoui* | Trois bassins | NA | 0 | NA |
| **17_0131** | *Mormopterus francoismoutoui* | Trois bassins | NA | 0 | NA |
| **17_0132** | *Mormopterus francoismoutoui* | Trois bassins | NA | 1 | OR752485 |
| **17_0133** | *Mormopterus francoismoutoui* | Trois bassins | NA | 0 | NA |
| **17_0134** | *Mormopterus francoismoutoui* | Trois bassins | NA | 0 | NA |
| **17_0135** | *Mormopterus francoismoutoui* | Trois bassins | NA | 0 | NA |
| **17_0136** | *Mormopterus francoismoutoui* | Trois bassins | NA | 0 | NA |
| **17_0137** | *Mormopterus francoismoutoui* | Trois bassins | NA | 0 | NA |
| **17_0138** | *Mormopterus francoismoutoui* | Trois bassins | NA | 0 | NA |
| **17_0139** | *Mormopterus francoismoutoui* | Trois bassins | NA | 0 | NA |
| **17_0140** | *Mormopterus francoismoutoui* | Trois bassins | NA | 0 | NA |
| **17_0141** | *Mormopterus francoismoutoui* | Trois bassins | NA | 0 | NA |
| **17_0142** | *Mormopterus francoismoutoui* | Trois bassins | NA | 0 | NA |
| **17_0143** | *Mormopterus francoismoutoui* | Trois bassins | NA | 0 | NA |
| **17_0144** | *Mormopterus francoismoutoui* | Trois bassins | NA | 0 | NA |
| **17_0145** | *Mormopterus francoismoutoui* | Trois bassins | NA | 0 | NA |
| **17_0146** | *Mormopterus francoismoutoui* | Trois bassins | NA | 0 | NA |
| **17_0147** | *Mormopterus francoismoutoui* | Trois bassins | NA | 0 | NA |
| **17_0148** | *Mormopterus francoismoutoui* | Trois bassins | NA | 0 | NA |
| **17_0149** | *Mormopterus francoismoutoui* | Trois bassins | NA | 0 | NA |
| **17_0150** | *Mormopterus francoismoutoui* | Trois bassins | NA | 0 | NA |
| **17_0151** | *Mormopterus francoismoutoui* | Trois bassins | NA | 0 | NA |
| **17_0152** | *Mormopterus francoismoutoui* | Trois bassins | NA | 0 | NA |
| **17_0153** | *Mormopterus francoismoutoui* | Trois bassins | NA | 0 | NA |
| **17_0154** | *Mormopterus francoismoutoui* | Trois bassins | NA | 0 | NA |
| **17_0155** | *Mormopterus francoismoutoui* | Trois bassins | NA | 0 | NA |
| **17_0156** | *Mormopterus francoismoutoui* | Trois bassins | NA | 0 | NA |
| **17_0157** | *Mormopterus francoismoutoui* | Trois bassins | NA | 0 | NA |
| **17_0158** | *Mormopterus francoismoutoui* | Trois bassins | NA | 0 | NA |
| **17_0159** | *Mormopterus francoismoutoui* | Trois bassins | NA | 0 | NA |
| **17_0160** | *Mormopterus francoismoutoui* | Trois bassins | NA | 0 | NA |
| **17_0161** | *Mormopterus francoismoutoui* | Trois bassins | NA | 0 | NA |
| **17_0162** | *Mormopterus francoismoutoui* | Trois bassins | NA | 0 | NA |
| **17_0163** | *Mormopterus francoismoutoui* | Trois bassins | NA | 0 | NA |
| **17_0164** | *Mormopterus francoismoutoui* | Trois bassins | NA | 0 | NA |
| **17_0165** | *Mormopterus francoismoutoui* | Trois bassins | NA | 0 | NA |
| **17_0166** | *Mormopterus francoismoutoui* | Trois bassins | NA | 0 | NA |
| **17_0167** | *Mormopterus francoismoutoui* | Trois bassins | NA | 0 | NA |
| **17_0168** | *Mormopterus francoismoutoui* | Trois bassins | NA | 0 | NA |
| **17_0169** | *Mormopterus francoismoutoui* | Trois bassins | NA | 0 | NA |
| **17_0170** | *Mormopterus francoismoutoui* | Trois bassins | NA | 0 | NA |
| **17_0181** | *Mormopterus francoismoutoui* | Trois bassins | NA | 0 | NA |
| **17_0182** | *Mormopterus francoismoutoui* | Trois bassins | NA | 0 | NA |
| **17_0183** | *Mormopterus francoismoutoui* | Trois bassins | NA | 0 | NA |
| **17_0184** | *Mormopterus francoismoutoui* | Trois bassins | NA | 0 | NA |
| **17_0185** | *Mormopterus francoismoutoui* | Trois bassins | NA | 0 | NA |
| **17_0186** | *Mormopterus francoismoutoui* | Trois bassins | NA | 1 | OR752486 |
| **17_0187** | *Mormopterus francoismoutoui* | Trois bassins | NA | 0 | NA |
| **17_0188** | *Mormopterus francoismoutoui* | Trois bassins | NA | 0 | NA |

| | | | | | |
|---|---|---|---|---|---|
| **17_0189** | *Mormopterus francoismoutoui* | Trois bassins | NA | 0 | NA |
| **17_0190** | *Mormopterus francoismoutoui* | Trois bassins | NA | 0 | NA |
| **17_0191** | *Mormopterus francoismoutoui* | Trois bassins | NA | 0 | NA |
| **17_0192** | *Mormopterus francoismoutoui* | Trois bassins | NA | 0 | NA |
| **17_0193** | *Mormopterus francoismoutoui* | Trois bassins | NA | 1 | OR752487 |
| **17_0194** | *Mormopterus francoismoutoui* | Trois bassins | NA | 1 | OR752488 |
| **17_0195** | *Mormopterus francoismoutoui* | Trois bassins | NA | 0 | NA |
| **17_0196** | *Mormopterus francoismoutoui* | Trois bassins | NA | 0 | NA |
| **17_0197** | *Mormopterus francoismoutoui* | Trois bassins | NA | 0 | NA |
| **17_0198** | *Mormopterus francoismoutoui* | Trois bassins | NA | 0 | NA |
| **17_0199** | *Mormopterus francoismoutoui* | Trois bassins | NA | 0 | NA |
| **17_0200** | *Mormopterus francoismoutoui* | Trois bassins | NA | 0 | NA |
| **17_0201** | *Mormopterus francoismoutoui* | Trois bassins | NA | 0 | NA |
| **17_0202** | *Mormopterus francoismoutoui* | Trois bassins | NA | 0 | NA |
| **17_0203** | *Mormopterus francoismoutoui* | Trois bassins | NA | 0 | NA |
| **17_0204** | *Mormopterus francoismoutoui* | Trois bassins | NA | 0 | NA |
| **17_0205** | *Mormopterus francoismoutoui* | Trois bassins | NA | 0 | NA |
| **17_0206** | *Mormopterus francoismoutoui* | Trois bassins | NA | 0 | NA |
| **17_0207** | *Mormopterus francoismoutoui* | Trois bassins | NA | 0 | NA |
| **17_0208** | *Mormopterus francoismoutoui* | Trois bassins | NA | 0 | NA |
| **17_0209** | *Mormopterus francoismoutoui* | Trois bassins | NA | 0 | NA |
| **17_0210** | *Mormopterus francoismoutoui* | Trois bassins | NA | 0 | NA |
| **17_0211** | *Mormopterus francoismoutoui* | Trois bassins | NA | 0 | NA |
| **17_0212** | *Mormopterus francoismoutoui* | Trois bassins | NA | 0 | NA |
| **17_0213** | *Mormopterus francoismoutoui* | Trois bassins | NA | 0 | NA |
| **17_0214** | *Mormopterus francoismoutoui* | Trois bassins | NA | 0 | NA |
| **17_0215** | *Mormopterus francoismoutoui* | Trois bassins | NA | 0 | NA |
| **17_0217** | *Mormopterus francoismoutoui* | Trois bassins | NA | 0 | NA |
| **17_0218** | *Mormopterus francoismoutoui* | Trois bassins | NA | 0 | NA |
| **17_0219** | *Mormopterus francoismoutoui* | Trois bassins | NA | 0 | NA |
| **17_0220** | *Mormopterus francoismoutoui* | Trois bassins | NA | 0 | NA |
| **17_0221** | *Mormopterus francoismoutoui* | Trois bassins | NA | 0 | NA |
| **17_0222** | *Mormopterus francoismoutoui* | Trois bassins | NA | 0 | NA |
| **17_0223** | *Mormopterus francoismoutoui* | Trois bassins | NA | 0 | NA |
| **17_0224** | *Mormopterus francoismoutoui* | Trois bassins | NA | 0 | NA |
| **17_0225** | *Mormopterus francoismoutoui* | Trois bassins | NA | 0 | NA |
| **17_0226** | *Mormopterus francoismoutoui* | Trois bassins | NA | 0 | NA |
| **17_0227** | *Mormopterus francoismoutoui* | Trois bassins | NA | 0 | NA |
| **17_0228** | *Mormopterus francoismoutoui* | Trois bassins | NA | 0 | NA |
| **17_0229** | *Mormopterus francoismoutoui* | Trois bassins | NA | 0 | NA |
| **17_0230** | *Mormopterus francoismoutoui* | Trois bassins | NA | 0 | NA |
| **17_0310** | *Mormopterus francoismoutoui* | Trois bassins | NA | 0 | NA |
| **17_0311** | *Mormopterus francoismoutoui* | Trois bassins | NA | 0 | NA |
| **17_0312** | *Mormopterus francoismoutoui* | Trois bassins | NA | 0 | NA |
| **17_0313** | *Mormopterus francoismoutoui* | Trois bassins | NA | 1 | OR752489 |
| **17_0314** | *Mormopterus francoismoutoui* | Trois bassins | NA | 0 | NA |
| **17_0315** | *Mormopterus francoismoutoui* | Trois bassins | NA | 0 | NA |
| **17_0316** | *Mormopterus francoismoutoui* | Trois bassins | NA | 0 | NA |
| **17_0317** | *Mormopterus francoismoutoui* | Trois bassins | NA | 0 | NA |
| **17_0318** | *Mormopterus francoismoutoui* | Trois bassins | NA | 1 | OR752490 |
| **17_0319** | *Mormopterus francoismoutoui* | Trois bassins | NA | 0 | NA |
| **17_0320** | *Mormopterus francoismoutoui* | Trois bassins | NA | 0 | NA |
| **17_0321** | *Mormopterus francoismoutoui* | Trois bassins | NA | 0 | NA |
| **17_0322** | *Mormopterus francoismoutoui* | Trois bassins | NA | 0 | NA |
| **17_0323** | *Mormopterus francoismoutoui* | Trois bassins | NA | 0 | NA |
| **17_0324** | *Mormopterus francoismoutoui* | Trois bassins | NA | 0 | NA |
| **17_0325** | *Mormopterus francoismoutoui* | Trois bassins | NA | 0 | NA |
| **17_0326** | *Mormopterus francoismoutoui* | Trois bassins | NA | 0 | NA |
| **17_0327** | *Mormopterus francoismoutoui* | Trois bassins | NA | 0 | NA |
| **17_0328** | *Mormopterus francoismoutoui* | Trois bassins | NA | 0 | NA |
| **17_0329** | *Mormopterus francoismoutoui* | Trois bassins | NA | 0 | NA |
| **17_0330** | *Mormopterus francoismoutoui* | Trois bassins | NA | 0 | NA |
| **17_0331** | *Mormopterus francoismoutoui* | Trois bassins | NA | 0 | NA |
| **17_0332** | *Mormopterus francoismoutoui* | Trois bassins | NA | 0 | NA |
| **17_0333** | *Mormopterus francoismoutoui* | Trois bassins | NA | 0 | NA |
| **17_0334** | *Mormopterus francoismoutoui* | Trois bassins | NA | 0 | NA |
| **17_0335** | *Mormopterus francoismoutoui* | Trois bassins | NA | 0 | NA |
| **17_0336** | *Mormopterus francoismoutoui* | Trois bassins | NA | 0 | NA |
| **17_0337** | *Mormopterus francoismoutoui* | Trois bassins | NA | 0 | NA |
| **17_0338** | *Mormopterus francoismoutoui* | Trois bassins | NA | 0 | NA |
| **17_0339** | *Mormopterus francoismoutoui* | Trois bassins | NA | 0 | NA |
| **17_0340** | *Mormopterus francoismoutoui* | Trois bassins | NA | 0 | NA |

| | | | | | |
|---|---|---|---|---|---|
| **17_0341** | *Mormopterus francoismoutoui* | Trois bassins | NA | 0 | NA |
| **17_0342** | *Mormopterus francoismoutoui* | Trois bassins | NA | 0 | NA |
| **17_0343** | *Mormopterus francoismoutoui* | Trois bassins | NA | 0 | NA |
| **17_0344** | *Mormopterus francoismoutoui* | Trois bassins | NA | 0 | NA |
| **17_0345** | *Mormopterus francoismoutoui* | Trois bassins | NA | 0 | NA |
| **17_0346** | *Mormopterus francoismoutoui* | Trois bassins | NA | 0 | NA |
| **17_0347** | *Mormopterus francoismoutoui* | Trois bassins | NA | 0 | NA |
| **17_0348** | *Mormopterus francoismoutoui* | Trois bassins | NA | 0 | NA |
| **17_0349** | *Mormopterus francoismoutoui* | Trois bassins | NA | 0 | NA |
| **17_0350** | *Mormopterus francoismoutoui* | Trois bassins | NA | 0 | NA |
| **17_0351** | *Mormopterus francoismoutoui* | Trois bassins | NA | 0 | NA |
| **17_0352** | *Mormopterus francoismoutoui* | Trois bassins | NA | 0 | NA |
| **17_0353** | *Mormopterus francoismoutoui* | Trois bassins | NA | 0 | NA |
| **17_0354** | *Mormopterus francoismoutoui* | Trois bassins | NA | 1 | OR752491 |
| **17_0355** | *Mormopterus francoismoutoui* | Trois bassins | NA | 0 | NA |
| **17_0356** | *Mormopterus francoismoutoui* | Trois bassins | NA | 0 | NA |
| **17_0357** | *Mormopterus francoismoutoui* | Trois bassins | NA | 0 | NA |
| **17_0358** | *Mormopterus francoismoutoui* | Trois bassins | NA | 0 | NA |
| **17_0359** | *Mormopterus francoismoutoui* | Trois bassins | NA | 0 | NA |
| **17_0372** | *Mormopterus francoismoutoui* | Trois bassins | NA | 0 | NA |
| **17_0373** | *Mormopterus francoismoutoui* | Trois bassins | NA | 0 | NA |
| **17_0374** | *Mormopterus francoismoutoui* | Trois bassins | NA | 0 | NA |
| **17_0375** | *Mormopterus francoismoutoui* | Trois bassins | NA | 0 | NA |
| **17_0376** | *Mormopterus francoismoutoui* | Trois bassins | NA | 0 | NA |
| **17_0377** | *Mormopterus francoismoutoui* | Trois bassins | NA | 0 | NA |
| **17_0378** | *Mormopterus francoismoutoui* | Trois bassins | NA | 0 | NA |
| **17_0379** | *Mormopterus francoismoutoui* | Trois bassins | NA | 0 | NA |
| **17_0380** | *Mormopterus francoismoutoui* | Trois bassins | NA | 0 | NA |
| **17_0381** | *Mormopterus francoismoutoui* | Trois bassins | NA | 0 | NA |
| **17_0382** | *Mormopterus francoismoutoui* | Trois bassins | NA | 0 | NA |
| **17_0383** | *Mormopterus francoismoutoui* | Trois bassins | NA | 0 | NA |
| **17_0384** | *Mormopterus francoismoutoui* | Trois bassins | NA | 0 | NA |
| **17_0385** | *Mormopterus francoismoutoui* | Trois bassins | NA | 0 | NA |
| **17_0386** | *Mormopterus francoismoutoui* | Trois bassins | NA | 0 | NA |
| **17_0387** | *Mormopterus francoismoutoui* | Trois bassins | NA | 0 | NA |
| **17_0388** | *Mormopterus francoismoutoui* | Trois bassins | NA | 0 | NA |
| **17_0389** | *Mormopterus francoismoutoui* | Trois bassins | NA | 0 | NA |
| **17_0390** | *Mormopterus francoismoutoui* | Trois bassins | NA | 0 | NA |
| **17_0391** | *Mormopterus francoismoutoui* | Trois bassins | NA | 0 | NA |
| **17_0392** | *Mormopterus francoismoutoui* | Trois bassins | NA | 0 | NA |
| **17_0393** | *Mormopterus francoismoutoui* | Trois bassins | NA | 0 | NA |
| **17_0394** | *Mormopterus francoismoutoui* | Trois bassins | NA | 0 | NA |
| **17_0395** | *Mormopterus francoismoutoui* | Trois bassins | NA | 0 | NA |
| **17_0396** | *Mormopterus francoismoutoui* | Trois bassins | NA | 0 | NA |
| **17_0397** | *Mormopterus francoismoutoui* | Trois bassins | NA | 0 | NA |
| **17_0398** | *Mormopterus francoismoutoui* | Trois bassins | NA | 0 | NA |
| **17_0399** | *Mormopterus francoismoutoui* | Trois bassins | NA | 0 | NA |
| **17_0400** | *Mormopterus francoismoutoui* | Trois bassins | NA | 0 | NA |
| **17_0401** | *Mormopterus francoismoutoui* | Trois bassins | NA | 0 | NA |
| **17_0402** | *Mormopterus francoismoutoui* | Trois bassins | NA | 0 | NA |
| **17_0403** | *Mormopterus francoismoutoui* | Trois bassins | NA | 0 | NA |
| **17_0404** | *Mormopterus francoismoutoui* | Trois bassins | NA | 0 | NA |
| **17_0405** | *Mormopterus francoismoutoui* | Trois bassins | NA | 0 | NA |
| **17_0406** | *Mormopterus francoismoutoui* | Trois bassins | NA | 0 | NA |
| **17_0407** | *Mormopterus francoismoutoui* | Trois bassins | NA | 0 | NA |
| **17_0408** | *Mormopterus francoismoutoui* | Trois bassins | NA | 0 | NA |
| **17_0409** | *Mormopterus francoismoutoui* | Trois bassins | NA | 0 | NA |
| **17_0410** | *Mormopterus francoismoutoui* | Trois bassins | NA | 0 | NA |
| **17_0411** | *Mormopterus francoismoutoui* | Trois bassins | NA | 0 | NA |
| **17_0412** | *Mormopterus francoismoutoui* | Trois bassins | NA | 0 | NA |
| **17_0413** | *Mormopterus francoismoutoui* | Trois bassins | NA | 0 | NA |
| **17_0414** | *Mormopterus francoismoutoui* | Trois bassins | NA | 0 | NA |
| **17_0415** | *Mormopterus francoismoutoui* | Trois bassins | NA | 0 | NA |
| **17_0416** | *Mormopterus francoismoutoui* | Trois bassins | NA | 0 | NA |
| **17_0417** | *Mormopterus francoismoutoui* | Trois bassins | NA | 0 | NA |
| **17_0418** | *Mormopterus francoismoutoui* | Trois bassins | NA | 0 | NA |
| **17_0419** | *Mormopterus francoismoutoui* | Trois bassins | NA | 0 | NA |
| **17_0420** | *Mormopterus francoismoutoui* | Trois bassins | NA | 0 | NA |
| **17_0421** | *Mormopterus francoismoutoui* | Trois bassins | NA | 0 | NA |
| **17_0432** | *Mormopterus francoismoutoui* | Trois bassins | NA | 0 | NA |
| **17_0433** | *Mormopterus francoismoutoui* | Trois bassins | NA | 0 | NA |
| **17_0434** | *Mormopterus francoismoutoui* | Trois bassins | NA | 0 | NA |

| | | | | | |
|---|---|---|---|---|---|
| **17_0435** | *Mormopterus francoismoutoui* | Trois bassins | NA | 0 | NA |
| **17_0436** | *Mormopterus francoismoutoui* | Trois bassins | NA | 0 | NA |
| **17_0437** | *Mormopterus francoismoutoui* | Trois bassins | NA | 0 | NA |
| **17_0438** | *Mormopterus francoismoutoui* | Trois bassins | NA | 0 | NA |
| **17_0439** | *Mormopterus francoismoutoui* | Trois bassins | NA | 0 | NA |
| **17_0440** | *Mormopterus francoismoutoui* | Trois bassins | NA | 0 | NA |
| **17_0441** | *Mormopterus francoismoutoui* | Trois bassins | NA | 1 | OR752492 |
| **17_0442** | *Mormopterus francoismoutoui* | Trois bassins | NA | 0 | NA |
| **17_0443** | *Mormopterus francoismoutoui* | Trois bassins | NA | 0 | NA |
| **17_0444** | *Mormopterus francoismoutoui* | Trois bassins | NA | 0 | NA |
| **17_0445** | *Mormopterus francoismoutoui* | Trois bassins | NA | 0 | NA |
| **17_0446** | *Mormopterus francoismoutoui* | Trois bassins | NA | 0 | NA |
| **17_0447** | *Mormopterus francoismoutoui* | Trois bassins | NA | 0 | NA |
| **17_0448** | *Mormopterus francoismoutoui* | Trois bassins | NA | 0 | NA |
| **17_0449** | *Mormopterus francoismoutoui* | Trois bassins | NA | 0 | NA |
| **17_0450** | *Mormopterus francoismoutoui* | Trois bassins | NA | 0 | NA |
| **17_0451** | *Mormopterus francoismoutoui* | Trois bassins | NA | 0 | NA |
| **17_0452** | *Mormopterus francoismoutoui* | Trois bassins | NA | 0 | NA |
| **17_0453** | *Mormopterus francoismoutoui* | Trois bassins | NA | 0 | NA |
| **17_0454** | *Mormopterus francoismoutoui* | Trois bassins | NA | 0 | NA |
| **17_0455** | *Mormopterus francoismoutoui* | Trois bassins | NA | 0 | NA |
| **17_0456** | *Mormopterus francoismoutoui* | Trois bassins | NA | 1 | OR752493 |
| **17_0457** | *Mormopterus francoismoutoui* | Trois bassins | NA | 0 | NA |
| **17_0458** | *Mormopterus francoismoutoui* | Trois bassins | NA | 0 | NA |
| **17_0459** | *Mormopterus francoismoutoui* | Trois bassins | NA | 0 | NA |
| **17_0460** | *Mormopterus francoismoutoui* | Trois bassins | NA | 0 | NA |
| **17_0461** | *Mormopterus francoismoutoui* | Trois bassins | NA | 1 | OR752494 |
| **17_0462** | *Mormopterus francoismoutoui* | Trois bassins | NA | 0 | NA |
| **17_0463** | *Mormopterus francoismoutoui* | Trois bassins | NA | 0 | NA |
| **17_0464** | *Mormopterus francoismoutoui* | Trois bassins | NA | 0 | NA |
| **17_0465** | *Mormopterus francoismoutoui* | Trois bassins | NA | 0 | NA |
| **17_0466** | *Mormopterus francoismoutoui* | Trois bassins | NA | 0 | NA |
| **17_0467** | *Mormopterus francoismoutoui* | Trois bassins | NA | 0 | NA |
| **17_0468** | *Mormopterus francoismoutoui* | Trois bassins | NA | 0 | NA |
| **17_0469** | *Mormopterus francoismoutoui* | Trois bassins | NA | 0 | NA |
| **17_0470** | *Mormopterus francoismoutoui* | Trois bassins | NA | 0 | NA |
| **17_0471** | *Mormopterus francoismoutoui* | Trois bassins | NA | 0 | NA |
| **17_0482** | *Mormopterus francoismoutoui* | Trois bassins | NA | 0 | NA |
| **17_0483** | *Mormopterus francoismoutoui* | Trois bassins | NA | 0 | NA |
| **17_0484** | *Mormopterus francoismoutoui* | Trois bassins | NA | 0 | NA |
| **17_0485** | *Mormopterus francoismoutoui* | Trois bassins | NA | 0 | NA |
| **17_0486** | *Mormopterus francoismoutoui* | Trois bassins | NA | 0 | NA |
| **17_0487** | *Mormopterus francoismoutoui* | Trois bassins | NA | 0 | NA |
| **17_0488** | *Mormopterus francoismoutoui* | Trois bassins | NA | 0 | NA |
| **17_0489** | *Mormopterus francoismoutoui* | Trois bassins | NA | 0 | NA |
| **17_0490** | *Mormopterus francoismoutoui* | Trois bassins | NA | 0 | NA |
| **17_0491** | *Mormopterus francoismoutoui* | Trois bassins | NA | 0 | NA |
| **17_0492** | *Mormopterus francoismoutoui* | Trois bassins | NA | 0 | NA |
| **17_0493** | *Mormopterus francoismoutoui* | Trois bassins | NA | 0 | NA |
| **17_0494** | *Mormopterus francoismoutoui* | Trois bassins | NA | 0 | NA |
| **17_0495** | *Mormopterus francoismoutoui* | Trois bassins | NA | 0 | NA |
| **17_0496** | *Mormopterus francoismoutoui* | Trois bassins | NA | 0 | NA |
| **17_0497** | *Mormopterus francoismoutoui* | Trois bassins | NA | 0 | NA |
| **17_0498** | *Mormopterus francoismoutoui* | Trois bassins | NA | 0 | NA |
| **17_0499** | *Mormopterus francoismoutoui* | Trois bassins | NA | 0 | NA |
| **17_0500** | *Mormopterus francoismoutoui* | Trois bassins | NA | 0 | NA |
| **17_0501** | *Mormopterus francoismoutoui* | Trois bassins | NA | 0 | NA |
| **17_0502** | *Mormopterus francoismoutoui* | Trois bassins | NA | 0 | NA |
| **17_0503** | *Mormopterus francoismoutoui* | Trois bassins | NA | 0 | NA |
| **17_0504** | *Mormopterus francoismoutoui* | Trois bassins | NA | 0 | NA |
| **17_0505** | *Mormopterus francoismoutoui* | Trois bassins | NA | 0 | NA |
| **17_0506** | *Mormopterus francoismoutoui* | Trois bassins | NA | 0 | NA |
| **17_0507** | *Mormopterus francoismoutoui* | Trois bassins | NA | 0 | NA |
| **17_0508** | *Mormopterus francoismoutoui* | Trois bassins | NA | 0 | NA |
| **17_0509** | *Mormopterus francoismoutoui* | Trois bassins | NA | 0 | NA |
| **17_0510** | *Mormopterus francoismoutoui* | Trois bassins | NA | 0 | NA |
| **17_0511** | *Mormopterus francoismoutoui* | Trois bassins | NA | 0 | NA |
| **17_0512** | *Mormopterus francoismoutoui* | Trois bassins | NA | 0 | NA |
| **17_0513** | *Mormopterus francoismoutoui* | Trois bassins | NA | 0 | NA |
| **17_0514** | *Mormopterus francoismoutoui* | Trois bassins | NA | 0 | NA |
| **17_0515** | *Mormopterus francoismoutoui* | Trois bassins | NA | 0 | NA |
| **17_0516** | *Mormopterus francoismoutoui* | Trois bassins | NA | 0 | NA |

| | | | | | |
|---|---|---|---|---|---|
| **17_0527** | *Mormopterus francoismoutoui* | Trois bassins | NA | 0 | NA |
| **17_0528** | *Mormopterus francoismoutoui* | Trois bassins | NA | 0 | NA |
| **17_0529** | *Mormopterus francoismoutoui* | Trois bassins | NA | 0 | NA |
| **17_0530** | *Mormopterus francoismoutoui* | Trois bassins | NA | 0 | NA |
| **17_0531** | *Mormopterus francoismoutoui* | Trois bassins | NA | 0 | NA |
| **17_0532** | *Mormopterus francoismoutoui* | Trois bassins | NA | 0 | NA |
| **17_0533** | *Mormopterus francoismoutoui* | Trois bassins | NA | 0 | NA |
| **17_0534** | *Mormopterus francoismoutoui* | Trois bassins | NA | 0 | NA |
| **17_0535** | *Mormopterus francoismoutoui* | Trois bassins | NA | 0 | NA |
| **17_0536** | *Mormopterus francoismoutoui* | Trois bassins | NA | 0 | NA |
| **17_0537** | *Mormopterus francoismoutoui* | Trois bassins | NA | 0 | NA |
| **17_0538** | *Mormopterus francoismoutoui* | Trois bassins | NA | 0 | NA |
| **17_0539** | *Mormopterus francoismoutoui* | Trois bassins | NA | 0 | NA |
| **17_0540** | *Mormopterus francoismoutoui* | Trois bassins | NA | 0 | NA |
| **17_0541** | *Mormopterus francoismoutoui* | Trois bassins | NA | 0 | NA |
| **17_0542** | *Mormopterus francoismoutoui* | Trois bassins | NA | 0 | NA |
| **17_0543** | *Mormopterus francoismoutoui* | Trois bassins | NA | 0 | NA |
| **17_0544** | *Mormopterus francoismoutoui* | Trois bassins | NA | 0 | NA |
| **17_0545** | *Mormopterus francoismoutoui* | Trois bassins | NA | 0 | NA |
| **17_0546** | *Mormopterus francoismoutoui* | Trois bassins | NA | 0 | NA |
| **17_0547** | *Mormopterus francoismoutoui* | Trois bassins | NA | 0 | NA |
| **17_0548** | *Mormopterus francoismoutoui* | Trois bassins | NA | 0 | NA |
| **17_0549** | *Mormopterus francoismoutoui* | Trois bassins | NA | 0 | NA |
| **17_0550** | *Mormopterus francoismoutoui* | Trois bassins | NA | 0 | NA |
| **17_0551** | *Mormopterus francoismoutoui* | Trois bassins | NA | 0 | NA |
| **17_1091** | *Mormopterus francoismoutoui* | Trois bassins | NA | 0 | NA |
| **17_1092** | *Mormopterus francoismoutoui* | Trois bassins | NA | 0 | NA |
| **17_1093** | *Mormopterus francoismoutoui* | Trois bassins | NA | 0 | NA |
| **17_1094** | *Mormopterus francoismoutoui* | Trois bassins | NA | 0 | NA |
| **17_1095** | *Mormopterus francoismoutoui* | Trois bassins | NA | 0 | NA |
| **17_1096** | *Mormopterus francoismoutoui* | Trois bassins | NA | 0 | NA |
| **17_1097** | *Mormopterus francoismoutoui* | Trois bassins | NA | 0 | NA |
| **17_1098** | *Mormopterus francoismoutoui* | Trois bassins | NA | 0 | NA |
| **17_1099** | *Mormopterus francoismoutoui* | Trois bassins | NA | 0 | NA |
| **17_1100** | *Mormopterus francoismoutoui* | Trois bassins | NA | 0 | NA |
| **17_1101** | *Mormopterus francoismoutoui* | Trois bassins | NA | 0 | NA |
| **17_1102** | *Mormopterus francoismoutoui* | Trois bassins | NA | 0 | NA |
| **17_1103** | *Mormopterus francoismoutoui* | Trois bassins | NA | 0 | NA |
| **17_1104** | *Mormopterus francoismoutoui* | Trois bassins | NA | 0 | NA |
| **17_1105** | *Mormopterus francoismoutoui* | Trois bassins | NA | 0 | NA |
| **17_1106** | *Mormopterus francoismoutoui* | Trois bassins | NA | 0 | NA |
| **17_1107** | *Mormopterus francoismoutoui* | Trois bassins | NA | 0 | NA |
| **17_1108** | *Mormopterus francoismoutoui* | Trois bassins | NA | 0 | NA |
| **17_1109** | *Mormopterus francoismoutoui* | Trois bassins | NA | 0 | NA |
| **17_1110** | *Mormopterus francoismoutoui* | Trois bassins | NA | 0 | NA |
| **17_1111** | *Mormopterus francoismoutoui* | Trois bassins | NA | 0 | NA |
| **17_1112** | *Mormopterus francoismoutoui* | Trois bassins | NA | 0 | NA |
| **17_1113** | *Mormopterus francoismoutoui* | Trois bassins | NA | 0 | NA |
| **17_1114** | *Mormopterus francoismoutoui* | Trois bassins | NA | 0 | NA |
| **17_1115** | *Mormopterus francoismoutoui* | Trois bassins | NA | 0 | NA |
| **17_1116** | *Mormopterus francoismoutoui* | Trois bassins | NA | 0 | NA |
| **17_1117** | *Mormopterus francoismoutoui* | Trois bassins | NA | 0 | NA |
| **17_1118** | *Mormopterus francoismoutoui* | Trois bassins | NA | 0 | NA |
| **17_1119** | *Mormopterus francoismoutoui* | Trois bassins | NA | 0 | NA |
| **17_1120** | *Mormopterus francoismoutoui* | Trois bassins | NA | 0 | NA |
| **17_1121** | *Mormopterus francoismoutoui* | Transformateur EDF Gillot | NA | 0 | NA |
| **17_1122** | *Mormopterus francoismoutoui* | Transformateur EDF Gillot | NA | 0 | NA |
| **17_1123** | *Mormopterus francoismoutoui* | Transformateur EDF Gillot | NA | 0 | NA |
| **17_1124** | *Mormopterus francoismoutoui* | Transformateur EDF Gillot | NA | 0 | NA |
| **17_1125** | *Mormopterus francoismoutoui* | Transformateur EDF Gillot | NA | 0 | NA |
| **17_1126** | *Mormopterus francoismoutoui* | Transformateur EDF Gillot | NA | 0 | NA |
| **17_1127** | *Mormopterus francoismoutoui* | Transformateur EDF Gillot | NA | 0 | NA |
| **17_1128** | *Mormopterus francoismoutoui* | Transformateur EDF Gillot | NA | 0 | NA |
| **17_1129** | *Mormopterus francoismoutoui* | Transformateur EDF Gillot | NA | 0 | NA |
| **17_1130** | *Mormopterus francoismoutoui* | Transformateur EDF Gillot | NA | 0 | NA |
| **17_1131** | *Mormopterus francoismoutoui* | Transformateur EDF Gillot | NA | 0 | NA |
| **17_1132** | *Mormopterus francoismoutoui* | Transformateur EDF Gillot | NA | 0 | NA |
| **17_1133** | *Mormopterus francoismoutoui* | Transformateur EDF Gillot | NA | 0 | NA |
| **17_1134** | *Mormopterus francoismoutoui* | Transformateur EDF Gillot | NA | 0 | NA |
| **17_1135** | *Mormopterus francoismoutoui* | Transformateur EDF Gillot | NA | 0 | NA |
| **17_1136** | *Mormopterus francoismoutoui* | Transformateur EDF Gillot | NA | 0 | NA |
| **17_1137** | *Mormopterus francoismoutoui* | Transformateur EDF Gillot | NA | 0 | NA |

| | | | | | |
|---|---|---|---|---|---|
| **17_1138** | *Mormopterus francoismoutoui* | Transformateur EDF Gillot | NA | 0 | NA |
| **17_1139** | *Mormopterus francoismoutoui* | Transformateur EDF Gillot | NA | 0 | NA |
| **17_1140** | *Mormopterus francoismoutoui* | Transformateur EDF Gillot | NA | 0 | NA |
| **17_1141** | *Mormopterus francoismoutoui* | Transformateur EDF Gillot | NA | 0 | NA |
| **17_1142** | *Mormopterus francoismoutoui* | Transformateur EDF Gillot | NA | 0 | NA |
| **17_1143** | *Mormopterus francoismoutoui* | Transformateur EDF Gillot | NA | 0 | NA |
| **17_1144** | *Mormopterus francoismoutoui* | Transformateur EDF Gillot | NA | 0 | NA |
| **17_1145** | *Mormopterus francoismoutoui* | Transformateur EDF Gillot | NA | 0 | NA |
| **17_1146** | *Mormopterus francoismoutoui* | Transformateur EDF Gillot | NA | 0 | NA |
| **17_1147** | *Mormopterus francoismoutoui* | Transformateur EDF Gillot | NA | 0 | NA |
| **17_1148** | *Mormopterus francoismoutoui* | Transformateur EDF Gillot | NA | 0 | NA |
| **17_1149** | *Mormopterus francoismoutoui* | Transformateur EDF Gillot | NA | 0 | NA |
| **17_1150** | *Mormopterus francoismoutoui* | Transformateur EDF Gillot | NA | 0 | NA |
| **17_1161** | *Mormopterus francoismoutoui* | Trois bassins | NA | 0 | NA |
| **17_1162** | *Mormopterus francoismoutoui* | Trois bassins | NA | 0 | NA |
| **17_1163** | *Mormopterus francoismoutoui* | Trois bassins | NA | 0 | NA |
| **17_1164** | *Mormopterus francoismoutoui* | Trois bassins | NA | 0 | NA |
| **17_1165** | *Mormopterus francoismoutoui* | Trois bassins | NA | 0 | NA |
| **17_1166** | *Mormopterus francoismoutoui* | Trois bassins | NA | 0 | NA |
| **17_1167** | *Mormopterus francoismoutoui* | Trois bassins | NA | 0 | NA |
| **17_1168** | *Mormopterus francoismoutoui* | Trois bassins | NA | 0 | NA |
| **17_1169** | *Mormopterus francoismoutoui* | Trois bassins | NA | 0 | NA |
| **17_1170** | *Mormopterus francoismoutoui* | Trois bassins | NA | 0 | NA |
| **17_1171** | *Mormopterus francoismoutoui* | Trois bassins | NA | 0 | NA |
| **17_1172** | *Mormopterus francoismoutoui* | Trois bassins | NA | 0 | NA |
| **17_1173** | *Mormopterus francoismoutoui* | Trois bassins | NA | 0 | NA |
| **17_1174** | *Mormopterus francoismoutoui* | Trois bassins | NA | 0 | NA |
| **17_1175** | *Mormopterus francoismoutoui* | Trois bassins | NA | 0 | NA |
| **17_1176** | *Mormopterus francoismoutoui* | Trois bassins | NA | 0 | NA |
| **17_1177** | *Mormopterus francoismoutoui* | Trois bassins | NA | 0 | NA |
| **17_1178** | *Mormopterus francoismoutoui* | Trois bassins | NA | 0 | NA |
| **17_1179** | *Mormopterus francoismoutoui* | Trois bassins | NA | 0 | NA |
| **17_1180** | *Mormopterus francoismoutoui* | Trois bassins | NA | 0 | NA |
| **17_1181** | *Mormopterus francoismoutoui* | Trois bassins | NA | 0 | NA |
| **17_1182** | *Mormopterus francoismoutoui* | Trois bassins | NA | 0 | NA |
| **17_1183** | *Mormopterus francoismoutoui* | Trois bassins | NA | 0 | NA |
| **17_1184** | *Mormopterus francoismoutoui* | Trois bassins | NA | 1 | OR752495 |
| **17_1185** | *Mormopterus francoismoutoui* | Trois bassins | NA | 0 | NA |
| **17_1186** | *Mormopterus francoismoutoui* | Trois bassins | NA | 0 | NA |
| **17_1187** | *Mormopterus francoismoutoui* | Trois bassins | NA | 0 | NA |
| **17_1188** | *Mormopterus francoismoutoui* | Trois bassins | NA | 0 | NA |
| **17_1189** | *Mormopterus francoismoutoui* | Trois bassins | NA | 0 | NA |
| **17_1190** | *Mormopterus francoismoutoui* | Trois bassins | NA | 0 | NA |
| **17_1191** | *Mormopterus francoismoutoui* | Trois bassins | NA | 0 | NA |
| **17_1192** | *Mormopterus francoismoutoui* | Trois bassins | NA | 0 | NA |
| **17_1193** | *Mormopterus francoismoutoui* | Trois bassins | NA | 0 | NA |
| **17_1194** | *Mormopterus francoismoutoui* | Trois bassins | NA | 0 | NA |
| **17_1195** | *Mormopterus francoismoutoui* | Trois bassins | NA | 0 | NA |
| **17_1196** | *Mormopterus francoismoutoui* | Trois bassins | NA | 0 | NA |
| **17_1197** | *Mormopterus francoismoutoui* | Trois bassins | NA | 0 | NA |
| **17_1198** | *Mormopterus francoismoutoui* | Trois bassins | NA | 0 | NA |
| **17_1199** | *Mormopterus francoismoutoui* | Trois bassins | NA | 0 | NA |
| **17_1200** | *Mormopterus francoismoutoui* | Trois bassins | NA | 0 | NA |
| **17_1201** | *Mormopterus francoismoutoui* | Trois bassins | NA | 0 | NA |
| **17_1202** | *Mormopterus francoismoutoui* | Trois bassins | NA | 0 | NA |
| **17_1203** | *Mormopterus francoismoutoui* | Trois bassins | NA | 0 | NA |
| **17_1204** | *Mormopterus francoismoutoui* | Trois bassins | NA | 0 | NA |
| **17_1205** | *Mormopterus francoismoutoui* | Trois bassins | NA | 0 | NA |
| **17_1206** | *Mormopterus francoismoutoui* | Trois bassins | NA | 0 | NA |
| **17_1207** | *Mormopterus francoismoutoui* | Trois bassins | NA | 0 | NA |
| **17_1208** | *Mormopterus francoismoutoui* | Trois bassins | NA | 0 | NA |
| **17_1209** | *Mormopterus francoismoutoui* | Trois bassins | NA | 0 | NA |
| **17_1210** | *Mormopterus francoismoutoui* | Trois bassins | NA | 0 | NA |
| **17_1211** | *Mormopterus francoismoutoui* | Transformateur EDF Gillot | NA | 0 | NA |
| **17_1212** | *Mormopterus francoismoutoui* | Transformateur EDF Gillot | NA | 0 | NA |
| **17_1213** | *Mormopterus francoismoutoui* | Transformateur EDF Gillot | NA | 0 | NA |
| **17_1214** | *Mormopterus francoismoutoui* | Transformateur EDF Gillot | NA | 0 | NA |
| **17_1215** | *Mormopterus francoismoutoui* | Transformateur EDF Gillot | NA | 0 | NA |
| **17_1216** | *Mormopterus francoismoutoui* | Transformateur EDF Gillot | NA | 0 | NA |
| **17_1217** | *Mormopterus francoismoutoui* | Transformateur EDF Gillot | NA | 0 | NA |
| **17_1218** | *Mormopterus francoismoutoui* | Transformateur EDF Gillot | NA | 0 | NA |
| **17_1219** | *Mormopterus francoismoutoui* | Transformateur EDF Gillot | NA | 0 | NA |

| | | | | | |
|---|---|---|---|---|---|
| **17_1220** | *Mormopterus francoismoutoui* | Transformateur EDF Gillot | NA | 0 | NA |
| **17_1221** | *Mormopterus francoismoutoui* | Transformateur EDF Gillot | NA | 0 | NA |
| **17_1222** | *Mormopterus francoismoutoui* | Transformateur EDF Gillot | NA | 0 | NA |
| **17_1223** | *Mormopterus francoismoutoui* | Transformateur EDF Gillot | NA | 0 | NA |
| **17_1224** | *Mormopterus francoismoutoui* | Transformateur EDF Gillot | NA | 0 | NA |
| **17_1225** | *Mormopterus francoismoutoui* | Transformateur EDF Gillot | NA | 0 | NA |
| **17_1226** | *Mormopterus francoismoutoui* | Transformateur EDF Gillot | NA | 0 | NA |
| **17_1227** | *Mormopterus francoismoutoui* | Transformateur EDF Gillot | NA | 0 | NA |
| **17_1228** | *Mormopterus francoismoutoui* | Transformateur EDF Gillot | NA | 0 | NA |
| **17_1229** | *Mormopterus francoismoutoui* | Transformateur EDF Gillot | NA | 0 | NA |
| **17_1230** | *Mormopterus francoismoutoui* | Transformateur EDF Gillot | NA | 0 | NA |
| **17_1231** | *Mormopterus francoismoutoui* | Transformateur EDF Gillot | NA | 0 | NA |
| **17_1232** | *Mormopterus francoismoutoui* | Transformateur EDF Gillot | NA | 0 | NA |
| **17_1233** | *Mormopterus francoismoutoui* | Transformateur EDF Gillot | NA | 0 | NA |
| **17_1234** | *Mormopterus francoismoutoui* | Transformateur EDF Gillot | NA | 0 | NA |
| **17_1235** | *Mormopterus francoismoutoui* | Transformateur EDF Gillot | NA | 0 | NA |
| **17_1236** | *Mormopterus francoismoutoui* | Transformateur EDF Gillot | NA | 0 | NA |
| **17_1237** | *Mormopterus francoismoutoui* | Transformateur EDF Gillot | NA | 0 | NA |
| **17_1238** | *Mormopterus francoismoutoui* | Transformateur EDF Gillot | NA | 0 | NA |
| **17_1239** | *Mormopterus francoismoutoui* | Transformateur EDF Gillot | NA | 0 | NA |
| **17_1240** | *Mormopterus francoismoutoui* | Transformateur EDF Gillot | NA | 0 | NA |
| **17_1251** | *Mormopterus francoismoutoui* | Trois bassins | NA | 0 | NA |
| **17_1252** | *Mormopterus francoismoutoui* | Trois bassins | NA | 0 | NA |
| **17_1253** | *Mormopterus francoismoutoui* | Trois bassins | NA | 0 | NA |
| **17_1254** | *Mormopterus francoismoutoui* | Trois bassins | NA | 1 | OR752496 |
| **17_1255** | *Mormopterus francoismoutoui* | Trois bassins | NA | 0 | NA |
| **17_1256** | *Mormopterus francoismoutoui* | Trois bassins | NA | 0 | NA |
| **17_1257** | *Mormopterus francoismoutoui* | Trois bassins | NA | 0 | NA |
| **17_1258** | *Mormopterus francoismoutoui* | Trois bassins | NA | 1 | OR752497 |
| **17_1259** | *Mormopterus francoismoutoui* | Trois bassins | NA | 0 | NA |
| **17_1260** | *Mormopterus francoismoutoui* | Trois bassins | NA | 0 | NA |
| **17_1261** | *Mormopterus francoismoutoui* | Trois bassins | NA | 0 | NA |
| **17_1262** | *Mormopterus francoismoutoui* | Trois bassins | NA | 0 | NA |
| **17_1263** | *Mormopterus francoismoutoui* | Trois bassins | NA | 0 | NA |
| **17_1264** | *Mormopterus francoismoutoui* | Trois bassins | NA | 0 | NA |
| **17_1265** | *Mormopterus francoismoutoui* | Trois bassins | NA | 0 | NA |
| **17_1266** | *Mormopterus francoismoutoui* | Trois bassins | NA | 0 | NA |
| **17_1267** | *Mormopterus francoismoutoui* | Trois bassins | NA | 0 | NA |
| **17_1268** | *Mormopterus francoismoutoui* | Trois bassins | NA | 0 | NA |
| **17_1269** | *Mormopterus francoismoutoui* | Trois bassins | NA | 0 | NA |
| **17_1270** | *Mormopterus francoismoutoui* | Trois bassins | NA | 0 | NA |
| **17_1271** | *Mormopterus francoismoutoui* | Trois bassins | NA | 0 | NA |
| **17_1272** | *Mormopterus francoismoutoui* | Trois bassins | NA | 0 | NA |
| **17_1273** | *Mormopterus francoismoutoui* | Trois bassins | NA | 0 | NA |
| **17_1274** | *Mormopterus francoismoutoui* | Trois bassins | NA | 0 | NA |
| **17_1275** | *Mormopterus francoismoutoui* | Trois bassins | NA | 0 | NA |
| **17_1276** | *Mormopterus francoismoutoui* | Trois bassins | NA | 0 | NA |
| **17_1277** | *Mormopterus francoismoutoui* | Trois bassins | NA | 0 | NA |
| **17_1278** | *Mormopterus francoismoutoui* | Trois bassins | NA | 0 | NA |
| **17_1279** | *Mormopterus francoismoutoui* | Trois bassins | NA | 0 | NA |
| **17_1280** | *Mormopterus francoismoutoui* | Trois bassins | NA | 0 | NA |
| **17_1281** | *Mormopterus francoismoutoui* | Trois bassins | NA | 0 | NA |
| **17_1282** | *Mormopterus francoismoutoui* | Trois bassins | NA | 0 | NA |
| **17_1283** | *Mormopterus francoismoutoui* | Trois bassins | NA | 0 | NA |
| **17_1284** | *Mormopterus francoismoutoui* | Trois bassins | NA | 0 | NA |
| **17_1285** | *Mormopterus francoismoutoui* | Trois bassins | NA | 0 | NA |
| **17_1286** | *Mormopterus francoismoutoui* | Trois bassins | NA | 0 | NA |
| **17_1287** | *Mormopterus francoismoutoui* | Trois bassins | NA | 0 | NA |
| **17_1288** | *Mormopterus francoismoutoui* | Trois bassins | NA | 0 | NA |
| **17_1289** | *Mormopterus francoismoutoui* | Trois bassins | NA | 0 | NA |
| **17_1290** | *Mormopterus francoismoutoui* | Trois bassins | NA | 1 | OR752498 |
| **17_1291** | *Mormopterus francoismoutoui* | Trois bassins | NA | 0 | NA |
| **17_1292** | *Mormopterus francoismoutoui* | Trois bassins | NA | 0 | NA |
| **17_1293** | *Mormopterus francoismoutoui* | Trois bassins | NA | 0 | NA |
| **17_1294** | *Mormopterus francoismoutoui* | Trois bassins | NA | 0 | NA |
| **17_1295** | *Mormopterus francoismoutoui* | Trois bassins | NA | 0 | NA |
| **17_1296** | *Mormopterus francoismoutoui* | Trois bassins | NA | 0 | NA |
| **17_1297** | *Mormopterus francoismoutoui* | Trois bassins | NA | 0 | NA |
| **17_1298** | *Mormopterus francoismoutoui* | Trois bassins | NA | 0 | NA |
| **17_1299** | *Mormopterus francoismoutoui* | Trois bassins | NA | 0 | NA |
| **17_1300** | *Mormopterus francoismoutoui* | Trois bassins | NA | 0 | NA |
| **17_1301** | *Mormopterus francoismoutoui* | Transformateur EDF Gillot | NA | 0 | NA |

| | | | | | |
|---|---|---|---|---|---|
| **17_1302** | *Mormopterus francoismoutoui* | Transformateur EDF Gillot | NA | 0 | NA |
| **17_1303** | *Mormopterus francoismoutoui* | Transformateur EDF Gillot | NA | 0 | NA |
| **17_1304** | *Mormopterus francoismoutoui* | Transformateur EDF Gillot | NA | 0 | NA |
| **17_1305** | *Mormopterus francoismoutoui* | Transformateur EDF Gillot | NA | 0 | NA |
| **17_1306** | *Mormopterus francoismoutoui* | Transformateur EDF Gillot | NA | 0 | NA |
| **17_1307** | *Mormopterus francoismoutoui* | Transformateur EDF Gillot | NA | 0 | NA |
| **17_1308** | *Mormopterus francoismoutoui* | Transformateur EDF Gillot | NA | 0 | NA |
| **17_1309** | *Mormopterus francoismoutoui* | Transformateur EDF Gillot | NA | 0 | NA |
| **17_1310** | *Mormopterus francoismoutoui* | Transformateur EDF Gillot | NA | 1 | OR752499 |
| **17_1311** | *Mormopterus francoismoutoui* | Transformateur EDF Gillot | NA | 0 | NA |
| **17_1312** | *Mormopterus francoismoutoui* | Transformateur EDF Gillot | NA | 0 | NA |
| **17_1313** | *Mormopterus francoismoutoui* | Transformateur EDF Gillot | NA | 0 | NA |
| **17_1314** | *Mormopterus francoismoutoui* | Transformateur EDF Gillot | NA | 0 | NA |
| **17_1315** | *Mormopterus francoismoutoui* | Transformateur EDF Gillot | NA | 0 | NA |
| **17_1316** | *Mormopterus francoismoutoui* | Transformateur EDF Gillot | NA | 0 | NA |
| **17_1317** | *Mormopterus francoismoutoui* | Transformateur EDF Gillot | NA | 0 | NA |
| **17_1318** | *Mormopterus francoismoutoui* | Transformateur EDF Gillot | NA | 0 | NA |
| **17_1319** | *Mormopterus francoismoutoui* | Transformateur EDF Gillot | NA | 0 | NA |
| **17_1320** | *Mormopterus francoismoutoui* | Transformateur EDF Gillot | NA | 0 | NA |
| **17_1321** | *Mormopterus francoismoutoui* | Transformateur EDF Gillot | NA | 0 | NA |
| **17_1322** | *Mormopterus francoismoutoui* | Transformateur EDF Gillot | NA | 0 | NA |
| **17_1323** | *Mormopterus francoismoutoui* | Transformateur EDF Gillot | NA | 0 | NA |
| **17_1324** | *Mormopterus francoismoutoui* | Transformateur EDF Gillot | NA | 0 | NA |
| **17_1325** | *Mormopterus francoismoutoui* | Transformateur EDF Gillot | NA | 0 | NA |
| **17_1326** | *Mormopterus francoismoutoui* | Transformateur EDF Gillot | NA | 0 | NA |
| **17_1327** | *Mormopterus francoismoutoui* | Transformateur EDF Gillot | NA | 0 | NA |
| **17_1328** | *Mormopterus francoismoutoui* | Transformateur EDF Gillot | NA | 0 | NA |
| **17_1329** | *Mormopterus francoismoutoui* | Transformateur EDF Gillot | NA | 0 | NA |
| **17_1330** | *Mormopterus francoismoutoui* | Transformateur EDF Gillot | NA | 0 | NA |
| **18_0001** | *Mormopterus francoismoutoui* | Transformateur EDF Gillot | NA | 0 | NA |
| **18_0002** | *Mormopterus francoismoutoui* | Transformateur EDF Gillot | NA | 0 | NA |
| **18_0003** | *Mormopterus francoismoutoui* | Transformateur EDF Gillot | NA | 0 | NA |
| **18_0004** | *Mormopterus francoismoutoui* | Transformateur EDF Gillot | NA | 0 | NA |
| **18_0005** | *Mormopterus francoismoutoui* | Transformateur EDF Gillot | NA | 0 | NA |
| **18_0006** | *Mormopterus francoismoutoui* | Transformateur EDF Gillot | NA | 0 | NA |
| **18_0007** | *Mormopterus francoismoutoui* | Transformateur EDF Gillot | NA | 0 | NA |
| **18_0008** | *Mormopterus francoismoutoui* | Transformateur EDF Gillot | NA | 1 | OR752500 |
| **18_0009** | *Mormopterus francoismoutoui* | Transformateur EDF Gillot | NA | 0 | NA |
| **18_0010** | *Mormopterus francoismoutoui* | Transformateur EDF Gillot | NA | 0 | NA |
| **18_0011** | *Mormopterus francoismoutoui* | Transformateur EDF Gillot | NA | 0 | NA |
| **18_0012** | *Mormopterus francoismoutoui* | Transformateur EDF Gillot | NA | 0 | NA |
| **18_0013** | *Mormopterus francoismoutoui* | Transformateur EDF Gillot | NA | 0 | NA |
| **18_0014** | *Mormopterus francoismoutoui* | Transformateur EDF Gillot | NA | 0 | NA |
| **18_0015** | *Mormopterus francoismoutoui* | Transformateur EDF Gillot | NA | 0 | NA |
| **18_0026** | *Mormopterus francoismoutoui* | Trois bassins | NA | 0 | NA |
| **18_0027** | *Mormopterus francoismoutoui* | Trois bassins | NA | 0 | NA |
| **18_0028** | *Mormopterus francoismoutoui* | Trois bassins | NA | 0 | NA |
| **18_0029** | *Mormopterus francoismoutoui* | Trois bassins | NA | 0 | NA |
| **18_0030** | *Mormopterus francoismoutoui* | Trois bassins | NA | 0 | NA |
| **18_0031** | *Mormopterus francoismoutoui* | Trois bassins | NA | 0 | NA |
| **18_0032** | *Mormopterus francoismoutoui* | Trois bassins | NA | 0 | NA |
| **18_0033** | *Mormopterus francoismoutoui* | Trois bassins | NA | 0 | NA |
| **18_0034** | *Mormopterus francoismoutoui* | Trois bassins | NA | 0 | NA |
| **18_0035** | *Mormopterus francoismoutoui* | Trois bassins | NA | 0 | NA |
| **18_0036** | *Mormopterus francoismoutoui* | Trois bassins | NA | 0 | NA |
| **18_0037** | *Mormopterus francoismoutoui* | Trois bassins | NA | 0 | NA |
| **18_0038** | *Mormopterus francoismoutoui* | Trois bassins | NA | 0 | NA |
| **18_0039** | *Mormopterus francoismoutoui* | Trois bassins | NA | 0 | NA |
| **18_0040** | *Mormopterus francoismoutoui* | Trois bassins | NA | 0 | NA |
| **18_0041** | *Mormopterus francoismoutoui* | Trois bassins | NA | 0 | NA |
| **18_0042** | *Mormopterus francoismoutoui* | Trois bassins | NA | 0 | NA |
| **18_0043** | *Mormopterus francoismoutoui* | Trois bassins | NA | 0 | NA |
| **18_0044** | *Mormopterus francoismoutoui* | Trois bassins | NA | 0 | NA |
| **18_0045** | *Mormopterus francoismoutoui* | Trois bassins | NA | 0 | NA |
| **18_0046** | *Mormopterus francoismoutoui* | Trois bassins | NA | 0 | NA |
| **18_0047** | *Mormopterus francoismoutoui* | Trois bassins | NA | 0 | NA |
| **18_0048** | *Mormopterus francoismoutoui* | Trois bassins | NA | 0 | NA |
| **18_0049** | *Mormopterus francoismoutoui* | Trois bassins | NA | 0 | NA |
| **18_0050** | *Mormopterus francoismoutoui* | Trois bassins | NA | 0 | NA |
| **18_0051** | *Mormopterus francoismoutoui* | Trois bassins | NA | 0 | NA |
| **18_0052** | *Mormopterus francoismoutoui* | Trois bassins | NA | 0 | NA |
| **18_0053** | *Mormopterus francoismoutoui* | Trois bassins | NA | 0 | NA |

| | | | | | |
|---|---|---|---|---|---|
| **18_0054** | *Mormopterus francoismoutoui* | Trois bassins | NA | 0 | NA |
| **18_0055** | *Mormopterus francoismoutoui* | Trois bassins | NA | 0 | NA |
| **18_0056** | *Mormopterus francoismoutoui* | Trois bassins | NA | 0 | NA |
| **18_0057** | *Mormopterus francoismoutoui* | Trois bassins | NA | 0 | NA |
| **18_0058** | *Mormopterus francoismoutoui* | Trois bassins | NA | 0 | NA |
| **18_0059** | *Mormopterus francoismoutoui* | Trois bassins | NA | 0 | NA |
| **18_0070** | *Mormopterus francoismoutoui* | Trois bassins | NA | 0 | NA |
| **18_0071** | *Mormopterus francoismoutoui* | Trois bassins | NA | 0 | NA |
| **18_0072** | *Mormopterus francoismoutoui* | Trois bassins | NA | 0 | NA |
| **18_0073** | *Mormopterus francoismoutoui* | Trois bassins | NA | 0 | NA |
| **18_0074** | *Mormopterus francoismoutoui* | Trois bassins | NA | 0 | NA |
| **18_0075** | *Mormopterus francoismoutoui* | Trois bassins | NA | 0 | NA |
| **18_0076** | *Mormopterus francoismoutoui* | Trois bassins | NA | 0 | NA |
| **18_0077** | *Mormopterus francoismoutoui* | Trois bassins | NA | 0 | NA |
| **18_0078** | *Mormopterus francoismoutoui* | Trois bassins | NA | 0 | NA |
| **18_0079** | *Mormopterus francoismoutoui* | Trois bassins | NA | 0 | NA |
| **18_0080** | *Mormopterus francoismoutoui* | Trois bassins | NA | 0 | NA |
| **18_0081** | *Mormopterus francoismoutoui* | Trois bassins | NA | 0 | NA |
| **18_0082** | *Mormopterus francoismoutoui* | Trois bassins | NA | 0 | NA |
| **18_0083** | *Mormopterus francoismoutoui* | Trois bassins | NA | 0 | NA |
| **18_0084** | *Mormopterus francoismoutoui* | Trois bassins | NA | 0 | NA |
| **18_0085** | *Mormopterus francoismoutoui* | Trois bassins | NA | 0 | NA |
| **18_0086** | *Mormopterus francoismoutoui* | Trois bassins | NA | 0 | NA |
| **18_0087** | *Mormopterus francoismoutoui* | Trois bassins | NA | 0 | NA |
| **18_0088** | *Mormopterus francoismoutoui* | Trois bassins | NA | 0 | NA |
| **18_0089** | *Mormopterus francoismoutoui* | Trois bassins | NA | 0 | NA |
| **18_0090** | *Mormopterus francoismoutoui* | Trois bassins | NA | 0 | NA |
| **18_0091** | *Mormopterus francoismoutoui* | Trois bassins | NA | 0 | NA |
| **18_0092** | *Mormopterus francoismoutoui* | Trois bassins | NA | 0 | NA |
| **18_0093** | *Mormopterus francoismoutoui* | Trois bassins | NA | 0 | NA |
| **18_0094** | *Mormopterus francoismoutoui* | Trois bassins | NA | 0 | NA |
| **18_0095** | *Mormopterus francoismoutoui* | Trois bassins | NA | 0 | NA |
| **18_0096** | *Mormopterus francoismoutoui* | Trois bassins | NA | 0 | NA |
| **18_0097** | *Mormopterus francoismoutoui* | Trois bassins | NA | 0 | NA |
| **18_0098** | *Mormopterus francoismoutoui* | Trois bassins | NA | 0 | NA |
| **18_0099** | *Mormopterus francoismoutoui* | Trois bassins | NA | 0 | NA |
| **18_0100** | *Mormopterus francoismoutoui* | Trois bassins | NA | 0 | NA |
| **18_0101** | *Mormopterus francoismoutoui* | Trois bassins | NA | 0 | NA |
| **18_0102** | *Mormopterus francoismoutoui* | Trois bassins | NA | 0 | NA |
| **18_0103** | *Mormopterus francoismoutoui* | Trois bassins | NA | 1 | OR752501 |
| **18_0104** | *Mormopterus francoismoutoui* | Trois bassins | NA | 1 | OR752502 |
| **18_0105** | *Mormopterus francoismoutoui* | Trois bassins | NA | 0 | NA |
| **18_0106** | *Mormopterus francoismoutoui* | Trois bassins | NA | 0 | NA |
| **18_0107** | *Mormopterus francoismoutoui* | Trois bassins | NA | 0 | NA |
| **18_0108** | *Mormopterus francoismoutoui* | Trois bassins | NA | 0 | NA |
| **18_0109** | *Mormopterus francoismoutoui* | Trois bassins | NA | 0 | NA |
| **18_0110** | *Mormopterus francoismoutoui* | Trois bassins | NA | 0 | NA |
| **18_0111** | *Mormopterus francoismoutoui* | Trois bassins | NA | 0 | NA |
| **18_0112** | *Mormopterus francoismoutoui* | Trois bassins | NA | 0 | NA |
| **18_0113** | *Mormopterus francoismoutoui* | Trois bassins | NA | 0 | NA |
| **18_0114** | *Mormopterus francoismoutoui* | Trois bassins | NA | 0 | NA |
| **18_0115** | *Mormopterus francoismoutoui* | Transformateur EDF Gillot | NA | 0 | NA |
| **18_0116** | *Mormopterus francoismoutoui* | Transformateur EDF Gillot | NA | 0 | NA |
| **18_0117** | *Mormopterus francoismoutoui* | Transformateur EDF Gillot | NA | 0 | NA |
| **18_0118** | *Mormopterus francoismoutoui* | Transformateur EDF Gillot | NA | 0 | NA |
| **18_0119** | *Mormopterus francoismoutoui* | Transformateur EDF Gillot | NA | 0 | NA |
| **18_0120** | *Mormopterus francoismoutoui* | Transformateur EDF Gillot | NA | 1 | OR752503 |
| **18_0121** | *Mormopterus francoismoutoui* | Transformateur EDF Gillot | NA | 0 | NA |
| **18_0122** | *Mormopterus francoismoutoui* | Transformateur EDF Gillot | NA | 0 | NA |
| **18_0123** | *Mormopterus francoismoutoui* | Transformateur EDF Gillot | NA | 0 | NA |
| **18_0124** | *Mormopterus francoismoutoui* | Transformateur EDF Gillot | NA | 0 | NA |
| **18_0125** | *Mormopterus francoismoutoui* | Transformateur EDF Gillot | NA | 0 | NA |
| **18_0126** | *Mormopterus francoismoutoui* | Transformateur EDF Gillot | NA | 0 | NA |
| **18_0127** | *Mormopterus francoismoutoui* | Transformateur EDF Gillot | NA | 0 | NA |
| **18_0128** | *Mormopterus francoismoutoui* | Transformateur EDF Gillot | NA | 0 | NA |
| **18_0129** | *Mormopterus francoismoutoui* | Transformateur EDF Gillot | NA | 0 | NA |
| **18_0130** | *Mormopterus francoismoutoui* | Transformateur EDF Gillot | NA | 0 | NA |
| **18_0131** | *Mormopterus francoismoutoui* | Transformateur EDF Gillot | NA | 0 | NA |
| **18_0132** | *Mormopterus francoismoutoui* | Transformateur EDF Gillot | NA | 0 | NA |
| **18_0133** | *Mormopterus francoismoutoui* | Transformateur EDF Gillot | NA | 0 | NA |
| **18_0134** | *Mormopterus francoismoutoui* | Transformateur EDF Gillot | NA | 0 | NA |
| **18_0135** | *Mormopterus francoismoutoui* | Transformateur EDF Gillot | NA | 0 | NA |

| | | | | | |
|---|---|---|---|---|---|
| **18_0136** | *Mormopterus francoismoutoui* | Transformateur EDF Gillot | NA | 0 | NA |
| **18_0137** | *Mormopterus francoismoutoui* | Transformateur EDF Gillot | NA | 0 | NA |
| **18_0138** | *Mormopterus francoismoutoui* | Transformateur EDF Gillot | NA | 0 | NA |
| **18_0139** | *Mormopterus francoismoutoui* | Transformateur EDF Gillot | NA | 0 | NA |
| **18_0140** | *Mormopterus francoismoutoui* | Transformateur EDF Gillot | NA | 0 | NA |
| **18_0141** | *Mormopterus francoismoutoui* | Transformateur EDF Gillot | NA | 0 | NA |
| **18_0142** | *Mormopterus francoismoutoui* | Transformateur EDF Gillot | NA | 0 | NA |
| **18_0143** | *Mormopterus francoismoutoui* | Transformateur EDF Gillot | NA | 0 | NA |
| **18_0144** | *Mormopterus francoismoutoui* | Transformateur EDF Gillot | NA | 0 | NA |
| **18_0155** | *Mormopterus francoismoutoui* | Trois bassins | NA | 0 | NA |
| **18_0156** | *Mormopterus francoismoutoui* | Trois bassins | NA | 0 | NA |
| **18_0157** | *Mormopterus francoismoutoui* | Trois bassins | NA | 0 | NA |
| **18_0158** | *Mormopterus francoismoutoui* | Trois bassins | NA | 0 | NA |
| **18_0159** | *Mormopterus francoismoutoui* | Trois bassins | NA | 0 | NA |
| **18_0160** | *Mormopterus francoismoutoui* | Trois bassins | NA | 0 | NA |
| **18_0161** | *Mormopterus francoismoutoui* | Trois bassins | NA | 0 | NA |
| **18_0162** | *Mormopterus francoismoutoui* | Trois bassins | NA | 0 | NA |
| **18_0163** | *Mormopterus francoismoutoui* | Trois bassins | NA | 0 | NA |
| **18_0164** | *Mormopterus francoismoutoui* | Trois bassins | NA | 0 | NA |
| **18_0165** | *Mormopterus francoismoutoui* | Trois bassins | NA | 0 | NA |
| **18_0166** | *Mormopterus francoismoutoui* | Trois bassins | NA | 0 | NA |
| **18_0167** | *Mormopterus francoismoutoui* | Trois bassins | NA | 0 | NA |
| **18_0168** | *Mormopterus francoismoutoui* | Trois bassins | NA | 0 | NA |
| **18_0169** | *Mormopterus francoismoutoui* | Trois bassins | NA | 0 | NA |
| **18_0170** | *Mormopterus francoismoutoui* | Trois bassins | NA | 0 | NA |
| **18_0171** | *Mormopterus francoismoutoui* | Trois bassins | NA | 0 | NA |
| **18_0172** | *Mormopterus francoismoutoui* | Trois bassins | NA | 0 | NA |
| **18_0173** | *Mormopterus francoismoutoui* | Trois bassins | NA | 0 | NA |
| **18_0174** | *Mormopterus francoismoutoui* | Trois bassins | NA | 0 | NA |
| **18_0175** | *Mormopterus francoismoutoui* | Trois bassins | NA | 0 | NA |
| **18_0176** | *Mormopterus francoismoutoui* | Trois bassins | NA | 0 | NA |
| **18_0177** | *Mormopterus francoismoutoui* | Trois bassins | NA | 0 | NA |
| **18_0178** | *Mormopterus francoismoutoui* | Trois bassins | NA | 0 | NA |
| **18_0179** | *Mormopterus francoismoutoui* | Trois bassins | NA | 0 | NA |
| **18_0180** | *Mormopterus francoismoutoui* | Trois bassins | NA | 0 | NA |
| **18_0181** | *Mormopterus francoismoutoui* | Trois bassins | NA | 0 | NA |
| **18_0182** | *Mormopterus francoismoutoui* | Trois bassins | NA | 0 | NA |
| **18_0183** | *Mormopterus francoismoutoui* | Trois bassins | NA | 0 | NA |
| **18_0184** | *Mormopterus francoismoutoui* | Trois bassins | NA | 0 | NA |
| **18_0185** | *Mormopterus francoismoutoui* | Trois bassins | NA | 0 | NA |
| **18_0186** | *Mormopterus francoismoutoui* | Trois bassins | NA | 0 | NA |
| **18_0187** | *Mormopterus francoismoutoui* | Trois bassins | NA | 0 | NA |
| **18_0188** | *Mormopterus francoismoutoui* | Trois bassins | NA | 0 | NA |
| **18_0189** | *Mormopterus francoismoutoui* | Trois bassins | NA | 0 | NA |
| **18_0190** | *Mormopterus francoismoutoui* | Trois bassins | NA | 0 | NA |
| **18_0191** | *Mormopterus francoismoutoui* | Trois bassins | NA | 0 | NA |
| **18_0192** | *Mormopterus francoismoutoui* | Trois bassins | NA | 0 | NA |
| **18_0193** | *Mormopterus francoismoutoui* | Trois bassins | NA | 0 | NA |
| **18_0194** | *Mormopterus francoismoutoui* | Trois bassins | NA | 0 | NA |
| **18_0195** | *Mormopterus francoismoutoui* | Trois bassins | NA | 0 | NA |
| **18_0196** | *Mormopterus francoismoutoui* | Trois bassins | NA | 0 | NA |
| **18_0197** | *Mormopterus francoismoutoui* | Trois bassins | NA | 0 | NA |
| **18_0198** | *Mormopterus francoismoutoui* | Trois bassins | NA | 0 | NA |
| **18_0199** | *Mormopterus francoismoutoui* | Trois bassins | NA | 0 | NA |
| **18_0200** | *Mormopterus francoismoutoui* | Trois bassins | NA | 0 | NA |
| **18_0201** | *Mormopterus francoismoutoui* | Trois bassins | NA | 0 | NA |
| **18_0202** | *Mormopterus francoismoutoui* | Trois bassins | NA | 0 | NA |
| **18_0203** | *Mormopterus francoismoutoui* | Trois bassins | NA | 0 | NA |
| **18_0204** | *Mormopterus francoismoutoui* | Trois bassins | NA | 0 | NA |
| **18_0205** | *Mormopterus francoismoutoui* | Transformateur EDF Gillot | NA | 0 | NA |
| **18_0206** | *Mormopterus francoismoutoui* | Transformateur EDF Gillot | NA | 0 | NA |
| **18_0207** | *Mormopterus francoismoutoui* | Transformateur EDF Gillot | NA | 0 | NA |
| **18_0208** | *Mormopterus francoismoutoui* | Transformateur EDF Gillot | NA | 0 | NA |
| **18_0209** | *Mormopterus francoismoutoui* | Transformateur EDF Gillot | NA | 0 | NA |
| **18_0210** | *Mormopterus francoismoutoui* | Transformateur EDF Gillot | NA | 0 | NA |
| **18_0211** | *Mormopterus francoismoutoui* | Transformateur EDF Gillot | NA | 0 | NA |
| **18_0212** | *Mormopterus francoismoutoui* | Transformateur EDF Gillot | NA | 0 | NA |
| **18_0213** | *Mormopterus francoismoutoui* | Transformateur EDF Gillot | NA | 0 | NA |
| **18_0214** | *Mormopterus francoismoutoui* | Transformateur EDF Gillot | NA | 0 | NA |
| **18_0215** | *Mormopterus francoismoutoui* | Transformateur EDF Gillot | NA | 0 | NA |
| **18_0216** | *Mormopterus francoismoutoui* | Transformateur EDF Gillot | NA | 0 | NA |
| **18_0217** | *Mormopterus francoismoutoui* | Transformateur EDF Gillot | NA | 0 | NA |

| | | | | | |
|---|---|---|---|---|---|
| **18_0218** | *Mormopterus francoismoutoui* | Transformateur EDF Gillot | NA | 0 | NA |
| **18_0219** | *Mormopterus francoismoutoui* | Transformateur EDF Gillot | NA | 0 | NA |
| **18_0220** | *Mormopterus francoismoutoui* | Transformateur EDF Gillot | NA | 0 | NA |
| **18_0221** | *Mormopterus francoismoutoui* | Transformateur EDF Gillot | NA | 0 | NA |
| **18_0222** | *Mormopterus francoismoutoui* | Transformateur EDF Gillot | NA | 0 | NA |
| **18_0223** | *Mormopterus francoismoutoui* | Transformateur EDF Gillot | NA | 0 | NA |
| **18_0224** | *Mormopterus francoismoutoui* | Transformateur EDF Gillot | NA | 0 | NA |
| **18_0225** | *Mormopterus francoismoutoui* | Transformateur EDF Gillot | NA | 0 | NA |
| **18_0226** | *Mormopterus francoismoutoui* | Transformateur EDF Gillot | NA | 0 | NA |
| **18_0227** | *Mormopterus francoismoutoui* | Transformateur EDF Gillot | NA | 0 | NA |
| **18_0228** | *Mormopterus francoismoutoui* | Transformateur EDF Gillot | NA | 0 | NA |
| **18_0229** | *Mormopterus francoismoutoui* | Transformateur EDF Gillot | NA | 0 | NA |
| **18_0230** | *Mormopterus francoismoutoui* | Transformateur EDF Gillot | NA | 0 | NA |
| **18_0231** | *Mormopterus francoismoutoui* | Transformateur EDF Gillot | NA | 0 | NA |
| **18_0232** | *Mormopterus francoismoutoui* | Transformateur EDF Gillot | NA | 0 | NA |
| **18_0233** | *Mormopterus francoismoutoui* | Transformateur EDF Gillot | NA | 0 | NA |
| **18_0234** | *Mormopterus francoismoutoui* | Transformateur EDF Gillot | NA | 0 | NA |
| **18_0245** | *Mormopterus francoismoutoui* | Trois bassins | NA | 0 | NA |
| **18_0246** | *Mormopterus francoismoutoui* | Trois bassins | NA | 0 | NA |
| **18_0247** | *Mormopterus francoismoutoui* | Trois bassins | NA | 0 | NA |
| **18_0248** | *Mormopterus francoismoutoui* | Trois bassins | NA | 0 | NA |
| **18_0249** | *Mormopterus francoismoutoui* | Trois bassins | NA | 0 | NA |
| **18_0250** | *Mormopterus francoismoutoui* | Trois bassins | NA | 0 | NA |
| **18_0251** | *Mormopterus francoismoutoui* | Trois bassins | NA | 0 | NA |
| **18_0252** | *Mormopterus francoismoutoui* | Trois bassins | NA | 0 | NA |
| **18_0253** | *Mormopterus francoismoutoui* | Trois bassins | NA | 0 | NA |
| **18_0254** | *Mormopterus francoismoutoui* | Trois bassins | NA | 0 | NA |
| **18_0255** | *Mormopterus francoismoutoui* | Trois bassins | NA | 0 | NA |
| **18_0256** | *Mormopterus francoismoutoui* | Trois bassins | NA | 0 | NA |
| **18_0257** | *Mormopterus francoismoutoui* | Trois bassins | NA | 0 | NA |
| **18_0258** | *Mormopterus francoismoutoui* | Trois bassins | NA | 0 | NA |
| **18_0259** | *Mormopterus francoismoutoui* | Trois bassins | NA | 0 | NA |
| **18_0260** | *Mormopterus francoismoutoui* | Trois bassins | NA | 0 | NA |
| **18_0261** | *Mormopterus francoismoutoui* | Trois bassins | NA | 0 | NA |
| **18_0262** | *Mormopterus francoismoutoui* | Trois bassins | NA | 0 | NA |
| **18_0263** | *Mormopterus francoismoutoui* | Trois bassins | NA | 0 | NA |
| **18_0264** | *Mormopterus francoismoutoui* | Trois bassins | NA | 0 | NA |
| **18_0265** | *Mormopterus francoismoutoui* | Trois bassins | NA | 0 | NA |
| **18_0266** | *Mormopterus francoismoutoui* | Trois bassins | NA | 0 | NA |
| **18_0267** | *Mormopterus francoismoutoui* | Trois bassins | NA | 1 | OR752504 |
| **18_0268** | *Mormopterus francoismoutoui* | Trois bassins | NA | 0 | NA |
| **18_0269** | *Mormopterus francoismoutoui* | Trois bassins | NA | 0 | NA |
| **18_0270** | *Mormopterus francoismoutoui* | Trois bassins | NA | 0 | NA |
| **18_0271** | *Mormopterus francoismoutoui* | Trois bassins | NA | 0 | NA |
| **18_0272** | *Mormopterus francoismoutoui* | Trois bassins | NA | 0 | NA |
| **18_0273** | *Mormopterus francoismoutoui* | Trois bassins | NA | 0 | NA |
| **18_0274** | *Mormopterus francoismoutoui* | Trois bassins | NA | 0 | NA |
| **18_0275** | *Mormopterus francoismoutoui* | Trois bassins | NA | 0 | NA |
| **18_0276** | *Mormopterus francoismoutoui* | Trois bassins | NA | 0 | NA |
| **18_0277** | *Mormopterus francoismoutoui* | Trois bassins | NA | 0 | NA |
| **18_0278** | *Mormopterus francoismoutoui* | Trois bassins | NA | 1 | OR752505 |
| **18_0279** | *Mormopterus francoismoutoui* | Trois bassins | NA | 0 | NA |
| **18_0280** | *Mormopterus francoismoutoui* | Trois bassins | NA | 0 | NA |
| **18_0281** | *Mormopterus francoismoutoui* | Trois bassins | NA | 0 | NA |
| **18_0282** | *Mormopterus francoismoutoui* | Trois bassins | NA | 0 | NA |
| **18_0283** | *Mormopterus francoismoutoui* | Trois bassins | NA | 0 | NA |
| **18_0284** | *Mormopterus francoismoutoui* | Trois bassins | NA | 0 | NA |
| **18_0285** | *Mormopterus francoismoutoui* | Trois bassins | NA | 0 | NA |
| **18_0286** | *Mormopterus francoismoutoui* | Trois bassins | NA | 0 | NA |
| **18_0287** | *Mormopterus francoismoutoui* | Trois bassins | NA | 0 | NA |
| **18_0288** | *Mormopterus francoismoutoui* | Trois bassins | NA | 0 | NA |
| **18_0289** | *Mormopterus francoismoutoui* | Trois bassins | NA | 0 | NA |
| **18_0290** | *Mormopterus francoismoutoui* | Trois bassins | NA | 0 | NA |
| **18_0291** | *Mormopterus francoismoutoui* | Trois bassins | NA | 0 | NA |
| **18_0292** | *Mormopterus francoismoutoui* | Trois bassins | NA | 0 | NA |
| **18_0293** | *Mormopterus francoismoutoui* | Trois bassins | NA | 0 | NA |
| **18_0294** | *Mormopterus francoismoutoui* | Trois bassins | NA | 0 | NA |
| **18_0295** | *Mormopterus francoismoutoui* | Transformateur EDF Gillot | NA | 0 | NA |
| **18_0296** | *Mormopterus francoismoutoui* | Transformateur EDF Gillot | NA | 0 | NA |
| **18_0297** | *Mormopterus francoismoutoui* | Transformateur EDF Gillot | NA | 0 | NA |
| **18_0298** | *Mormopterus francoismoutoui* | Transformateur EDF Gillot | NA | 0 | NA |
| **18_0299** | *Mormopterus francoismoutoui* | Transformateur EDF Gillot | NA | 0 | NA |

| | | | | | |
|---|---|---|---|---|---|
| **18_0300** | *Mormopterus francoismoutoui* | Transformateur EDF Gillot | NA | 0 | NA |
| **18_0301** | *Mormopterus francoismoutoui* | Transformateur EDF Gillot | NA | 0 | NA |
| **18_0302** | *Mormopterus francoismoutoui* | Transformateur EDF Gillot | NA | 0 | NA |
| **18_0303** | *Mormopterus francoismoutoui* | Transformateur EDF Gillot | NA | 0 | NA |
| **18_0304** | *Mormopterus francoismoutoui* | Transformateur EDF Gillot | NA | 0 | NA |
| **18_0305** | *Mormopterus francoismoutoui* | Transformateur EDF Gillot | NA | 0 | NA |
| **18_0306** | *Mormopterus francoismoutoui* | Transformateur EDF Gillot | NA | 0 | NA |
| **18_0307** | *Mormopterus francoismoutoui* | Transformateur EDF Gillot | NA | 0 | NA |
| **18_0308** | *Mormopterus francoismoutoui* | Transformateur EDF Gillot | NA | 0 | NA |
| **18_0309** | *Mormopterus francoismoutoui* | Transformateur EDF Gillot | NA | 0 | NA |
| **18_0310** | *Mormopterus francoismoutoui* | Transformateur EDF Gillot | NA | 0 | NA |
| **18_0311** | *Mormopterus francoismoutoui* | Transformateur EDF Gillot | NA | 0 | NA |
| **18_0312** | *Mormopterus francoismoutoui* | Transformateur EDF Gillot | NA | 0 | NA |
| **18_0313** | *Mormopterus francoismoutoui* | Transformateur EDF Gillot | NA | 0 | NA |
| **18_0314** | *Mormopterus francoismoutoui* | Transformateur EDF Gillot | NA | 0 | NA |
| **18_0325** | *Mormopterus francoismoutoui* | Trois bassins | NA | 0 | NA |
| **18_0326** | *Mormopterus francoismoutoui* | Trois bassins | NA | 0 | NA |
| **18_0327** | *Mormopterus francoismoutoui* | Trois bassins | NA | 0 | NA |
| **18_0328** | *Mormopterus francoismoutoui* | Trois bassins | NA | 0 | NA |
| **18_0329** | *Mormopterus francoismoutoui* | Trois bassins | NA | 0 | NA |
| **18_0330** | *Mormopterus francoismoutoui* | Trois bassins | NA | 0 | NA |
| **18_0331** | *Mormopterus francoismoutoui* | Trois bassins | NA | 0 | NA |
| **18_0332** | *Mormopterus francoismoutoui* | Trois bassins | NA | 0 | NA |
| **18_0333** | *Mormopterus francoismoutoui* | Trois bassins | NA | 0 | NA |
| **18_0334** | *Mormopterus francoismoutoui* | Trois bassins | NA | 0 | NA |
| **18_0335** | *Mormopterus francoismoutoui* | Trois bassins | NA | 0 | NA |
| **18_0336** | *Mormopterus francoismoutoui* | Trois bassins | NA | 0 | NA |
| **18_0337** | *Mormopterus francoismoutoui* | Trois bassins | NA | 0 | NA |
| **18_0338** | *Mormopterus francoismoutoui* | Trois bassins | NA | 0 | NA |
| **18_0339** | *Mormopterus francoismoutoui* | Trois bassins | NA | 0 | NA |
| **18_0340** | *Mormopterus francoismoutoui* | Trois bassins | NA | 0 | NA |
| **18_0341** | *Mormopterus francoismoutoui* | Trois bassins | NA | 0 | NA |
| **18_0342** | *Mormopterus francoismoutoui* | Trois bassins | NA | 1 | OR752506 |
| **18_0343** | *Mormopterus francoismoutoui* | Trois bassins | NA | 0 | NA |
| **18_0344** | *Mormopterus francoismoutoui* | Trois bassins | NA | 0 | NA |
| **18_0345** | *Mormopterus francoismoutoui* | Trois bassins | NA | 0 | NA |
| **18_0346** | *Mormopterus francoismoutoui* | Trois bassins | NA | 0 | NA |
| **18_0347** | *Mormopterus francoismoutoui* | Trois bassins | NA | 0 | NA |
| **18_0348** | *Mormopterus francoismoutoui* | Trois bassins | NA | 0 | NA |
| **18_0349** | *Mormopterus francoismoutoui* | Trois bassins | NA | 0 | NA |
| **18_0350** | *Mormopterus francoismoutoui* | Trois bassins | NA | 0 | NA |
| **18_0351** | *Mormopterus francoismoutoui* | Trois bassins | NA | 0 | NA |
| **18_0352** | *Mormopterus francoismoutoui* | Trois bassins | NA | 0 | NA |
| **18_0353** | *Mormopterus francoismoutoui* | Trois bassins | NA | 0 | NA |
| **18_0354** | *Mormopterus francoismoutoui* | Trois bassins | NA | 0 | NA |
| **18_0355** | *Mormopterus francoismoutoui* | Trois bassins | NA | 0 | NA |
| **18_0356** | *Mormopterus francoismoutoui* | Trois bassins | NA | 0 | NA |
| **18_0357** | *Mormopterus francoismoutoui* | Trois bassins | NA | 0 | NA |
| **18_0358** | *Mormopterus francoismoutoui* | Trois bassins | NA | 0 | NA |
| **18_0359** | *Mormopterus francoismoutoui* | Trois bassins | NA | 0 | NA |
| **18_0360** | *Mormopterus francoismoutoui* | Trois bassins | NA | 0 | NA |
| **18_0361** | *Mormopterus francoismoutoui* | Trois bassins | NA | 1 | OR752507 |
| **18_0362** | *Mormopterus francoismoutoui* | Trois bassins | NA | 0 | NA |
| **18_0363** | *Mormopterus francoismoutoui* | Trois bassins | NA | 0 | NA |
| **18_0364** | *Mormopterus francoismoutoui* | Trois bassins | NA | 0 | NA |
| **18_0365** | *Mormopterus francoismoutoui* | Trois bassins | NA | 0 | NA |
| **18_0366** | *Mormopterus francoismoutoui* | Trois bassins | NA | 0 | NA |
| **18_0367** | *Mormopterus francoismoutoui* | Trois bassins | NA | 0 | NA |
| **18_0368** | *Mormopterus francoismoutoui* | Trois bassins | NA | 0 | NA |
| **18_0369** | *Mormopterus francoismoutoui* | Trois bassins | NA | 0 | NA |
| **18_0370** | *Mormopterus francoismoutoui* | Trois bassins | NA | 0 | NA |
| **18_0371** | *Mormopterus francoismoutoui* | Trois bassins | NA | 0 | NA |
| **18_0372** | *Mormopterus francoismoutoui* | Trois bassins | NA | 0 | NA |
| **18_0373** | *Mormopterus francoismoutoui* | Trois bassins | NA | 0 | NA |
| **18_0374** | *Mormopterus francoismoutoui* | Trois bassins | NA | 1 | OR752508 |
| **18_0375** | *Mormopterus francoismoutoui* | Transformateur EDF Gillot | NA | 0 | NA |
| **18_0376** | *Mormopterus francoismoutoui* | Transformateur EDF Gillot | NA | 0 | NA |
| **18_0377** | *Mormopterus francoismoutoui* | Transformateur EDF Gillot | NA | 0 | NA |
| **18_0378** | *Mormopterus francoismoutoui* | Transformateur EDF Gillot | NA | 0 | NA |
| **18_0379** | *Mormopterus francoismoutoui* | Transformateur EDF Gillot | NA | 0 | NA |
| **18_0380** | *Mormopterus francoismoutoui* | Transformateur EDF Gillot | NA | 0 | NA |
| **18_0381** | *Mormopterus francoismoutoui* | Transformateur EDF Gillot | NA | 0 | NA |

| | | | | | |
|---|---|---|---|---|---|
| **18_0382** | *Mormopterus francoismoutoui* | Transformateur EDF Gillot | NA | 0 | NA |
| **18_0383** | *Mormopterus francoismoutoui* | Transformateur EDF Gillot | NA | 0 | NA |
| **18_0384** | *Mormopterus francoismoutoui* | Transformateur EDF Gillot | NA | 0 | NA |
| **18_0395** | *Mormopterus francoismoutoui* | Trois bassins | NA | 0 | NA |
| **18_0396** | *Mormopterus francoismoutoui* | Trois bassins | NA | 0 | NA |
| **18_0397** | *Mormopterus francoismoutoui* | Trois bassins | NA | 0 | NA |
| **18_0398** | *Mormopterus francoismoutoui* | Trois bassins | NA | 0 | NA |
| **18_0399** | *Mormopterus francoismoutoui* | Trois bassins | NA | 0 | NA |
| **18_0400** | *Mormopterus francoismoutoui* | Trois bassins | NA | 0 | NA |
| **18_0401** | *Mormopterus francoismoutoui* | Trois bassins | NA | 0 | NA |
| **18_0402** | *Mormopterus francoismoutoui* | Trois bassins | NA | 0 | NA |
| **18_0403** | *Mormopterus francoismoutoui* | Trois bassins | NA | 0 | NA |
| **18_0404** | *Mormopterus francoismoutoui* | Trois bassins | NA | 0 | NA |
| **18_0405** | *Mormopterus francoismoutoui* | Trois bassins | NA | 0 | NA |
| **18_0406** | *Mormopterus francoismoutoui* | Trois bassins | NA | 0 | NA |
| **18_0407** | *Mormopterus francoismoutoui* | Trois bassins | NA | 0 | NA |
| **18_0408** | *Mormopterus francoismoutoui* | Trois bassins | NA | 0 | NA |
| **18_0409** | *Mormopterus francoismoutoui* | Trois bassins | NA | 0 | NA |
| **18_0410** | *Mormopterus francoismoutoui* | Trois bassins | NA | 0 | NA |
| **18_0411** | *Mormopterus francoismoutoui* | Trois bassins | NA | 0 | NA |
| **18_0412** | *Mormopterus francoismoutoui* | Trois bassins | NA | 0 | NA |
| **18_0413** | *Mormopterus francoismoutoui* | Trois bassins | NA | 0 | NA |
| **18_0414** | *Mormopterus francoismoutoui* | Trois bassins | NA | 0 | NA |
| **18_0415** | *Mormopterus francoismoutoui* | Trois bassins | NA | 0 | NA |
| **18_0416** | *Mormopterus francoismoutoui* | Trois bassins | NA | 0 | NA |
| **18_0417** | *Mormopterus francoismoutoui* | Trois bassins | NA | 1 | OR752509 |
| **18_0418** | *Mormopterus francoismoutoui* | Trois bassins | NA | 0 | NA |
| **18_0419** | *Mormopterus francoismoutoui* | Trois bassins | NA | 0 | NA |
| **18_0420** | *Mormopterus francoismoutoui* | Trois bassins | NA | 0 | NA |
| **18_0421** | *Mormopterus francoismoutoui* | Trois bassins | NA | 0 | NA |
| **18_0422** | *Mormopterus francoismoutoui* | Trois bassins | NA | 0 | NA |
| **18_0423** | *Mormopterus francoismoutoui* | Trois bassins | NA | 0 | NA |
| **18_0424** | *Mormopterus francoismoutoui* | Trois bassins | NA | 0 | NA |
| **18_0425** | *Mormopterus francoismoutoui* | Trois bassins | NA | 0 | NA |
| **18_0426** | *Mormopterus francoismoutoui* | Trois bassins | NA | 0 | NA |
| **18_0427** | *Mormopterus francoismoutoui* | Trois bassins | NA | 0 | NA |
| **18_0428** | *Mormopterus francoismoutoui* | Trois bassins | NA | 0 | NA |
| **18_0429** | *Mormopterus francoismoutoui* | Trois bassins | NA | 0 | NA |
| **18_0430** | *Mormopterus francoismoutoui* | Trois bassins | NA | 0 | NA |
| **18_0431** | *Mormopterus francoismoutoui* | Trois bassins | NA | 0 | NA |
| **18_0432** | *Mormopterus francoismoutoui* | Trois bassins | NA | 0 | NA |
| **18_0433** | *Mormopterus francoismoutoui* | Trois bassins | NA | 0 | NA |
| **18_0434** | *Mormopterus francoismoutoui* | Trois bassins | NA | 0 | NA |
| **18_0435** | *Mormopterus francoismoutoui* | Trois bassins | NA | 0 | NA |
| **18_0436** | *Mormopterus francoismoutoui* | Trois bassins | NA | 0 | NA |
| **18_0437** | *Mormopterus francoismoutoui* | Trois bassins | NA | 0 | NA |
| **18_0438** | *Mormopterus francoismoutoui* | Trois bassins | NA | 0 | NA |
| **18_0439** | *Mormopterus francoismoutoui* | Trois bassins | NA | 0 | NA |
| **18_0440** | *Mormopterus francoismoutoui* | Trois bassins | NA | 0 | NA |
| **18_0441** | *Mormopterus francoismoutoui* | Trois bassins | NA | 0 | NA |
| **18_0442** | *Mormopterus francoismoutoui* | Trois bassins | NA | 0 | NA |
| **18_0443** | *Mormopterus francoismoutoui* | Trois bassins | NA | 0 | NA |
| **18_0444** | *Mormopterus francoismoutoui* | Trois bassins | NA | 0 | NA |
| **18_0445** | *Mormopterus francoismoutoui* | Transformateur EDF Gillot | NA | 0 | NA |
| **18_0446** | *Mormopterus francoismoutoui* | Transformateur EDF Gillot | NA | 0 | NA |
| **18_0447** | *Mormopterus francoismoutoui* | Transformateur EDF Gillot | NA | 0 | NA |
| **18_0448** | *Mormopterus francoismoutoui* | Transformateur EDF Gillot | NA | 0 | NA |
| **18_0449** | *Mormopterus francoismoutoui* | Transformateur EDF Gillot | NA | 0 | NA |
| **18_0450** | *Mormopterus francoismoutoui* | Transformateur EDF Gillot | NA | 0 | NA |
| **18_0451** | *Mormopterus francoismoutoui* | Transformateur EDF Gillot | NA | 0 | NA |
| **18_0452** | *Mormopterus francoismoutoui* | Transformateur EDF Gillot | NA | 0 | NA |
| **18_0453** | *Mormopterus francoismoutoui* | Transformateur EDF Gillot | NA | 0 | NA |
| **18_0454** | *Mormopterus francoismoutoui* | Transformateur EDF Gillot | NA | 0 | NA |
| **18_0455** | *Mormopterus francoismoutoui* | Transformateur EDF Gillot | NA | 0 | NA |
| **18_0456** | *Mormopterus francoismoutoui* | Transformateur EDF Gillot | NA | 0 | NA |
| **18_0457** | *Mormopterus francoismoutoui* | Transformateur EDF Gillot | NA | 0 | NA |
| **18_0458** | *Mormopterus francoismoutoui* | Transformateur EDF Gillot | NA | 0 | NA |
| **18_0459** | *Mormopterus francoismoutoui* | Transformateur EDF Gillot | NA | 0 | NA |
| **18_0460** | *Mormopterus francoismoutoui* | Transformateur EDF Gillot | NA | 0 | NA |
| **18_0461** | *Mormopterus francoismoutoui* | Transformateur EDF Gillot | NA | 0 | NA |
| **18_0462** | *Mormopterus francoismoutoui* | Transformateur EDF Gillot | NA | 0 | NA |
| **18_0463** | *Mormopterus francoismoutoui* | Transformateur EDF Gillot | NA | 0 | NA |

| | | | | | |
|---|---|---|---|---|---|
| **18_0464** | *Mormopterus francoismoutoui* | Transformateur EDF Gillot | NA | 0 | NA |
| **18_0465** | *Mormopterus francoismoutoui* | Transformateur EDF Gillot | NA | 0 | NA |
| **18_0466** | *Mormopterus francoismoutoui* | Transformateur EDF Gillot | NA | 0 | NA |
| **18_0467** | *Mormopterus francoismoutoui* | Transformateur EDF Gillot | NA | 0 | NA |
| **18_0468** | *Mormopterus francoismoutoui* | Transformateur EDF Gillot | NA | 0 | NA |
| **18_0469** | *Mormopterus francoismoutoui* | Transformateur EDF Gillot | NA | 0 | NA |
| **18_0470** | *Mormopterus francoismoutoui* | Transformateur EDF Gillot | NA | 0 | NA |
| **18_0471** | *Mormopterus francoismoutoui* | Transformateur EDF Gillot | NA | 0 | NA |
| **18_0472** | *Mormopterus francoismoutoui* | Transformateur EDF Gillot | NA | 0 | NA |
| **18_0473** | *Mormopterus francoismoutoui* | Transformateur EDF Gillot | NA | 0 | NA |
| **18_0474** | *Mormopterus francoismoutoui* | Transformateur EDF Gillot | NA | 0 | NA |
| **18_0485** | *Mormopterus francoismoutoui* | Trois bassins | NA | 0 | NA |
| **18_0486** | *Mormopterus francoismoutoui* | Trois bassins | NA | 0 | NA |
| **18_0487** | *Mormopterus francoismoutoui* | Trois bassins | NA | 0 | NA |
| **18_0488** | *Mormopterus francoismoutoui* | Trois bassins | NA | 0 | NA |
| **18_0489** | *Mormopterus francoismoutoui* | Trois bassins | NA | 0 | NA |
| **18_0490** | *Mormopterus francoismoutoui* | Trois bassins | NA | 0 | NA |
| **18_0491** | *Mormopterus francoismoutoui* | Trois bassins | NA | 0 | NA |
| **18_0492** | *Mormopterus francoismoutoui* | Trois bassins | NA | 0 | NA |
| **18_0493** | *Mormopterus francoismoutoui* | Trois bassins | NA | 0 | NA |
| **18_0494** | *Mormopterus francoismoutoui* | Trois bassins | NA | 0 | NA |
| **18_0495** | *Mormopterus francoismoutoui* | Trois bassins | NA | 0 | NA |
| **18_0496** | *Mormopterus francoismoutoui* | Trois bassins | NA | 0 | NA |
| **18_0497** | *Mormopterus francoismoutoui* | Trois bassins | NA | 0 | NA |
| **18_0498** | *Mormopterus francoismoutoui* | Trois bassins | NA | 0 | NA |
| **18_0499** | *Mormopterus francoismoutoui* | Trois bassins | NA | 0 | NA |
| **18_0500** | *Mormopterus francoismoutoui* | Trois bassins | NA | 0 | NA |
| **18_0501** | *Mormopterus francoismoutoui* | Trois bassins | NA | 0 | NA |
| **18_0502** | *Mormopterus francoismoutoui* | Trois bassins | NA | 0 | NA |
| **18_0503** | *Mormopterus francoismoutoui* | Trois bassins | NA | 0 | NA |
| **18_0504** | *Mormopterus francoismoutoui* | Trois bassins | NA | 0 | NA |
| **18_0505** | *Mormopterus francoismoutoui* | Trois bassins | NA | 0 | NA |
| **18_0506** | *Mormopterus francoismoutoui* | Trois bassins | NA | 0 | NA |
| **18_0507** | *Mormopterus francoismoutoui* | Trois bassins | NA | 0 | NA |
| **18_0508** | *Mormopterus francoismoutoui* | Trois bassins | NA | 0 | NA |
| **18_0509** | *Mormopterus francoismoutoui* | Trois bassins | NA | 0 | NA |
| **18_0510** | *Mormopterus francoismoutoui* | Trois bassins | NA | 0 | NA |
| **18_0511** | *Mormopterus francoismoutoui* | Trois bassins | NA | 0 | NA |
| **18_0512** | *Mormopterus francoismoutoui* | Trois bassins | NA | 0 | NA |
| **18_0513** | *Mormopterus francoismoutoui* | Trois bassins | NA | 0 | NA |
| **18_0514** | *Mormopterus francoismoutoui* | Trois bassins | NA | 0 | NA |
| **18_0515** | *Mormopterus francoismoutoui* | Transformateur EDF Gillot | NA | 0 | NA |
| **18_0516** | *Mormopterus francoismoutoui* | Transformateur EDF Gillot | NA | 0 | NA |
| **18_0517** | *Mormopterus francoismoutoui* | Transformateur EDF Gillot | NA | 0 | NA |
| **18_0518** | *Mormopterus francoismoutoui* | Transformateur EDF Gillot | NA | 0 | NA |
| **18_0519** | *Mormopterus francoismoutoui* | Transformateur EDF Gillot | NA | 0 | NA |
| **18_0520** | *Mormopterus francoismoutoui* | Transformateur EDF Gillot | NA | 0 | NA |
| **18_0521** | *Mormopterus francoismoutoui* | Transformateur EDF Gillot | NA | 0 | NA |
| **18_0522** | *Mormopterus francoismoutoui* | Transformateur EDF Gillot | NA | 0 | NA |
| **18_0523** | *Mormopterus francoismoutoui* | Transformateur EDF Gillot | NA | 0 | NA |
| **18_0524** | *Mormopterus francoismoutoui* | Transformateur EDF Gillot | NA | 0 | NA |
| **18_0525** | *Mormopterus francoismoutoui* | Transformateur EDF Gillot | NA | 0 | NA |
| **18_0526** | *Mormopterus francoismoutoui* | Transformateur EDF Gillot | NA | 0 | NA |
| **18_0527** | *Mormopterus francoismoutoui* | Transformateur EDF Gillot | NA | 0 | NA |
| **18_0528** | *Mormopterus francoismoutoui* | Transformateur EDF Gillot | NA | 0 | NA |
| **18_0529** | *Mormopterus francoismoutoui* | Transformateur EDF Gillot | NA | 0 | NA |
| **18_0530** | *Mormopterus francoismoutoui* | Transformateur EDF Gillot | NA | 0 | NA |
| **18_0531** | *Mormopterus francoismoutoui* | Transformateur EDF Gillot | NA | 0 | NA |
| **18_0532** | *Mormopterus francoismoutoui* | Transformateur EDF Gillot | NA | 0 | NA |
| **18_0533** | *Mormopterus francoismoutoui* | Transformateur EDF Gillot | NA | 0 | NA |
| **18_0534** | *Mormopterus francoismoutoui* | Transformateur EDF Gillot | NA | 0 | NA |
| **18_0535** | *Mormopterus francoismoutoui* | Transformateur EDF Gillot | NA | 0 | NA |
| **18_0536** | *Mormopterus francoismoutoui* | Transformateur EDF Gillot | NA | 0 | NA |
| **18_0537** | *Mormopterus francoismoutoui* | Transformateur EDF Gillot | NA | 0 | NA |
| **18_0538** | *Mormopterus francoismoutoui* | Transformateur EDF Gillot | NA | 0 | NA |
| **18_0539** | *Mormopterus francoismoutoui* | Transformateur EDF Gillot | NA | 0 | NA |
| **18_0540** | *Mormopterus francoismoutoui* | Transformateur EDF Gillot | NA | 0 | NA |
| **18_0541** | *Mormopterus francoismoutoui* | Transformateur EDF Gillot | NA | 0 | NA |
| **18_0542** | *Mormopterus francoismoutoui* | Transformateur EDF Gillot | NA | 0 | NA |
| **18_0543** | *Mormopterus francoismoutoui* | Transformateur EDF Gillot | NA | 0 | NA |
| **18_0544** | *Mormopterus francoismoutoui* | Transformateur EDF Gillot | NA | 0 | NA |
| **18_0555** | *Mormopterus francoismoutoui* | Trois bassins | NA | 0 | NA |

| | | | | | |
|---|---|---|---|---|---|
| **18_0556** | *Mormopterus francoismoutoui* | Trois bassins | NA | 0 | NA |
| **18_0557** | *Mormopterus francoismoutoui* | Trois bassins | NA | 0 | NA |
| **18_0558** | *Mormopterus francoismoutoui* | Trois bassins | NA | 0 | NA |
| **18_0559** | *Mormopterus francoismoutoui* | Trois bassins | NA | 0 | NA |
| **18_0560** | *Mormopterus francoismoutoui* | Trois bassins | NA | 0 | NA |
| **18_0561** | *Mormopterus francoismoutoui* | Trois bassins | NA | 0 | NA |
| **18_0562** | *Mormopterus francoismoutoui* | Trois bassins | NA | 0 | NA |
| **18_0563** | *Mormopterus francoismoutoui* | Trois bassins | NA | 0 | NA |
| **18_0564** | *Mormopterus francoismoutoui* | Trois bassins | NA | 0 | NA |
| **18_0565** | *Mormopterus francoismoutoui* | Trois bassins | NA | 0 | NA |
| **18_0566** | *Mormopterus francoismoutoui* | Trois bassins | NA | 0 | NA |
| **18_0567** | *Mormopterus francoismoutoui* | Trois bassins | NA | 0 | NA |
| **18_0568** | *Mormopterus francoismoutoui* | Trois bassins | NA | 0 | NA |
| **18_0569** | *Mormopterus francoismoutoui* | Trois bassins | NA | 0 | NA |
| **18_0570** | *Mormopterus francoismoutoui* | Trois bassins | NA | 0 | NA |
| **18_0571** | *Mormopterus francoismoutoui* | Trois bassins | NA | 0 | NA |
| **18_0572** | *Mormopterus francoismoutoui* | Trois bassins | NA | 0 | NA |
| **18_0573** | *Mormopterus francoismoutoui* | Trois bassins | NA | 0 | NA |
| **18_0574** | *Mormopterus francoismoutoui* | Trois bassins | NA | 0 | NA |
| **18_0575** | *Mormopterus francoismoutoui* | Trois bassins | NA | 0 | NA |
| **18_0576** | *Mormopterus francoismoutoui* | Trois bassins | NA | 0 | NA |
| **18_0577** | *Mormopterus francoismoutoui* | Trois bassins | NA | 0 | NA |
| **18_0578** | *Mormopterus francoismoutoui* | Trois bassins | NA | 0 | NA |
| **18_0579** | *Mormopterus francoismoutoui* | Trois bassins | NA | 0 | NA |
| **18_0580** | *Mormopterus francoismoutoui* | Trois bassins | NA | 0 | NA |
| **18_0581** | *Mormopterus francoismoutoui* | Trois bassins | NA | 0 | NA |
| **18_0582** | *Mormopterus francoismoutoui* | Trois bassins | NA | 0 | NA |
| **18_0583** | *Mormopterus francoismoutoui* | Trois bassins | NA | 0 | NA |
| **18_0584** | *Mormopterus francoismoutoui* | Trois bassins | NA | 0 | NA |
| **18_0585** | *Mormopterus francoismoutoui* | Transformateur EDF Gillot | NA | 0 | NA |
| **18_0586** | *Mormopterus francoismoutoui* | Transformateur EDF Gillot | NA | 0 | NA |
| **18_0587** | *Mormopterus francoismoutoui* | Transformateur EDF Gillot | NA | 0 | NA |
| **18_0588** | *Mormopterus francoismoutoui* | Transformateur EDF Gillot | NA | 0 | NA |
| **18_0589** | *Mormopterus francoismoutoui* | Transformateur EDF Gillot | NA | 0 | NA |
| **18_0590** | *Mormopterus francoismoutoui* | Transformateur EDF Gillot | NA | 0 | NA |
| **18_0591** | *Mormopterus francoismoutoui* | Transformateur EDF Gillot | NA | 0 | NA |
| **18_0592** | *Mormopterus francoismoutoui* | Transformateur EDF Gillot | NA | 0 | NA |
| **18_0593** | *Mormopterus francoismoutoui* | Transformateur EDF Gillot | NA | 0 | NA |
| **18_0594** | *Mormopterus francoismoutoui* | Transformateur EDF Gillot | NA | 1 | OR752510 |
| **18_0595** | *Mormopterus francoismoutoui* | Transformateur EDF Gillot | NA | 0 | NA |
| **18_0596** | *Mormopterus francoismoutoui* | Transformateur EDF Gillot | NA | 0 | NA |
| **18_0597** | *Mormopterus francoismoutoui* | Transformateur EDF Gillot | NA | 0 | NA |
| **18_0598** | *Mormopterus francoismoutoui* | Transformateur EDF Gillot | NA | 0 | NA |
| **18_0599** | *Mormopterus francoismoutoui* | Transformateur EDF Gillot | NA | 0 | NA |
| **18_0600** | *Mormopterus francoismoutoui* | Transformateur EDF Gillot | NA | 0 | NA |
| **18_0601** | *Mormopterus francoismoutoui* | Transformateur EDF Gillot | NA | 0 | NA |
| **18_0602** | *Mormopterus francoismoutoui* | Transformateur EDF Gillot | NA | 0 | NA |
| **18_0603** | *Mormopterus francoismoutoui* | Transformateur EDF Gillot | NA | 0 | NA |
| **18_0604** | *Mormopterus francoismoutoui* | Transformateur EDF Gillot | NA | 0 | NA |
| **18_0605** | *Mormopterus francoismoutoui* | Transformateur EDF Gillot | NA | 0 | NA |
| **18_0606** | *Mormopterus francoismoutoui* | Transformateur EDF Gillot | NA | 0 | NA |
| **18_0607** | *Mormopterus francoismoutoui* | Transformateur EDF Gillot | NA | 0 | NA |
| **18_0608** | *Mormopterus francoismoutoui* | Transformateur EDF Gillot | NA | 0 | NA |
| **18_0609** | *Mormopterus francoismoutoui* | Transformateur EDF Gillot | NA | 0 | NA |
| **18_0610** | *Mormopterus francoismoutoui* | Transformateur EDF Gillot | NA | 0 | NA |
| **18_0611** | *Mormopterus francoismoutoui* | Transformateur EDF Gillot | NA | 0 | NA |
| **18_0612** | *Mormopterus francoismoutoui* | Transformateur EDF Gillot | NA | 0 | NA |
| **18_0613** | *Mormopterus francoismoutoui* | Transformateur EDF Gillot | NA | 0 | NA |
| **18_0614** | *Mormopterus francoismoutoui* | Transformateur EDF Gillot | NA | 0 | NA |
| **18_0625** | *Mormopterus francoismoutoui* | Trois bassins | NA | 0 | NA |
| **18_0626** | *Mormopterus francoismoutoui* | Trois bassins | NA | 0 | NA |
| **18_0627** | *Mormopterus francoismoutoui* | Trois bassins | NA | 0 | NA |
| **18_0628** | *Mormopterus francoismoutoui* | Trois bassins | NA | 0 | NA |
| **18_0629** | *Mormopterus francoismoutoui* | Trois bassins | NA | 0 | NA |
| **18_0630** | *Mormopterus francoismoutoui* | Trois bassins | NA | 0 | NA |
| **18_0631** | *Mormopterus francoismoutoui* | Trois bassins | NA | 0 | NA |
| **18_0632** | *Mormopterus francoismoutoui* | Trois bassins | NA | 0 | NA |
| **18_0633** | *Mormopterus francoismoutoui* | Trois bassins | NA | 0 | NA |
| **18_0634** | *Mormopterus francoismoutoui* | Trois bassins | NA | 0 | NA |
| **18_0635** | *Mormopterus francoismoutoui* | Trois bassins | NA | 0 | NA |
| **18_0636** | *Mormopterus francoismoutoui* | Trois bassins | NA | 0 | NA |
| **18_0637** | *Mormopterus francoismoutoui* | Trois bassins | NA | 0 | NA |

| | | | | | |
|---|---|---|---|---|---|
| **18_0638** | *Mormopterus francoismoutoui* | Trois bassins | NA | 0 | NA |
| **18_0639** | *Mormopterus francoismoutoui* | Trois bassins | NA | 0 | NA |
| **18_0640** | *Mormopterus francoismoutoui* | Trois bassins | NA | 0 | NA |
| **18_0641** | *Mormopterus francoismoutoui* | Trois bassins | NA | 0 | NA |
| **18_0642** | *Mormopterus francoismoutoui* | Trois bassins | NA | 0 | NA |
| **18_0643** | *Mormopterus francoismoutoui* | Trois bassins | NA | 0 | NA |
| **18_0644** | *Mormopterus francoismoutoui* | Trois bassins | NA | 0 | NA |
| **18_0645** | *Mormopterus francoismoutoui* | Trois bassins | NA | 0 | NA |
| **18_0646** | *Mormopterus francoismoutoui* | Trois bassins | NA | 0 | NA |
| **18_0647** | *Mormopterus francoismoutoui* | Trois bassins | NA | 0 | NA |
| **18_0648** | *Mormopterus francoismoutoui* | Trois bassins | NA | 0 | NA |
| **18_0649** | *Mormopterus francoismoutoui* | Trois bassins | NA | 0 | NA |
| **18_0650** | *Mormopterus francoismoutoui* | Trois bassins | NA | 0 | NA |
| **18_0651** | *Mormopterus francoismoutoui* | Trois bassins | NA | 0 | NA |
| **18_0652** | *Mormopterus francoismoutoui* | Trois bassins | NA | 0 | NA |
| **18_0653** | *Mormopterus francoismoutoui* | Trois bassins | NA | 0 | NA |
| **18_0654** | *Mormopterus francoismoutoui* | Trois bassins | NA | 0 | NA |
| **18_0655** | *Mormopterus francoismoutoui* | Transformateur EDF Gillot | NA | 0 | NA |
| **18_0656** | *Mormopterus francoismoutoui* | Transformateur EDF Gillot | NA | 0 | NA |
| **18_0657** | *Mormopterus francoismoutoui* | Transformateur EDF Gillot | NA | 0 | NA |
| **18_0658** | *Mormopterus francoismoutoui* | Transformateur EDF Gillot | NA | 0 | NA |
| **18_0659** | *Mormopterus francoismoutoui* | Transformateur EDF Gillot | NA | 0 | NA |
| **18_0660** | *Mormopterus francoismoutoui* | Transformateur EDF Gillot | NA | 0 | NA |
| **18_0661** | *Mormopterus francoismoutoui* | Transformateur EDF Gillot | NA | 0 | NA |
| **18_0662** | *Mormopterus francoismoutoui* | Transformateur EDF Gillot | NA | 0 | NA |
| **18_0663** | *Mormopterus francoismoutoui* | Transformateur EDF Gillot | NA | 0 | NA |
| **18_0664** | *Mormopterus francoismoutoui* | Transformateur EDF Gillot | NA | 0 | NA |
| **18_0665** | *Mormopterus francoismoutoui* | Transformateur EDF Gillot | NA | 0 | NA |
| **18_0666** | *Mormopterus francoismoutoui* | Transformateur EDF Gillot | NA | 0 | NA |
| **18_0667** | *Mormopterus francoismoutoui* | Transformateur EDF Gillot | NA | 0 | NA |
| **18_0668** | *Mormopterus francoismoutoui* | Transformateur EDF Gillot | NA | 0 | NA |
| **18_0669** | *Mormopterus francoismoutoui* | Transformateur EDF Gillot | NA | 0 | NA |
| **18_0670** | *Mormopterus francoismoutoui* | Transformateur EDF Gillot | NA | 0 | NA |
| **18_0671** | *Mormopterus francoismoutoui* | Transformateur EDF Gillot | NA | 0 | NA |
| **18_0672** | *Mormopterus francoismoutoui* | Transformateur EDF Gillot | NA | 0 | NA |
| **18_0673** | *Mormopterus francoismoutoui* | Transformateur EDF Gillot | NA | 0 | NA |
| **18_0674** | *Mormopterus francoismoutoui* | Transformateur EDF Gillot | NA | 0 | NA |
| **18_0675** | *Mormopterus francoismoutoui* | Transformateur EDF Gillot | NA | 0 | NA |
| **18_0676** | *Mormopterus francoismoutoui* | Transformateur EDF Gillot | NA | 0 | NA |
| **18_0677** | *Mormopterus francoismoutoui* | Transformateur EDF Gillot | NA | 0 | NA |
| **18_0678** | *Mormopterus francoismoutoui* | Transformateur EDF Gillot | NA | 0 | NA |
| **18_0679** | *Mormopterus francoismoutoui* | Transformateur EDF Gillot | NA | 0 | NA |
| **18_0680** | *Mormopterus francoismoutoui* | Transformateur EDF Gillot | NA | 0 | NA |
| **18_0681** | *Mormopterus francoismoutoui* | Transformateur EDF Gillot | NA | 0 | NA |
| **18_0682** | *Mormopterus francoismoutoui* | Transformateur EDF Gillot | NA | 0 | NA |
| **18_0683** | *Mormopterus francoismoutoui* | Transformateur EDF Gillot | NA | 0 | NA |
| **18_0684** | *Mormopterus francoismoutoui* | Transformateur EDF Gillot | NA | 0 | NA |
| **18_0695** | *Mormopterus francoismoutoui* | Transformateur EDF Gillot | NA | 0 | NA |
| **18_0696** | *Mormopterus francoismoutoui* | Transformateur EDF Gillot | NA | 0 | NA |
| **18_0697** | *Mormopterus francoismoutoui* | Transformateur EDF Gillot | NA | 0 | NA |
| **18_0698** | *Mormopterus francoismoutoui* | Transformateur EDF Gillot | NA | 0 | NA |
| **18_0699** | *Mormopterus francoismoutoui* | Transformateur EDF Gillot | NA | 0 | NA |
| **18_0700** | *Mormopterus francoismoutoui* | Transformateur EDF Gillot | NA | 0 | NA |
| **18_0701** | *Mormopterus francoismoutoui* | Transformateur EDF Gillot | NA | 1 | OR752511 |
| **18_0702** | *Mormopterus francoismoutoui* | Transformateur EDF Gillot | NA | 0 | NA |
| **18_0703** | *Mormopterus francoismoutoui* | Transformateur EDF Gillot | NA | 0 | NA |
| **18_0704** | *Mormopterus francoismoutoui* | Transformateur EDF Gillot | NA | 0 | NA |
| **18_0705** | *Mormopterus francoismoutoui* | Transformateur EDF Gillot | NA | 0 | NA |
| **18_0706** | *Mormopterus francoismoutoui* | Transformateur EDF Gillot | NA | 0 | NA |
| **18_0707** | *Mormopterus francoismoutoui* | Transformateur EDF Gillot | NA | 0 | NA |
| **18_0708** | *Mormopterus francoismoutoui* | Transformateur EDF Gillot | NA | 0 | NA |
| **18_0709** | *Mormopterus francoismoutoui* | Transformateur EDF Gillot | NA | 0 | NA |
| **18_0710** | *Mormopterus francoismoutoui* | Transformateur EDF Gillot | NA | 1 | OR752512 |
| **18_0711** | *Mormopterus francoismoutoui* | Transformateur EDF Gillot | NA | 0 | NA |
| **18_0712** | *Mormopterus francoismoutoui* | Transformateur EDF Gillot | NA | 0 | NA |
| **18_0713** | *Mormopterus francoismoutoui* | Transformateur EDF Gillot | NA | 0 | NA |
| **18_0714** | *Mormopterus francoismoutoui* | Transformateur EDF Gillot | NA | 0 | NA |
| **18_0715** | *Mormopterus francoismoutoui* | Transformateur EDF Gillot | NA | 0 | NA |
| **18_0716** | *Mormopterus francoismoutoui* | Transformateur EDF Gillot | NA | 0 | NA |
| **18_0717** | *Mormopterus francoismoutoui* | Transformateur EDF Gillot | NA | 0 | NA |
| **18_0718** | *Mormopterus francoismoutoui* | Transformateur EDF Gillot | NA | 0 | NA |
| **18_0719** | *Mormopterus francoismoutoui* | Transformateur EDF Gillot | NA | 0 | NA |

| | | | | | |
|---|---|---|---|---|---|
| **18_0720** | *Mormopterus francoismoutoui* | Transformateur EDF Gillot | NA | 0 | NA |
| **18_0721** | *Mormopterus francoismoutoui* | Transformateur EDF Gillot | NA | 0 | NA |
| **18_0722** | *Mormopterus francoismoutoui* | Transformateur EDF Gillot | NA | 0 | NA |
| **18_0723** | *Mormopterus francoismoutoui* | Transformateur EDF Gillot | NA | 0 | NA |
| **18_0724** | *Mormopterus francoismoutoui* | Transformateur EDF Gillot | NA | 0 | NA |
| **18_1061** | *Mormopterus francoismoutoui* | Transformateur EDF Gillot | NA | 0 | NA |
| **18_1062** | *Mormopterus francoismoutoui* | Transformateur EDF Gillot | NA | 0 | NA |
| **18_1063** | *Mormopterus francoismoutoui* | Transformateur EDF Gillot | NA | 0 | NA |
| **18_1064** | *Mormopterus francoismoutoui* | Transformateur EDF Gillot | NA | 0 | NA |
| **18_1065** | *Mormopterus francoismoutoui* | Transformateur EDF Gillot | NA | 0 | NA |
| **18_1066** | *Mormopterus francoismoutoui* | Transformateur EDF Gillot | NA | 0 | NA |
| **18_1067** | *Mormopterus francoismoutoui* | Transformateur EDF Gillot | NA | 0 | NA |
| **18_1068** | *Mormopterus francoismoutoui* | Transformateur EDF Gillot | NA | 0 | NA |
| **18_1069** | *Mormopterus francoismoutoui* | Transformateur EDF Gillot | NA | 0 | NA |
| **18_1070** | *Mormopterus francoismoutoui* | Transformateur EDF Gillot | NA | 0 | NA |
| **18_1071** | *Mormopterus francoismoutoui* | Transformateur EDF Gillot | NA | 0 | NA |
| **18_1072** | *Mormopterus francoismoutoui* | Transformateur EDF Gillot | NA | 0 | NA |
| **18_1073** | *Mormopterus francoismoutoui* | Transformateur EDF Gillot | NA | 0 | NA |
| **18_1074** | *Mormopterus francoismoutoui* | Transformateur EDF Gillot | NA | 0 | NA |
| **18_1075** | *Mormopterus francoismoutoui* | Transformateur EDF Gillot | NA | 0 | NA |
| **18_1076** | *Mormopterus francoismoutoui* | Transformateur EDF Gillot | NA | 0 | NA |
| **18_1077** | *Mormopterus francoismoutoui* | Transformateur EDF Gillot | NA | 0 | NA |
| **18_1078** | *Mormopterus francoismoutoui* | Transformateur EDF Gillot | NA | 0 | NA |
| **18_1079** | *Mormopterus francoismoutoui* | Transformateur EDF Gillot | NA | 0 | NA |
| **18_1080** | *Mormopterus francoismoutoui* | Transformateur EDF Gillot | NA | 0 | NA |
| **18_1081** | *Mormopterus francoismoutoui* | Transformateur EDF Gillot | NA | 0 | NA |
| **18_1082** | *Mormopterus francoismoutoui* | Transformateur EDF Gillot | NA | 0 | NA |
| **18_1083** | *Mormopterus francoismoutoui* | Transformateur EDF Gillot | NA | 0 | NA |
| **18_1084** | *Mormopterus francoismoutoui* | Transformateur EDF Gillot | NA | 0 | NA |
| **18_1085** | *Mormopterus francoismoutoui* | Transformateur EDF Gillot | NA | 0 | NA |
| **18_1086** | *Mormopterus francoismoutoui* | Transformateur EDF Gillot | NA | 0 | NA |
| **18_1087** | *Mormopterus francoismoutoui* | Transformateur EDF Gillot | NA | 0 | NA |
| **18_1088** | *Mormopterus francoismoutoui* | Transformateur EDF Gillot | NA | 0 | NA |
| **18_1089** | *Mormopterus francoismoutoui* | Transformateur EDF Gillot | NA | 0 | NA |
| **18_1090** | *Mormopterus francoismoutoui* | Transformateur EDF Gillot | NA | 0 | NA |
| **18_1101** | *Mormopterus francoismoutoui* | Transformateur EDF Gillot | NA | 0 | NA |
| **18_1102** | *Mormopterus francoismoutoui* | Transformateur EDF Gillot | NA | 0 | NA |
| **18_1103** | *Mormopterus francoismoutoui* | Transformateur EDF Gillot | NA | 0 | NA |
| **18_1104** | *Mormopterus francoismoutoui* | Transformateur EDF Gillot | NA | 0 | NA |
| **18_1105** | *Mormopterus francoismoutoui* | Transformateur EDF Gillot | NA | 0 | NA |
| **18_1106** | *Mormopterus francoismoutoui* | Transformateur EDF Gillot | NA | 1 | OR752513 |
| **18_1107** | *Mormopterus francoismoutoui* | Transformateur EDF Gillot | NA | 0 | NA |
| **18_1108** | *Mormopterus francoismoutoui* | Transformateur EDF Gillot | NA | 0 | NA |
| **18_1109** | *Mormopterus francoismoutoui* | Transformateur EDF Gillot | NA | 0 | NA |
| **18_1110** | *Mormopterus francoismoutoui* | Transformateur EDF Gillot | NA | 0 | NA |
| **18_1111** | *Mormopterus francoismoutoui* | Transformateur EDF Gillot | NA | 0 | NA |
| **18_1112** | *Mormopterus francoismoutoui* | Transformateur EDF Gillot | NA | 0 | NA |
| **18_1113** | *Mormopterus francoismoutoui* | Transformateur EDF Gillot | NA | 0 | NA |
| **18_1114** | *Mormopterus francoismoutoui* | Transformateur EDF Gillot | NA | 1 | OR752514 |
| **18_1115** | *Mormopterus francoismoutoui* | Transformateur EDF Gillot | NA | 0 | NA |
| **18_1116** | *Mormopterus francoismoutoui* | Transformateur EDF Gillot | NA | 0 | NA |
| **18_1117** | *Mormopterus francoismoutoui* | Transformateur EDF Gillot | NA | 0 | NA |
| **18_1118** | *Mormopterus francoismoutoui* | Transformateur EDF Gillot | NA | 0 | NA |
| **18_1119** | *Mormopterus francoismoutoui* | Transformateur EDF Gillot | NA | 1 | OR752515 |
| **18_1120** | *Mormopterus francoismoutoui* | Transformateur EDF Gillot | NA | 0 | NA |
| **18_1121** | *Mormopterus francoismoutoui* | Transformateur EDF Gillot | NA | 0 | NA |
| **18_1122** | *Mormopterus francoismoutoui* | Transformateur EDF Gillot | NA | 0 | NA |
| **18_1123** | *Mormopterus francoismoutoui* | Transformateur EDF Gillot | NA | 0 | NA |
| **18_1124** | *Mormopterus francoismoutoui* | Transformateur EDF Gillot | NA | 0 | NA |
| **18_1125** | *Mormopterus francoismoutoui* | Transformateur EDF Gillot | NA | 0 | NA |
| **18_1126** | *Mormopterus francoismoutoui* | Transformateur EDF Gillot | NA | 0 | NA |
| **18_1127** | *Mormopterus francoismoutoui* | Transformateur EDF Gillot | NA | 0 | NA |
| **18_1128** | *Mormopterus francoismoutoui* | Transformateur EDF Gillot | NA | 0 | NA |
| **18_1129** | *Mormopterus francoismoutoui* | Transformateur EDF Gillot | NA | 0 | NA |
| **18_1130** | *Mormopterus francoismoutoui* | Transformateur EDF Gillot | NA | 0 | NA |
| **18_1141** | *Mormopterus francoismoutoui* | Transformateur EDF Gillot | NA | 0 | NA |
| **18_1142** | *Mormopterus francoismoutoui* | Transformateur EDF Gillot | NA | 0 | NA |
| **18_1143** | *Mormopterus francoismoutoui* | Transformateur EDF Gillot | NA | 0 | NA |
| **18_1144** | *Mormopterus francoismoutoui* | Transformateur EDF Gillot | NA | 0 | NA |
| **18_1145** | *Mormopterus francoismoutoui* | Transformateur EDF Gillot | NA | 0 | NA |
| **18_1146** | *Mormopterus francoismoutoui* | Transformateur EDF Gillot | NA | 0 | NA |
| **18_1147** | *Mormopterus francoismoutoui* | Transformateur EDF Gillot | NA | 0 | NA |

| | | | | | |
|---|---|---|---|---|---|
| **18_1148** | *Mormopterus francoismoutoui* | Transformateur EDF Gillot | NA | 0 | NA |
| **18_1149** | *Mormopterus francoismoutoui* | Transformateur EDF Gillot | NA | 0 | NA |
| **18_1150** | *Mormopterus francoismoutoui* | Transformateur EDF Gillot | NA | 0 | NA |
| **18_1151** | *Mormopterus francoismoutoui* | Transformateur EDF Gillot | NA | 0 | NA |
| **18_1152** | *Mormopterus francoismoutoui* | Transformateur EDF Gillot | NA | 0 | NA |
| **18_1153** | *Mormopterus francoismoutoui* | Transformateur EDF Gillot | NA | 0 | NA |
| **18_1154** | *Mormopterus francoismoutoui* | Transformateur EDF Gillot | NA | 0 | NA |
| **18_1155** | *Mormopterus francoismoutoui* | Transformateur EDF Gillot | NA | 0 | NA |
| **18_1156** | *Mormopterus francoismoutoui* | Transformateur EDF Gillot | NA | 0 | NA |
| **18_1157** | *Mormopterus francoismoutoui* | Transformateur EDF Gillot | NA | 0 | NA |
| **18_1158** | *Mormopterus francoismoutoui* | Transformateur EDF Gillot | NA | 0 | NA |
| **18_1159** | *Mormopterus francoismoutoui* | Transformateur EDF Gillot | NA | 0 | NA |
| **18_1160** | *Mormopterus francoismoutoui* | Transformateur EDF Gillot | NA | 0 | NA |
| **18_1161** | *Mormopterus francoismoutoui* | Transformateur EDF Gillot | NA | 0 | NA |
| **18_1162** | *Mormopterus francoismoutoui* | Transformateur EDF Gillot | NA | 0 | NA |
| **18_1163** | *Mormopterus francoismoutoui* | Transformateur EDF Gillot | NA | 0 | NA |
| **18_1164** | *Mormopterus francoismoutoui* | Transformateur EDF Gillot | NA | 0 | NA |
| **18_1165** | *Mormopterus francoismoutoui* | Transformateur EDF Gillot | NA | 0 | NA |
| **18_1166** | *Mormopterus francoismoutoui* | Transformateur EDF Gillot | NA | 0 | NA |
| **18_1167** | *Mormopterus francoismoutoui* | Transformateur EDF Gillot | NA | 0 | NA |
| **18_1168** | *Mormopterus francoismoutoui* | Transformateur EDF Gillot | NA | 0 | NA |
| **18_1169** | *Mormopterus francoismoutoui* | Transformateur EDF Gillot | NA | 0 | NA |
| **18_1170** | *Mormopterus francoismoutoui* | Transformateur EDF Gillot | NA | 0 | NA |
| **18_1181** | *Mormopterus francoismoutoui* | Transformateur EDF Gillot | NA | 0 | NA |
| **18_1182** | *Mormopterus francoismoutoui* | Transformateur EDF Gillot | NA | 0 | NA |
| **18_1183** | *Mormopterus francoismoutoui* | Transformateur EDF Gillot | NA | 0 | NA |
| **18_1184** | *Mormopterus francoismoutoui* | Transformateur EDF Gillot | NA | 0 | NA |
| **18_1185** | *Mormopterus francoismoutoui* | Transformateur EDF Gillot | NA | 0 | NA |
| **18_1186** | *Mormopterus francoismoutoui* | Transformateur EDF Gillot | NA | 0 | NA |
| **18_1187** | *Mormopterus francoismoutoui* | Transformateur EDF Gillot | NA | 0 | NA |
| **18_1188** | *Mormopterus francoismoutoui* | Transformateur EDF Gillot | NA | 0 | NA |
| **18_1189** | *Mormopterus francoismoutoui* | Transformateur EDF Gillot | NA | 0 | NA |
| **18_1190** | *Mormopterus francoismoutoui* | Transformateur EDF Gillot | NA | 0 | NA |
| **18_1191** | *Mormopterus francoismoutoui* | Transformateur EDF Gillot | NA | 0 | NA |
| **18_1192** | *Mormopterus francoismoutoui* | Transformateur EDF Gillot | NA | 0 | NA |
| **18_1193** | *Mormopterus francoismoutoui* | Transformateur EDF Gillot | NA | 0 | NA |
| **18_1194** | *Mormopterus francoismoutoui* | Transformateur EDF Gillot | NA | 0 | NA |
| **18_1195** | *Mormopterus francoismoutoui* | Transformateur EDF Gillot | NA | 0 | NA |
| **18_1196** | *Mormopterus francoismoutoui* | Transformateur EDF Gillot | NA | 1 | OR752516 |
| **18_1197** | *Mormopterus francoismoutoui* | Transformateur EDF Gillot | NA | 0 | NA |
| **18_1198** | *Mormopterus francoismoutoui* | Transformateur EDF Gillot | NA | 0 | NA |
| **18_1199** | *Mormopterus francoismoutoui* | Transformateur EDF Gillot | NA | 0 | NA |
| **18_1200** | *Mormopterus francoismoutoui* | Transformateur EDF Gillot | NA | 0 | NA |
| **18_1201** | *Mormopterus francoismoutoui* | Transformateur EDF Gillot | NA | 0 | NA |
| **18_1202** | *Mormopterus francoismoutoui* | Transformateur EDF Gillot | NA | 0 | NA |
| **18_1203** | *Mormopterus francoismoutoui* | Transformateur EDF Gillot | NA | 0 | NA |
| **18_1204** | *Mormopterus francoismoutoui* | Transformateur EDF Gillot | NA | 0 | NA |
| **18_1205** | *Mormopterus francoismoutoui* | Transformateur EDF Gillot | NA | 0 | NA |
| **18_1206** | *Mormopterus francoismoutoui* | Transformateur EDF Gillot | NA | 0 | NA |
| **18_1207** | *Mormopterus francoismoutoui* | Transformateur EDF Gillot | NA | 0 | NA |
| **18_1208** | *Mormopterus francoismoutoui* | Transformateur EDF Gillot | NA | 0 | NA |
| **18_1209** | *Mormopterus francoismoutoui* | Transformateur EDF Gillot | NA | 0 | NA |
| **18_1210** | *Mormopterus francoismoutoui* | Transformateur EDF Gillot | NA | 0 | NA |
| **18_1211** | *Mormopterus francoismoutoui* | Trois bassins | NA | 0 | NA |
| **18_1212** | *Mormopterus francoismoutoui* | Trois bassins | NA | 0 | NA |
| **18_1213** | *Mormopterus francoismoutoui* | Trois bassins | NA | 0 | NA |
| **18_1214** | *Mormopterus francoismoutoui* | Trois bassins | NA | 0 | NA |
| **18_1215** | *Mormopterus francoismoutoui* | Trois bassins | NA | 0 | NA |
| **18_1216** | *Mormopterus francoismoutoui* | Trois bassins | NA | 0 | NA |
| **18_1217** | *Mormopterus francoismoutoui* | Trois bassins | NA | 0 | NA |
| **18_1218** | *Mormopterus francoismoutoui* | Trois bassins | NA | 0 | NA |
| **18_1219** | *Mormopterus francoismoutoui* | Trois bassins | NA | 0 | NA |
| **18_1220** | *Mormopterus francoismoutoui* | Trois bassins | NA | 0 | NA |
| **18_1221** | *Mormopterus francoismoutoui* | Trois bassins | NA | 0 | NA |
| **18_1222** | *Mormopterus francoismoutoui* | Trois bassins | NA | 0 | NA |
| **18_1223** | *Mormopterus francoismoutoui* | Trois bassins | NA | 0 | NA |
| **18_1224** | *Mormopterus francoismoutoui* | Trois bassins | NA | 0 | NA |
| **18_1225** | *Mormopterus francoismoutoui* | Trois bassins | NA | 0 | NA |
| **18_1226** | *Mormopterus francoismoutoui* | Trois bassins | NA | 0 | NA |
| **18_1227** | *Mormopterus francoismoutoui* | Trois bassins | NA | 0 | NA |
| **18_1228** | *Mormopterus francoismoutoui* | Trois bassins | NA | 0 | NA |
| **18_1229** | *Mormopterus francoismoutoui* | Trois bassins | NA | 0 | NA |

| | | | | | |
|---|---|---|---|---|---|
| **18_1230** | *Mormopterus francoismoutoui* | Trois bassins | NA | 0 | NA |
| **18_1231** | *Mormopterus francoismoutoui* | Trois bassins | NA | 0 | NA |
| **18_1232** | *Mormopterus francoismoutoui* | Trois bassins | NA | 0 | NA |
| **18_1234** | *Mormopterus francoismoutoui* | Trois bassins | NA | 0 | NA |
| **18_1235** | *Mormopterus francoismoutoui* | Trois bassins | NA | 0 | NA |
| **18_1236** | *Mormopterus francoismoutoui* | Trois bassins | NA | 0 | NA |
| **18_1237** | *Mormopterus francoismoutoui* | Trois bassins | NA | 0 | NA |
| **18_1238** | *Mormopterus francoismoutoui* | Trois bassins | NA | 0 | NA |
| **18_1239** | *Mormopterus francoismoutoui* | Trois bassins | NA | 0 | NA |
| **18_1240** | *Mormopterus francoismoutoui* | Trois bassins | NA | 0 | NA |
| **18_1241** | *Mormopterus francoismoutoui* | Trois bassins | NA | 0 | NA |
| **18_1242** | *Mormopterus francoismoutoui* | Trois bassins | NA | 0 | NA |
| **18_1243** | *Mormopterus francoismoutoui* | Trois bassins | NA | 0 | NA |
| **18_1244** | *Mormopterus francoismoutoui* | Trois bassins | NA | 0 | NA |
| **18_1245** | *Mormopterus francoismoutoui* | Trois bassins | NA | 0 | NA |
| **18_1246** | *Mormopterus francoismoutoui* | Trois bassins | NA | 0 | NA |
| **18_1247** | *Mormopterus francoismoutoui* | Trois bassins | NA | 0 | NA |
| **18_1248** | *Mormopterus francoismoutoui* | Trois bassins | NA | 0 | NA |
| **18_1249** | *Mormopterus francoismoutoui* | Trois bassins | NA | 0 | NA |
| **18_1250** | *Mormopterus francoismoutoui* | Trois bassins | NA | 0 | NA |
| **18_1251** | *Mormopterus francoismoutoui* | Trois bassins | NA | 0 | NA |
| **18_1252** | *Mormopterus francoismoutoui* | Trois bassins | NA | 0 | NA |
| **18_1253** | *Mormopterus francoismoutoui* | Trois bassins | NA | 0 | NA |
| **18_1254** | *Mormopterus francoismoutoui* | Trois bassins | NA | 0 | NA |
| **18_1255** | *Mormopterus francoismoutoui* | Trois bassins | NA | 0 | NA |
| **18_1256** | *Mormopterus francoismoutoui* | Trois bassins | NA | 0 | NA |
| **18_1257** | *Mormopterus francoismoutoui* | Trois bassins | NA | 0 | NA |
| **18_1258** | *Mormopterus francoismoutoui* | Trois bassins | NA | 0 | NA |
| **18_1259** | *Mormopterus francoismoutoui* | Trois bassins | NA | 0 | NA |
| **18_1260** | *Mormopterus francoismoutoui* | Trois bassins | NA | 0 | NA |
| **18_1261** | *Mormopterus francoismoutoui* | Trois bassins | NA | 0 | NA |
| **18_1262** | *Mormopterus francoismoutoui* | Transformateur EDF Gillot | NA | 0 | NA |
| **18_1263** | *Mormopterus francoismoutoui* | Transformateur EDF Gillot | NA | 0 | NA |
| **18_1264** | *Mormopterus francoismoutoui* | Transformateur EDF Gillot | NA | 0 | NA |
| **18_1265** | *Mormopterus francoismoutoui* | Transformateur EDF Gillot | NA | 0 | NA |
| **18_1266** | *Mormopterus francoismoutoui* | Transformateur EDF Gillot | NA | 0 | NA |
| **18_1267** | *Mormopterus francoismoutoui* | Transformateur EDF Gillot | NA | 0 | NA |
| **18_1268** | *Mormopterus francoismoutoui* | Transformateur EDF Gillot | NA | 0 | NA |
| **18_1269** | *Mormopterus francoismoutoui* | Transformateur EDF Gillot | NA | 0 | NA |
| **18_1270** | *Mormopterus francoismoutoui* | Transformateur EDF Gillot | NA | 0 | NA |
| **18_1271** | *Mormopterus francoismoutoui* | Transformateur EDF Gillot | NA | 0 | NA |
| **18_1272** | *Mormopterus francoismoutoui* | Transformateur EDF Gillot | NA | 0 | NA |
| **18_1273** | *Mormopterus francoismoutoui* | Transformateur EDF Gillot | NA | 0 | NA |
| **18_1274** | *Mormopterus francoismoutoui* | Transformateur EDF Gillot | NA | 0 | NA |
| **18_1275** | *Mormopterus francoismoutoui* | Transformateur EDF Gillot | NA | 0 | NA |
| **18_1276** | *Mormopterus francoismoutoui* | Transformateur EDF Gillot | NA | 0 | NA |
| **18_1277** | *Mormopterus francoismoutoui* | Transformateur EDF Gillot | NA | 0 | NA |
| **18_1278** | *Mormopterus francoismoutoui* | Transformateur EDF Gillot | NA | 0 | NA |
| **18_1279** | *Mormopterus francoismoutoui* | Transformateur EDF Gillot | NA | 0 | NA |
| **18_1280** | *Mormopterus francoismoutoui* | Transformateur EDF Gillot | NA | 0 | NA |
| **18_1281** | *Mormopterus francoismoutoui* | Transformateur EDF Gillot | NA | 0 | NA |
| **18_1282** | *Mormopterus francoismoutoui* | Transformateur EDF Gillot | NA | 0 | NA |
| **18_1283** | *Mormopterus francoismoutoui* | Transformateur EDF Gillot | NA | 0 | NA |
| **18_1284** | *Mormopterus francoismoutoui* | Transformateur EDF Gillot | NA | 0 | NA |
| **18_1285** | *Mormopterus francoismoutoui* | Transformateur EDF Gillot | NA | 0 | NA |
| **18_1286** | *Mormopterus francoismoutoui* | Transformateur EDF Gillot | NA | 0 | NA |
| **18_1287** | *Mormopterus francoismoutoui* | Transformateur EDF Gillot | NA | 0 | NA |
| **18_1288** | *Mormopterus francoismoutoui* | Transformateur EDF Gillot | NA | 0 | NA |
| **18_1289** | *Mormopterus francoismoutoui* | Transformateur EDF Gillot | NA | 0 | NA |
| **18_1290** | *Mormopterus francoismoutoui* | Transformateur EDF Gillot | NA | 0 | NA |
| **18_1291** | *Mormopterus francoismoutoui* | Transformateur EDF Gillot | NA | 0 | NA |
| **18_1292** | *Mormopterus francoismoutoui* | Trois bassins | NA | 0 | NA |
| **18_1293** | *Mormopterus francoismoutoui* | Trois bassins | NA | 0 | NA |
| **18_1294** | *Mormopterus francoismoutoui* | Trois bassins | NA | 0 | NA |
| **18_1295** | *Mormopterus francoismoutoui* | Trois bassins | NA | 0 | NA |
| **18_1296** | *Mormopterus francoismoutoui* | Trois bassins | NA | 0 | NA |
| **18_1297** | *Mormopterus francoismoutoui* | Trois bassins | NA | 0 | NA |
| **18_1298** | *Mormopterus francoismoutoui* | Trois bassins | NA | 0 | NA |
| **18_1299** | *Mormopterus francoismoutoui* | Trois bassins | NA | 0 | NA |
| **18_1300** | *Mormopterus francoismoutoui* | Trois bassins | NA | 0 | NA |
| **18_1301** | *Mormopterus francoismoutoui* | Trois bassins | NA | 0 | NA |
| **18_1302** | *Mormopterus francoismoutoui* | Trois bassins | NA | 0 | NA |

| | | | | | |
|---|---|---|---|---|---|
| **18_1303** | *Mormopterus francoismoutoui* | Trois bassins | NA | 0 | NA |
| **18_1304** | *Mormopterus francoismoutoui* | Trois bassins | NA | 0 | NA |
| **18_1305** | *Mormopterus francoismoutoui* | Trois bassins | NA | 0 | NA |
| **18_1306** | *Mormopterus francoismoutoui* | Trois bassins | NA | 0 | NA |
| **18_1307** | *Mormopterus francoismoutoui* | Trois bassins | NA | 0 | NA |
| **18_1308** | *Mormopterus francoismoutoui* | Trois bassins | NA | 0 | NA |
| **18_1309** | *Mormopterus francoismoutoui* | Trois bassins | NA | 0 | NA |
| **18_1310** | *Mormopterus francoismoutoui* | Trois bassins | NA | 0 | NA |
| **18_1311** | *Mormopterus francoismoutoui* | Trois bassins | NA | 0 | NA |
| **18_1312** | *Mormopterus francoismoutoui* | Trois bassins | NA | 0 | NA |
| **18_1313** | *Mormopterus francoismoutoui* | Trois bassins | NA | 0 | NA |
| **18_1314** | *Mormopterus francoismoutoui* | Trois bassins | NA | 0 | NA |
| **18_1315** | *Mormopterus francoismoutoui* | Trois bassins | NA | 0 | NA |
| **18_1316** | *Mormopterus francoismoutoui* | Trois bassins | NA | 0 | NA |
| **18_1317** | *Mormopterus francoismoutoui* | Trois bassins | NA | 0 | NA |
| **18_1318** | *Mormopterus francoismoutoui* | Trois bassins | NA | 0 | NA |
| **18_1319** | *Mormopterus francoismoutoui* | Trois bassins | NA | 0 | NA |
| **18_1320** | *Mormopterus francoismoutoui* | Trois bassins | NA | 0 | NA |
| **18_1321** | *Mormopterus francoismoutoui* | Trois bassins | NA | 0 | NA |
| **18_1322** | *Mormopterus francoismoutoui* | Trois bassins | NA | 0 | NA |
| **18_1323** | *Mormopterus francoismoutoui* | Trois bassins | NA | 0 | NA |
| **18_1324** | *Mormopterus francoismoutoui* | Trois bassins | NA | 0 | NA |
| **18_1325** | *Mormopterus francoismoutoui* | Trois bassins | NA | 0 | NA |
| **18_1326** | *Mormopterus francoismoutoui* | Trois bassins | NA | 0 | NA |
| **18_1327** | *Mormopterus francoismoutoui* | Trois bassins | NA | 0 | NA |
| **18_1328** | *Mormopterus francoismoutoui* | Trois bassins | NA | 0 | NA |
| **18_1329** | *Mormopterus francoismoutoui* | Trois bassins | NA | 0 | NA |
| **18_1330** | *Mormopterus francoismoutoui* | Trois bassins | NA | 0 | NA |
| **18_1331** | *Mormopterus francoismoutoui* | Trois bassins | NA | 0 | NA |
| **18_1332** | *Mormopterus francoismoutoui* | Trois bassins | NA | 0 | NA |
| **18_1333** | *Mormopterus francoismoutoui* | Trois bassins | NA | 0 | NA |
| **18_1334** | *Mormopterus francoismoutoui* | Trois bassins | NA | 0 | NA |
| **18_1335** | *Mormopterus francoismoutoui* | Trois bassins | NA | 0 | NA |
| **18_1336** | *Mormopterus francoismoutoui* | Trois bassins | NA | 0 | NA |
| **18_1337** | *Mormopterus francoismoutoui* | Trois bassins | NA | 0 | NA |
| **18_1338** | *Mormopterus francoismoutoui* | Trois bassins | NA | 0 | NA |
| **18_1339** | *Mormopterus francoismoutoui* | Trois bassins | NA | 0 | NA |
| **18_1340** | *Mormopterus francoismoutoui* | Trois bassins | NA | 0 | NA |
| **18_1341** | *Mormopterus francoismoutoui* | Trois bassins | NA | 0 | NA |
| **18_1342** | *Mormopterus francoismoutoui* | Transformateur EDF Gillot | NA | 0 | NA |
| **18_1343** | *Mormopterus francoismoutoui* | Transformateur EDF Gillot | NA | 0 | NA |
| **18_1344** | *Mormopterus francoismoutoui* | Transformateur EDF Gillot | NA | 0 | NA |
| **18_1345** | *Mormopterus francoismoutoui* | Transformateur EDF Gillot | NA | 0 | NA |
| **18_1346** | *Mormopterus francoismoutoui* | Transformateur EDF Gillot | NA | 0 | NA |
| **18_1347** | *Mormopterus francoismoutoui* | Transformateur EDF Gillot | NA | 0 | NA |
| **18_1348** | *Mormopterus francoismoutoui* | Transformateur EDF Gillot | NA | 0 | NA |
| **18_1349** | *Mormopterus francoismoutoui* | Transformateur EDF Gillot | NA | 0 | NA |
| **18_1350** | *Mormopterus francoismoutoui* | Transformateur EDF Gillot | NA | 0 | NA |
| **18_1351** | *Mormopterus francoismoutoui* | Transformateur EDF Gillot | NA | 0 | NA |
| **18_1352** | *Mormopterus francoismoutoui* | Transformateur EDF Gillot | NA | 0 | NA |
| **18_1353** | *Mormopterus francoismoutoui* | Transformateur EDF Gillot | NA | 0 | NA |
| **18_1354** | *Mormopterus francoismoutoui* | Transformateur EDF Gillot | NA | 0 | NA |
| **18_1355** | *Mormopterus francoismoutoui* | Transformateur EDF Gillot | NA | 0 | NA |
| **18_1356** | *Mormopterus francoismoutoui* | Transformateur EDF Gillot | NA | 0 | NA |
| **18_1357** | *Mormopterus francoismoutoui* | Transformateur EDF Gillot | NA | 0 | NA |
| **18_1358** | *Mormopterus francoismoutoui* | Transformateur EDF Gillot | NA | 0 | NA |
| **18_1359** | *Mormopterus francoismoutoui* | Transformateur EDF Gillot | NA | 0 | NA |
| **18_1360** | *Mormopterus francoismoutoui* | Transformateur EDF Gillot | NA | 0 | NA |
| **18_1361** | *Mormopterus francoismoutoui* | Transformateur EDF Gillot | NA | 0 | NA |
| **18_1362** | *Mormopterus francoismoutoui* | Transformateur EDF Gillot | NA | 0 | NA |
| **18_1363** | *Mormopterus francoismoutoui* | Transformateur EDF Gillot | NA | 0 | NA |
| **18_1364** | *Mormopterus francoismoutoui* | Transformateur EDF Gillot | NA | 0 | NA |
| **18_1365** | *Mormopterus francoismoutoui* | Transformateur EDF Gillot | NA | 0 | NA |
| **18_1366** | *Mormopterus francoismoutoui* | Transformateur EDF Gillot | NA | 0 | NA |
| **18_1367** | *Mormopterus francoismoutoui* | Transformateur EDF Gillot | NA | 0 | NA |
| **18_1368** | *Mormopterus francoismoutoui* | Transformateur EDF Gillot | NA | 0 | NA |
| **18_1369** | *Mormopterus francoismoutoui* | Transformateur EDF Gillot | NA | 0 | NA |
| **18_1370** | *Mormopterus francoismoutoui* | Transformateur EDF Gillot | NA | 0 | NA |
| **18_1371** | *Mormopterus francoismoutoui* | Transformateur EDF Gillot | NA | 0 | NA |
| **18_1417** | *Mormopterus francoismoutoui* | Trois bassins | NA | 0 | NA |
| **18_1418** | *Mormopterus francoismoutoui* | Trois bassins | NA | 0 | NA |
| **18_1419** | *Mormopterus francoismoutoui* | Trois bassins | NA | 0 | NA |

| | | | | | |
|---|---|---|---|---|---|
| **18_1420** | *Mormopterus francoismoutoui* | Trois bassins | NA | 0 | NA |
| **18_1421** | *Mormopterus francoismoutoui* | Trois bassins | NA | 0 | NA |
| **18_1422** | *Mormopterus francoismoutoui* | Trois bassins | NA | 0 | NA |
| **18_1423** | *Mormopterus francoismoutoui* | Trois bassins | NA | 0 | NA |
| **18_1424** | *Mormopterus francoismoutoui* | Trois bassins | NA | 0 | NA |
| **18_1425** | *Mormopterus francoismoutoui* | Trois bassins | NA | 0 | NA |
| **18_1426** | *Mormopterus francoismoutoui* | Trois bassins | NA | 0 | NA |
| **18_1427** | *Mormopterus francoismoutoui* | Trois bassins | NA | 0 | NA |
| **18_1428** | *Mormopterus francoismoutoui* | Trois bassins | NA | 0 | NA |
| **18_1429** | *Mormopterus francoismoutoui* | Trois bassins | NA | 0 | NA |
| **18_1430** | *Mormopterus francoismoutoui* | Trois bassins | NA | 0 | NA |
| **18_1431** | *Mormopterus francoismoutoui* | Trois bassins | NA | 0 | NA |
| **18_1432** | *Mormopterus francoismoutoui* | Trois bassins | NA | 0 | NA |
| **18_1433** | *Mormopterus francoismoutoui* | Trois bassins | NA | 0 | NA |
| **18_1434** | *Mormopterus francoismoutoui* | Trois bassins | NA | 0 | NA |
| **18_1435** | *Mormopterus francoismoutoui* | Trois bassins | NA | 0 | NA |
| **18_1436** | *Mormopterus francoismoutoui* | Trois bassins | NA | 0 | NA |
| **18_1437** | *Mormopterus francoismoutoui* | Trois bassins | NA | 0 | NA |
| **18_1438** | *Mormopterus francoismoutoui* | Trois bassins | NA | 0 | NA |
| **18_1439** | *Mormopterus francoismoutoui* | Trois bassins | NA | 0 | NA |
| **18_1440** | *Mormopterus francoismoutoui* | Trois bassins | NA | 0 | NA |
| **18_1441** | *Mormopterus francoismoutoui* | Trois bassins | NA | 0 | NA |
| **18_1442** | *Mormopterus francoismoutoui* | Trois bassins | NA | 0 | NA |
| **18_1443** | *Mormopterus francoismoutoui* | Trois bassins | NA | 0 | NA |
| **18_1444** | *Mormopterus francoismoutoui* | Trois bassins | NA | 0 | NA |
| **18_1445** | *Mormopterus francoismoutoui* | Trois bassins | NA | 0 | NA |
| **18_1446** | *Mormopterus francoismoutoui* | Trois bassins | NA | 0 | NA |
| **18_1447** | *Mormopterus francoismoutoui* | Trois bassins | NA | 0 | NA |
| **18_1448** | *Mormopterus francoismoutoui* | Trois bassins | NA | 0 | NA |
| **18_1449** | *Mormopterus francoismoutoui* | Trois bassins | NA | 0 | NA |
| **18_1450** | *Mormopterus francoismoutoui* | Trois bassins | NA | 0 | NA |
| **18_1451** | *Mormopterus francoismoutoui* | Trois bassins | NA | 0 | NA |
| **18_1452** | *Mormopterus francoismoutoui* | Trois bassins | NA | 0 | NA |
| **18_1453** | *Mormopterus francoismoutoui* | Trois bassins | NA | 0 | NA |
| **18_1454** | *Mormopterus francoismoutoui* | Trois bassins | NA | 0 | NA |
| **18_1455** | *Mormopterus francoismoutoui* | Trois bassins | NA | 0 | NA |
| **18_1456** | *Mormopterus francoismoutoui* | Trois bassins | NA | 0 | NA |
| **18_1457** | *Mormopterus francoismoutoui* | Trois bassins | NA | 0 | NA |
| **18_1458** | *Mormopterus francoismoutoui* | Trois bassins | NA | 0 | NA |
| **18_1459** | *Mormopterus francoismoutoui* | Trois bassins | NA | 0 | NA |
| **18_1460** | *Mormopterus francoismoutoui* | Trois bassins | NA | 0 | NA |
| **18_1461** | *Mormopterus francoismoutoui* | Trois bassins | NA | 0 | NA |
| **18_1462** | *Mormopterus francoismoutoui* | Trois bassins | NA | 0 | NA |
| **18_1463** | *Mormopterus francoismoutoui* | Trois bassins | NA | 0 | NA |
| **18_1464** | *Mormopterus francoismoutoui* | Trois bassins | NA | 0 | NA |
| **18_1465** | *Mormopterus francoismoutoui* | Trois bassins | NA | 0 | NA |
| **18_1466** | *Mormopterus francoismoutoui* | Trois bassins | NA | 0 | NA |
| **18_1467** | *Mormopterus francoismoutoui* | Transformateur EDF Gillot | NA | 0 | NA |
| **18_1468** | *Mormopterus francoismoutoui* | Transformateur EDF Gillot | NA | 0 | NA |
| **18_1469** | *Mormopterus francoismoutoui* | Transformateur EDF Gillot | NA | 0 | NA |
| **18_1470** | *Mormopterus francoismoutoui* | Transformateur EDF Gillot | NA | 0 | NA |
| **18_1471** | *Mormopterus francoismoutoui* | Transformateur EDF Gillot | NA | 0 | NA |
| **18_1472** | *Mormopterus francoismoutoui* | Transformateur EDF Gillot | NA | 0 | NA |
| **18_1473** | *Mormopterus francoismoutoui* | Transformateur EDF Gillot | NA | 0 | NA |
| **18_1474** | *Mormopterus francoismoutoui* | Transformateur EDF Gillot | NA | 0 | NA |
| **18_1475** | *Mormopterus francoismoutoui* | Transformateur EDF Gillot | NA | 0 | NA |
| **18_1476** | *Mormopterus francoismoutoui* | Transformateur EDF Gillot | NA | 0 | NA |
| **18_1477** | *Mormopterus francoismoutoui* | Transformateur EDF Gillot | NA | 0 | NA |
| **18_1478** | *Mormopterus francoismoutoui* | Transformateur EDF Gillot | NA | 0 | NA |
| **18_1479** | *Mormopterus francoismoutoui* | Transformateur EDF Gillot | NA | 0 | NA |
| **18_1480** | *Mormopterus francoismoutoui* | Transformateur EDF Gillot | NA | 0 | NA |
| **18_1481** | *Mormopterus francoismoutoui* | Transformateur EDF Gillot | NA | 0 | NA |
| **18_1482** | *Mormopterus francoismoutoui* | Transformateur EDF Gillot | NA | 0 | NA |
| **18_1483** | *Mormopterus francoismoutoui* | Transformateur EDF Gillot | NA | 0 | NA |
| **18_1484** | *Mormopterus francoismoutoui* | Transformateur EDF Gillot | NA | 0 | NA |
| **18_1485** | *Mormopterus francoismoutoui* | Transformateur EDF Gillot | NA | 0 | NA |
| **18_1486** | *Mormopterus francoismoutoui* | Transformateur EDF Gillot | NA | 0 | NA |
| **18_1487** | *Mormopterus francoismoutoui* | Transformateur EDF Gillot | NA | 0 | NA |
| **18_1488** | *Mormopterus francoismoutoui* | Transformateur EDF Gillot | NA | 0 | NA |
| **18_1489** | *Mormopterus francoismoutoui* | Transformateur EDF Gillot | NA | 0 | NA |
| **18_1490** | *Mormopterus francoismoutoui* | Transformateur EDF Gillot | NA | 0 | NA |
| **18_1491** | *Mormopterus francoismoutoui* | Transformateur EDF Gillot | NA | 0 | NA |

| | | | | | |
|---|---|---|---|---|---|
| **18_1492** | *Mormopterus francoismoutoui* | Transformateur EDF Gillot | NA | 0 | NA |
| **18_1493** | *Mormopterus francoismoutoui* | Transformateur EDF Gillot | NA | 0 | NA |
| **18_1494** | *Mormopterus francoismoutoui* | Transformateur EDF Gillot | NA | 0 | NA |
| **18_1495** | *Mormopterus francoismoutoui* | Transformateur EDF Gillot | NA | 0 | NA |
| **18_1496** | *Mormopterus francoismoutoui* | Transformateur EDF Gillot | NA | 0 | NA |
| **19_0001** | *Mormopterus francoismoutoui* | Trois bassins | NA | 0 | NA |
| **19_0002** | *Mormopterus francoismoutoui* | Trois bassins | NA | 0 | NA |
| **19_0003** | *Mormopterus francoismoutoui* | Trois bassins | NA | 0 | NA |
| **19_0004** | *Mormopterus francoismoutoui* | Trois bassins | NA | 0 | NA |
| **19_0005** | *Mormopterus francoismoutoui* | Trois bassins | NA | 0 | NA |
| **19_0006** | *Mormopterus francoismoutoui* | Trois bassins | NA | 0 | NA |
| **19_0007** | *Mormopterus francoismoutoui* | Trois bassins | NA | 0 | NA |
| **19_0008** | *Mormopterus francoismoutoui* | Trois bassins | NA | 0 | NA |
| **19_0009** | *Mormopterus francoismoutoui* | Trois bassins | NA | 0 | NA |
| **19_0010** | *Mormopterus francoismoutoui* | Trois bassins | NA | 0 | NA |
| **19_0011** | *Mormopterus francoismoutoui* | Trois bassins | NA | 0 | NA |
| **19_0012** | *Mormopterus francoismoutoui* | Trois bassins | NA | 0 | NA |
| **19_0013** | *Mormopterus francoismoutoui* | Trois bassins | NA | 0 | NA |
| **19_0014** | *Mormopterus francoismoutoui* | Trois bassins | NA | 0 | NA |
| **19_0015** | *Mormopterus francoismoutoui* | Trois bassins | NA | 0 | NA |
| **19_0016** | *Mormopterus francoismoutoui* | Trois bassins | NA | 0 | NA |
| **19_0017** | *Mormopterus francoismoutoui* | Trois bassins | NA | 0 | NA |
| **19_0018** | *Mormopterus francoismoutoui* | Trois bassins | NA | 0 | NA |
| **19_0019** | *Mormopterus francoismoutoui* | Trois bassins | NA | 0 | NA |
| **19_0020** | *Mormopterus francoismoutoui* | Trois bassins | NA | 0 | NA |
| **19_0021** | *Mormopterus francoismoutoui* | Trois bassins | NA | 0 | NA |
| **19_0022** | *Mormopterus francoismoutoui* | Trois bassins | NA | 0 | NA |
| **19_0023** | *Mormopterus francoismoutoui* | Trois bassins | NA | 0 | NA |
| **19_0024** | *Mormopterus francoismoutoui* | Trois bassins | NA | 0 | NA |
| **19_0025** | *Mormopterus francoismoutoui* | Trois bassins | NA | 0 | NA |
| **19_0026** | *Mormopterus francoismoutoui* | Trois bassins | NA | 0 | NA |
| **19_0027** | *Mormopterus francoismoutoui* | Trois bassins | NA | 0 | NA |
| **19_0028** | *Mormopterus francoismoutoui* | Trois bassins | NA | 0 | NA |
| **19_0029** | *Mormopterus francoismoutoui* | Trois bassins | NA | 0 | NA |
| **19_0030** | *Mormopterus francoismoutoui* | Trois bassins | NA | 0 | NA |
| **19_0031** | *Mormopterus francoismoutoui* | Trois bassins | NA | 0 | NA |
| **19_0032** | *Mormopterus francoismoutoui* | Trois bassins | NA | 1 | OR752517 |
| **19_0033** | *Mormopterus francoismoutoui* | Trois bassins | NA | 0 | NA |
| **19_0034** | *Mormopterus francoismoutoui* | Trois bassins | NA | 0 | NA |
| **19_0035** | *Mormopterus francoismoutoui* | Trois bassins | NA | 0 | NA |
| **19_0036** | *Mormopterus francoismoutoui* | Trois bassins | NA | 0 | NA |
| **19_0037** | *Mormopterus francoismoutoui* | Trois bassins | NA | 0 | NA |
| **19_0038** | *Mormopterus francoismoutoui* | Trois bassins | NA | 0 | NA |
| **19_0039** | *Mormopterus francoismoutoui* | Trois bassins | NA | 0 | NA |
| **19_0040** | *Mormopterus francoismoutoui* | Trois bassins | NA | 0 | NA |
| **19_0041** | *Mormopterus francoismoutoui* | Trois bassins | NA | 0 | NA |
| **19_0042** | *Mormopterus francoismoutoui* | Trois bassins | NA | 0 | NA |
| **19_0043** | *Mormopterus francoismoutoui* | Trois bassins | NA | 0 | NA |
| **19_0044** | *Mormopterus francoismoutoui* | Trois bassins | NA | 0 | NA |
| **19_0045** | *Mormopterus francoismoutoui* | Trois bassins | NA | 0 | NA |
| **19_0046** | *Mormopterus francoismoutoui* | Trois bassins | NA | 0 | NA |
| **19_0047** | *Mormopterus francoismoutoui* | Trois bassins | NA | 0 | NA |
| **19_0048** | *Mormopterus francoismoutoui* | Trois bassins | NA | 0 | NA |
| **19_0049** | *Mormopterus francoismoutoui* | Trois bassins | NA | 0 | NA |
| **19_0050** | *Mormopterus francoismoutoui* | Trois bassins | NA | 0 | NA |
| **19_0051** | *Mormopterus francoismoutoui* | Transformateur EDF Gillot | NA | 0 | NA |
| **19_0052** | *Mormopterus francoismoutoui* | Transformateur EDF Gillot | NA | 0 | NA |
| **19_0053** | *Mormopterus francoismoutoui* | Transformateur EDF Gillot | NA | 0 | NA |
| **19_0054** | *Mormopterus francoismoutoui* | Transformateur EDF Gillot | NA | 0 | NA |
| **19_0055** | *Mormopterus francoismoutoui* | Transformateur EDF Gillot | NA | 0 | NA |
| **19_0056** | *Mormopterus francoismoutoui* | Transformateur EDF Gillot | NA | 0 | NA |
| **19_0057** | *Mormopterus francoismoutoui* | Transformateur EDF Gillot | NA | 0 | NA |
| **19_0058** | *Mormopterus francoismoutoui* | Transformateur EDF Gillot | NA | 0 | NA |
| **19_0059** | *Mormopterus francoismoutoui* | Transformateur EDF Gillot | NA | 0 | NA |
| **19_0060** | *Mormopterus francoismoutoui* | Transformateur EDF Gillot | NA | 0 | NA |
| **19_0061** | *Mormopterus francoismoutoui* | Transformateur EDF Gillot | NA | 0 | NA |
| **19_0062** | *Mormopterus francoismoutoui* | Transformateur EDF Gillot | NA | 0 | NA |
| **19_0063** | *Mormopterus francoismoutoui* | Transformateur EDF Gillot | NA | 0 | NA |
| **19_0064** | *Mormopterus francoismoutoui* | Transformateur EDF Gillot | NA | 0 | NA |
| **19_0065** | *Mormopterus francoismoutoui* | Transformateur EDF Gillot | NA | 0 | NA |
| **19_0066** | *Mormopterus francoismoutoui* | Transformateur EDF Gillot | NA | 0 | NA |
| **19_0067** | *Mormopterus francoismoutoui* | Transformateur EDF Gillot | NA | 0 | NA |

| | | | | | |
|---|---|---|---|---|---|
| **19_0068** | *Mormopterus francoismoutoui* | Transformateur EDF Gillot | NA | 0 | NA |
| **19_0069** | *Mormopterus francoismoutoui* | Transformateur EDF Gillot | NA | 0 | NA |
| **19_0070** | *Mormopterus francoismoutoui* | Transformateur EDF Gillot | NA | 0 | NA |
| **19_0071** | *Mormopterus francoismoutoui* | Transformateur EDF Gillot | NA | 0 | NA |
| **19_0072** | *Mormopterus francoismoutoui* | Transformateur EDF Gillot | NA | 0 | NA |
| **19_0073** | *Mormopterus francoismoutoui* | Transformateur EDF Gillot | NA | 0 | NA |
| **19_0074** | *Mormopterus francoismoutoui* | Transformateur EDF Gillot | NA | 0 | NA |
| **19_0075** | *Mormopterus francoismoutoui* | Transformateur EDF Gillot | NA | 0 | NA |
| **19_0076** | *Mormopterus francoismoutoui* | Transformateur EDF Gillot | NA | 0 | NA |
| **19_0077** | *Mormopterus francoismoutoui* | Transformateur EDF Gillot | NA | 0 | NA |
| **19_0078** | *Mormopterus francoismoutoui* | Transformateur EDF Gillot | NA | 0 | NA |
| **19_0079** | *Mormopterus francoismoutoui* | Transformateur EDF Gillot | NA | 0 | NA |
| **19_0080** | *Mormopterus francoismoutoui* | Transformateur EDF Gillot | NA | 0 | NA |
| **19_0082** | *Mormopterus francoismoutoui* | Trois bassins | NA | 0 | NA |
| **19_0083** | *Mormopterus francoismoutoui* | Trois bassins | NA | 0 | NA |
| **19_0084** | *Mormopterus francoismoutoui* | Trois bassins | NA | 0 | NA |
| **19_0085** | *Mormopterus francoismoutoui* | Trois bassins | NA | 0 | NA |
| **19_0086** | *Mormopterus francoismoutoui* | Trois bassins | NA | 0 | NA |
| **19_0087** | *Mormopterus francoismoutoui* | Trois bassins | NA | 0 | NA |
| **19_0088** | *Mormopterus francoismoutoui* | Trois bassins | NA | 0 | NA |
| **19_0089** | *Mormopterus francoismoutoui* | Trois bassins | NA | 0 | NA |
| **19_0090** | *Mormopterus francoismoutoui* | Trois bassins | NA | 0 | NA |
| **19_0091** | *Mormopterus francoismoutoui* | Trois bassins | NA | 0 | NA |
| **19_0092** | *Mormopterus francoismoutoui* | Trois bassins | NA | 0 | NA |
| **19_0093** | *Mormopterus francoismoutoui* | Trois bassins | NA | 0 | NA |
| **19_0094** | *Mormopterus francoismoutoui* | Trois bassins | NA | 0 | NA |
| **19_0095** | *Mormopterus francoismoutoui* | Trois bassins | NA | 0 | NA |
| **19_0096** | *Mormopterus francoismoutoui* | Trois bassins | NA | 0 | NA |
| **19_0097** | *Mormopterus francoismoutoui* | Trois bassins | NA | 0 | NA |
| **19_0098** | *Mormopterus francoismoutoui* | Trois bassins | NA | 0 | NA |
| **19_0099** | *Mormopterus francoismoutoui* | Trois bassins | NA | 0 | NA |
| **19_0100** | *Mormopterus francoismoutoui* | Trois bassins | NA | 0 | NA |
| **19_0101** | *Mormopterus francoismoutoui* | Trois bassins | NA | 0 | NA |
| **19_0102** | *Mormopterus francoismoutoui* | Trois bassins | NA | 0 | NA |
| **19_0103** | *Mormopterus francoismoutoui* | Trois bassins | NA | 0 | NA |
| **19_0104** | *Mormopterus francoismoutoui* | Trois bassins | NA | 0 | NA |
| **19_0105** | *Mormopterus francoismoutoui* | Trois bassins | NA | 0 | NA |
| **19_0106** | *Mormopterus francoismoutoui* | Trois bassins | NA | 0 | NA |
| **19_0107** | *Mormopterus francoismoutoui* | Trois bassins | NA | 0 | NA |
| **19_0108** | *Mormopterus francoismoutoui* | Trois bassins | NA | 0 | NA |
| **19_0109** | *Mormopterus francoismoutoui* | Trois bassins | NA | 0 | NA |
| **19_0110** | *Mormopterus francoismoutoui* | Trois bassins | NA | 0 | NA |
| **19_0111** | *Mormopterus francoismoutoui* | Trois bassins | NA | 0 | NA |
| **19_0112** | *Mormopterus francoismoutoui* | Trois bassins | NA | 0 | NA |
| **19_0113** | *Mormopterus francoismoutoui* | Trois bassins | NA | 0 | NA |
| **19_0114** | *Mormopterus francoismoutoui* | Trois bassins | NA | 0 | NA |
| **19_0115** | *Mormopterus francoismoutoui* | Trois bassins | NA | 0 | NA |
| **19_0116** | *Mormopterus francoismoutoui* | Trois bassins | NA | 0 | NA |
| **19_0117** | *Mormopterus francoismoutoui* | Trois bassins | NA | 0 | NA |
| **19_0118** | *Mormopterus francoismoutoui* | Trois bassins | NA | 0 | NA |
| **19_0119** | *Mormopterus francoismoutoui* | Trois bassins | NA | 0 | NA |
| **19_0120** | *Mormopterus francoismoutoui* | Trois bassins | NA | 0 | NA |
| **19_0121** | *Mormopterus francoismoutoui* | Trois bassins | NA | 0 | NA |
| **19_0122** | *Mormopterus francoismoutoui* | Trois bassins | NA | 0 | NA |
| **19_0123** | *Mormopterus francoismoutoui* | Trois bassins | NA | 0 | NA |
| **19_0124** | *Mormopterus francoismoutoui* | Trois bassins | NA | 0 | NA |
| **19_0125** | *Mormopterus francoismoutoui* | Trois bassins | NA | 0 | NA |
| **19_0126** | *Mormopterus francoismoutoui* | Trois bassins | NA | 0 | NA |
| **19_0127** | *Mormopterus francoismoutoui* | Trois bassins | NA | 0 | NA |
| **19_0128** | *Mormopterus francoismoutoui* | Trois bassins | NA | 0 | NA |
| **19_0129** | *Mormopterus francoismoutoui* | Trois bassins | NA | 0 | NA |
| **19_0130** | *Mormopterus francoismoutoui* | Trois bassins | NA | 0 | NA |
| **19_0131** | *Mormopterus francoismoutoui* | Trois bassins | NA | 0 | NA |
| **19_0132** | *Mormopterus francoismoutoui* | Transformateur EDF Gillot | NA | 0 | NA |
| **19_0133** | *Mormopterus francoismoutoui* | Transformateur EDF Gillot | NA | 0 | NA |
| **19_0134** | *Mormopterus francoismoutoui* | Transformateur EDF Gillot | NA | 0 | NA |
| **19_0135** | *Mormopterus francoismoutoui* | Transformateur EDF Gillot | NA | 0 | NA |
| **19_0136** | *Mormopterus francoismoutoui* | Transformateur EDF Gillot | NA | 0 | NA |
| **19_0137** | *Mormopterus francoismoutoui* | Transformateur EDF Gillot | NA | 0 | NA |
| **19_0138** | *Mormopterus francoismoutoui* | Transformateur EDF Gillot | NA | 0 | NA |
| **19_0139** | *Mormopterus francoismoutoui* | Transformateur EDF Gillot | NA | 0 | NA |
| **19_0140** | *Mormopterus francoismoutoui* | Transformateur EDF Gillot | NA | 0 | NA |

| | | | | | |
|---|---|---|---|---|---|
| **19_0141** | *Mormopterus francoismoutoui* | Transformateur EDF Gillot | NA | 0 | NA |
| **19_0142** | *Mormopterus francoismoutoui* | Transformateur EDF Gillot | NA | 0 | NA |
| **19_0143** | *Mormopterus francoismoutoui* | Transformateur EDF Gillot | NA | 0 | NA |
| **19_0144** | *Mormopterus francoismoutoui* | Transformateur EDF Gillot | NA | 0 | NA |
| **19_0145** | *Mormopterus francoismoutoui* | Transformateur EDF Gillot | NA | 0 | NA |
| **19_0146** | *Mormopterus francoismoutoui* | Transformateur EDF Gillot | NA | 0 | NA |
| **19_0147** | *Mormopterus francoismoutoui* | Transformateur EDF Gillot | NA | 0 | NA |
| **19_0148** | *Mormopterus francoismoutoui* | Transformateur EDF Gillot | NA | 0 | NA |
| **19_0149** | *Mormopterus francoismoutoui* | Transformateur EDF Gillot | NA | 0 | NA |
| **19_0150** | *Mormopterus francoismoutoui* | Transformateur EDF Gillot | NA | 0 | NA |
| **19_0151** | *Mormopterus francoismoutoui* | Transformateur EDF Gillot | NA | 0 | NA |
| **19_0152** | *Mormopterus francoismoutoui* | Transformateur EDF Gillot | NA | 0 | NA |
| **19_0153** | *Mormopterus francoismoutoui* | Transformateur EDF Gillot | NA | 0 | NA |
| **19_0155** | *Mormopterus francoismoutoui* | Transformateur EDF Gillot | NA | 0 | NA |
| **19_0156** | *Mormopterus francoismoutoui* | Transformateur EDF Gillot | NA | 0 | NA |
| **19_0157** | *Mormopterus francoismoutoui* | Transformateur EDF Gillot | NA | 0 | NA |
| **19_0158** | *Mormopterus francoismoutoui* | Transformateur EDF Gillot | NA | 0 | NA |
| **19_0159** | *Mormopterus francoismoutoui* | Transformateur EDF Gillot | NA | 0 | NA |
| **19_0160** | *Mormopterus francoismoutoui* | Transformateur EDF Gillot | NA | 0 | NA |
| **19_0161** | *Mormopterus francoismoutoui* | Transformateur EDF Gillot | NA | 0 | NA |
| **19_0162** | *Mormopterus francoismoutoui* | Transformateur EDF Gillot | NA | 0 | NA |
| **19_0163** | *Mormopterus francoismoutoui* | Trois bassins | NA | 0 | NA |
| **19_0164** | *Mormopterus francoismoutoui* | Trois bassins | NA | 0 | NA |
| **19_0165** | *Mormopterus francoismoutoui* | Trois bassins | NA | 0 | NA |
| **19_0166** | *Mormopterus francoismoutoui* | Trois bassins | NA | 0 | NA |
| **19_0167** | *Mormopterus francoismoutoui* | Trois bassins | NA | 0 | NA |
| **19_0168** | *Mormopterus francoismoutoui* | Trois bassins | NA | 0 | NA |
| **19_0169** | *Mormopterus francoismoutoui* | Trois bassins | NA | 0 | NA |
| **19_0170** | *Mormopterus francoismoutoui* | Trois bassins | NA | 0 | NA |
| **19_0171** | *Mormopterus francoismoutoui* | Trois bassins | NA | 0 | NA |
| **19_0172** | *Mormopterus francoismoutoui* | Trois bassins | NA | 0 | NA |
| **19_0173** | *Mormopterus francoismoutoui* | Trois bassins | NA | 0 | NA |
| **19_0174** | *Mormopterus francoismoutoui* | Trois bassins | NA | 0 | NA |
| **19_0175** | *Mormopterus francoismoutoui* | Trois bassins | NA | 0 | NA |
| **19_0176** | *Mormopterus francoismoutoui* | Trois bassins | NA | 0 | NA |
| **19_0177** | *Mormopterus francoismoutoui* | Trois bassins | NA | 0 | NA |
| **19_0178** | *Mormopterus francoismoutoui* | Trois bassins | NA | 0 | NA |
| **19_0179** | *Mormopterus francoismoutoui* | Trois bassins | NA | 0 | NA |
| **19_0180** | *Mormopterus francoismoutoui* | Trois bassins | NA | 0 | NA |
| **19_0181** | *Mormopterus francoismoutoui* | Trois bassins | NA | 0 | NA |
| **19_0182** | *Mormopterus francoismoutoui* | Trois bassins | NA | 0 | NA |
| **19_0183** | *Mormopterus francoismoutoui* | Trois bassins | NA | 0 | NA |
| **19_0184** | *Mormopterus francoismoutoui* | Trois bassins | NA | 0 | NA |
| **19_0185** | *Mormopterus francoismoutoui* | Trois bassins | NA | 0 | NA |
| **19_0186** | *Mormopterus francoismoutoui* | Trois bassins | NA | 0 | NA |
| **19_0187** | *Mormopterus francoismoutoui* | Trois bassins | NA | 0 | NA |
| **19_0188** | *Mormopterus francoismoutoui* | Trois bassins | NA | 0 | NA |
| **19_0189** | *Mormopterus francoismoutoui* | Trois bassins | NA | 0 | NA |
| **19_0190** | *Mormopterus francoismoutoui* | Trois bassins | NA | 0 | NA |
| **19_0191** | *Mormopterus francoismoutoui* | Trois bassins | NA | 0 | NA |
| **19_0192** | *Mormopterus francoismoutoui* | Trois bassins | NA | 0 | NA |
| **19_0193** | *Mormopterus francoismoutoui* | Trois bassins | NA | 0 | NA |
| **19_0194** | *Mormopterus francoismoutoui* | Trois bassins | NA | 0 | NA |
| **19_0195** | *Mormopterus francoismoutoui* | Trois bassins | NA | 0 | NA |
| **19_0196** | *Mormopterus francoismoutoui* | Trois bassins | NA | 0 | NA |
| **19_0197** | *Mormopterus francoismoutoui* | Trois bassins | NA | 0 | NA |
| **19_0198** | *Mormopterus francoismoutoui* | Trois bassins | NA | 0 | NA |
| **19_0199** | *Mormopterus francoismoutoui* | Trois bassins | NA | 0 | NA |
| **19_0200** | *Mormopterus francoismoutoui* | Trois bassins | NA | 0 | NA |
| **19_0201** | *Mormopterus francoismoutoui* | Trois bassins | NA | 0 | NA |
| **19_0202** | *Mormopterus francoismoutoui* | Trois bassins | NA | 0 | NA |
| **19_0203** | *Mormopterus francoismoutoui* | Trois bassins | NA | 0 | NA |
| **19_0204** | *Mormopterus francoismoutoui* | Trois bassins | NA | 0 | NA |
| **19_0205** | *Mormopterus francoismoutoui* | Trois bassins | NA | 0 | NA |
| **19_0206** | *Mormopterus francoismoutoui* | Trois bassins | NA | 0 | NA |
| **19_0207** | *Mormopterus francoismoutoui* | Trois bassins | NA | 0 | NA |
| **19_0208** | *Mormopterus francoismoutoui* | Trois bassins | NA | 0 | NA |
| **19_0209** | *Mormopterus francoismoutoui* | Trois bassins | NA | 0 | NA |
| **19_0210** | *Mormopterus francoismoutoui* | Trois bassins | NA | 0 | NA |
| **19_0211** | *Mormopterus francoismoutoui* | Trois bassins | NA | 0 | NA |
| **19_0212** | *Mormopterus francoismoutoui* | Trois bassins | NA | 0 | NA |
| **19_0213** | *Mormopterus francoismoutoui* | Transformateur EDF Gillot | NA | 0 | NA |

| | | | | | |
|---|---|---|---|---|---|
| **19_0214** | *Mormopterus francoismoutoui* | Transformateur EDF Gillot | NA | 0 | NA |
| **19_0215** | *Mormopterus francoismoutoui* | Transformateur EDF Gillot | NA | 0 | NA |
| **19_0216** | *Mormopterus francoismoutoui* | Transformateur EDF Gillot | NA | 0 | NA |
| **19_0217** | *Mormopterus francoismoutoui* | Transformateur EDF Gillot | NA | 0 | NA |
| **19_0218** | *Mormopterus francoismoutoui* | Transformateur EDF Gillot | NA | 0 | NA |
| **19_0219** | *Mormopterus francoismoutoui* | Transformateur EDF Gillot | NA | 0 | NA |
| **19_0220** | *Mormopterus francoismoutoui* | Transformateur EDF Gillot | NA | 0 | NA |
| **19_0221** | *Mormopterus francoismoutoui* | Transformateur EDF Gillot | NA | 0 | NA |
| **19_0222** | *Mormopterus francoismoutoui* | Transformateur EDF Gillot | NA | 0 | NA |
| **19_0223** | *Mormopterus francoismoutoui* | Transformateur EDF Gillot | NA | 0 | NA |
| **19_0224** | *Mormopterus francoismoutoui* | Transformateur EDF Gillot | NA | 0 | NA |
| **19_0225** | *Mormopterus francoismoutoui* | Transformateur EDF Gillot | NA | 0 | NA |
| **19_0226** | *Mormopterus francoismoutoui* | Transformateur EDF Gillot | NA | 0 | NA |
| **19_0227** | *Mormopterus francoismoutoui* | Transformateur EDF Gillot | NA | 0 | NA |
| **19_0228** | *Mormopterus francoismoutoui* | Transformateur EDF Gillot | NA | 0 | NA |
| **19_0229** | *Mormopterus francoismoutoui* | Transformateur EDF Gillot | NA | 0 | NA |
| **19_0230** | *Mormopterus francoismoutoui* | Transformateur EDF Gillot | NA | 0 | NA |
| **19_0231** | *Mormopterus francoismoutoui* | Transformateur EDF Gillot | NA | 0 | NA |
| **19_0232** | *Mormopterus francoismoutoui* | Transformateur EDF Gillot | NA | 0 | NA |
| **19_0233** | *Mormopterus francoismoutoui* | Transformateur EDF Gillot | NA | 0 | NA |
| **19_0234** | *Mormopterus francoismoutoui* | Transformateur EDF Gillot | NA | 0 | NA |
| **19_0235** | *Mormopterus francoismoutoui* | Transformateur EDF Gillot | NA | 0 | NA |
| **19_0236** | *Mormopterus francoismoutoui* | Transformateur EDF Gillot | NA | 0 | NA |
| **19_0237** | *Mormopterus francoismoutoui* | Transformateur EDF Gillot | NA | 0 | NA |
| **19_0238** | *Mormopterus francoismoutoui* | Transformateur EDF Gillot | NA | 0 | NA |
| **19_0239** | *Mormopterus francoismoutoui* | Transformateur EDF Gillot | NA | 0 | NA |
| **19_0240** | *Mormopterus francoismoutoui* | Transformateur EDF Gillot | NA | 0 | NA |
| **19_0241** | *Mormopterus francoismoutoui* | Transformateur EDF Gillot | NA | 0 | NA |
| **19_0242** | *Mormopterus francoismoutoui* | Transformateur EDF Gillot | NA | 0 | NA |
| **19_0401** | *Mormopterus francoismoutoui* | Trois bassins | NA | 0 | NA |
| **19_0402** | *Mormopterus francoismoutoui* | Trois bassins | NA | 0 | NA |
| **19_0403** | *Mormopterus francoismoutoui* | Trois bassins | NA | 0 | NA |
| **19_0404** | *Mormopterus francoismoutoui* | Trois bassins | NA | 0 | NA |
| **19_0405** | *Mormopterus francoismoutoui* | Trois bassins | NA | 0 | NA |
| **19_0406** | *Mormopterus francoismoutoui* | Trois bassins | NA | 0 | NA |
| **19_0407** | *Mormopterus francoismoutoui* | Trois bassins | NA | 0 | NA |
| **19_0408** | *Mormopterus francoismoutoui* | Trois bassins | NA | 0 | NA |
| **19_0409** | *Mormopterus francoismoutoui* | Trois bassins | NA | 0 | NA |
| **19_0410** | *Mormopterus francoismoutoui* | Trois bassins | NA | 0 | NA |
| **19_0411** | *Mormopterus francoismoutoui* | Trois bassins | NA | 0 | NA |
| **19_0412** | *Mormopterus francoismoutoui* | Trois bassins | NA | 0 | NA |
| **19_0413** | *Mormopterus francoismoutoui* | Trois bassins | NA | 0 | NA |
| **19_0414** | *Mormopterus francoismoutoui* | Trois bassins | NA | 0 | NA |
| **19_0415** | *Mormopterus francoismoutoui* | Trois bassins | NA | 0 | NA |
| **19_0416** | *Mormopterus francoismoutoui* | Trois bassins | NA | 0 | NA |
| **19_0417** | *Mormopterus francoismoutoui* | Trois bassins | NA | 0 | NA |
| **19_0418** | *Mormopterus francoismoutoui* | Trois bassins | NA | 0 | NA |
| **19_0419** | *Mormopterus francoismoutoui* | Trois bassins | NA | 0 | NA |
| **19_0420** | *Mormopterus francoismoutoui* | Trois bassins | NA | 0 | NA |
| **19_0421** | *Mormopterus francoismoutoui* | Trois bassins | NA | 0 | NA |
| **19_0422** | *Mormopterus francoismoutoui* | Trois bassins | NA | 0 | NA |
| **19_0423** | *Mormopterus francoismoutoui* | Trois bassins | NA | 0 | NA |
| **19_0424** | *Mormopterus francoismoutoui* | Trois bassins | NA | 0 | NA |
| **19_0425** | *Mormopterus francoismoutoui* | Trois bassins | NA | 0 | NA |
| **19_0426** | *Mormopterus francoismoutoui* | Trois bassins | NA | 0 | NA |
| **19_0427** | *Mormopterus francoismoutoui* | Trois bassins | NA | 0 | NA |
| **19_0428** | *Mormopterus francoismoutoui* | Trois bassins | NA | 0 | NA |
| **19_0429** | *Mormopterus francoismoutoui* | Trois bassins | NA | 0 | NA |
| **19_0430** | *Mormopterus francoismoutoui* | Trois bassins | NA | 0 | NA |
| **19_0431** | *Mormopterus francoismoutoui* | Trois bassins | NA | 0 | NA |
| **19_0432** | *Mormopterus francoismoutoui* | Trois bassins | NA | 0 | NA |
| **19_0433** | *Mormopterus francoismoutoui* | Trois bassins | NA | 0 | NA |
| **19_0434** | *Mormopterus francoismoutoui* | Trois bassins | NA | 0 | NA |
| **19_0435** | *Mormopterus francoismoutoui* | Trois bassins | NA | 0 | NA |
| **19_0436** | *Mormopterus francoismoutoui* | Trois bassins | NA | 0 | NA |
| **19_0437** | *Mormopterus francoismoutoui* | Trois bassins | NA | 0 | NA |
| **19_0438** | *Mormopterus francoismoutoui* | Trois bassins | NA | 0 | NA |
| **19_0439** | *Mormopterus francoismoutoui* | Trois bassins | NA | 0 | NA |
| **19_0440** | *Mormopterus francoismoutoui* | Trois bassins | NA | 0 | NA |
| **19_0441** | *Mormopterus francoismoutoui* | Trois bassins | NA | 0 | NA |
| **19_0442** | *Mormopterus francoismoutoui* | Trois bassins | NA | 0 | NA |
| **19_0443** | *Mormopterus francoismoutoui* | Trois bassins | NA | 0 | NA |

| | | | | | |
|---|---|---|---|---|---|
| **19_0444** | *Mormopterus francoismoutoui* | Trois bassins | NA | 0 | NA |
| **19_0445** | *Mormopterus francoismoutoui* | Trois bassins | NA | 0 | NA |
| **19_0446** | *Mormopterus francoismoutoui* | Trois bassins | NA | 0 | NA |
| **19_0447** | *Mormopterus francoismoutoui* | Trois bassins | NA | 0 | NA |
| **19_0448** | *Mormopterus francoismoutoui* | Trois bassins | NA | 0 | NA |
| **19_0449** | *Mormopterus francoismoutoui* | Trois bassins | NA | 0 | NA |
| **19_0450** | *Mormopterus francoismoutoui* | Trois bassins | NA | 0 | NA |
| **19_0451** | *Mormopterus francoismoutoui* | Transformateur EDF Gillot | NA | 0 | NA |
| **19_0452** | *Mormopterus francoismoutoui* | Transformateur EDF Gillot | NA | 0 | NA |
| **19_0453** | *Mormopterus francoismoutoui* | Transformateur EDF Gillot | NA | 0 | NA |
| **19_0454** | *Mormopterus francoismoutoui* | Transformateur EDF Gillot | NA | 0 | NA |
| **19_0455** | *Mormopterus francoismoutoui* | Transformateur EDF Gillot | NA | 0 | NA |
| **19_0456** | *Mormopterus francoismoutoui* | Transformateur EDF Gillot | NA | 0 | NA |
| **19_0457** | *Mormopterus francoismoutoui* | Transformateur EDF Gillot | NA | 0 | NA |
| **19_0458** | *Mormopterus francoismoutoui* | Transformateur EDF Gillot | NA | 0 | NA |
| **19_0459** | *Mormopterus francoismoutoui* | Transformateur EDF Gillot | NA | 0 | NA |
| **19_0460** | *Mormopterus francoismoutoui* | Transformateur EDF Gillot | NA | 0 | NA |
| **19_0461** | *Mormopterus francoismoutoui* | Transformateur EDF Gillot | NA | 0 | NA |
| **19_0462** | *Mormopterus francoismoutoui* | Transformateur EDF Gillot | NA | 0 | NA |
| **19_0463** | *Mormopterus francoismoutoui* | Transformateur EDF Gillot | NA | 0 | NA |
| **19_0464** | *Mormopterus francoismoutoui* | Transformateur EDF Gillot | NA | 0 | NA |
| **19_0465** | *Mormopterus francoismoutoui* | Transformateur EDF Gillot | NA | 0 | NA |
| **19_0466** | *Mormopterus francoismoutoui* | Transformateur EDF Gillot | NA | 0 | NA |
| **19_0467** | *Mormopterus francoismoutoui* | Transformateur EDF Gillot | NA | 0 | NA |
| **19_0468** | *Mormopterus francoismoutoui* | Transformateur EDF Gillot | NA | 0 | NA |
| **19_0469** | *Mormopterus francoismoutoui* | Transformateur EDF Gillot | NA | 0 | NA |
| **19_0470** | *Mormopterus francoismoutoui* | Transformateur EDF Gillot | NA | 0 | NA |
| **19_0471** | *Mormopterus francoismoutoui* | Transformateur EDF Gillot | NA | 0 | NA |
| **19_0472** | *Mormopterus francoismoutoui* | Transformateur EDF Gillot | NA | 0 | NA |
| **19_0473** | *Mormopterus francoismoutoui* | Transformateur EDF Gillot | NA | 0 | NA |
| **19_0474** | *Mormopterus francoismoutoui* | Transformateur EDF Gillot | NA | 0 | NA |
| **19_0475** | *Mormopterus francoismoutoui* | Transformateur EDF Gillot | NA | 0 | NA |
| **19_0476** | *Mormopterus francoismoutoui* | Transformateur EDF Gillot | NA | 0 | NA |
| **19_0477** | *Mormopterus francoismoutoui* | Transformateur EDF Gillot | NA | 0 | NA |
| **19_0478** | *Mormopterus francoismoutoui* | Transformateur EDF Gillot | NA | 0 | NA |
| **19_0479** | *Mormopterus francoismoutoui* | Transformateur EDF Gillot | NA | 0 | NA |
| **19_0480** | *Mormopterus francoismoutoui* | Transformateur EDF Gillot | NA | 0 | NA |
| **19_0481** | *Mormopterus francoismoutoui* | Trois bassins | NA | 0 | NA |
| **19_0482** | *Mormopterus francoismoutoui* | Trois bassins | NA | 0 | NA |
| **19_0483** | *Mormopterus francoismoutoui* | Trois bassins | NA | 0 | NA |
| **19_0484** | *Mormopterus francoismoutoui* | Trois bassins | NA | 0 | NA |
| **19_0485** | *Mormopterus francoismoutoui* | Trois bassins | NA | 0 | NA |
| **19_0486** | *Mormopterus francoismoutoui* | Trois bassins | NA | 0 | NA |
| **19_0487** | *Mormopterus francoismoutoui* | Trois bassins | NA | 0 | NA |
| **19_0488** | *Mormopterus francoismoutoui* | Trois bassins | NA | 0 | NA |
| **19_0489** | *Mormopterus francoismoutoui* | Trois bassins | NA | 0 | NA |
| **19_0490** | *Mormopterus francoismoutoui* | Trois bassins | NA | 0 | NA |
| **19_0491** | *Mormopterus francoismoutoui* | Trois bassins | NA | 0 | NA |
| **19_0492** | *Mormopterus francoismoutoui* | Trois bassins | NA | 0 | NA |
| **19_0493** | *Mormopterus francoismoutoui* | Trois bassins | NA | 0 | NA |
| **19_0494** | *Mormopterus francoismoutoui* | Trois bassins | NA | 0 | NA |
| **19_0495** | *Mormopterus francoismoutoui* | Trois bassins | NA | 0 | NA |
| **19_0496** | *Mormopterus francoismoutoui* | Trois bassins | NA | 0 | NA |
| **19_0497** | *Mormopterus francoismoutoui* | Trois bassins | NA | 0 | NA |
| **19_0498** | *Mormopterus francoismoutoui* | Trois bassins | NA | 0 | NA |
| **19_0499** | *Mormopterus francoismoutoui* | Trois bassins | NA | 0 | NA |
| **19_0500** | *Mormopterus francoismoutoui* | Trois bassins | NA | 0 | NA |
| **19_0501** | *Mormopterus francoismoutoui* | Trois bassins | NA | 0 | NA |
| **19_0502** | *Mormopterus francoismoutoui* | Trois bassins | NA | 0 | NA |
| **19_0503** | *Mormopterus francoismoutoui* | Trois bassins | NA | 0 | NA |
| **19_0504** | *Mormopterus francoismoutoui* | Trois bassins | NA | 0 | NA |
| **19_0505** | *Mormopterus francoismoutoui* | Trois bassins | NA | 0 | NA |
| **19_0506** | *Mormopterus francoismoutoui* | Trois bassins | NA | 0 | NA |
| **19_0507** | *Mormopterus francoismoutoui* | Trois bassins | NA | 0 | NA |
| **19_0508** | *Mormopterus francoismoutoui* | Trois bassins | NA | 0 | NA |
| **19_0509** | *Mormopterus francoismoutoui* | Trois bassins | NA | 0 | NA |
| **19_0510** | *Mormopterus francoismoutoui* | Trois bassins | NA | 0 | NA |
| **19_0511** | *Mormopterus francoismoutoui* | Trois bassins | NA | 0 | NA |
| **19_0512** | *Mormopterus francoismoutoui* | Trois bassins | NA | 0 | NA |
| **19_0513** | *Mormopterus francoismoutoui* | Trois bassins | NA | 0 | NA |
| **19_0514** | *Mormopterus francoismoutoui* | Trois bassins | NA | 0 | NA |
| **19_0515** | *Mormopterus francoismoutoui* | Trois bassins | NA | 0 | NA |

| | | | | | |
|---|---|---|---|---|---|
| **19_0516** | *Mormopterus francoismoutoui* | Trois bassins | NA | 0 | NA |
| **19_0517** | *Mormopterus francoismoutoui* | Trois bassins | NA | 0 | NA |
| **19_0518** | *Mormopterus francoismoutoui* | Trois bassins | NA | 0 | NA |
| **19_0519** | *Mormopterus francoismoutoui* | Trois bassins | NA | 0 | NA |
| **19_0520** | *Mormopterus francoismoutoui* | Trois bassins | NA | 0 | NA |
| **19_0521** | *Mormopterus francoismoutoui* | Trois bassins | NA | 0 | NA |
| **19_0522** | *Mormopterus francoismoutoui* | Trois bassins | NA | 0 | NA |
| **19_0523** | *Mormopterus francoismoutoui* | Trois bassins | NA | 0 | NA |
| **19_0524** | *Mormopterus francoismoutoui* | Trois bassins | NA | 0 | NA |
| **19_0525** | *Mormopterus francoismoutoui* | Trois bassins | NA | 0 | NA |
| **19_0526** | *Mormopterus francoismoutoui* | Trois bassins | NA | 0 | NA |
| **19_0527** | *Mormopterus francoismoutoui* | Trois bassins | NA | 0 | NA |
| **19_0528** | *Mormopterus francoismoutoui* | Trois bassins | NA | 0 | NA |
| **19_0529** | *Mormopterus francoismoutoui* | Trois bassins | NA | 0 | NA |
| **19_0530** | *Mormopterus francoismoutoui* | Trois bassins | NA | 0 | NA |
| **19_0531** | *Mormopterus francoismoutoui* | Transformateur EDF Gillot | NA | 0 | NA |
| **19_0532** | *Mormopterus francoismoutoui* | Transformateur EDF Gillot | NA | 0 | NA |
| **19_0533** | *Mormopterus francoismoutoui* | Transformateur EDF Gillot | NA | 0 | NA |
| **19_0534** | *Mormopterus francoismoutoui* | Transformateur EDF Gillot | NA | 0 | NA |
| **19_0535** | *Mormopterus francoismoutoui* | Transformateur EDF Gillot | NA | 0 | NA |
| **19_0536** | *Mormopterus francoismoutoui* | Transformateur EDF Gillot | NA | 0 | NA |
| **19_0537** | *Mormopterus francoismoutoui* | Transformateur EDF Gillot | NA | 1 | OR752518 |
| **19_0538** | *Mormopterus francoismoutoui* | Transformateur EDF Gillot | NA | 0 | NA |
| **19_0539** | *Mormopterus francoismoutoui* | Transformateur EDF Gillot | NA | 0 | NA |
| **19_0540** | *Mormopterus francoismoutoui* | Transformateur EDF Gillot | NA | 0 | NA |
| **19_0541** | *Mormopterus francoismoutoui* | Transformateur EDF Gillot | NA | 0 | NA |
| **19_0542** | *Mormopterus francoismoutoui* | Transformateur EDF Gillot | NA | 0 | NA |
| **19_0543** | *Mormopterus francoismoutoui* | Transformateur EDF Gillot | NA | 0 | NA |
| **19_0544** | *Mormopterus francoismoutoui* | Transformateur EDF Gillot | NA | 0 | NA |
| **19_0545** | *Mormopterus francoismoutoui* | Transformateur EDF Gillot | NA | 0 | NA |
| **19_0546** | *Mormopterus francoismoutoui* | Transformateur EDF Gillot | NA | 0 | NA |
| **19_0547** | *Mormopterus francoismoutoui* | Transformateur EDF Gillot | NA | 0 | NA |
| **19_0548** | *Mormopterus francoismoutoui* | Transformateur EDF Gillot | NA | 0 | NA |
| **19_0549** | *Mormopterus francoismoutoui* | Transformateur EDF Gillot | NA | 0 | NA |
| **19_0550** | *Mormopterus francoismoutoui* | Transformateur EDF Gillot | NA | 0 | NA |
| **19_0551** | *Mormopterus francoismoutoui* | Trois bassins | NA | 0 | NA |
| **19_0552** | *Mormopterus francoismoutoui* | Trois bassins | NA | 0 | NA |
| **19_0553** | *Mormopterus francoismoutoui* | Trois bassins | NA | 0 | NA |
| **19_0554** | *Mormopterus francoismoutoui* | Trois bassins | NA | 0 | NA |
| **19_0555** | *Mormopterus francoismoutoui* | Trois bassins | NA | 0 | NA |
| **19_0556** | *Mormopterus francoismoutoui* | Trois bassins | NA | 0 | NA |
| **19_0557** | *Mormopterus francoismoutoui* | Trois bassins | NA | 0 | NA |
| **19_0558** | *Mormopterus francoismoutoui* | Trois bassins | NA | 0 | NA |
| **19_0559** | *Mormopterus francoismoutoui* | Trois bassins | NA | 0 | NA |
| **19_0560** | *Mormopterus francoismoutoui* | Trois bassins | NA | 0 | NA |
| **19_0561** | *Mormopterus francoismoutoui* | Trois bassins | NA | 0 | NA |
| **19_0562** | *Mormopterus francoismoutoui* | Trois bassins | NA | 0 | NA |
| **19_0563** | *Mormopterus francoismoutoui* | Trois bassins | NA | 1 | OR752519 |
| **19_0564** | *Mormopterus francoismoutoui* | Trois bassins | NA | 0 | NA |
| **19_0565** | *Mormopterus francoismoutoui* | Trois bassins | NA | 0 | NA |
| **19_0566** | *Mormopterus francoismoutoui* | Trois bassins | NA | 0 | NA |
| **19_0567** | *Mormopterus francoismoutoui* | Trois bassins | NA | 0 | NA |
| **19_0568** | *Mormopterus francoismoutoui* | Trois bassins | NA | 0 | NA |
| **19_0570** | *Mormopterus francoismoutoui* | Trois bassins | NA | 0 | NA |
| **19_0571** | *Mormopterus francoismoutoui* | Trois bassins | NA | 0 | NA |
| **19_0572** | *Mormopterus francoismoutoui* | Trois bassins | NA | 0 | NA |
| **19_0573** | *Mormopterus francoismoutoui* | Trois bassins | NA | 0 | NA |
| **19_0574** | *Mormopterus francoismoutoui* | Trois bassins | NA | 0 | NA |
| **19_0575** | *Mormopterus francoismoutoui* | Trois bassins | NA | 0 | NA |
| **19_0576** | *Mormopterus francoismoutoui* | Trois bassins | NA | 0 | NA |
| **19_0577** | *Mormopterus francoismoutoui* | Trois bassins | NA | 0 | NA |
| **19_0578** | *Mormopterus francoismoutoui* | Trois bassins | NA | 0 | NA |
| **19_0579** | *Mormopterus francoismoutoui* | Trois bassins | NA | 0 | NA |
| **19_0580** | *Mormopterus francoismoutoui* | Trois bassins | NA | 0 | NA |
| **19_0581** | *Mormopterus francoismoutoui* | Trois bassins | NA | 0 | NA |
| **19_0582** | *Mormopterus francoismoutoui* | Trois bassins | NA | 0 | NA |
| **19_0583** | *Mormopterus francoismoutoui* | Trois bassins | NA | 0 | NA |
| **19_0584** | *Mormopterus francoismoutoui* | Trois bassins | NA | 0 | NA |
| **19_0585** | *Mormopterus francoismoutoui* | Trois bassins | NA | 0 | NA |
| **19_0586** | *Mormopterus francoismoutoui* | Trois bassins | NA | 0 | NA |
| **19_0587** | *Mormopterus francoismoutoui* | Trois bassins | NA | 0 | NA |
| **19_0588** | *Mormopterus francoismoutoui* | Trois bassins | NA | 0 | NA |

| | | | | | |
|---|---|---|---|---|---|
| **19_0589** | *Mormopterus francoismoutoui* | Trois bassins | NA | 0 | NA |
| **19_0590** | *Mormopterus francoismoutoui* | Trois bassins | NA | 0 | NA |
| **19_0591** | *Mormopterus francoismoutoui* | Trois bassins | NA | 0 | NA |
| **19_0592** | *Mormopterus francoismoutoui* | Trois bassins | NA | 0 | NA |
| **19_0593** | *Mormopterus francoismoutoui* | Trois bassins | NA | 0 | NA |
| **19_0594** | *Mormopterus francoismoutoui* | Trois bassins | NA | 0 | NA |
| **19_0595** | *Mormopterus francoismoutoui* | Trois bassins | NA | 0 | NA |
| **19_0596** | *Mormopterus francoismoutoui* | Trois bassins | NA | 0 | NA |
| **19_0597** | *Mormopterus francoismoutoui* | Trois bassins | NA | 0 | NA |
| **19_0598** | *Mormopterus francoismoutoui* | Trois bassins | NA | 0 | NA |
| **19_0599** | *Mormopterus francoismoutoui* | Trois bassins | NA | 0 | NA |
| **19_0600** | *Mormopterus francoismoutoui* | Trois bassins | NA | 0 | NA |
| **19_0601** | *Mormopterus francoismoutoui* | Trois bassins | NA | 0 | NA |
| **19_0602** | *Mormopterus francoismoutoui* | Transformateur EDF Gillot | NA | 0 | NA |
| **19_0603** | *Mormopterus francoismoutoui* | Transformateur EDF Gillot | NA | 0 | NA |
| **19_0604** | *Mormopterus francoismoutoui* | Transformateur EDF Gillot | NA | 0 | NA |
| **19_0605** | *Mormopterus francoismoutoui* | Transformateur EDF Gillot | NA | 0 | NA |
| **19_0606** | *Mormopterus francoismoutoui* | Transformateur EDF Gillot | NA | 0 | NA |
| **19_0607** | *Mormopterus francoismoutoui* | Trois bassins | NA | 0 | NA |
| **19_0608** | *Mormopterus francoismoutoui* | Trois bassins | NA | 0 | NA |
| **19_0609** | *Mormopterus francoismoutoui* | Trois bassins | NA | 0 | NA |
| **19_0610** | *Mormopterus francoismoutoui* | Trois bassins | NA | 0 | NA |
| **19_0611** | *Mormopterus francoismoutoui* | Trois bassins | NA | 0 | NA |
| **19_0612** | *Mormopterus francoismoutoui* | Trois bassins | NA | 0 | NA |
| **19_0613** | *Mormopterus francoismoutoui* | Trois bassins | NA | 0 | NA |
| **19_0614** | *Mormopterus francoismoutoui* | Trois bassins | NA | 0 | NA |
| **19_0615** | *Mormopterus francoismoutoui* | Trois bassins | NA | 0 | NA |
| **19_0616** | *Mormopterus francoismoutoui* | Trois bassins | NA | 0 | NA |
| **19_0617** | *Mormopterus francoismoutoui* | Trois bassins | NA | 0 | NA |
| **19_0618** | *Mormopterus francoismoutoui* | Trois bassins | NA | 0 | NA |
| **19_0619** | *Mormopterus francoismoutoui* | Trois bassins | NA | 0 | NA |
| **19_0620** | *Mormopterus francoismoutoui* | Trois bassins | NA | 0 | NA |
| **19_0621** | *Mormopterus francoismoutoui* | Trois bassins | NA | 0 | NA |
| **19_0622** | *Mormopterus francoismoutoui* | Trois bassins | NA | 0 | NA |
| **19_0623** | *Mormopterus francoismoutoui* | Trois bassins | NA | 0 | NA |
| **19_0624** | *Mormopterus francoismoutoui* | Trois bassins | NA | 0 | NA |
| **19_0625** | *Mormopterus francoismoutoui* | Trois bassins | NA | 0 | NA |
| **19_0626** | *Mormopterus francoismoutoui* | Trois bassins | NA | 0 | NA |
| **19_0627** | *Mormopterus francoismoutoui* | Trois bassins | NA | 0 | NA |
| **19_0628** | *Mormopterus francoismoutoui* | Trois bassins | NA | 0 | NA |
| **19_0629** | *Mormopterus francoismoutoui* | Trois bassins | NA | 0 | NA |
| **19_0630** | *Mormopterus francoismoutoui* | Trois bassins | NA | 0 | NA |
| **19_0631** | *Mormopterus francoismoutoui* | Trois bassins | NA | 0 | NA |
| **19_0632** | *Mormopterus francoismoutoui* | Trois bassins | NA | 0 | NA |
| **19_0633** | *Mormopterus francoismoutoui* | Trois bassins | NA | 0 | NA |
| **19_0634** | *Mormopterus francoismoutoui* | Trois bassins | NA | 0 | NA |
| **19_0635** | *Mormopterus francoismoutoui* | Trois bassins | NA | 0 | NA |
| **19_0636** | *Mormopterus francoismoutoui* | Trois bassins | NA | 0 | NA |
| **19_0637** | *Mormopterus francoismoutoui* | Trois bassins | NA | 0 | NA |
| **19_0638** | *Mormopterus francoismoutoui* | Trois bassins | NA | 0 | NA |
| **19_0639** | *Mormopterus francoismoutoui* | Trois bassins | NA | 0 | NA |
| **19_0640** | *Mormopterus francoismoutoui* | Trois bassins | NA | 0 | NA |
| **19_0641** | *Mormopterus francoismoutoui* | Trois bassins | NA | 0 | NA |
| **19_0642** | *Mormopterus francoismoutoui* | Trois bassins | NA | 0 | NA |
| **19_0643** | *Mormopterus francoismoutoui* | Trois bassins | NA | 0 | NA |
| **19_0644** | *Mormopterus francoismoutoui* | Trois bassins | NA | 0 | NA |
| **19_0645** | *Mormopterus francoismoutoui* | Trois bassins | NA | 0 | NA |
| **19_0646** | *Mormopterus francoismoutoui* | Trois bassins | NA | 0 | NA |
| **19_0647** | *Mormopterus francoismoutoui* | Trois bassins | NA | 0 | NA |
| **19_0648** | *Mormopterus francoismoutoui* | Trois bassins | NA | 0 | NA |
| **19_0649** | *Mormopterus francoismoutoui* | Trois bassins | NA | 0 | NA |
| **19_0650** | *Mormopterus francoismoutoui* | Trois bassins | NA | 0 | NA |
| **19_0651** | *Mormopterus francoismoutoui* | Trois bassins | NA | 0 | NA |
| **19_0652** | *Mormopterus francoismoutoui* | Trois bassins | NA | 0 | NA |
| **19_0653** | *Mormopterus francoismoutoui* | Trois bassins | NA | 0 | NA |
| **19_0654** | *Mormopterus francoismoutoui* | Trois bassins | NA | 0 | NA |
| **19_0655** | *Mormopterus francoismoutoui* | Trois bassins | NA | 0 | NA |
| **19_0656** | *Mormopterus francoismoutoui* | Trois bassins | NA | 0 | NA |
| **19_0657** | *Mormopterus francoismoutoui* | Transformateur EDF Gillot | NA | 0 | NA |
| **19_0658** | *Mormopterus francoismoutoui* | Transformateur EDF Gillot | NA | 0 | NA |
| **19_0659** | *Mormopterus francoismoutoui* | Transformateur EDF Gillot | NA | 0 | NA |
| **19_0660** | *Mormopterus francoismoutoui* | Transformateur EDF Gillot | NA | 0 | NA |

| | | | | | |
|---|---|---|---|---|---|
| **19_0661** | *Mormopterus francoismoutoui* | Transformateur EDF Gillot | NA | 0 | NA |
| **19_0662** | *Mormopterus francoismoutoui* | Transformateur EDF Gillot | NA | 0 | NA |
| **19_0663** | *Mormopterus francoismoutoui* | Transformateur EDF Gillot | NA | 0 | NA |
| **19_0664** | *Mormopterus francoismoutoui* | Transformateur EDF Gillot | NA | 0 | NA |
| **19_0665** | *Mormopterus francoismoutoui* | Transformateur EDF Gillot | NA | 0 | NA |
| **19_0666** | *Mormopterus francoismoutoui* | Transformateur EDF Gillot | NA | 0 | NA |
| **19_0667** | *Mormopterus francoismoutoui* | Transformateur EDF Gillot | NA | 0 | NA |
| **19_0668** | *Mormopterus francoismoutoui* | Transformateur EDF Gillot | NA | 0 | NA |
| **19_0669** | *Mormopterus francoismoutoui* | Transformateur EDF Gillot | NA | 0 | NA |
| **19_0670** | *Mormopterus francoismoutoui* | Transformateur EDF Gillot | NA | 0 | NA |
| **19_0671** | *Mormopterus francoismoutoui* | Transformateur EDF Gillot | NA | 0 | NA |
| **19_0672** | *Mormopterus francoismoutoui* | Transformateur EDF Gillot | NA | 0 | NA |
| **19_0673** | *Mormopterus francoismoutoui* | Transformateur EDF Gillot | NA | 0 | NA |
| **19_0674** | *Mormopterus francoismoutoui* | Transformateur EDF Gillot | NA | 0 | NA |
| **19_0675** | *Mormopterus francoismoutoui* | Transformateur EDF Gillot | NA | 0 | NA |
| **19_0676** | *Mormopterus francoismoutoui* | Transformateur EDF Gillot | NA | 0 | NA |
| **19_0677** | *Mormopterus francoismoutoui* | Transformateur EDF Gillot | NA | 0 | NA |
| **19_0678** | *Mormopterus francoismoutoui* | Transformateur EDF Gillot | NA | 0 | NA |
| **19_0679** | *Mormopterus francoismoutoui* | Transformateur EDF Gillot | NA | 0 | NA |
| **19_0680** | *Mormopterus francoismoutoui* | Transformateur EDF Gillot | NA | 0 | NA |
| **19_0681** | *Mormopterus francoismoutoui* | Transformateur EDF Gillot | NA | 0 | NA |
| **19_0682** | *Mormopterus francoismoutoui* | Transformateur EDF Gillot | NA | 0 | NA |
| **19_0683** | *Mormopterus francoismoutoui* | Transformateur EDF Gillot | NA | 0 | NA |
| **19_0684** | *Mormopterus francoismoutoui* | Transformateur EDF Gillot | NA | 0 | NA |
| **19_0685** | *Mormopterus francoismoutoui* | Transformateur EDF Gillot | NA | 0 | NA |
| **19_0686** | *Mormopterus francoismoutoui* | Transformateur EDF Gillot | NA | 0 | NA |
| **19_0687** | *Mormopterus francoismoutoui* | Trois bassins | NA | 0 | NA |
| **19_0688** | *Mormopterus francoismoutoui* | Trois bassins | NA | 0 | NA |
| **19_0689** | *Mormopterus francoismoutoui* | Trois bassins | NA | 0 | NA |
| **19_0690** | *Mormopterus francoismoutoui* | Trois bassins | NA | 0 | NA |
| **19_0691** | *Mormopterus francoismoutoui* | Trois bassins | NA | 0 | NA |
| **19_0692** | *Mormopterus francoismoutoui* | Trois bassins | NA | 0 | NA |
| **19_0693** | *Mormopterus francoismoutoui* | Trois bassins | NA | 0 | NA |
| **19_0694** | *Mormopterus francoismoutoui* | Trois bassins | NA | 0 | NA |
| **19_0695** | *Mormopterus francoismoutoui* | Trois bassins | NA | 0 | NA |
| **19_0696** | *Mormopterus francoismoutoui* | Trois bassins | NA | 0 | NA |
| **19_0697** | *Mormopterus francoismoutoui* | Trois bassins | NA | 0 | NA |
| **19_0698** | *Mormopterus francoismoutoui* | Trois bassins | NA | 0 | NA |
| **19_0699** | *Mormopterus francoismoutoui* | Trois bassins | NA | 1 | OR752520 |
| **19_0700** | *Mormopterus francoismoutoui* | Trois bassins | NA | 0 | NA |
| **19_0701** | *Mormopterus francoismoutoui* | Trois bassins | NA | 0 | NA |
| **19_0702** | *Mormopterus francoismoutoui* | Trois bassins | NA | 0 | NA |
| **19_0703** | *Mormopterus francoismoutoui* | Trois bassins | NA | 0 | NA |
| **19_0704** | *Mormopterus francoismoutoui* | Trois bassins | NA | 0 | NA |
| **19_0705** | *Mormopterus francoismoutoui* | Trois bassins | NA | 0 | NA |
| **19_0706** | *Mormopterus francoismoutoui* | Trois bassins | NA | 1 | OR752521 |
| **19_0707** | *Mormopterus francoismoutoui* | Trois bassins | NA | 0 | NA |
| **19_0708** | *Mormopterus francoismoutoui* | Trois bassins | NA | 0 | NA |
| **19_0709** | *Mormopterus francoismoutoui* | Trois bassins | NA | 0 | NA |
| **19_0710** | *Mormopterus francoismoutoui* | Trois bassins | NA | 0 | NA |
| **19_0711** | *Mormopterus francoismoutoui* | Trois bassins | NA | 0 | NA |
| **19_0712** | *Mormopterus francoismoutoui* | Transformateur EDF Gillot | NA | 0 | NA |
| **19_0713** | *Mormopterus francoismoutoui* | Transformateur EDF Gillot | NA | 0 | NA |
| **19_0714** | *Mormopterus francoismoutoui* | Transformateur EDF Gillot | NA | 0 | NA |
| **19_0715** | *Mormopterus francoismoutoui* | Transformateur EDF Gillot | NA | 0 | NA |
| **19_0716** | *Mormopterus francoismoutoui* | Transformateur EDF Gillot | NA | 0 | NA |
| **19_0717** | *Mormopterus francoismoutoui* | Transformateur EDF Gillot | NA | 0 | NA |
| **19_0718** | *Mormopterus francoismoutoui* | Transformateur EDF Gillot | NA | 0 | NA |
| **19_0719** | *Mormopterus francoismoutoui* | Transformateur EDF Gillot | NA | 0 | NA |
| **19_0720** | *Mormopterus francoismoutoui* | Transformateur EDF Gillot | NA | 0 | NA |
| **19_0884** | *Mormopterus francoismoutoui* | Transformateur EDF Gillot | NA | 0 | NA |
| **19_0885** | *Mormopterus francoismoutoui* | Transformateur EDF Gillot | NA | 0 | NA |
| **19_0886** | *Mormopterus francoismoutoui* | Transformateur EDF Gillot | NA | 0 | NA |
| **19_0887** | *Mormopterus francoismoutoui* | Transformateur EDF Gillot | NA | 0 | NA |
| **19_0888** | *Mormopterus francoismoutoui* | Transformateur EDF Gillot | NA | 0 | NA |
| **19_0889** | *Mormopterus francoismoutoui* | Transformateur EDF Gillot | NA | 0 | NA |
| **19_0890** | *Mormopterus francoismoutoui* | Transformateur EDF Gillot | NA | 0 | NA |
| **19_0891** | *Mormopterus francoismoutoui* | Transformateur EDF Gillot | NA | 0 | NA |
| **19_0892** | *Mormopterus francoismoutoui* | Transformateur EDF Gillot | NA | 0 | NA |
| **19_0893** | *Mormopterus francoismoutoui* | Transformateur EDF Gillot | NA | 0 | NA |
| **19_0894** | *Mormopterus francoismoutoui* | Transformateur EDF Gillot | NA | 0 | NA |
| **19_0895** | *Mormopterus francoismoutoui* | Transformateur EDF Gillot | NA | 0 | NA |

| | | | | | |
|---|---|---|---|---|---|
| **19_0896** | *Mormopterus francoismoutoui* | Transformateur EDF Gillot | NA | 0 | NA |
| **19_0897** | *Mormopterus francoismoutoui* | Transformateur EDF Gillot | NA | 0 | NA |
| **19_0898** | *Mormopterus francoismoutoui* | Transformateur EDF Gillot | NA | 0 | NA |
| **19_0899** | *Mormopterus francoismoutoui* | Transformateur EDF Gillot | NA | 0 | NA |
| **19_0900** | *Mormopterus francoismoutoui* | Transformateur EDF Gillot | NA | 0 | NA |
| **19_0901** | *Mormopterus francoismoutoui* | Transformateur EDF Gillot | NA | 0 | NA |
| **19_0902** | *Mormopterus francoismoutoui* | Transformateur EDF Gillot | NA | 0 | NA |
| **19_0903** | *Mormopterus francoismoutoui* | Transformateur EDF Gillot | NA | 0 | NA |
| **19_0904** | *Mormopterus francoismoutoui* | Transformateur EDF Gillot | NA | 0 | NA |
| **19_0905** | *Mormopterus francoismoutoui* | Transformateur EDF Gillot | NA | 0 | NA |
| **19_0906** | *Mormopterus francoismoutoui* | Transformateur EDF Gillot | NA | 0 | NA |
| **19_0907** | *Mormopterus francoismoutoui* | Transformateur EDF Gillot | NA | 0 | NA |
| **19_0908** | *Mormopterus francoismoutoui* | Transformateur EDF Gillot | NA | 0 | NA |
| **19_0909** | *Mormopterus francoismoutoui* | Transformateur EDF Gillot | NA | 0 | NA |
| **19_0910** | *Mormopterus francoismoutoui* | Transformateur EDF Gillot | NA | 0 | NA |
| **19_0911** | *Mormopterus francoismoutoui* | Transformateur EDF Gillot | NA | 0 | NA |
| **19_0912** | *Mormopterus francoismoutoui* | Transformateur EDF Gillot | NA | 0 | NA |
| **19_0913** | *Mormopterus francoismoutoui* | Transformateur EDF Gillot | NA | 0 | NA |
| **19_0914** | *Mormopterus francoismoutoui* | Transformateur EDF Gillot | NA | 0 | NA |
| **19_0915** | *Mormopterus francoismoutoui* | Transformateur EDF Gillot | NA | 0 | NA |
| **19_0916** | *Mormopterus francoismoutoui* | Transformateur EDF Gillot | NA | 0 | NA |
| **19_0917** | *Mormopterus francoismoutoui* | Transformateur EDF Gillot | NA | 0 | NA |
| **19_0918** | *Mormopterus francoismoutoui* | Transformateur EDF Gillot | NA | 0 | NA |
| **19_0919** | *Mormopterus francoismoutoui* | Transformateur EDF Gillot | NA | 0 | NA |
| **19_0920** | *Mormopterus francoismoutoui* | Transformateur EDF Gillot | NA | 0 | NA |
| **19_0921** | *Mormopterus francoismoutoui* | Transformateur EDF Gillot | NA | 0 | NA |
| **19_0922** | *Mormopterus francoismoutoui* | Transformateur EDF Gillot | NA | 0 | NA |
| **19_0923** | *Mormopterus francoismoutoui* | Transformateur EDF Gillot | NA | 0 | NA |
| **19_0924** | *Mormopterus francoismoutoui* | Transformateur EDF Gillot | NA | 0 | NA |
| **19_0925** | *Mormopterus francoismoutoui* | Transformateur EDF Gillot | NA | 0 | NA |
| **19_0926** | *Mormopterus francoismoutoui* | Transformateur EDF Gillot | NA | 0 | NA |
| **19_0927** | *Mormopterus francoismoutoui* | Transformateur EDF Gillot | NA | 0 | NA |
| **19_0928** | *Mormopterus francoismoutoui* | Transformateur EDF Gillot | NA | 0 | NA |
| **19_0929** | *Mormopterus francoismoutoui* | Transformateur EDF Gillot | NA | 0 | NA |
| **19_0930** | *Mormopterus francoismoutoui* | Transformateur EDF Gillot | NA | 0 | NA |
| **19_0931** | *Mormopterus francoismoutoui* | Transformateur EDF Gillot | NA | 0 | NA |
| **19_0932** | *Mormopterus francoismoutoui* | Transformateur EDF Gillot | NA | 0 | NA |
| **19_0933** | *Mormopterus francoismoutoui* | Transformateur EDF Gillot | NA | 0 | NA |
| **19_0934** | *Mormopterus francoismoutoui* | Transformateur EDF Gillot | NA | 0 | NA |
| **19_0935** | *Mormopterus francoismoutoui* | Transformateur EDF Gillot | NA | 0 | NA |
| **19_0936** | *Mormopterus francoismoutoui* | Transformateur EDF Gillot | NA | 0 | NA |
| **19_0937** | *Mormopterus francoismoutoui* | Transformateur EDF Gillot | NA | 0 | NA |
| **19_0938** | *Mormopterus francoismoutoui* | Transformateur EDF Gillot | NA | 0 | NA |
| **19_0939** | *Mormopterus francoismoutoui* | Transformateur EDF Gillot | NA | 0 | NA |
| **19_0940** | *Mormopterus francoismoutoui* | Transformateur EDF Gillot | NA | 0 | NA |
| **19_0941** | *Mormopterus francoismoutoui* | Transformateur EDF Gillot | NA | 0 | NA |
| **19_0942** | *Mormopterus francoismoutoui* | Transformateur EDF Gillot | NA | 1 | OR752522 |
| **19_0943** | *Mormopterus francoismoutoui* | Transformateur EDF Gillot | NA | 0 | NA |
| **19_0944** | *Mormopterus francoismoutoui* | Transformateur EDF Gillot | NA | 0 | NA |
| **19_0945** | *Mormopterus francoismoutoui* | Transformateur EDF Gillot | NA | 0 | NA |
| **19_0946** | *Mormopterus francoismoutoui* | Transformateur EDF Gillot | NA | 0 | NA |
| **19_0947** | *Mormopterus francoismoutoui* | Transformateur EDF Gillot | NA | 0 | NA |
| **19_0948** | *Mormopterus francoismoutoui* | Transformateur EDF Gillot | NA | 0 | NA |
| **19_0949** | *Mormopterus francoismoutoui* | Transformateur EDF Gillot | NA | 0 | NA |
| **19_0950** | *Mormopterus francoismoutoui* | Transformateur EDF Gillot | NA | 0 | NA |
| **19_0951** | *Mormopterus francoismoutoui* | Transformateur EDF Gillot | NA | 0 | NA |
| **19_0952** | *Mormopterus francoismoutoui* | Transformateur EDF Gillot | NA | 0 | NA |
| **19_0953** | *Mormopterus francoismoutoui* | Transformateur EDF Gillot | NA | 0 | NA |
| **19_0954** | *Mormopterus francoismoutoui* | Transformateur EDF Gillot | NA | 0 | NA |
| **19_1201** | *Mormopterus francoismoutoui* | Transformateur EDF Gillot | NA | 0 | NA |
| **19_1202** | *Mormopterus francoismoutoui* | Transformateur EDF Gillot | NA | 0 | NA |
| **19_1203** | *Mormopterus francoismoutoui* | Transformateur EDF Gillot | NA | 0 | NA |
| **19_1204** | *Mormopterus francoismoutoui* | Transformateur EDF Gillot | NA | 0 | NA |
| **19_1205** | *Mormopterus francoismoutoui* | Transformateur EDF Gillot | NA | 0 | NA |
| **19_1206** | *Mormopterus francoismoutoui* | Transformateur EDF Gillot | NA | 0 | NA |
| **19_1207** | *Mormopterus francoismoutoui* | Transformateur EDF Gillot | NA | 0 | NA |
| **19_1208** | *Mormopterus francoismoutoui* | Transformateur EDF Gillot | NA | 0 | NA |
| **19_1209** | *Mormopterus francoismoutoui* | Transformateur EDF Gillot | NA | 0 | NA |
| **19_1210** | *Mormopterus francoismoutoui* | Transformateur EDF Gillot | NA | 0 | NA |
| **19_1211** | *Mormopterus francoismoutoui* | Transformateur EDF Gillot | NA | 0 | NA |
| **19_1212** | *Mormopterus francoismoutoui* | Transformateur EDF Gillot | NA | 0 | NA |
| **19_1213** | *Mormopterus francoismoutoui* | Transformateur EDF Gillot | NA | 0 | NA |

| | | | | | |
|---|---|---|---|---|---|
| **19_1214** | *Mormopterus francoismoutoui* | Transformateur EDF Gillot | NA | 0 | NA |
| **19_1215** | *Mormopterus francoismoutoui* | Transformateur EDF Gillot | NA | 0 | NA |
| **19_1216** | *Mormopterus francoismoutoui* | Transformateur EDF Gillot | NA | 0 | NA |
| **19_1217** | *Mormopterus francoismoutoui* | Transformateur EDF Gillot | NA | 0 | NA |
| **19_1218** | *Mormopterus francoismoutoui* | Transformateur EDF Gillot | NA | 0 | NA |
| **19_1219** | *Mormopterus francoismoutoui* | Transformateur EDF Gillot | NA | 0 | NA |
| **19_1220** | *Mormopterus francoismoutoui* | Transformateur EDF Gillot | NA | 0 | NA |
| **19_1301** | *Mormopterus francoismoutoui* | Transformateur EDF Gillot | NA | 0 | NA |
| **19_1302** | *Mormopterus francoismoutoui* | Transformateur EDF Gillot | NA | 0 | NA |
| **19_1303** | *Mormopterus francoismoutoui* | Transformateur EDF Gillot | NA | 0 | NA |
| **19_1304** | *Mormopterus francoismoutoui* | Transformateur EDF Gillot | NA | 0 | NA |
| **19_1305** | *Mormopterus francoismoutoui* | Transformateur EDF Gillot | NA | 0 | NA |
| **19_1306** | *Mormopterus francoismoutoui* | Transformateur EDF Gillot | NA | 0 | NA |
| **19_1307** | *Mormopterus francoismoutoui* | Transformateur EDF Gillot | NA | 0 | NA |
| **19_1308** | *Mormopterus francoismoutoui* | Transformateur EDF Gillot | NA | 0 | NA |
| **19_1309** | *Mormopterus francoismoutoui* | Transformateur EDF Gillot | NA | 0 | NA |
| **19_1310** | *Mormopterus francoismoutoui* | Transformateur EDF Gillot | NA | 0 | NA |
| **19_1311** | *Mormopterus francoismoutoui* | Transformateur EDF Gillot | NA | 0 | NA |
| **19_1312** | *Mormopterus francoismoutoui* | Transformateur EDF Gillot | NA | 0 | NA |
| **19_1313** | *Mormopterus francoismoutoui* | Transformateur EDF Gillot | NA | 0 | NA |
| **19_1314** | *Mormopterus francoismoutoui* | Transformateur EDF Gillot | NA | 0 | NA |
| **19_1315** | *Mormopterus francoismoutoui* | Transformateur EDF Gillot | NA | 0 | NA |
| **19_1316** | *Mormopterus francoismoutoui* | Transformateur EDF Gillot | NA | 0 | NA |
| **19_1317** | *Mormopterus francoismoutoui* | Transformateur EDF Gillot | NA | 0 | NA |
| **19_1318** | *Mormopterus francoismoutoui* | Transformateur EDF Gillot | NA | 0 | NA |
| **19_1319** | *Mormopterus francoismoutoui* | Transformateur EDF Gillot | NA | 0 | NA |
| **19_1320** | *Mormopterus francoismoutoui* | Transformateur EDF Gillot | NA | 0 | NA |
| **19_1321** | *Mormopterus francoismoutoui* | Transformateur EDF Gillot | NA | 0 | NA |
| **19_1322** | *Mormopterus francoismoutoui* | Transformateur EDF Gillot | NA | 0 | NA |
| **19_1323** | *Mormopterus francoismoutoui* | Transformateur EDF Gillot | NA | 0 | NA |
| **19_1324** | *Mormopterus francoismoutoui* | Transformateur EDF Gillot | NA | 0 | NA |
| **19_1325** | *Mormopterus francoismoutoui* | Transformateur EDF Gillot | NA | 0 | NA |
| **19_1326** | *Mormopterus francoismoutoui* | Transformateur EDF Gillot | NA | 0 | NA |
| **19_1327** | *Mormopterus francoismoutoui* | Transformateur EDF Gillot | NA | 0 | NA |
| **19_1328** | *Mormopterus francoismoutoui* | Transformateur EDF Gillot | NA | 0 | NA |
| **19_1329** | *Mormopterus francoismoutoui* | Transformateur EDF Gillot | NA | 0 | NA |
| **19_1330** | *Mormopterus francoismoutoui* | Transformateur EDF Gillot | NA | 0 | NA |
| **19_1331** | *Mormopterus francoismoutoui* | Transformateur EDF Gillot | NA | 0 | NA |
| **19_1332** | *Mormopterus francoismoutoui* | Transformateur EDF Gillot | NA | 0 | NA |
| **19_1333** | *Mormopterus francoismoutoui* | Transformateur EDF Gillot | NA | 0 | NA |
| **19_1334** | *Mormopterus francoismoutoui* | Transformateur EDF Gillot | NA | 0 | NA |
| **19_1335** | *Mormopterus francoismoutoui* | Transformateur EDF Gillot | NA | 0 | NA |
| **19_1336** | *Mormopterus francoismoutoui* | Transformateur EDF Gillot | NA | 0 | NA |
| **19_1337** | *Mormopterus francoismoutoui* | Transformateur EDF Gillot | NA | 0 | NA |
| **19_1338** | *Mormopterus francoismoutoui* | Transformateur EDF Gillot | NA | 0 | NA |
| **19_1339** | *Mormopterus francoismoutoui* | Transformateur EDF Gillot | NA | 0 | NA |
| **19_1340** | *Mormopterus francoismoutoui* | Transformateur EDF Gillot | NA | 0 | NA |
| **19_1341** | *Mormopterus francoismoutoui* | Transformateur EDF Gillot | NA | 0 | NA |
| **19_1342** | *Mormopterus francoismoutoui* | Transformateur EDF Gillot | NA | 0 | NA |
| **19_1343** | *Mormopterus francoismoutoui* | Transformateur EDF Gillot | NA | 0 | NA |
| **19_1344** | *Mormopterus francoismoutoui* | Transformateur EDF Gillot | NA | 0 | NA |
| **19_1345** | *Mormopterus francoismoutoui* | Transformateur EDF Gillot | NA | 0 | NA |
| **19_1346** | *Mormopterus francoismoutoui* | Transformateur EDF Gillot | NA | 0 | NA |
| **19_1347** | *Mormopterus francoismoutoui* | Transformateur EDF Gillot | NA | 0 | NA |
| **19_1348** | *Mormopterus francoismoutoui* | Transformateur EDF Gillot | NA | 0 | NA |
| **19_1349** | *Mormopterus francoismoutoui* | Transformateur EDF Gillot | NA | 0 | NA |
| **19_1350** | *Mormopterus francoismoutoui* | Transformateur EDF Gillot | NA | 0 | NA |
| **19_1351** | *Mormopterus francoismoutoui* | Transformateur EDF Gillot | NA | 0 | NA |
| **19_1352** | *Mormopterus francoismoutoui* | Transformateur EDF Gillot | NA | 0 | NA |
| **19_1353** | *Mormopterus francoismoutoui* | Transformateur EDF Gillot | NA | 0 | NA |
| **19_1354** | *Mormopterus francoismoutoui* | Transformateur EDF Gillot | NA | 0 | NA |
| **19_1355** | *Mormopterus francoismoutoui* | Transformateur EDF Gillot | NA | 0 | NA |
| **19_1356** | *Mormopterus francoismoutoui* | Transformateur EDF Gillot | NA | 0 | NA |
| **19_1357** | *Mormopterus francoismoutoui* | Transformateur EDF Gillot | NA | 0 | NA |
| **19_1358** | *Mormopterus francoismoutoui* | Transformateur EDF Gillot | NA | 0 | NA |
| **19_1359** | *Mormopterus francoismoutoui* | Transformateur EDF Gillot | NA | 0 | NA |
| **19_1360** | *Mormopterus francoismoutoui* | Transformateur EDF Gillot | NA | 0 | NA |
| **19_1361** | *Mormopterus francoismoutoui* | Transformateur EDF Gillot | NA | 0 | NA |
| **19_1362** | *Mormopterus francoismoutoui* | Transformateur EDF Gillot | NA | 0 | NA |
| **19_1363** | *Mormopterus francoismoutoui* | Transformateur EDF Gillot | NA | 0 | NA |
| **19_1364** | *Mormopterus francoismoutoui* | Transformateur EDF Gillot | NA | 0 | NA |
| **19_1365** | *Mormopterus francoismoutoui* | Transformateur EDF Gillot | NA | 0 | NA |

| | | | | | |
|---|---|---|---|---|---|
| **19_1366** | *Mormopterus francoismoutoui* | Transformateur EDF Gillot | NA | 0 | NA |
| **19_1367** | *Mormopterus francoismoutoui* | Transformateur EDF Gillot | NA | 0 | NA |
| **19_1368** | *Mormopterus francoismoutoui* | Transformateur EDF Gillot | NA | 0 | NA |
| **19_1369** | *Mormopterus francoismoutoui* | Transformateur EDF Gillot | NA | 0 | NA |
| **19_1370** | *Mormopterus francoismoutoui* | Transformateur EDF Gillot | NA | 0 | NA |
| **19_1371** | *Mormopterus francoismoutoui* | Transformateur EDF Gillot | NA | 0 | NA |
| **19_1372** | *Mormopterus francoismoutoui* | Transformateur EDF Gillot | NA | 0 | NA |
| **19_1373** | *Mormopterus francoismoutoui* | Transformateur EDF Gillot | NA | 0 | NA |
| **19_1374** | *Mormopterus francoismoutoui* | Transformateur EDF Gillot | NA | 0 | NA |
| **19_1375** | *Mormopterus francoismoutoui* | Transformateur EDF Gillot | NA | 0 | NA |
| **19_1376** | *Mormopterus francoismoutoui* | Transformateur EDF Gillot | NA | 0 | NA |
| **19_1377** | *Mormopterus francoismoutoui* | Transformateur EDF Gillot | NA | 0 | NA |
| **19_1378** | *Mormopterus francoismoutoui* | Transformateur EDF Gillot | NA | 0 | NA |
| **19_1379** | *Mormopterus francoismoutoui* | Transformateur EDF Gillot | NA | 0 | NA |
| **19_1380** | *Mormopterus francoismoutoui* | Transformateur EDF Gillot | NA | 0 | NA |
| **19_1381** | *Mormopterus francoismoutoui* | Transformateur EDF Gillot | NA | 0 | NA |
| **19_1382** | *Mormopterus francoismoutoui* | Transformateur EDF Gillot | NA | 0 | NA |
| **19_1383** | *Mormopterus francoismoutoui* | Transformateur EDF Gillot | NA | 0 | NA |
| **19_1384** | *Mormopterus francoismoutoui* | Transformateur EDF Gillot | NA | 0 | NA |
| **19_1385** | *Mormopterus francoismoutoui* | Transformateur EDF Gillot | NA | 0 | NA |
| **19_1386** | *Mormopterus francoismoutoui* | Transformateur EDF Gillot | NA | 0 | NA |
| **19_1387** | *Mormopterus francoismoutoui* | Transformateur EDF Gillot | NA | 0 | NA |
| **19_1388** | *Mormopterus francoismoutoui* | Transformateur EDF Gillot | NA | 0 | NA |
| **19_1389** | *Mormopterus francoismoutoui* | Transformateur EDF Gillot | NA | 0 | NA |
| **19_1390** | *Mormopterus francoismoutoui* | Transformateur EDF Gillot | NA | 0 | NA |
| **19_1391** | *Mormopterus francoismoutoui* | Trois bassins | NA | 0 | NA |
| **19_1392** | *Mormopterus francoismoutoui* | Trois bassins | NA | 0 | NA |
| **19_1393** | *Mormopterus francoismoutoui* | Trois bassins | NA | 0 | NA |
| **19_1394** | *Mormopterus francoismoutoui* | Trois bassins | NA | 0 | NA |
| **19_1395** | *Mormopterus francoismoutoui* | Trois bassins | NA | 0 | NA |
| **19_1396** | *Mormopterus francoismoutoui* | Trois bassins | NA | 0 | NA |
| **19_1397** | *Mormopterus francoismoutoui* | Trois bassins | NA | 0 | NA |
| **19_1398** | *Mormopterus francoismoutoui* | Trois bassins | NA | 0 | NA |
| **19_1399** | *Mormopterus francoismoutoui* | Trois bassins | NA | 1 | OR752523 |
| **19_1400** | *Mormopterus francoismoutoui* | Trois bassins | NA | 0 | NA |
| **19_1401** | *Mormopterus francoismoutoui* | Trois bassins | NA | 0 | NA |
| **19_1402** | *Mormopterus francoismoutoui* | Trois bassins | NA | 0 | NA |
| **19_1403** | *Mormopterus francoismoutoui* | Trois bassins | NA | 0 | NA |
| **19_1404** | *Mormopterus francoismoutoui* | Trois bassins | NA | 0 | NA |
| **19_1405** | *Mormopterus francoismoutoui* | Trois bassins | NA | 0 | NA |
| **19_1406** | *Mormopterus francoismoutoui* | Trois bassins | NA | 0 | NA |
| **19_1407** | *Mormopterus francoismoutoui* | Trois bassins | NA | 0 | NA |
| **19_1408** | *Mormopterus francoismoutoui* | Trois bassins | NA | 0 | NA |
| **19_1409** | *Mormopterus francoismoutoui* | Trois bassins | NA | 0 | NA |
| **19_1410** | *Mormopterus francoismoutoui* | Trois bassins | NA | 0 | NA |
| **19_1411** | *Mormopterus francoismoutoui* | Trois bassins | NA | 0 | NA |
| **19_1412** | *Mormopterus francoismoutoui* | Trois bassins | NA | 0 | NA |
| **19_1413** | *Mormopterus francoismoutoui* | Trois bassins | NA | 0 | NA |
| **19_1414** | *Mormopterus francoismoutoui* | Trois bassins | NA | 0 | NA |
| **19_1415** | *Mormopterus francoismoutoui* | Trois bassins | NA | 0 | NA |
| **19_1416** | *Mormopterus francoismoutoui* | Trois bassins | NA | 0 | NA |
| **19_1417** | *Mormopterus francoismoutoui* | Trois bassins | NA | 0 | NA |
| **19_1418** | *Mormopterus francoismoutoui* | Trois bassins | NA | 0 | NA |
| **19_1419** | *Mormopterus francoismoutoui* | Trois bassins | NA | 0 | NA |
| **19_1420** | *Mormopterus francoismoutoui* | Trois bassins | NA | 0 | NA |
| **19_1421** | *Mormopterus francoismoutoui* | Trois bassins | NA | 0 | NA |
| **19_1422** | *Mormopterus francoismoutoui* | Trois bassins | NA | 0 | NA |
| **19_1423** | *Mormopterus francoismoutoui* | Trois bassins | NA | 0 | NA |
| **19_1424** | *Mormopterus francoismoutoui* | Trois bassins | NA | 0 | NA |
| **19_1425** | *Mormopterus francoismoutoui* | Trois bassins | NA | 0 | NA |
| **19_1426** | *Mormopterus francoismoutoui* | Trois bassins | NA | 0 | NA |
| **19_1427** | *Mormopterus francoismoutoui* | Trois bassins | NA | 0 | NA |
| **19_1428** | *Mormopterus francoismoutoui* | Trois bassins | NA | 0 | NA |
| **19_1429** | *Mormopterus francoismoutoui* | Trois bassins | NA | 0 | NA |
| **19_1430** | *Mormopterus francoismoutoui* | Trois bassins | NA | 0 | NA |
| **19_1431** | *Mormopterus francoismoutoui* | Trois bassins | NA | 0 | NA |
| **19_1432** | *Mormopterus francoismoutoui* | Trois bassins | NA | 0 | NA |
| **19_1433** | *Mormopterus francoismoutoui* | Trois bassins | NA | 0 | NA |
| **19_1434** | *Mormopterus francoismoutoui* | Trois bassins | NA | 0 | NA |
| **19_1435** | *Mormopterus francoismoutoui* | Trois bassins | NA | 0 | NA |
| **19_1436** | *Mormopterus francoismoutoui* | Trois bassins | NA | 0 | NA |
| **19_1437** | *Mormopterus francoismoutoui* | Trois bassins | NA | 0 | NA |

| | | | | | |
|---|---|---|---|---|---|
| **19_1438** | *Mormopterus francoismoutoui* | Trois bassins | NA | 0 | NA |
| **19_1439** | *Mormopterus francoismoutoui* | Trois bassins | NA | 0 | NA |
| **19_1440** | *Mormopterus francoismoutoui* | Trois bassins | NA | 0 | NA |
| **19_1441** | *Mormopterus francoismoutoui* | Transformateur EDF Gillot | NA | 0 | NA |
| **19_1442** | *Mormopterus francoismoutoui* | Transformateur EDF Gillot | NA | 0 | NA |
| **19_1443** | *Mormopterus francoismoutoui* | Transformateur EDF Gillot | NA | 0 | NA |
| **19_1444** | *Mormopterus francoismoutoui* | Transformateur EDF Gillot | NA | 1 | OR752524 |
| **19_1445** | *Mormopterus francoismoutoui* | Transformateur EDF Gillot | NA | 0 | NA |
| **19_1446** | *Mormopterus francoismoutoui* | Transformateur EDF Gillot | NA | 0 | NA |
| **19_1447** | *Mormopterus francoismoutoui* | Transformateur EDF Gillot | NA | 0 | NA |
| **19_1448** | *Mormopterus francoismoutoui* | Transformateur EDF Gillot | NA | 0 | NA |
| **19_1449** | *Mormopterus francoismoutoui* | Transformateur EDF Gillot | NA | 0 | NA |
| **19_1450** | *Mormopterus francoismoutoui* | Transformateur EDF Gillot | NA | 0 | NA |
| **19_1451** | *Mormopterus francoismoutoui* | Transformateur EDF Gillot | NA | 0 | NA |
| **19_1452** | *Mormopterus francoismoutoui* | Transformateur EDF Gillot | NA | 0 | NA |
| **19_1453** | *Mormopterus francoismoutoui* | Transformateur EDF Gillot | NA | 0 | NA |
| **19_1454** | *Mormopterus francoismoutoui* | Transformateur EDF Gillot | NA | 0 | NA |
| **19_1455** | *Mormopterus francoismoutoui* | Transformateur EDF Gillot | NA | 0 | NA |
| **19_1456** | *Mormopterus francoismoutoui* | Transformateur EDF Gillot | NA | 1 | OR752525 |
| **19_1457** | *Mormopterus francoismoutoui* | Transformateur EDF Gillot | NA | 0 | NA |
| **19_1458** | *Mormopterus francoismoutoui* | Transformateur EDF Gillot | NA | 0 | NA |
| **19_1459** | *Mormopterus francoismoutoui* | Transformateur EDF Gillot | NA | 0 | NA |
| **19_1460** | *Mormopterus francoismoutoui* | Transformateur EDF Gillot | NA | 0 | NA |
| **19_1461** | *Mormopterus francoismoutoui* | Transformateur EDF Gillot | NA | 0 | NA |
| **19_1462** | *Mormopterus francoismoutoui* | Transformateur EDF Gillot | NA | 0 | NA |
| **19_1463** | *Mormopterus francoismoutoui* | Transformateur EDF Gillot | NA | 1 | OR752526 |
| **19_1464** | *Mormopterus francoismoutoui* | Transformateur EDF Gillot | NA | 0 | NA |
| **19_1465** | *Mormopterus francoismoutoui* | Transformateur EDF Gillot | NA | 0 | NA |
| **19_1466** | *Mormopterus francoismoutoui* | Transformateur EDF Gillot | NA | 0 | NA |
| **19_1467** | *Mormopterus francoismoutoui* | Transformateur EDF Gillot | NA | 0 | NA |
| **19_1468** | *Mormopterus francoismoutoui* | Transformateur EDF Gillot | NA | 0 | NA |
| **19_1469** | *Mormopterus francoismoutoui* | Transformateur EDF Gillot | NA | 0 | NA |
| **19_1470** | *Mormopterus francoismoutoui* | Transformateur EDF Gillot | NA | 0 | NA |
| **19_1471** | *Mormopterus francoismoutoui* | Trois bassins | NA | 0 | NA |
| **19_1472** | *Mormopterus francoismoutoui* | Trois bassins | NA | 0 | NA |
| **19_1473** | *Mormopterus francoismoutoui* | Trois bassins | NA | 0 | NA |
| **19_1474** | *Mormopterus francoismoutoui* | Trois bassins | NA | 0 | NA |
| **19_1475** | *Mormopterus francoismoutoui* | Trois bassins | NA | 0 | NA |
| **19_1476** | *Mormopterus francoismoutoui* | Trois bassins | NA | 0 | NA |
| **19_1477** | *Mormopterus francoismoutoui* | Trois bassins | NA | 0 | NA |
| **19_1478** | *Mormopterus francoismoutoui* | Trois bassins | NA | 0 | NA |
| **19_1479** | *Mormopterus francoismoutoui* | Trois bassins | NA | 0 | NA |
| **19_1480** | *Mormopterus francoismoutoui* | Trois bassins | NA | 0 | NA |
| **19_1481** | *Mormopterus francoismoutoui* | Trois bassins | NA | 0 | NA |
| **19_1482** | *Mormopterus francoismoutoui* | Trois bassins | NA | 0 | NA |
| **19_1483** | *Mormopterus francoismoutoui* | Trois bassins | NA | 0 | NA |
| **19_1484** | *Mormopterus francoismoutoui* | Trois bassins | NA | 0 | NA |
| **19_1485** | *Mormopterus francoismoutoui* | Trois bassins | NA | 0 | NA |
| **19_1486** | *Mormopterus francoismoutoui* | Trois bassins | NA | 0 | NA |
| **19_1487** | *Mormopterus francoismoutoui* | Trois bassins | NA | 0 | NA |
| **19_1488** | *Mormopterus francoismoutoui* | Trois bassins | NA | 0 | NA |
| **19_1489** | *Mormopterus francoismoutoui* | Trois bassins | NA | 0 | NA |
| **19_1490** | *Mormopterus francoismoutoui* | Trois bassins | NA | 0 | NA |
| **19_1491** | *Mormopterus francoismoutoui* | Trois bassins | NA | 0 | NA |
| **19_1492** | *Mormopterus francoismoutoui* | Trois bassins | NA | 0 | NA |
| **19_1493** | *Mormopterus francoismoutoui* | Trois bassins | NA | 1 | OR752527 |
| **19_1494** | *Mormopterus francoismoutoui* | Trois bassins | NA | 0 | NA |
| **19_1495** | *Mormopterus francoismoutoui* | Trois bassins | NA | 0 | NA |
| **19_1496** | *Mormopterus francoismoutoui* | Trois bassins | NA | 0 | NA |
| **19_1497** | *Mormopterus francoismoutoui* | Trois bassins | NA | 0 | NA |
| **19_1498** | *Mormopterus francoismoutoui* | Trois bassins | NA | 0 | NA |
| **19_1499** | *Mormopterus francoismoutoui* | Trois bassins | NA | 0 | NA |
| **19_1500** | *Mormopterus francoismoutoui* | Trois bassins | NA | 0 | NA |
| **19_1501** | *Mormopterus francoismoutoui* | Trois bassins | NA | 0 | NA |
| **19_1502** | *Mormopterus francoismoutoui* | Trois bassins | NA | 0 | NA |
| **19_1503** | *Mormopterus francoismoutoui* | Trois bassins | NA | 0 | NA |
| **19_1504** | *Mormopterus francoismoutoui* | Trois bassins | NA | 0 | NA |
| **19_1505** | *Mormopterus francoismoutoui* | Trois bassins | NA | 0 | NA |
| **19_1506** | *Mormopterus francoismoutoui* | Trois bassins | NA | 1 | OR752528 |
| **19_1507** | *Mormopterus francoismoutoui* | Trois bassins | NA | 0 | NA |
| **19_1508** | *Mormopterus francoismoutoui* | Trois bassins | NA | 0 | NA |
| **19_1509** | *Mormopterus francoismoutoui* | Trois bassins | NA | 0 | NA |

| | | | | | |
|---|---|---|---|---|---|
| **19_1510** | *Mormopterus francoismoutoui* | Trois bassins | NA | 0 | NA |
| **19_1511** | *Mormopterus francoismoutoui* | Trois bassins | NA | 0 | NA |
| **19_1512** | *Mormopterus francoismoutoui* | Trois bassins | NA | 0 | NA |
| **19_1513** | *Mormopterus francoismoutoui* | Trois bassins | NA | 0 | NA |
| **19_1514** | *Mormopterus francoismoutoui* | Trois bassins | NA | 0 | NA |
| **19_1515** | *Mormopterus francoismoutoui* | Trois bassins | NA | 0 | NA |
| **19_1516** | *Mormopterus francoismoutoui* | Trois bassins | NA | 0 | NA |
| **19_1517** | *Mormopterus francoismoutoui* | Trois bassins | NA | 0 | NA |
| **19_1518** | *Mormopterus francoismoutoui* | Trois bassins | NA | 0 | NA |
| **19_1519** | *Mormopterus francoismoutoui* | Trois bassins | NA | 0 | NA |
| **19_1520** | *Mormopterus francoismoutoui* | Trois bassins | NA | 0 | NA |
| **19_1521** | *Mormopterus francoismoutoui* | Transformateur EDF Gillot | NA | 0 | NA |
| **19_1522** | *Mormopterus francoismoutoui* | Transformateur EDF Gillot | NA | 0 | NA |
| **19_1523** | *Mormopterus francoismoutoui* | Transformateur EDF Gillot | NA | 0 | NA |
| **19_1524** | *Mormopterus francoismoutoui* | Transformateur EDF Gillot | NA | 0 | NA |
| **19_1525** | *Mormopterus francoismoutoui* | Transformateur EDF Gillot | NA | 0 | NA |
| **19_1526** | *Mormopterus francoismoutoui* | Transformateur EDF Gillot | NA | 0 | NA |
| **19_1527** | *Mormopterus francoismoutoui* | Transformateur EDF Gillot | NA | 0 | NA |
| **19_1528** | *Mormopterus francoismoutoui* | Transformateur EDF Gillot | NA | 0 | NA |
| **19_1529** | *Mormopterus francoismoutoui* | Transformateur EDF Gillot | NA | 0 | NA |
| **19_1530** | *Mormopterus francoismoutoui* | Transformateur EDF Gillot | NA | 0 | NA |
| **19_1531** | *Mormopterus francoismoutoui* | Transformateur EDF Gillot | NA | 0 | NA |
| **19_1532** | *Mormopterus francoismoutoui* | Transformateur EDF Gillot | NA | 0 | NA |
| **19_1533** | *Mormopterus francoismoutoui* | Transformateur EDF Gillot | NA | 0 | NA |
| **19_1534** | *Mormopterus francoismoutoui* | Transformateur EDF Gillot | NA | 0 | NA |
| **19_1535** | *Mormopterus francoismoutoui* | Transformateur EDF Gillot | NA | 0 | NA |
| **19_1536** | *Mormopterus francoismoutoui* | Transformateur EDF Gillot | NA | 0 | NA |
| **19_1537** | *Mormopterus francoismoutoui* | Transformateur EDF Gillot | NA | 0 | NA |
| **19_1538** | *Mormopterus francoismoutoui* | Transformateur EDF Gillot | NA | 0 | NA |
| **19_1539** | *Mormopterus francoismoutoui* | Transformateur EDF Gillot | NA | 0 | NA |
| **19_1540** | *Mormopterus francoismoutoui* | Transformateur EDF Gillot | NA | 0 | NA |
| **19_1541** | *Mormopterus francoismoutoui* | Transformateur EDF Gillot | NA | 0 | NA |
| **19_1542** | *Mormopterus francoismoutoui* | Transformateur EDF Gillot | NA | 0 | NA |
| **19_1543** | *Mormopterus francoismoutoui* | Transformateur EDF Gillot | NA | 0 | NA |
| **19_1544** | *Mormopterus francoismoutoui* | Transformateur EDF Gillot | NA | 0 | NA |
| **19_1545** | *Mormopterus francoismoutoui* | Transformateur EDF Gillot | NA | 0 | NA |
| **19_1546** | *Mormopterus francoismoutoui* | Transformateur EDF Gillot | NA | 0 | NA |
| **19_1547** | *Mormopterus francoismoutoui* | Transformateur EDF Gillot | NA | 0 | NA |
| **19_1548** | *Mormopterus francoismoutoui* | Transformateur EDF Gillot | NA | 0 | NA |
| **19_1549** | *Mormopterus francoismoutoui* | Transformateur EDF Gillot | NA | 0 | NA |
| **19_1550** | *Mormopterus francoismoutoui* | Transformateur EDF Gillot | NA | 0 | NA |
| **19_1551** | *Mormopterus francoismoutoui* | Trois bassins | NA | 0 | NA |
| **19_1552** | *Mormopterus francoismoutoui* | Trois bassins | NA | 0 | NA |
| **19_1553** | *Mormopterus francoismoutoui* | Trois bassins | NA | 0 | NA |
| **19_1554** | *Mormopterus francoismoutoui* | Trois bassins | NA | 0 | NA |
| **19_1555** | *Mormopterus francoismoutoui* | Trois bassins | NA | 0 | NA |
| **19_1556** | *Mormopterus francoismoutoui* | Trois bassins | NA | 0 | NA |
| **19_1557** | *Mormopterus francoismoutoui* | Trois bassins | NA | 0 | NA |
| **19_1558** | *Mormopterus francoismoutoui* | Trois bassins | NA | 0 | NA |
| **19_1559** | *Mormopterus francoismoutoui* | Trois bassins | NA | 0 | NA |
| **19_1560** | *Mormopterus francoismoutoui* | Trois bassins | NA | 0 | NA |
| **19_1561** | *Mormopterus francoismoutoui* | Trois bassins | NA | 0 | NA |
| **19_1562** | *Mormopterus francoismoutoui* | Trois bassins | NA | 0 | NA |
| **19_1563** | *Mormopterus francoismoutoui* | Trois bassins | NA | 0 | NA |
| **19_1564** | *Mormopterus francoismoutoui* | Trois bassins | NA | 0 | NA |
| **19_1565** | *Mormopterus francoismoutoui* | Trois bassins | NA | 0 | NA |
| **19_1566** | *Mormopterus francoismoutoui* | Trois bassins | NA | 0 | NA |
| **19_1567** | *Mormopterus francoismoutoui* | Trois bassins | NA | 0 | NA |
| **19_1568** | *Mormopterus francoismoutoui* | Trois bassins | NA | 0 | NA |
| **19_1569** | *Mormopterus francoismoutoui* | Trois bassins | NA | 0 | NA |
| **19_1570** | *Mormopterus francoismoutoui* | Trois bassins | NA | 0 | NA |
| **19_1571** | *Mormopterus francoismoutoui* | Trois bassins | NA | 0 | NA |
| **19_1572** | *Mormopterus francoismoutoui* | Trois bassins | NA | 0 | NA |
| **19_1573** | *Mormopterus francoismoutoui* | Trois bassins | NA | 0 | NA |
| **19_1574** | *Mormopterus francoismoutoui* | Trois bassins | NA | 0 | NA |
| **19_1575** | *Mormopterus francoismoutoui* | Trois bassins | NA | 0 | NA |
| **19_1576** | *Mormopterus francoismoutoui* | Trois bassins | NA | 0 | NA |
| **19_1577** | *Mormopterus francoismoutoui* | Trois bassins | NA | 0 | NA |
| **19_1578** | *Mormopterus francoismoutoui* | Trois bassins | NA | 0 | NA |
| **19_1579** | *Mormopterus francoismoutoui* | Trois bassins | NA | 0 | NA |
| **19_1580** | *Mormopterus francoismoutoui* | Trois bassins | NA | 0 | NA |
| **19_1581** | *Mormopterus francoismoutoui* | Trois bassins | NA | 0 | NA |

| | | | | | |
|---|---|---|---|---|---|
| **19_1582** | *Mormopterus francoismoutoui* | Trois bassins | NA | 0 | NA |
| **19_1583** | *Mormopterus francoismoutoui* | Trois bassins | NA | 0 | NA |
| **19_1584** | *Mormopterus francoismoutoui* | Trois bassins | NA | 0 | NA |
| **19_1585** | *Mormopterus francoismoutoui* | Trois bassins | NA | 0 | NA |
| **19_1586** | *Mormopterus francoismoutoui* | Trois bassins | NA | 0 | NA |
| **19_1587** | *Mormopterus francoismoutoui* | Trois bassins | NA | 0 | NA |
| **19_1588** | *Mormopterus francoismoutoui* | Trois bassins | NA | 0 | NA |
| **19_1589** | *Mormopterus francoismoutoui* | Trois bassins | NA | 0 | NA |
| **19_1590** | *Mormopterus francoismoutoui* | Trois bassins | NA | 0 | NA |
| **19_1591** | *Mormopterus francoismoutoui* | Trois bassins | NA | 0 | NA |
| **19_1592** | *Mormopterus francoismoutoui* | Trois bassins | NA | 0 | NA |
| **19_1593** | *Mormopterus francoismoutoui* | Trois bassins | NA | 0 | NA |
| **19_1594** | *Mormopterus francoismoutoui* | Trois bassins | NA | 0 | NA |
| **19_1595** | *Mormopterus francoismoutoui* | Trois bassins | NA | 0 | NA |
| **19_1596** | *Mormopterus francoismoutoui* | Trois bassins | NA | 0 | NA |
| **19_1597** | *Mormopterus francoismoutoui* | Trois bassins | NA | 0 | NA |
| **19_1598** | *Mormopterus francoismoutoui* | Trois bassins | NA | 0 | NA |
| **19_1599** | *Mormopterus francoismoutoui* | Trois bassins | NA | 0 | NA |
| **19_1600** | *Mormopterus francoismoutoui* | Trois bassins | NA | 0 | NA |
| **19_1601** | *Mormopterus francoismoutoui* | Transformateur EDF Gillot | NA | 0 | NA |
| **19_1602** | *Mormopterus francoismoutoui* | Transformateur EDF Gillot | NA | 0 | NA |
| **19_1603** | *Mormopterus francoismoutoui* | Transformateur EDF Gillot | NA | 0 | NA |
| **19_1604** | *Mormopterus francoismoutoui* | Transformateur EDF Gillot | NA | 0 | NA |
| **19_1605** | *Mormopterus francoismoutoui* | Transformateur EDF Gillot | NA | 0 | NA |
| **19_1606** | *Mormopterus francoismoutoui* | Transformateur EDF Gillot | NA | 0 | NA |
| **19_1607** | *Mormopterus francoismoutoui* | Transformateur EDF Gillot | NA | 0 | NA |
| **19_1608** | *Mormopterus francoismoutoui* | Transformateur EDF Gillot | NA | 0 | NA |
| **19_1609** | *Mormopterus francoismoutoui* | Transformateur EDF Gillot | NA | 0 | NA |
| **19_1610** | *Mormopterus francoismoutoui* | Transformateur EDF Gillot | NA | 0 | NA |
| **19_1611** | *Mormopterus francoismoutoui* | Transformateur EDF Gillot | NA | 0 | NA |
| **19_1612** | *Mormopterus francoismoutoui* | Transformateur EDF Gillot | NA | 0 | NA |
| **19_1613** | *Mormopterus francoismoutoui* | Transformateur EDF Gillot | NA | 0 | NA |
| **19_1614** | *Mormopterus francoismoutoui* | Transformateur EDF Gillot | NA | 0 | NA |
| **19_1615** | *Mormopterus francoismoutoui* | Transformateur EDF Gillot | NA | 0 | NA |
| **19_1616** | *Mormopterus francoismoutoui* | Transformateur EDF Gillot | NA | 0 | NA |
| **19_1617** | *Mormopterus francoismoutoui* | Transformateur EDF Gillot | NA | 0 | NA |
| **19_1618** | *Mormopterus francoismoutoui* | Transformateur EDF Gillot | NA | 0 | NA |
| **19_1619** | *Mormopterus francoismoutoui* | Transformateur EDF Gillot | NA | 0 | NA |
| **19_1620** | *Mormopterus francoismoutoui* | Transformateur EDF Gillot | NA | 0 | NA |
| **19_1621** | *Mormopterus francoismoutoui* | Transformateur EDF Gillot | NA | 0 | NA |
| **19_1622** | *Mormopterus francoismoutoui* | Transformateur EDF Gillot | NA | 0 | NA |
| **19_1623** | *Mormopterus francoismoutoui* | Transformateur EDF Gillot | NA | 0 | NA |
| **19_1624** | *Mormopterus francoismoutoui* | Transformateur EDF Gillot | NA | 0 | NA |
| **19_1625** | *Mormopterus francoismoutoui* | Transformateur EDF Gillot | NA | 0 | NA |
| **19_1626** | *Mormopterus francoismoutoui* | Transformateur EDF Gillot | NA | 0 | NA |
| **19_1627** | *Mormopterus francoismoutoui* | Transformateur EDF Gillot | NA | 0 | NA |
| **19_1628** | *Mormopterus francoismoutoui* | Transformateur EDF Gillot | NA | 0 | NA |
| **19_1629** | *Mormopterus francoismoutoui* | Transformateur EDF Gillot | NA | 0 | NA |
| **19_1630** | *Mormopterus francoismoutoui* | Transformateur EDF Gillot | NA | 0 | NA |
| **19_1631** | *Mormopterus francoismoutoui* | Trois bassins | NA | 0 | NA |
| **19_1632** | *Mormopterus francoismoutoui* | Trois bassins | NA | 0 | NA |
| **19_1633** | *Mormopterus francoismoutoui* | Trois bassins | NA | 0 | NA |
| **19_1634** | *Mormopterus francoismoutoui* | Trois bassins | NA | 0 | NA |
| **19_1635** | *Mormopterus francoismoutoui* | Trois bassins | NA | 0 | NA |
| **19_1636** | *Mormopterus francoismoutoui* | Trois bassins | NA | 0 | NA |
| **19_1637** | *Mormopterus francoismoutoui* | Trois bassins | NA | 0 | NA |
| **19_1638** | *Mormopterus francoismoutoui* | Trois bassins | NA | 0 | NA |
| **19_1639** | *Mormopterus francoismoutoui* | Trois bassins | NA | 0 | NA |
| **19_1640** | *Mormopterus francoismoutoui* | Trois bassins | NA | 0 | NA |
| **19_1641** | *Mormopterus francoismoutoui* | Trois bassins | NA | 0 | NA |
| **19_1642** | *Mormopterus francoismoutoui* | Trois bassins | NA | 0 | NA |
| **19_1643** | *Mormopterus francoismoutoui* | Trois bassins | NA | 0 | NA |
| **19_1644** | *Mormopterus francoismoutoui* | Trois bassins | NA | 0 | NA |
| **19_1645** | *Mormopterus francoismoutoui* | Trois bassins | NA | 0 | NA |
| **19_1646** | *Mormopterus francoismoutoui* | Trois bassins | NA | 0 | NA |
| **19_1647** | *Mormopterus francoismoutoui* | Trois bassins | NA | 0 | NA |
| **19_1648** | *Mormopterus francoismoutoui* | Trois bassins | NA | 0 | NA |
| **19_1649** | *Mormopterus francoismoutoui* | Trois bassins | NA | 0 | NA |
| **19_1650** | *Mormopterus francoismoutoui* | Trois bassins | NA | 0 | NA |
| **19_1651** | *Mormopterus francoismoutoui* | Trois bassins | NA | 0 | NA |
| **19_1652** | *Mormopterus francoismoutoui* | Trois bassins | NA | 0 | NA |
| **19_1653** | *Mormopterus francoismoutoui* | Trois bassins | NA | 0 | NA |

| | | | | | |
|---|---|---|---|---|---|
| **19_1654** | *Mormopterus francoismoutoui* | Trois bassins | NA | 0 | NA |
| **19_1655** | *Mormopterus francoismoutoui* | Trois bassins | NA | 0 | NA |
| **19_1656** | *Mormopterus francoismoutoui* | Trois bassins | NA | 0 | NA |
| **19_1657** | *Mormopterus francoismoutoui* | Trois bassins | NA | 0 | NA |
| **19_1658** | *Mormopterus francoismoutoui* | Trois bassins | NA | 0 | NA |
| **19_1659** | *Mormopterus francoismoutoui* | Trois bassins | NA | 0 | NA |
| **19_1660** | *Mormopterus francoismoutoui* | Trois bassins | NA | 0 | NA |
| **19_1661** | *Mormopterus francoismoutoui* | Trois bassins | NA | 0 | NA |
| **19_1662** | *Mormopterus francoismoutoui* | Trois bassins | NA | 0 | NA |
| **19_1663** | *Mormopterus francoismoutoui* | Trois bassins | NA | 0 | NA |
| **19_1664** | *Mormopterus francoismoutoui* | Trois bassins | NA | 0 | NA |
| **19_1665** | *Mormopterus francoismoutoui* | Trois bassins | NA | 0 | NA |
| **19_1666** | *Mormopterus francoismoutoui* | Trois bassins | NA | 0 | NA |
| **19_1667** | *Mormopterus francoismoutoui* | Trois bassins | NA | 0 | NA |
| **19_1668** | *Mormopterus francoismoutoui* | Trois bassins | NA | 0 | NA |
| **19_1669** | *Mormopterus francoismoutoui* | Trois bassins | NA | 0 | NA |
| **19_1670** | *Mormopterus francoismoutoui* | Trois bassins | NA | 0 | NA |
| **19_1671** | *Mormopterus francoismoutoui* | Trois bassins | NA | 0 | NA |
| **19_1672** | *Mormopterus francoismoutoui* | Trois bassins | NA | 0 | NA |
| **19_1673** | *Mormopterus francoismoutoui* | Trois bassins | NA | 0 | NA |
| **19_1674** | *Mormopterus francoismoutoui* | Trois bassins | NA | 0 | NA |
| **19_1675** | *Mormopterus francoismoutoui* | Trois bassins | NA | 0 | NA |
| **19_1676** | *Mormopterus francoismoutoui* | Trois bassins | NA | 0 | NA |
| **19_1677** | *Mormopterus francoismoutoui* | Trois bassins | NA | 0 | NA |
| **19_1678** | *Mormopterus francoismoutoui* | Trois bassins | NA | 0 | NA |
| **19_1679** | *Mormopterus francoismoutoui* | Trois bassins | NA | 0 | NA |
| **19_1680** | *Mormopterus francoismoutoui* | Trois bassins | NA | 0 | NA |
| **19_1681** | *Mormopterus francoismoutoui* | Transformateur EDF Gillot | NA | 0 | NA |
| **19_1682** | *Mormopterus francoismoutoui* | Transformateur EDF Gillot | NA | 0 | NA |
| **19_1683** | *Mormopterus francoismoutoui* | Transformateur EDF Gillot | NA | 0 | NA |
| **19_1684** | *Mormopterus francoismoutoui* | Transformateur EDF Gillot | NA | 0 | NA |
| **19_1685** | *Mormopterus francoismoutoui* | Transformateur EDF Gillot | NA | 0 | NA |
| **19_1686** | *Mormopterus francoismoutoui* | Transformateur EDF Gillot | NA | 0 | NA |
| **19_1687** | *Mormopterus francoismoutoui* | Transformateur EDF Gillot | NA | 0 | NA |
| **20_0001** | *Mormopterus francoismoutoui* | Trois bassins | NA | 0 | NA |
| **20_0002** | *Mormopterus francoismoutoui* | Trois bassins | NA | 1 | OR752529 |
| **20_0003** | *Mormopterus francoismoutoui* | Trois bassins | NA | 0 | NA |
| **20_0004** | *Mormopterus francoismoutoui* | Trois bassins | NA | 0 | NA |
| **20_0005** | *Mormopterus francoismoutoui* | Trois bassins | NA | 0 | NA |
| **20_0006** | *Mormopterus francoismoutoui* | Trois bassins | NA | 0 | NA |
| **20_0007** | *Mormopterus francoismoutoui* | Trois bassins | NA | 0 | NA |
| **20_0008** | *Mormopterus francoismoutoui* | Trois bassins | NA | 0 | NA |
| **20_0009** | *Mormopterus francoismoutoui* | Trois bassins | NA | 0 | NA |
| **20_0010** | *Mormopterus francoismoutoui* | Trois bassins | NA | 0 | NA |
| **20_0011** | *Mormopterus francoismoutoui* | Trois bassins | NA | 0 | NA |
| **20_0012** | *Mormopterus francoismoutoui* | Trois bassins | NA | 0 | NA |
| **20_0013** | *Mormopterus francoismoutoui* | Trois bassins | NA | 0 | NA |
| **20_0014** | *Mormopterus francoismoutoui* | Trois bassins | NA | 0 | NA |
| **20_0015** | *Mormopterus francoismoutoui* | Trois bassins | NA | 0 | NA |
| **20_0016** | *Mormopterus francoismoutoui* | Trois bassins | NA | 0 | NA |
| **20_0017** | *Mormopterus francoismoutoui* | Trois bassins | NA | 0 | NA |
| **20_0018** | *Mormopterus francoismoutoui* | Trois bassins | NA | 0 | NA |
| **20_0019** | *Mormopterus francoismoutoui* | Trois bassins | NA | 0 | NA |
| **20_0020** | *Mormopterus francoismoutoui* | Trois bassins | NA | 0 | NA |
| **20_0021** | *Mormopterus francoismoutoui* | Trois bassins | NA | 0 | NA |
| **20_0022** | *Mormopterus francoismoutoui* | Trois bassins | NA | 0 | NA |
| **20_0023** | *Mormopterus francoismoutoui* | Trois bassins | NA | 0 | NA |
| **20_0024** | *Mormopterus francoismoutoui* | Trois bassins | NA | 0 | NA |
| **20_0025** | *Mormopterus francoismoutoui* | Trois bassins | NA | 0 | NA |
| **20_0026** | *Mormopterus francoismoutoui* | Trois bassins | NA | 0 | NA |
| **20_0027** | *Mormopterus francoismoutoui* | Trois bassins | NA | 0 | NA |
| **20_0028** | *Mormopterus francoismoutoui* | Trois bassins | NA | 0 | NA |
| **20_0029** | *Mormopterus francoismoutoui* | Trois bassins | NA | 0 | NA |
| **20_0030** | *Mormopterus francoismoutoui* | Trois bassins | NA | 0 | NA |
| **20_0031** | *Mormopterus francoismoutoui* | Trois bassins | NA | 0 | NA |
| **20_0032** | *Mormopterus francoismoutoui* | Trois bassins | NA | 0 | NA |
| **20_0033** | *Mormopterus francoismoutoui* | Trois bassins | NA | 0 | NA |
| **20_0034** | *Mormopterus francoismoutoui* | Trois bassins | NA | 0 | NA |
| **20_0035** | *Mormopterus francoismoutoui* | Trois bassins | NA | 0 | NA |
| **20_0036** | *Mormopterus francoismoutoui* | Trois bassins | NA | 0 | NA |
| **20_0037** | *Mormopterus francoismoutoui* | Trois bassins | NA | 0 | NA |
| **20_0038** | *Mormopterus francoismoutoui* | Trois bassins | NA | 0 | NA |

| | | | | | |
|---|---|---|---|---|---|
| **20_0039** | *Mormopterus francoismoutoui* | Trois bassins | NA | 0 | NA |
| **20_0040** | *Mormopterus francoismoutoui* | Trois bassins | NA | 0 | NA |
| **20_0041** | *Mormopterus francoismoutoui* | Transformateur EDF Gillot | NA | 0 | NA |
| **20_0042** | *Mormopterus francoismoutoui* | Transformateur EDF Gillot | NA | 0 | NA |
| **20_0043** | *Mormopterus francoismoutoui* | Transformateur EDF Gillot | NA | 0 | NA |
| **20_0044** | *Mormopterus francoismoutoui* | Transformateur EDF Gillot | NA | 0 | NA |
| **20_0045** | *Mormopterus francoismoutoui* | Transformateur EDF Gillot | NA | 0 | NA |
| **20_0046** | *Mormopterus francoismoutoui* | Transformateur EDF Gillot | NA | 0 | NA |
| **20_0047** | *Mormopterus francoismoutoui* | Transformateur EDF Gillot | NA | 0 | NA |
| **20_0048** | *Mormopterus francoismoutoui* | Transformateur EDF Gillot | NA | 0 | NA |
| **20_0049** | *Mormopterus francoismoutoui* | Transformateur EDF Gillot | NA | 0 | NA |
| **20_0050** | *Mormopterus francoismoutoui* | Transformateur EDF Gillot | NA | 0 | NA |
| **20_0051** | *Mormopterus francoismoutoui* | Transformateur EDF Gillot | NA | 0 | NA |
| **20_0052** | *Mormopterus francoismoutoui* | Transformateur EDF Gillot | NA | 0 | NA |
| **20_0053** | *Mormopterus francoismoutoui* | Transformateur EDF Gillot | NA | 0 | NA |
| **20_0054** | *Mormopterus francoismoutoui* | Transformateur EDF Gillot | NA | 0 | NA |
| **20_0055** | *Mormopterus francoismoutoui* | Transformateur EDF Gillot | NA | 0 | NA |
| **20_0056** | *Mormopterus francoismoutoui* | Transformateur EDF Gillot | NA | 0 | NA |
| **20_0057** | *Mormopterus francoismoutoui* | Transformateur EDF Gillot | NA | 0 | NA |
| **20_0058** | *Mormopterus francoismoutoui* | Transformateur EDF Gillot | NA | 0 | NA |
| **20_0059** | *Mormopterus francoismoutoui* | Transformateur EDF Gillot | NA | 0 | NA |
| **20_0060** | *Mormopterus francoismoutoui* | Transformateur EDF Gillot | NA | 0 | NA |
| **20_0061** | *Mormopterus francoismoutoui* | Transformateur EDF Gillot | NA | 0 | NA |
| **20_0062** | *Mormopterus francoismoutoui* | Transformateur EDF Gillot | NA | 0 | NA |
| **20_0063** | *Mormopterus francoismoutoui* | Transformateur EDF Gillot | NA | 1 | OR752530 |
| **20_0064** | *Mormopterus francoismoutoui* | Transformateur EDF Gillot | NA | 0 | NA |
| **20_0065** | *Mormopterus francoismoutoui* | Transformateur EDF Gillot | NA | 0 | NA |
| **20_0066** | *Mormopterus francoismoutoui* | Transformateur EDF Gillot | NA | 0 | NA |
| **20_0067** | *Mormopterus francoismoutoui* | Transformateur EDF Gillot | NA | 0 | NA |
| **20_0068** | *Mormopterus francoismoutoui* | Transformateur EDF Gillot | NA | 0 | NA |
| **20_0069** | *Mormopterus francoismoutoui* | Transformateur EDF Gillot | NA | 0 | NA |
| **20_0070** | *Mormopterus francoismoutoui* | Transformateur EDF Gillot | NA | 0 | NA |
| **20_0071** | *Mormopterus francoismoutoui* | Trois bassins | NA | 0 | NA |
| **20_0072** | *Mormopterus francoismoutoui* | Trois bassins | NA | 0 | NA |
| **20_0073** | *Mormopterus francoismoutoui* | Trois bassins | NA | 0 | NA |
| **20_0074** | *Mormopterus francoismoutoui* | Trois bassins | NA | 0 | NA |
| **20_0075** | *Mormopterus francoismoutoui* | Trois bassins | NA | 0 | NA |
| **20_0076** | *Mormopterus francoismoutoui* | Trois bassins | NA | 0 | NA |
| **20_0077** | *Mormopterus francoismoutoui* | Trois bassins | NA | 0 | NA |
| **20_0078** | *Mormopterus francoismoutoui* | Trois bassins | NA | 0 | NA |
| **20_0079** | *Mormopterus francoismoutoui* | Trois bassins | NA | 0 | NA |
| **20_0080** | *Mormopterus francoismoutoui* | Trois bassins | NA | 0 | NA |
| **20_0081** | *Mormopterus francoismoutoui* | Trois bassins | NA | 0 | NA |
| **20_0082** | *Mormopterus francoismoutoui* | Trois bassins | NA | 0 | NA |
| **20_0083** | *Mormopterus francoismoutoui* | Trois bassins | NA | 0 | NA |
| **20_0084** | *Mormopterus francoismoutoui* | Trois bassins | NA | 0 | NA |
| **20_0085** | *Mormopterus francoismoutoui* | Trois bassins | NA | 0 | NA |
| **20_0086** | *Mormopterus francoismoutoui* | Trois bassins | NA | 0 | NA |
| **20_0087** | *Mormopterus francoismoutoui* | Trois bassins | NA | 0 | NA |
| **20_0088** | *Mormopterus francoismoutoui* | Trois bassins | NA | 0 | NA |
| **20_0089** | *Mormopterus francoismoutoui* | Trois bassins | NA | 0 | NA |
| **20_0090** | *Mormopterus francoismoutoui* | Trois bassins | NA | 0 | NA |
| **20_0091** | *Mormopterus francoismoutoui* | Trois bassins | NA | 0 | NA |
| **20_0092** | *Mormopterus francoismoutoui* | Trois bassins | NA | 0 | NA |
| **20_0093** | *Mormopterus francoismoutoui* | Trois bassins | NA | 1 | OR752531 |
| **20_0094** | *Mormopterus francoismoutoui* | Trois bassins | NA | 0 | NA |
| **20_0095** | *Mormopterus francoismoutoui* | Trois bassins | NA | 0 | NA |
| **20_0096** | *Mormopterus francoismoutoui* | Trois bassins | NA | 0 | NA |
| **20_0097** | *Mormopterus francoismoutoui* | Trois bassins | NA | 0 | NA |
| **20_0098** | *Mormopterus francoismoutoui* | Trois bassins | NA | 0 | NA |
| **20_0099** | *Mormopterus francoismoutoui* | Trois bassins | NA | 0 | NA |
| **20_0100** | *Mormopterus francoismoutoui* | Trois bassins | NA | 0 | NA |
| **20_0101** | *Mormopterus francoismoutoui* | Trois bassins | NA | 0 | NA |
| **20_0102** | *Mormopterus francoismoutoui* | Trois bassins | NA | 0 | NA |
| **20_0103** | *Mormopterus francoismoutoui* | Trois bassins | NA | 0 | NA |
| **20_0104** | *Mormopterus francoismoutoui* | Trois bassins | NA | 1 | OR752532 |
| **20_0105** | *Mormopterus francoismoutoui* | Trois bassins | NA | 0 | NA |
| **20_0106** | *Mormopterus francoismoutoui* | Trois bassins | NA | 0 | NA |
| **20_0107** | *Mormopterus francoismoutoui* | Trois bassins | NA | 0 | NA |
| **20_0108** | *Mormopterus francoismoutoui* | Trois bassins | NA | 0 | NA |
| **20_0109** | *Mormopterus francoismoutoui* | Trois bassins | NA | 0 | NA |
| **20_0110** | *Mormopterus francoismoutoui* | Trois bassins | NA | 0 | NA |

| | | | | | |
|---|---|---|---|---|---|
| **20_0111** | *Mormopterus francoismoutoui* | Trois bassins | NA | 0 | NA |
| **20_0112** | *Mormopterus francoismoutoui* | Trois bassins | NA | 0 | NA |
| **20_0113** | *Mormopterus francoismoutoui* | Trois bassins | NA | 0 | NA |
| **20_0114** | *Mormopterus francoismoutoui* | Trois bassins | NA | 0 | NA |
| **20_0115** | *Mormopterus francoismoutoui* | Trois bassins | NA | 0 | NA |
| **20_0116** | *Mormopterus francoismoutoui* | Trois bassins | NA | 1 | OR752533 |
| **20_0117** | *Mormopterus francoismoutoui* | Trois bassins | NA | 0 | NA |
| **20_0118** | *Mormopterus francoismoutoui* | Trois bassins | NA | 0 | NA |
| **20_0119** | *Mormopterus francoismoutoui* | Trois bassins | NA | 0 | NA |
| **20_0120** | *Mormopterus francoismoutoui* | Trois bassins | NA | 0 | NA |
| **20_0121** | *Mormopterus francoismoutoui* | Transformateur EDF Gillot | NA | 0 | NA |
| **20_0122** | *Mormopterus francoismoutoui* | Transformateur EDF Gillot | NA | 0 | NA |
| **20_0123** | *Mormopterus francoismoutoui* | Transformateur EDF Gillot | NA | 0 | NA |
| **20_0124** | *Mormopterus francoismoutoui* | Transformateur EDF Gillot | NA | 0 | NA |
| **20_0125** | *Mormopterus francoismoutoui* | Transformateur EDF Gillot | NA | 0 | NA |
| **20_0126** | *Mormopterus francoismoutoui* | Transformateur EDF Gillot | NA | 0 | NA |
| **20_0127** | *Mormopterus francoismoutoui* | Transformateur EDF Gillot | NA | 0 | NA |
| **20_0128** | *Mormopterus francoismoutoui* | Transformateur EDF Gillot | NA | 0 | NA |
| **20_0129** | *Mormopterus francoismoutoui* | Transformateur EDF Gillot | NA | 0 | NA |
| **20_0130** | *Mormopterus francoismoutoui* | Transformateur EDF Gillot | NA | 0 | NA |
| **20_0131** | *Mormopterus francoismoutoui* | Transformateur EDF Gillot | NA | 0 | NA |
| **20_0132** | *Mormopterus francoismoutoui* | Transformateur EDF Gillot | NA | 0 | NA |
| **20_0133** | *Mormopterus francoismoutoui* | Transformateur EDF Gillot | NA | 0 | NA |
| **20_0134** | *Mormopterus francoismoutoui* | Transformateur EDF Gillot | NA | 0 | NA |
| **20_0135** | *Mormopterus francoismoutoui* | Transformateur EDF Gillot | NA | 0 | NA |
| **20_0136** | *Mormopterus francoismoutoui* | Transformateur EDF Gillot | NA | 0 | NA |
| **20_0137** | *Mormopterus francoismoutoui* | Transformateur EDF Gillot | NA | 0 | NA |
| **20_0138** | *Mormopterus francoismoutoui* | Transformateur EDF Gillot | NA | 0 | NA |
| **20_0139** | *Mormopterus francoismoutoui* | Transformateur EDF Gillot | NA | 0 | NA |
| **20_0140** | *Mormopterus francoismoutoui* | Transformateur EDF Gillot | NA | 0 | NA |
| **20_0141** | *Mormopterus francoismoutoui* | Transformateur EDF Gillot | NA | 0 | NA |
| **20_0142** | *Mormopterus francoismoutoui* | Transformateur EDF Gillot | NA | 0 | NA |
| **20_0143** | *Mormopterus francoismoutoui* | Transformateur EDF Gillot | NA | 0 | NA |
| **20_0144** | *Mormopterus francoismoutoui* | Transformateur EDF Gillot | NA | 0 | NA |
| **20_0145** | *Mormopterus francoismoutoui* | Transformateur EDF Gillot | NA | 0 | NA |
| **20_0146** | *Mormopterus francoismoutoui* | Transformateur EDF Gillot | NA | 0 | NA |
| **20_0147** | *Mormopterus francoismoutoui* | Transformateur EDF Gillot | NA | 0 | NA |
| **20_0148** | *Mormopterus francoismoutoui* | Transformateur EDF Gillot | NA | 0 | NA |
| **20_0149** | *Mormopterus francoismoutoui* | Transformateur EDF Gillot | NA | 0 | NA |
| **20_0150** | *Mormopterus francoismoutoui* | Transformateur EDF Gillot | NA | 0 | NA |
| **20_0151** | *Mormopterus francoismoutoui* | Trois bassins | NA | 0 | NA |
| **20_0152** | *Mormopterus francoismoutoui* | Trois bassins | NA | 0 | NA |
| **20_0153** | *Mormopterus francoismoutoui* | Trois bassins | NA | 0 | NA |
| **20_0154** | *Mormopterus francoismoutoui* | Trois bassins | NA | 0 | NA |
| **20_0155** | *Mormopterus francoismoutoui* | Trois bassins | NA | 0 | NA |
| **20_0156** | *Mormopterus francoismoutoui* | Trois bassins | NA | 0 | NA |
| **20_0157** | *Mormopterus francoismoutoui* | Trois bassins | NA | 0 | NA |
| **20_0158** | *Mormopterus francoismoutoui* | Trois bassins | NA | 0 | NA |
| **20_0159** | *Mormopterus francoismoutoui* | Trois bassins | NA | 0 | NA |
| **20_0160** | *Mormopterus francoismoutoui* | Trois bassins | NA | 0 | NA |
| **20_0161** | *Mormopterus francoismoutoui* | Trois bassins | NA | 0 | NA |
| **20_0162** | *Mormopterus francoismoutoui* | Trois bassins | NA | 0 | NA |
| **20_0163** | *Mormopterus francoismoutoui* | Trois bassins | NA | 0 | NA |
| **20_0164** | *Mormopterus francoismoutoui* | Trois bassins | NA | 0 | NA |
| **20_0165** | *Mormopterus francoismoutoui* | Trois bassins | NA | 0 | NA |
| **20_0166** | *Mormopterus francoismoutoui* | Trois bassins | NA | 0 | NA |
| **20_0167** | *Mormopterus francoismoutoui* | Trois bassins | NA | 0 | NA |
| **20_0168** | *Mormopterus francoismoutoui* | Trois bassins | NA | 0 | NA |
| **20_0169** | *Mormopterus francoismoutoui* | Trois bassins | NA | 0 | NA |
| **20_0170** | *Mormopterus francoismoutoui* | Trois bassins | NA | 0 | NA |
| **20_0171** | *Mormopterus francoismoutoui* | Trois bassins | NA | 0 | NA |
| **20_0172** | *Mormopterus francoismoutoui* | Trois bassins | NA | 0 | NA |
| **20_0173** | *Mormopterus francoismoutoui* | Trois bassins | NA | 0 | NA |
| **20_0174** | *Mormopterus francoismoutoui* | Trois bassins | NA | 0 | NA |
| **20_0175** | *Mormopterus francoismoutoui* | Trois bassins | NA | 0 | NA |
| **20_0176** | *Mormopterus francoismoutoui* | Trois bassins | NA | 0 | NA |
| **20_0177** | *Mormopterus francoismoutoui* | Trois bassins | NA | 0 | NA |
| **20_0178** | *Mormopterus francoismoutoui* | Trois bassins | NA | 0 | NA |
| **20_0179** | *Mormopterus francoismoutoui* | Trois bassins | NA | 0 | NA |
| **20_0180** | *Mormopterus francoismoutoui* | Trois bassins | NA | 0 | NA |
| **20_0181** | *Mormopterus francoismoutoui* | Trois bassins | NA | 0 | NA |
| **20_0182** | *Mormopterus francoismoutoui* | Trois bassins | NA | 0 | NA |

| | | | | | |
|---|---|---|---|---|---|
| **20_0183** | *Mormopterus francoismoutoui* | Trois bassins | NA | 0 | NA |
| **20_0184** | *Mormopterus francoismoutoui* | Trois bassins | NA | 0 | NA |
| **20_0185** | *Mormopterus francoismoutoui* | Trois bassins | NA | 0 | NA |
| **20_0186** | *Mormopterus francoismoutoui* | Trois bassins | NA | 0 | NA |
| **20_0187** | *Mormopterus francoismoutoui* | Trois bassins | NA | 0 | NA |
| **20_0188** | *Mormopterus francoismoutoui* | Trois bassins | NA | 0 | NA |
| **20_0189** | *Mormopterus francoismoutoui* | Trois bassins | NA | 0 | NA |
| **20_0190** | *Mormopterus francoismoutoui* | Trois bassins | NA | 0 | NA |
| **20_0191** | *Mormopterus francoismoutoui* | Trois bassins | NA | 0 | NA |
| **20_0192** | *Mormopterus francoismoutoui* | Trois bassins | NA | 1 | OR752534 |
| **20_0193** | *Mormopterus francoismoutoui* | Trois bassins | NA | 0 | NA |
| **20_0194** | *Mormopterus francoismoutoui* | Trois bassins | NA | 0 | NA |
| **20_0195** | *Mormopterus francoismoutoui* | Trois bassins | NA | 0 | NA |
| **20_0196** | *Mormopterus francoismoutoui* | Trois bassins | NA | 0 | NA |
| **20_0197** | *Mormopterus francoismoutoui* | Trois bassins | NA | 0 | NA |
| **20_0198** | *Mormopterus francoismoutoui* | Trois bassins | NA | 0 | NA |
| **20_0199** | *Mormopterus francoismoutoui* | Trois bassins | NA | 0 | NA |
| **20_0200** | *Mormopterus francoismoutoui* | Trois bassins | NA | 0 | NA |
| **20_0201** | *Mormopterus francoismoutoui* | Transformateur EDF Gillot | NA | 0 | NA |
| **20_0202** | *Mormopterus francoismoutoui* | Transformateur EDF Gillot | NA | 0 | NA |
| **20_0203** | *Mormopterus francoismoutoui* | Transformateur EDF Gillot | NA | 0 | NA |
| **20_0204** | *Mormopterus francoismoutoui* | Transformateur EDF Gillot | NA | 0 | NA |
| **20_0205** | *Mormopterus francoismoutoui* | Transformateur EDF Gillot | NA | 0 | NA |
| **20_0206** | *Mormopterus francoismoutoui* | Transformateur EDF Gillot | NA | 0 | NA |
| **20_0207** | *Mormopterus francoismoutoui* | Transformateur EDF Gillot | NA | 0 | NA |
| **20_0208** | *Mormopterus francoismoutoui* | Transformateur EDF Gillot | NA | 0 | NA |
| **20_0209** | *Mormopterus francoismoutoui* | Transformateur EDF Gillot | NA | 0 | NA |
| **20_0210** | *Mormopterus francoismoutoui* | Transformateur EDF Gillot | NA | 0 | NA |
| **20_0211** | *Mormopterus francoismoutoui* | Transformateur EDF Gillot | NA | 0 | NA |
| **20_0212** | *Mormopterus francoismoutoui* | Transformateur EDF Gillot | NA | 0 | NA |
| **20_0213** | *Mormopterus francoismoutoui* | Transformateur EDF Gillot | NA | 0 | NA |
| **20_0214** | *Mormopterus francoismoutoui* | Transformateur EDF Gillot | NA | 0 | NA |
| **20_0215** | *Mormopterus francoismoutoui* | Transformateur EDF Gillot | NA | 0 | NA |
| **20_0216** | *Mormopterus francoismoutoui* | Transformateur EDF Gillot | NA | 0 | NA |
| **20_0217** | *Mormopterus francoismoutoui* | Transformateur EDF Gillot | NA | 0 | NA |
| **20_0218** | *Mormopterus francoismoutoui* | Transformateur EDF Gillot | NA | 1 | OR752535 |
| **20_0219** | *Mormopterus francoismoutoui* | Transformateur EDF Gillot | NA | 0 | NA |
| **20_0220** | *Mormopterus francoismoutoui* | Transformateur EDF Gillot | NA | 0 | NA |
| **20_0221** | *Mormopterus francoismoutoui* | Transformateur EDF Gillot | NA | 0 | NA |
| **20_0222** | *Mormopterus francoismoutoui* | Transformateur EDF Gillot | NA | 0 | NA |
| **20_0223** | *Mormopterus francoismoutoui* | Transformateur EDF Gillot | NA | 0 | NA |
| **20_0224** | *Mormopterus francoismoutoui* | Transformateur EDF Gillot | NA | 0 | NA |
| **20_0225** | *Mormopterus francoismoutoui* | Transformateur EDF Gillot | NA | 0 | NA |
| **20_0226** | *Mormopterus francoismoutoui* | Transformateur EDF Gillot | NA | 0 | NA |
| **20_0227** | *Mormopterus francoismoutoui* | Transformateur EDF Gillot | NA | 0 | NA |
| **20_0228** | *Mormopterus francoismoutoui* | Transformateur EDF Gillot | NA | 0 | NA |
| **20_0229** | *Mormopterus francoismoutoui* | Transformateur EDF Gillot | NA | 0 | NA |
| **20_0230** | *Mormopterus francoismoutoui* | Transformateur EDF Gillot | NA | 0 | NA |
| **20_0411** | *Mormopterus francoismoutoui* | Transformateur EDF Gillot | NA | 0 | NA |
| **20_0412** | *Mormopterus francoismoutoui* | Transformateur EDF Gillot | NA | 0 | NA |
| **20_0413** | *Mormopterus francoismoutoui* | Transformateur EDF Gillot | NA | 0 | NA |
| **20_0414** | *Mormopterus francoismoutoui* | Transformateur EDF Gillot | NA | 0 | NA |
| **20_0415** | *Mormopterus francoismoutoui* | Transformateur EDF Gillot | NA | 0 | NA |
| **20_0416** | *Mormopterus francoismoutoui* | Transformateur EDF Gillot | NA | 0 | NA |
| **20_0417** | *Mormopterus francoismoutoui* | Transformateur EDF Gillot | NA | 0 | NA |
| **20_0418** | *Mormopterus francoismoutoui* | Transformateur EDF Gillot | NA | 0 | NA |
| **20_0419** | *Mormopterus francoismoutoui* | Transformateur EDF Gillot | NA | 0 | NA |
| **20_0420** | *Mormopterus francoismoutoui* | Transformateur EDF Gillot | NA | 0 | NA |
| **20_0421** | *Mormopterus francoismoutoui* | Transformateur EDF Gillot | NA | 0 | NA |
| **20_0422** | *Mormopterus francoismoutoui* | Transformateur EDF Gillot | NA | 0 | NA |
| **20_0423** | *Mormopterus francoismoutoui* | Transformateur EDF Gillot | NA | 0 | NA |
| **20_0424** | *Mormopterus francoismoutoui* | Transformateur EDF Gillot | NA | 0 | NA |
| **20_0425** | *Mormopterus francoismoutoui* | Transformateur EDF Gillot | NA | 0 | NA |
| **20_0426** | *Mormopterus francoismoutoui* | Transformateur EDF Gillot | NA | 0 | NA |
| **20_0427** | *Mormopterus francoismoutoui* | Transformateur EDF Gillot | NA | 0 | NA |
| **20_0428** | *Mormopterus francoismoutoui* | Transformateur EDF Gillot | NA | 0 | NA |
| **20_0429** | *Mormopterus francoismoutoui* | Transformateur EDF Gillot | NA | 0 | NA |
| **20_0430** | *Mormopterus francoismoutoui* | Transformateur EDF Gillot | NA | 0 | NA |
| **20_0431** | *Mormopterus francoismoutoui* | Transformateur EDF Gillot | NA | 0 | NA |
| **20_0432** | *Mormopterus francoismoutoui* | Transformateur EDF Gillot | NA | 0 | NA |
| **20_0433** | *Mormopterus francoismoutoui* | Transformateur EDF Gillot | NA | 0 | NA |
| **20_0434** | *Mormopterus francoismoutoui* | Transformateur EDF Gillot | NA | 0 | NA |

| | | | | | |
|---|---|---|---|---|---|
| **20_0435** | *Mormopterus francoismoutoui* | Transformateur EDF Gillot | NA | 0 | NA |
| **20_0436** | *Mormopterus francoismoutoui* | Transformateur EDF Gillot | NA | 0 | NA |
| **20_0437** | *Mormopterus francoismoutoui* | Transformateur EDF Gillot | NA | 0 | NA |
| **20_0438** | *Mormopterus francoismoutoui* | Transformateur EDF Gillot | NA | 0 | NA |
| **20_0439** | *Mormopterus francoismoutoui* | Transformateur EDF Gillot | NA | 0 | NA |
| **20_0440** | *Mormopterus francoismoutoui* | Transformateur EDF Gillot | NA | 0 | NA |
| **20_0441** | *Mormopterus francoismoutoui* | Trois bassins | NA | 0 | NA |
| **20_0442** | *Mormopterus francoismoutoui* | Trois bassins | NA | 0 | NA |
| **20_0443** | *Mormopterus francoismoutoui* | Trois bassins | NA | 0 | NA |
| **20_0444** | *Mormopterus francoismoutoui* | Trois bassins | NA | 0 | NA |
| **20_0445** | *Mormopterus francoismoutoui* | Trois bassins | NA | 0 | NA |
| **20_0446** | *Mormopterus francoismoutoui* | Trois bassins | NA | 0 | NA |
| **20_0447** | *Mormopterus francoismoutoui* | Trois bassins | NA | 0 | NA |
| **20_0448** | *Mormopterus francoismoutoui* | Trois bassins | NA | 0 | NA |
| **20_0449** | *Mormopterus francoismoutoui* | Trois bassins | NA | 0 | NA |
| **20_0450** | *Mormopterus francoismoutoui* | Trois bassins | NA | 0 | NA |
| **20_0451** | *Mormopterus francoismoutoui* | Trois bassins | NA | 0 | NA |
| **20_0452** | *Mormopterus francoismoutoui* | Trois bassins | NA | 0 | NA |
| **20_0453** | *Mormopterus francoismoutoui* | Trois bassins | NA | 0 | NA |
| **20_0454** | *Mormopterus francoismoutoui* | Trois bassins | NA | 0 | NA |
| **20_0455** | *Mormopterus francoismoutoui* | Trois bassins | NA | 0 | NA |
| **20_0456** | *Mormopterus francoismoutoui* | Trois bassins | NA | 0 | NA |
| **20_0457** | *Mormopterus francoismoutoui* | Trois bassins | NA | 0 | NA |
| **20_0458** | *Mormopterus francoismoutoui* | Trois bassins | NA | 0 | NA |
| **20_0459** | *Mormopterus francoismoutoui* | Trois bassins | NA | 0 | NA |
| **20_0460** | *Mormopterus francoismoutoui* | Trois bassins | NA | 0 | NA |
| **20_0461** | *Mormopterus francoismoutoui* | Trois bassins | NA | 0 | NA |
| **20_0462** | *Mormopterus francoismoutoui* | Trois bassins | NA | 0 | NA |
| **20_0463** | *Mormopterus francoismoutoui* | Trois bassins | NA | 0 | NA |
| **20_0464** | *Mormopterus francoismoutoui* | Trois bassins | NA | 0 | NA |
| **20_0465** | *Mormopterus francoismoutoui* | Trois bassins | NA | 0 | NA |
| **20_0466** | *Mormopterus francoismoutoui* | Trois bassins | NA | 1 | OR752536 |
| **20_0467** | *Mormopterus francoismoutoui* | Trois bassins | NA | 1 | OR752537 |
| **20_0468** | *Mormopterus francoismoutoui* | Trois bassins | NA | 0 | NA |
| **20_0469** | *Mormopterus francoismoutoui* | Trois bassins | NA | 0 | NA |
| **20_0470** | *Mormopterus francoismoutoui* | Trois bassins | NA | 0 | NA |
| **20_0471** | *Mormopterus francoismoutoui* | Trois bassins | NA | 0 | NA |
| **20_0472** | *Mormopterus francoismoutoui* | Trois bassins | NA | 0 | NA |
| **20_0473** | *Mormopterus francoismoutoui* | Trois bassins | NA | 0 | NA |
| **20_0474** | *Mormopterus francoismoutoui* | Trois bassins | NA | 0 | NA |
| **20_0475** | *Mormopterus francoismoutoui* | Trois bassins | NA | 0 | NA |
| **20_0476** | *Mormopterus francoismoutoui* | Trois bassins | NA | 0 | NA |
| **20_0477** | *Mormopterus francoismoutoui* | Trois bassins | NA | 0 | NA |
| **20_0478** | *Mormopterus francoismoutoui* | Trois bassins | NA | 0 | NA |
| **20_0479** | *Mormopterus francoismoutoui* | Trois bassins | NA | 0 | NA |
| **20_0480** | *Mormopterus francoismoutoui* | Trois bassins | NA | 0 | NA |
| **20_0481** | *Mormopterus francoismoutoui* | Trois bassins | NA | 0 | NA |
| **20_0482** | *Mormopterus francoismoutoui* | Trois bassins | NA | 0 | NA |
| **20_0483** | *Mormopterus francoismoutoui* | Trois bassins | NA | 0 | NA |
| **20_0484** | *Mormopterus francoismoutoui* | Trois bassins | NA | 0 | NA |
| **20_0485** | *Mormopterus francoismoutoui* | Trois bassins | NA | 1 | OR752538 |
| **20_0486** | *Mormopterus francoismoutoui* | Trois bassins | NA | 0 | NA |
| **20_0487** | *Mormopterus francoismoutoui* | Trois bassins | NA | 0 | NA |
| **20_0488** | *Mormopterus francoismoutoui* | Trois bassins | NA | 0 | NA |
| **20_0489** | *Mormopterus francoismoutoui* | Trois bassins | NA | 0 | NA |
| **20_0490** | *Mormopterus francoismoutoui* | Trois bassins | NA | 0 | NA |
| **20_0491** | *Mormopterus francoismoutoui* | Trois bassins | NA | 0 | NA |
| **20_0492** | *Mormopterus francoismoutoui* | Trois bassins | NA | 0 | NA |
| **20_0493** | *Mormopterus francoismoutoui* | Trois bassins | NA | 0 | NA |
| **20_0494** | *Mormopterus francoismoutoui* | Trois bassins | NA | 0 | NA |
| **20_0495** | *Mormopterus francoismoutoui* | Trois bassins | NA | 0 | NA |
| **20_0496** | *Mormopterus francoismoutoui* | Trois bassins | NA | 0 | NA |
| **20_0497** | *Mormopterus francoismoutoui* | Trois bassins | NA | 0 | NA |
| **20_0498** | *Mormopterus francoismoutoui* | Trois bassins | NA | 0 | NA |
| **20_0499** | *Mormopterus francoismoutoui* | Trois bassins | NA | 0 | NA |
| **20_0500** | *Mormopterus francoismoutoui* | Trois bassins | NA | 0 | NA |
| **20_0501** | *Mormopterus francoismoutoui* | Trois bassins | NA | 0 | NA |
| **20_0502** | *Mormopterus francoismoutoui* | Trois bassins | NA | 0 | NA |
| **20_0503** | *Mormopterus francoismoutoui* | Trois bassins | NA | 0 | NA |
| **20_0504** | *Mormopterus francoismoutoui* | Trois bassins | NA | 0 | NA |
| **20_0505** | *Mormopterus francoismoutoui* | Trois bassins | NA | 0 | NA |
| **20_0506** | *Mormopterus francoismoutoui* | Trois bassins | NA | 0 | NA |

| | | | | | |
|---|---|---|---|---|---|
| **20_0507** | *Mormopterus francoismoutoui* | Trois bassins | NA | 0 | NA |
| **20_0508** | *Mormopterus francoismoutoui* | Trois bassins | NA | 1 | OR752539 |
| **20_0509** | *Mormopterus francoismoutoui* | Trois bassins | NA | 0 | NA |
| **20_0510** | *Mormopterus francoismoutoui* | Trois bassins | NA | 0 | NA |
| **20_0511** | *Mormopterus francoismoutoui* | Trois bassins | NA | 0 | NA |
| **20_0512** | *Mormopterus francoismoutoui* | Trois bassins | NA | 0 | NA |
| **20_0513** | *Mormopterus francoismoutoui* | Trois bassins | NA | 0 | NA |
| **20_0514** | *Mormopterus francoismoutoui* | Trois bassins | NA | 0 | NA |
| **20_0515** | *Mormopterus francoismoutoui* | Trois bassins | NA | 0 | NA |
| **20_0516** | *Mormopterus francoismoutoui* | Trois bassins | NA | 0 | NA |
| **20_0517** | *Mormopterus francoismoutoui* | Trois bassins | NA | 0 | NA |
| **20_0518** | *Mormopterus francoismoutoui* | Trois bassins | NA | 0 | NA |
| **20_0519** | *Mormopterus francoismoutoui* | Trois bassins | NA | 0 | NA |
| **20_0520** | *Mormopterus francoismoutoui* | Trois bassins | NA | 0 | NA |
| **20_0521** | *Mormopterus francoismoutoui* | Trois bassins | NA | 0 | NA |
| **20_0522** | *Mormopterus francoismoutoui* | Trois bassins | NA | 0 | NA |
| **20_0523** | *Mormopterus francoismoutoui* | Trois bassins | NA | 0 | NA |
| **20_0524** | *Mormopterus francoismoutoui* | Trois bassins | NA | 0 | NA |
| **20_0525** | *Mormopterus francoismoutoui* | Trois bassins | NA | 0 | NA |
| **20_0526** | *Mormopterus francoismoutoui* | Trois bassins | NA | 0 | NA |
| **20_0527** | *Mormopterus francoismoutoui* | Trois bassins | NA | 0 | NA |
| **20_0528** | *Mormopterus francoismoutoui* | Trois bassins | NA | 0 | NA |
| **20_0529** | *Mormopterus francoismoutoui* | Trois bassins | NA | 0 | NA |
| **20_0530** | *Mormopterus francoismoutoui* | Trois bassins | NA | 0 | NA |
| **20_0531** | *Mormopterus francoismoutoui* | Trois bassins | NA | 0 | NA |
| **20_0532** | *Mormopterus francoismoutoui* | Trois bassins | NA | 0 | NA |
| **20_0533** | *Mormopterus francoismoutoui* | Trois bassins | NA | 0 | NA |
| **20_0534** | *Mormopterus francoismoutoui* | Trois bassins | NA | 0 | NA |
| **20_0535** | *Mormopterus francoismoutoui* | Trois bassins | NA | 0 | NA |
| **20_0536** | *Mormopterus francoismoutoui* | Trois bassins | NA | 0 | NA |
| **20_0537** | *Mormopterus francoismoutoui* | Trois bassins | NA | 0 | NA |
| **20_0538** | *Mormopterus francoismoutoui* | Trois bassins | NA | 0 | NA |
| **20_0539** | *Mormopterus francoismoutoui* | Trois bassins | NA | 0 | NA |
| **20_0540** | *Mormopterus francoismoutoui* | Trois bassins | NA | 0 | NA |
| **20_0541** | *Mormopterus francoismoutoui* | Transformateur EDF Gillot | NA | 0 | NA |
| **20_0542** | *Mormopterus francoismoutoui* | Transformateur EDF Gillot | NA | 0 | NA |
| **20_0543** | *Mormopterus francoismoutoui* | Transformateur EDF Gillot | NA | 0 | NA |
| **20_0544** | *Mormopterus francoismoutoui* | Transformateur EDF Gillot | NA | 0 | NA |
| **20_0545** | *Mormopterus francoismoutoui* | Transformateur EDF Gillot | NA | 0 | NA |
| **20_0546** | *Mormopterus francoismoutoui* | Transformateur EDF Gillot | NA | 0 | NA |
| **20_0547** | *Mormopterus francoismoutoui* | Transformateur EDF Gillot | NA | 0 | NA |
| **20_0548** | *Mormopterus francoismoutoui* | Transformateur EDF Gillot | NA | 0 | NA |
| **20_0549** | *Mormopterus francoismoutoui* | Transformateur EDF Gillot | NA | 0 | NA |
| **20_0550** | *Mormopterus francoismoutoui* | Transformateur EDF Gillot | NA | 0 | NA |
| **20_0551** | *Mormopterus francoismoutoui* | Transformateur EDF Gillot | NA | 0 | NA |
| **20_0552** | *Mormopterus francoismoutoui* | Transformateur EDF Gillot | NA | 0 | NA |
| **20_0553** | *Mormopterus francoismoutoui* | Transformateur EDF Gillot | NA | 0 | NA |
| **20_0554** | *Mormopterus francoismoutoui* | Transformateur EDF Gillot | NA | 0 | NA |
| **20_0555** | *Mormopterus francoismoutoui* | Transformateur EDF Gillot | NA | 0 | NA |
| **20_0556** | *Mormopterus francoismoutoui* | Transformateur EDF Gillot | NA | 0 | NA |
| **20_0557** | *Mormopterus francoismoutoui* | Transformateur EDF Gillot | NA | 0 | NA |
| **20_0558** | *Mormopterus francoismoutoui* | Transformateur EDF Gillot | NA | 1 | OR752540 |
| **20_0559** | *Mormopterus francoismoutoui* | Transformateur EDF Gillot | NA | 0 | NA |
| **20_0560** | *Mormopterus francoismoutoui* | Transformateur EDF Gillot | NA | 0 | NA |
| **20_0561** | *Mormopterus francoismoutoui* | Transformateur EDF Gillot | NA | 0 | NA |
| **20_0562** | *Mormopterus francoismoutoui* | Transformateur EDF Gillot | NA | 0 | NA |
| **20_0563** | *Mormopterus francoismoutoui* | Transformateur EDF Gillot | NA | 0 | NA |
| **20_0564** | *Mormopterus francoismoutoui* | Transformateur EDF Gillot | NA | 0 | NA |
| **20_0565** | *Mormopterus francoismoutoui* | Transformateur EDF Gillot | NA | 0 | NA |
| **20_0566** | *Mormopterus francoismoutoui* | Transformateur EDF Gillot | NA | 0 | NA |
| **20_0567** | *Mormopterus francoismoutoui* | Transformateur EDF Gillot | NA | 0 | NA |
| **20_0568** | *Mormopterus francoismoutoui* | Transformateur EDF Gillot | NA | 0 | NA |
| **20_0569** | *Mormopterus francoismoutoui* | Transformateur EDF Gillot | NA | 0 | NA |
| **20_0570** | *Mormopterus francoismoutoui* | Transformateur EDF Gillot | NA | 0 | NA |
| **20_0611** | *Mormopterus francoismoutoui* | Transformateur EDF Gillot | NA | 0 | NA |
| **20_0612** | *Mormopterus francoismoutoui* | Transformateur EDF Gillot | NA | 0 | NA |
| **20_0613** | *Mormopterus francoismoutoui* | Transformateur EDF Gillot | NA | 0 | NA |
| **20_0614** | *Mormopterus francoismoutoui* | Transformateur EDF Gillot | NA | 0 | NA |
| **20_0615** | *Mormopterus francoismoutoui* | Transformateur EDF Gillot | NA | 0 | NA |
| **20_0616** | *Mormopterus francoismoutoui* | Transformateur EDF Gillot | NA | 0 | NA |
| **20_0617** | *Mormopterus francoismoutoui* | Transformateur EDF Gillot | NA | 0 | NA |
| **20_0618** | *Mormopterus francoismoutoui* | Transformateur EDF Gillot | NA | 0 | NA |

| | | | | | |
|---|---|---|---|---|---|
| **20_0619** | *Mormopterus francoismoutoui* | Transformateur EDF Gillot | NA | 0 | NA |
| **20_0620** | *Mormopterus francoismoutoui* | Transformateur EDF Gillot | NA | 0 | NA |
| **20_0621** | *Mormopterus francoismoutoui* | Transformateur EDF Gillot | NA | 0 | NA |
| **20_0622** | *Mormopterus francoismoutoui* | Transformateur EDF Gillot | NA | 0 | NA |
| **20_0623** | *Mormopterus francoismoutoui* | Transformateur EDF Gillot | NA | 0 | NA |
| **20_0624** | *Mormopterus francoismoutoui* | Transformateur EDF Gillot | NA | 0 | NA |
| **20_0625** | *Mormopterus francoismoutoui* | Transformateur EDF Gillot | NA | 0 | NA |
| **20_0626** | *Mormopterus francoismoutoui* | Transformateur EDF Gillot | NA | 0 | NA |
| **20_0627** | *Mormopterus francoismoutoui* | Transformateur EDF Gillot | NA | 0 | NA |
| **20_0628** | *Mormopterus francoismoutoui* | Transformateur EDF Gillot | NA | 0 | NA |
| **20_0629** | *Mormopterus francoismoutoui* | Transformateur EDF Gillot | NA | 0 | NA |
| **20_0630** | *Mormopterus francoismoutoui* | Transformateur EDF Gillot | NA | 0 | NA |
| **20_0631** | *Mormopterus francoismoutoui* | Transformateur EDF Gillot | NA | 0 | NA |
| **20_0632** | *Mormopterus francoismoutoui* | Transformateur EDF Gillot | NA | 0 | NA |
| **20_0633** | *Mormopterus francoismoutoui* | Transformateur EDF Gillot | NA | 0 | NA |
| **20_0634** | *Mormopterus francoismoutoui* | Transformateur EDF Gillot | NA | 0 | NA |
| **20_0635** | *Mormopterus francoismoutoui* | Transformateur EDF Gillot | NA | 0 | NA |
| **20_0636** | *Mormopterus francoismoutoui* | Transformateur EDF Gillot | NA | 0 | NA |
| **20_0637** | *Mormopterus francoismoutoui* | Transformateur EDF Gillot | NA | 0 | NA |
| **20_0638** | *Mormopterus francoismoutoui* | Transformateur EDF Gillot | NA | 0 | NA |
| **20_0639** | *Mormopterus francoismoutoui* | Transformateur EDF Gillot | NA | 0 | NA |
| **20_0640** | *Mormopterus francoismoutoui* | Transformateur EDF Gillot | NA | 0 | NA |
| **20_0646** | *Mormopterus francoismoutoui* | Transformateur EDF Gillot | NA | 0 | NA |
| **20_0647** | *Mormopterus francoismoutoui* | Transformateur EDF Gillot | NA | 0 | NA |
| **20_0648** | *Mormopterus francoismoutoui* | Transformateur EDF Gillot | NA | 0 | NA |
| **20_0649** | *Mormopterus francoismoutoui* | Transformateur EDF Gillot | NA | 0 | NA |
| **20_0650** | *Mormopterus francoismoutoui* | Transformateur EDF Gillot | NA | 0 | NA |
| **20_0651** | *Mormopterus francoismoutoui* | Transformateur EDF Gillot | NA | 0 | NA |
| **20_0652** | *Mormopterus francoismoutoui* | Transformateur EDF Gillot | NA | 0 | NA |
| **20_0653** | *Mormopterus francoismoutoui* | Transformateur EDF Gillot | NA | 0 | NA |
| **20_0654** | *Mormopterus francoismoutoui* | Transformateur EDF Gillot | NA | 0 | NA |
| **20_0655** | *Mormopterus francoismoutoui* | Transformateur EDF Gillot | NA | 0 | NA |
| **20_0656** | *Mormopterus francoismoutoui* | Transformateur EDF Gillot | NA | 0 | NA |
| **20_0657** | *Mormopterus francoismoutoui* | Transformateur EDF Gillot | NA | 0 | NA |
| **20_0658** | *Mormopterus francoismoutoui* | Transformateur EDF Gillot | NA | 0 | NA |
| **20_0659** | *Mormopterus francoismoutoui* | Transformateur EDF Gillot | NA | 0 | NA |
| **20_0660** | *Mormopterus francoismoutoui* | Transformateur EDF Gillot | NA | 0 | NA |
| **20_0661** | *Mormopterus francoismoutoui* | Transformateur EDF Gillot | NA | 0 | NA |
| **20_0662** | *Mormopterus francoismoutoui* | Transformateur EDF Gillot | NA | 0 | NA |
| **20_0663** | *Mormopterus francoismoutoui* | Transformateur EDF Gillot | NA | 0 | NA |
| **20_0664** | *Mormopterus francoismoutoui* | Transformateur EDF Gillot | NA | 0 | NA |
| **20_0665** | *Mormopterus francoismoutoui* | Transformateur EDF Gillot | NA | 0 | NA |
| **20_0666** | *Mormopterus francoismoutoui* | Transformateur EDF Gillot | NA | 0 | NA |
| **20_0667** | *Mormopterus francoismoutoui* | Transformateur EDF Gillot | NA | 0 | NA |
| **20_0668** | *Mormopterus francoismoutoui* | Transformateur EDF Gillot | NA | 0 | NA |
| **20_0669** | *Mormopterus francoismoutoui* | Transformateur EDF Gillot | NA | 0 | NA |
| **20_0670** | *Mormopterus francoismoutoui* | Transformateur EDF Gillot | NA | 1 | OR752541 |
| **20_0671** | *Mormopterus francoismoutoui* | Transformateur EDF Gillot | NA | 0 | NA |
| **20_0672** | *Mormopterus francoismoutoui* | Transformateur EDF Gillot | NA | 0 | NA |
| **20_0673** | *Mormopterus francoismoutoui* | Transformateur EDF Gillot | NA | 0 | NA |
| **20_0674** | *Mormopterus francoismoutoui* | Transformateur EDF Gillot | NA | 0 | NA |
| **20_0675** | *Mormopterus francoismoutoui* | Transformateur EDF Gillot | NA | 0 | NA |
| **20_0676** | *Mormopterus francoismoutoui* | Transformateur EDF Gillot | NA | 0 | NA |
| **20_0677** | *Mormopterus francoismoutoui* | Transformateur EDF Gillot | NA | 0 | NA |
| **20_0678** | *Mormopterus francoismoutoui* | Transformateur EDF Gillot | NA | 0 | NA |
| **20_0679** | *Mormopterus francoismoutoui* | Transformateur EDF Gillot | NA | 0 | NA |
| **20_0680** | *Mormopterus francoismoutoui* | Transformateur EDF Gillot | NA | 0 | NA |
| **20_0681** | *Mormopterus francoismoutoui* | Transformateur EDF Gillot | NA | 0 | NA |
| **20_0682** | *Mormopterus francoismoutoui* | Transformateur EDF Gillot | NA | 0 | NA |
| **20_0683** | *Mormopterus francoismoutoui* | Transformateur EDF Gillot | NA | 0 | NA |
| **20_0684** | *Mormopterus francoismoutoui* | Transformateur EDF Gillot | NA | 0 | NA |
| **20_0685** | *Mormopterus francoismoutoui* | Transformateur EDF Gillot | NA | 0 | NA |
| **20_0686** | *Mormopterus francoismoutoui* | Transformateur EDF Gillot | NA | 0 | NA |
| **20_0687** | *Mormopterus francoismoutoui* | Transformateur EDF Gillot | NA | 0 | NA |
| **20_0688** | *Mormopterus francoismoutoui* | Transformateur EDF Gillot | NA | 0 | NA |
| **20_0689** | *Mormopterus francoismoutoui* | Transformateur EDF Gillot | NA | 0 | NA |
| **20_0690** | *Mormopterus francoismoutoui* | Transformateur EDF Gillot | NA | 0 | NA |
| **20_0691** | *Mormopterus francoismoutoui* | Transformateur EDF Gillot | NA | 0 | NA |
| **20_0692** | *Mormopterus francoismoutoui* | Transformateur EDF Gillot | NA | 0 | NA |
| **20_0693** | *Mormopterus francoismoutoui* | Transformateur EDF Gillot | NA | 0 | NA |
| **20_0694** | *Mormopterus francoismoutoui* | Transformateur EDF Gillot | NA | 0 | NA |
| **20_0695** | *Mormopterus francoismoutoui* | Transformateur EDF Gillot | NA | 0 | NA |

| | | | | | |
|---|---|---|---|---|---|
| **20_0696** | *Mormopterus francoismoutoui* | Transformateur EDF Gillot | NA | 0 | NA |
| **20_0697** | *Mormopterus francoismoutoui* | Transformateur EDF Gillot | NA | 0 | NA |
| **20_0698** | *Mormopterus francoismoutoui* | Transformateur EDF Gillot | NA | 0 | NA |
| **20_0699** | *Mormopterus francoismoutoui* | Transformateur EDF Gillot | NA | 0 | NA |
| **20_0700** | *Mormopterus francoismoutoui* | Transformateur EDF Gillot | NA | 0 | NA |
| **20_0701** | *Mormopterus francoismoutoui* | Transformateur EDF Gillot | NA | 0 | NA |
| **20_0702** | *Mormopterus francoismoutoui* | Transformateur EDF Gillot | NA | 0 | NA |
| **20_0703** | *Mormopterus francoismoutoui* | Transformateur EDF Gillot | NA | 0 | NA |
| **20_0704** | *Mormopterus francoismoutoui* | Transformateur EDF Gillot | NA | 0 | NA |
| **20_0705** | *Mormopterus francoismoutoui* | Transformateur EDF Gillot | NA | 0 | NA |
| **20_0706** | *Mormopterus francoismoutoui* | Transformateur EDF Gillot | NA | 0 | NA |
| **20_0707** | *Mormopterus francoismoutoui* | Transformateur EDF Gillot | NA | 0 | NA |
| **20_0708** | *Mormopterus francoismoutoui* | Transformateur EDF Gillot | NA | 0 | NA |
| **20_0709** | *Mormopterus francoismoutoui* | Transformateur EDF Gillot | NA | 0 | NA |
| **20_0710** | *Mormopterus francoismoutoui* | Transformateur EDF Gillot | NA | 0 | NA |
| **20_0711** | *Mormopterus francoismoutoui* | Transformateur EDF Gillot | NA | 0 | NA |
| **20_0712** | *Mormopterus francoismoutoui* | Transformateur EDF Gillot | NA | 0 | NA |
| **20_0713** | *Mormopterus francoismoutoui* | Transformateur EDF Gillot | NA | 0 | NA |
| **20_0714** | *Mormopterus francoismoutoui* | Transformateur EDF Gillot | NA | 0 | NA |
| **20_0715** | *Mormopterus francoismoutoui* | Transformateur EDF Gillot | NA | 0 | NA |
| **20_0716** | *Mormopterus francoismoutoui* | Transformateur EDF Gillot | NA | 0 | NA |
| **20_0717** | *Mormopterus francoismoutoui* | Transformateur EDF Gillot | NA | 0 | NA |
| **20_0718** | *Mormopterus francoismoutoui* | Transformateur EDF Gillot | NA | 0 | NA |
| **20_0719** | *Mormopterus francoismoutoui* | Transformateur EDF Gillot | NA | 0 | NA |
| **20_0720** | *Mormopterus francoismoutoui* | Transformateur EDF Gillot | NA | 0 | NA |
| **20_0721** | *Mormopterus francoismoutoui* | Transformateur EDF Gillot | NA | 0 | NA |
| **20_0722** | *Mormopterus francoismoutoui* | Transformateur EDF Gillot | NA | 0 | NA |
| **20_0723** | *Mormopterus francoismoutoui* | Transformateur EDF Gillot | NA | 0 | NA |
| **20_0724** | *Mormopterus francoismoutoui* | Transformateur EDF Gillot | NA | 0 | NA |
| **20_0725** | *Mormopterus francoismoutoui* | Transformateur EDF Gillot | NA | 0 | NA |
| **20_0726** | *Mormopterus francoismoutoui* | Transformateur EDF Gillot | NA | 0 | NA |
| **20_0727** | *Mormopterus francoismoutoui* | Transformateur EDF Gillot | NA | 0 | NA |
| **20_0728** | *Mormopterus francoismoutoui* | Transformateur EDF Gillot | NA | 0 | NA |
| **20_0729** | *Mormopterus francoismoutoui* | Transformateur EDF Gillot | NA | 0 | NA |
| **20_0730** | *Mormopterus francoismoutoui* | Transformateur EDF Gillot | NA | 0 | NA |
| **20_0731** | *Mormopterus francoismoutoui* | Transformateur EDF Gillot | NA | 0 | NA |
| **20_0732** | *Mormopterus francoismoutoui* | Transformateur EDF Gillot | NA | 0 | NA |
| **20_0733** | *Mormopterus francoismoutoui* | Transformateur EDF Gillot | NA | 0 | NA |
| **20_0734** | *Mormopterus francoismoutoui* | Transformateur EDF Gillot | NA | 0 | NA |
| **20_0735** | *Mormopterus francoismoutoui* | Transformateur EDF Gillot | NA | 0 | NA |
| **20_0736** | *Mormopterus francoismoutoui* | Transformateur EDF Gillot | NA | 0 | NA |
| **20_0737** | *Mormopterus francoismoutoui* | Transformateur EDF Gillot | NA | 0 | NA |
| **20_0738** | *Mormopterus francoismoutoui* | Transformateur EDF Gillot | NA | 0 | NA |
| **20_0739** | *Mormopterus francoismoutoui* | Transformateur EDF Gillot | NA | 0 | NA |
| **20_0740** | *Mormopterus francoismoutoui* | Transformateur EDF Gillot | NA | 0 | NA |
| **20_0741** | *Mormopterus francoismoutoui* | Transformateur EDF Gillot | NA | 0 | NA |
| **20_0742** | *Mormopterus francoismoutoui* | Transformateur EDF Gillot | NA | 0 | NA |
| **20_0743** | *Mormopterus francoismoutoui* | Transformateur EDF Gillot | NA | 0 | NA |
| **20_0744** | *Mormopterus francoismoutoui* | Transformateur EDF Gillot | NA | 0 | NA |
| **20_0745** | *Mormopterus francoismoutoui* | Transformateur EDF Gillot | NA | 0 | NA |
| **20_0746** | *Mormopterus francoismoutoui* | Transformateur EDF Gillot | NA | 0 | NA |
| **20_0747** | *Mormopterus francoismoutoui* | Transformateur EDF Gillot | NA | 0 | NA |
| **20_0748** | *Mormopterus francoismoutoui* | Transformateur EDF Gillot | NA | 0 | NA |
| **20_0749** | *Mormopterus francoismoutoui* | Transformateur EDF Gillot | NA | 0 | NA |
| **20_0750** | *Mormopterus francoismoutoui* | Transformateur EDF Gillot | NA | 0 | NA |
| **20_0751** | *Mormopterus francoismoutoui* | Transformateur EDF Gillot | NA | 0 | NA |
| **20_0752** | *Mormopterus francoismoutoui* | Transformateur EDF Gillot | NA | 0 | NA |
| **20_0753** | *Mormopterus francoismoutoui* | Transformateur EDF Gillot | NA | 0 | NA |
| **20_0754** | *Mormopterus francoismoutoui* | Transformateur EDF Gillot | NA | 0 | NA |
| **20_0755** | *Mormopterus francoismoutoui* | Transformateur EDF Gillot | NA | 0 | NA |
| **20_0756** | *Mormopterus francoismoutoui* | Transformateur EDF Gillot | NA | 0 | NA |
| **20_0757** | *Mormopterus francoismoutoui* | Transformateur EDF Gillot | NA | 0 | NA |
| **20_0758** | *Mormopterus francoismoutoui* | Transformateur EDF Gillot | NA | 0 | NA |
| **20_0759** | *Mormopterus francoismoutoui* | Transformateur EDF Gillot | NA | 0 | NA |
| **20_0760** | *Mormopterus francoismoutoui* | Transformateur EDF Gillot | NA | 0 | NA |
| **20_0761** | *Mormopterus francoismoutoui* | Transformateur EDF Gillot | NA | 0 | NA |
| **20_0762** | *Mormopterus francoismoutoui* | Transformateur EDF Gillot | NA | 0 | NA |
| **20_0763** | *Mormopterus francoismoutoui* | Transformateur EDF Gillot | NA | 0 | NA |
| **20_0764** | *Mormopterus francoismoutoui* | Transformateur EDF Gillot | NA | 0 | NA |
| **20_0765** | *Mormopterus francoismoutoui* | Transformateur EDF Gillot | NA | 0 | NA |
| **20_0766** | *Mormopterus francoismoutoui* | Transformateur EDF Gillot | NA | 0 | NA |
| **20_0767** | *Mormopterus francoismoutoui* | Transformateur EDF Gillot | NA | 0 | NA |

| | | | | | |
|---|---|---|---|---|---|
| **20_0768** | *Mormopterus francoismoutoui* | Transformateur EDF Gillot | NA | 0 | NA |
| **20_0769** | *Mormopterus francoismoutoui* | Transformateur EDF Gillot | NA | 0 | NA |
| **20_0770** | *Mormopterus francoismoutoui* | Transformateur EDF Gillot | NA | 0 | NA |
| **20_0771** | *Mormopterus francoismoutoui* | Transformateur EDF Gillot | NA | 0 | NA |
| **20_0772** | *Mormopterus francoismoutoui* | Transformateur EDF Gillot | NA | 0 | NA |
| **20_0773** | *Mormopterus francoismoutoui* | Transformateur EDF Gillot | NA | 0 | NA |
| **20_0774** | *Mormopterus francoismoutoui* | Transformateur EDF Gillot | NA | 0 | NA |
| **20_0775** | *Mormopterus francoismoutoui* | Transformateur EDF Gillot | NA | 0 | NA |
| **20_0776** | *Mormopterus francoismoutoui* | Transformateur EDF Gillot | NA | 0 | NA |
| **20_0777** | *Mormopterus francoismoutoui* | Transformateur EDF Gillot | NA | 0 | NA |
| **20_0778** | *Mormopterus francoismoutoui* | Transformateur EDF Gillot | NA | 0 | NA |
| **20_0779** | *Mormopterus francoismoutoui* | Transformateur EDF Gillot | NA | 0 | NA |
| **20_0780** | *Mormopterus francoismoutoui* | Transformateur EDF Gillot | NA | 0 | NA |
| **20_0781** | *Mormopterus francoismoutoui* | Transformateur EDF Gillot | NA | 0 | NA |
| **20_0782** | *Mormopterus francoismoutoui* | Transformateur EDF Gillot | NA | 0 | NA |
| **20_0783** | *Mormopterus francoismoutoui* | Transformateur EDF Gillot | NA | 0 | NA |
| **20_0784** | *Mormopterus francoismoutoui* | Transformateur EDF Gillot | NA | 0 | NA |
| **20_0785** | *Mormopterus francoismoutoui* | Transformateur EDF Gillot | NA | 0 | NA |
| **20_0786** | *Mormopterus francoismoutoui* | Transformateur EDF Gillot | NA | 0 | NA |
| **20_0787** | *Mormopterus francoismoutoui* | Transformateur EDF Gillot | NA | 0 | NA |
| **20_0788** | *Mormopterus francoismoutoui* | Transformateur EDF Gillot | NA | 0 | NA |
| **20_0789** | *Mormopterus francoismoutoui* | Transformateur EDF Gillot | NA | 0 | NA |
| **20_0790** | *Mormopterus francoismoutoui* | Transformateur EDF Gillot | NA | 0 | NA |
| **20_0791** | *Mormopterus francoismoutoui* | Transformateur EDF Gillot | NA | 0 | NA |
| **20_0792** | *Mormopterus francoismoutoui* | Transformateur EDF Gillot | NA | 0 | NA |
| **20_0793** | *Mormopterus francoismoutoui* | Transformateur EDF Gillot | NA | 0 | NA |
| **20_0794** | *Mormopterus francoismoutoui* | Transformateur EDF Gillot | NA | 0 | NA |
| **20_0795** | *Mormopterus francoismoutoui* | Transformateur EDF Gillot | NA | 0 | NA |
| **20_0846** | *Mormopterus francoismoutoui* | Transformateur EDF Gillot | NA | 0 | NA |
| **20_0847** | *Mormopterus francoismoutoui* | Transformateur EDF Gillot | NA | 0 | NA |
| **20_0848** | *Mormopterus francoismoutoui* | Transformateur EDF Gillot | NA | 0 | NA |
| **20_0849** | *Mormopterus francoismoutoui* | Transformateur EDF Gillot | NA | 0 | NA |
| **20_0850** | *Mormopterus francoismoutoui* | Transformateur EDF Gillot | NA | 0 | NA |
| **20_0851** | *Mormopterus francoismoutoui* | Transformateur EDF Gillot | NA | 0 | NA |
| **20_0852** | *Mormopterus francoismoutoui* | Transformateur EDF Gillot | NA | 0 | NA |
| **20_0853** | *Mormopterus francoismoutoui* | Transformateur EDF Gillot | NA | 0 | NA |
| **20_0854** | *Mormopterus francoismoutoui* | Transformateur EDF Gillot | NA | 0 | NA |
| **20_0855** | *Mormopterus francoismoutoui* | Transformateur EDF Gillot | NA | 0 | NA |
| **20_0856** | *Mormopterus francoismoutoui* | Transformateur EDF Gillot | NA | 0 | NA |
| **20_0857** | *Mormopterus francoismoutoui* | Transformateur EDF Gillot | NA | 0 | NA |
| **20_0858** | *Mormopterus francoismoutoui* | Transformateur EDF Gillot | NA | 0 | NA |
| **20_0859** | *Mormopterus francoismoutoui* | Transformateur EDF Gillot | NA | 0 | NA |
| **20_0860** | *Mormopterus francoismoutoui* | Transformateur EDF Gillot | NA | 0 | NA |
| **20_0861** | *Mormopterus francoismoutoui* | Transformateur EDF Gillot | NA | 0 | NA |
| **20_0862** | *Mormopterus francoismoutoui* | Transformateur EDF Gillot | NA | 0 | NA |
| **20_0863** | *Mormopterus francoismoutoui* | Transformateur EDF Gillot | NA | 0 | NA |
| **20_0864** | *Mormopterus francoismoutoui* | Transformateur EDF Gillot | NA | 0 | NA |
| **20_0865** | *Mormopterus francoismoutoui* | Transformateur EDF Gillot | NA | 0 | NA |
| **20_0866** | *Mormopterus francoismoutoui* | Transformateur EDF Gillot | NA | 0 | NA |
| **20_0867** | *Mormopterus francoismoutoui* | Transformateur EDF Gillot | NA | 0 | NA |
| **20_0868** | *Mormopterus francoismoutoui* | Transformateur EDF Gillot | NA | 0 | NA |
| **20_0869** | *Mormopterus francoismoutoui* | Transformateur EDF Gillot | NA | 0 | NA |
| **20_0870** | *Mormopterus francoismoutoui* | Transformateur EDF Gillot | NA | 0 | NA |
| **20_0871** | *Mormopterus francoismoutoui* | Transformateur EDF Gillot | NA | 0 | NA |
| **20_0872** | *Mormopterus francoismoutoui* | Transformateur EDF Gillot | NA | 0 | NA |
| **20_0873** | *Mormopterus francoismoutoui* | Transformateur EDF Gillot | NA | 0 | NA |
| **20_0874** | *Mormopterus francoismoutoui* | Transformateur EDF Gillot | NA | 0 | NA |
| **20_0875** | *Mormopterus francoismoutoui* | Transformateur EDF Gillot | NA | 0 | NA |
| **GLM0137** | *Mus musculus* | SAVANE ST LEU | F | 0 | NA |
| **GLM0162** | *Mus musculus* | GRAND FOND TAKAMAKA | F | 0 | NA |
| **GLM0324** | *Mus musculus* | CHEMIN FEOGA | F | 1 | OR752584 |
| **GLM0325** | *Mus musculus* | CHEMIN FEOGA | M | 0 | NA |
| **GLM0326** | *Mus musculus* | CHEMIN FEOGA | M | 1 | OR752585 |
| **GLM0336** | *Mus musculus* | COL DE BELLEVUE | F | 0 | NA |
| **GLM0351** | *Mus musculus* | COL DE BELLEVUE | M | 0 | NA |
| **GLM0352** | *Mus musculus* | COL DE BELLEVUE | F | 0 | NA |
| **GLM0365** | *Mus musculus* | FORET TAMARINS MAIDO | F | 0 | NA |
| **GLM0389** | *Mus musculus* | MAIDO | M | 0 | NA |
| **GLM0390** | *Mus musculus* | MAIDO | F | 0 | NA |
| **GLM0392** | *Mus musculus* | MAIDO | F | 0 | NA |
| **GLM0393** | *Mus musculus* | MAIDO | M | 0 | NA |
| **GLM0394** | *Mus musculus* | MAIDO | M | 0 | NA |

| | | | | | |
|---|---|---|---|---|---|
| **GLM0395** | *Mus musculus* | MAIDO | M | 0 | NA |
| **GLM0397** | *Mus musculus* | MAIDO | M | 0 | NA |
| **GLM0446** | *Mus musculus* | RF BOIS DE NEFLES | F | 1 | OR752607 |
| **GLM0521** | *Mus musculus* | SANS SOUCIS | F | 0 | NA |
| **GLM0577** | *Mus musculus* | RIVIÈRE DES ROCHES FRONT DE MER | M | 0 | NA |
| **GLM0609** | *Mus musculus* | PORT EST | F | 0 | NA |
| **GLM0635** | *Mus musculus* | CHEMIN FEOGA | F | 1 | OR752618 |
| **GLM0636** | *Mus musculus* | CHEMIN FEOGA | F | 0 | NA |
| **GLM0637** | *Mus musculus* | CHEMIN FEOGA | M | 1 | OR752619 |
| **GLM0638** | *Mus musculus* | CHEMIN FEOGA | F | 0 | NA |
| **GLM0639** | *Mus musculus* | CHEMIN FEOGA | F | 0 | NA |
| **GLM0645** | *Mus musculus* | CHEMIN FEOGA | M | 0 | NA |
| **GLM0646** | *Mus musculus* | CHEMIN FEOGA | M | 0 | NA |
| **GLM0648** | *Mus musculus* | CHEMIN FEOGA | M | 0 | NA |
| **GLM0649** | *Mus musculus* | CHEMIN FEOGA | F | 0 | NA |
| **GLM0730** | *Mus musculus* | COL DE BELLEVUE | F | 0 | NA |
| **GLM0868** | *Mus musculus* | FORET TAMARINS MAIDO | F | 0 | NA |
| **GLM0870** | *Mus musculus* | BRAS DES CALUMETS | M | 0 | NA |
| **GLM0941** | *Mus musculus* | PLAINE DES CAFRES | F | 0 | NA |
| **GLM0953** | *Mus musculus* | PLAINE DES CAFRES | M | 0 | NA |
| **GLM0958** | *Mus musculus* | MAIDO | F | 0 | NA |
| **GLM0962** | *Mus musculus* | PLAINE DES CAFRES | M | 0 | NA |
| **GLM0006** | *Rattus norvegicus* | CASE MARIANNE | M | 1 | OR752557 |
| **GLM0007** | *Rattus norvegicus* | CASE MARIANNE | M | 0 | NA |
| **GLM0008** | *Rattus norvegicus* | CASE MARIANNE | M | 0 | NA |
| **GLM0009** | *Rattus norvegicus* | CASE MARIANNE | M | 0 | NA |
| **GLM0010** | *Rattus norvegicus* | CASE MARIANNE | F | 1 | OR752558 |
| **GLM0011** | *Rattus norvegicus* | CASE MARIANNE | M | 1 | OR752559 |
| **GLM0012** | *Rattus norvegicus* | CASE MARIANNE | F | 0 | NA |
| **GLM0013** | *Rattus norvegicus* | CASE MARIANNE | F | 0 | NA |
| **GLM0014** | *Rattus norvegicus* | CASE MARIANNE | F | 1 | OR752560 |
| **GLM0015** | *Rattus norvegicus* | PIC ADAM | M | 0 | NA |
| **GLM0025** | *Rattus norvegicus* | PIC ADAM | M | 0 | NA |
| **GLM0042** | *Rattus norvegicus* | ETANG ST PAUL | M | 1 | OR752564 |
| **GLM0059** | *Rattus norvegicus* | ETANG ST PAUL | M | 0 | NA |
| **GLM0146** | *Rattus norvegicus* | ALLEE DES PLUIES D OR | F | 0 | NA |
| **GLM0151** | *Rattus norvegicus* | GRAND FOND TAKAMAKA | M | 1 | OR752566 |
| **GLM0153** | *Rattus norvegicus* | GRAND FOND TAKAMAKA | M | 1 | OR752567 |
| **GLM0155** | *Rattus norvegicus* | GRAND FOND TAKAMAKA | F | 0 | NA |
| **GLM0156** | *Rattus norvegicus* | GRAND FOND TAKAMAKA | M | 1 | OR752568 |
| **GLM0157** | *Rattus norvegicus* | GRAND FOND TAKAMAKA | M | 0 | NA |
| **GLM0159** | *Rattus norvegicus* | GRAND FOND TAKAMAKA | F | 0 | NA |
| **GLM0188** | *Rattus norvegicus* | RIVIÈRE DES ROCHES FRONT DE MER | F | 0 | NA |
| **GLM0273** | *Rattus norvegicus* | PLAINE DES PALMISTES | M | 0 | NA |
| **GLM0277** | *Rattus norvegicus* | PLAINE DES PALMISTES | F | 1 | OR752571 |
| **GLM0278** | *Rattus norvegicus* | PLAINE DES PALMISTES | F | 1 | OR752572 |
| **GLM0284** | *Rattus norvegicus* | PLAINE DES PALMISTES | F | 0 | NA |
| **GLM0294** | *Rattus norvegicus* | PLAINE DES PALMISTES | F | 1 | OR752575 |
| **GLM0301** | *Rattus norvegicus* | PLAINE DES PALMISTES | F | 1 | OR752577 |
| **GLM0307** | *Rattus norvegicus* | PLAINE DES PALMISTES | F | 1 | OR752581 |
| **GLM0327** | *Rattus norvegicus* | CHEMIN FEOGA | F | 0 | NA |
| **GLM0329** | *Rattus norvegicus* | CHEMIN FEOGA | M | 0 | NA |
| **GLM0337** | *Rattus norvegicus* | COL DE BELLEVUE | M | 0 | NA |
| **GLM0338** | *Rattus norvegicus* | COL DE BELLEVUE | M | 0 | NA |
| **GLM0339** | *Rattus norvegicus* | COL DE BELLEVUE | M | 1 | OR752587 |
| **GLM0340** | *Rattus norvegicus* | COL DE BELLEVUE | F | 1 | OR752588 |
| **GLM0341** | *Rattus norvegicus* | COL DE BELLEVUE | M | 0 | NA |
| **GLM0343** | *Rattus norvegicus* | COL DE BELLEVUE | M | 0 | NA |
| **GLM0345** | *Rattus norvegicus* | COL DE BELLEVUE | M | 0 | NA |
| **GLM0353** | *Rattus norvegicus* | COL DE BELLEVUE | M | 0 | NA |
| **GLM0354** | *Rattus norvegicus* | COL DE BELLEVUE | NA | 0 | NA |
| **GLM0355** | *Rattus norvegicus* | COL DE BELLEVUE | F | 0 | NA |
| **GLM0356** | *Rattus norvegicus* | FORET TAMARINS MAIDO | F | 1 | OR752589 |
| **GLM0357** | *Rattus norvegicus* | FORET TAMARINS MAIDO | M | 0 | NA |
| **GLM0358** | *Rattus norvegicus* | FORET TAMARINS MAIDO | M | 0 | NA |
| **GLM0359** | *Rattus norvegicus* | FORET TAMARINS MAIDO | F | 0 | NA |
| **GLM0360** | *Rattus norvegicus* | FORET TAMARINS MAIDO | M | 1 | OR752590 |
| **GLM0361** | *Rattus norvegicus* | FORET TAMARINS MAIDO | F | 0 | NA |
| **GLM0368** | *Rattus norvegicus* | FORET TAMARINS MAIDO | M | 1 | OR752591 |
| **GLM0371** | *Rattus norvegicus* | FORET TAMARINS MAIDO | F | 0 | NA |
| **GLM0374** | *Rattus norvegicus* | FORET TAMARINS MAIDO | M | 0 | NA |
| **GLM0378** | *Rattus norvegicus* | FORET TAMARINS MAIDO | F | 0 | NA |

| | | | | | |
|---|---|---|---|---|---|
| **GLM0380** | *Rattus norvegicus* | FORET TAMARINS MAIDO | M | 1 | OR752592 |
| **GLM0381** | *Rattus norvegicus* | FORET TAMARINS MAIDO | F | 0 | NA |
| **GLM0382** | *Rattus norvegicus* | FORET TAMARINS MAIDO | M | 1 | OR752593 |
| **GLM0383** | *Rattus norvegicus* | FORET TAMARINS MAIDO | M | 1 | OR752594 |
| **GLM0407** | *Rattus norvegicus* | GRAND ETANG | M | 1 | OR752596 |
| **GLM0408** | *Rattus norvegicus* | GRAND ETANG | F | 1 | OR752597 |
| **GLM0410** | *Rattus norvegicus* | GRAND ETANG | F | 1 | OR752599 |
| **GLM0411** | *Rattus norvegicus* | GRAND ETANG | M | 1 | OR752600 |
| **GLM0413** | *Rattus norvegicus* | GRAND ETANG | M | 0 | NA |
| **GLM0428** | *Rattus norvegicus* | RF BOIS DE NEFLES | M | 0 | NA |
| **GLM0429** | *Rattus norvegicus* | RF BOIS DE NEFLES | M | 0 | NA |
| **GLM0430** | *Rattus norvegicus* | RF BOIS DE NEFLES | M | 0 | NA |
| **GLM0431** | *Rattus norvegicus* | RF BOIS DE NEFLES | F | 1 | OR752602 |
| **GLM0437** | *Rattus norvegicus* | RF BOIS DE NEFLES | F | 1 | OR752604 |
| **GLM0440** | *Rattus norvegicus* | RF BOIS DE NEFLES | M | 1 | OR752605 |
| **GLM0442** | *Rattus norvegicus* | RF BOIS DE NEFLES | M | 1 | OR752606 |
| **GLM0476** | *Rattus norvegicus* | PLANTATION MELISSA | F | 0 | NA |
| **GLM0507** | *Rattus norvegicus* | PLAINE DES CAFRES | M | 1 | OR752615 |
| **GLM0508** | *Rattus norvegicus* | PLAINE DES CAFRES | M | 1 | OR752616 |
| **GLM0510** | *Rattus norvegicus* | PLAINE DES CAFRES | M | 1 | OR752617 |
| **GLM0551** | *Rattus norvegicus* | RIVIÈRE DES ROCHES FRONT DE MER | F | 0 | NA |
| **GLM0732** | *Rattus norvegicus* | COL DE BELLEVUE | F | 1 | OR752623 |
| **GLM0734** | *Rattus norvegicus* | COL DE BELLEVUE | F | 1 | OR752624 |
| **GLM0735** | *Rattus norvegicus* | COL DE BELLEVUE | M | 1 | OR752625 |
| **GLM0004** | *Rattus rattus* | RAVINE 3 BASSINS | M | 0 | NA |
| **GLM0005** | *Rattus rattus* | RAVINE 3 BASSINS | M | 0 | NA |
| **GLM0016** | *Rattus rattus* | PIC ADAM | F | 0 | NA |
| **GLM0017** | *Rattus rattus* | PIC ADAM | F | 0 | NA |
| **GLM0018** | *Rattus rattus* | PIC ADAM | F | 1 | OR752561 |
| **GLM0019** | *Rattus rattus* | PIC ADAM | M | 1 | OR752562 |
| **GLM0020** | *Rattus rattus* | PIC ADAM | F | 0 | NA |
| **GLM0021** | *Rattus rattus* | PIC ADAM | M | 0 | NA |
| **GLM0026** | *Rattus rattus* | PIC ADAM | NA | 0 | NA |
| **GLM0027** | *Rattus rattus* | PIC ADAM | F | 0 | NA |
| **GLM0028** | *Rattus rattus* | PIC ADAM | M | 1 | OR752563 |
| **GLM0029** | *Rattus rattus* | PIC ADAM | F | 0 | NA |
| **GLM0030** | *Rattus rattus* | PIC ADAM | M | 0 | NA |
| **GLM0031** | *Rattus rattus* | PIC ADAM | M | 0 | NA |
| **GLM0040** | *Rattus rattus* | ETANG ST PAUL | M | 0 | NA |
| **GLM0041** | *Rattus rattus* | ETANG ST PAUL | NA | 0 | NA |
| **GLM0043** | *Rattus rattus* | ETANG ST PAUL | F | 0 | NA |
| **GLM0044** | *Rattus rattus* | ETANG ST PAUL | M | 0 | NA |
| **GLM0046** | *Rattus rattus* | ETANG ST PAUL | M | 0 | NA |
| **GLM0047** | *Rattus rattus* | ETANG ST PAUL | M | 0 | NA |
| **GLM0048** | *Rattus rattus* | ETANG ST PAUL | F | 0 | NA |
| **GLM0049** | *Rattus rattus* | ETANG ST PAUL | M | 0 | NA |
| **GLM0053** | *Rattus rattus* | ETANG ST PAUL | F | 0 | NA |
| **GLM0054** | *Rattus rattus* | ETANG ST PAUL | F | 0 | NA |
| **GLM0055** | *Rattus rattus* | ETANG ST PAUL | F | 0 | NA |
| **GLM0056** | *Rattus rattus* | ETANG ST PAUL | M | 0 | NA |
| **GLM0057** | *Rattus rattus* | ETANG ST PAUL | F | 0 | NA |
| **GLM0060** | *Rattus rattus* | ETANG ST PAUL | F | 0 | NA |
| **GLM0061** | *Rattus rattus* | ETANG ST PAUL | F | 0 | NA |
| **GLM0062** | *Rattus rattus* | ETANG ST PAUL | M | 0 | NA |
| **GLM0063** | *Rattus rattus* | ETANG ST PAUL | F | 0 | NA |
| **GLM0064** | *Rattus rattus* | ETANG ST PAUL | F | 0 | NA |
| **GLM0065** | *Rattus rattus* | ETANG ST PAUL | F | 0 | NA |
| **GLM0066** | *Rattus rattus* | ETANG ST PAUL | M | 0 | NA |
| **GLM0067** | *Rattus rattus* | ETANG ST PAUL | F | 0 | NA |
| **GLM0068** | *Rattus rattus* | ETANG ST PAUL | F | 0 | NA |
| **GLM0069** | *Rattus rattus* | ETANG ST PAUL | F | 0 | NA |
| **GLM0080** | *Rattus rattus* | RIVIERE DES PLUIES | M | 0 | NA |
| **GLM0093** | *Rattus rattus* | RIVIERE DES PLUIES | M | 0 | NA |
| **GLM0094** | *Rattus rattus* | RIVIERE DES PLUIES | F | 1 | OR752565 |
| **GLM0095** | *Rattus rattus* | RIVIERE DES PLUIES | F | 0 | NA |
| **GLM0102** | *Rattus rattus* | ILET COCO | F | 0 | NA |
| **GLM0106** | *Rattus rattus* | SANS SOUCIS | M | 0 | NA |
| **GLM0107** | *Rattus rattus* | SANS SOUCIS | M | 0 | NA |
| **GLM0108** | *Rattus rattus* | SANS SOUCIS | F | 0 | NA |
| **GLM0109** | *Rattus rattus* | SANS SOUCIS | F | 0 | NA |
| **GLM0110** | *Rattus rattus* | SANS SOUCIS | F | 0 | NA |
| **GLM0117** | *Rattus rattus* | SANS SOUCIS | F | 0 | NA |

| | | | | | |
|---|---|---|---|---|---|
| **GLM0140** | *Rattus rattus* | SAVANE ST LEU | F | 0 | NA |
| **GLM0164** | *Rattus rattus* | GRAND FOND TAKAMAKA | M | 0 | NA |
| **GLM0165** | *Rattus rattus* | GRAND FOND TAKAMAKA | M | 0 | NA |
| **GLM0166** | *Rattus rattus* | GRAND FOND TAKAMAKA | M | 1 | OR752569 |
| **GLM0167** | *Rattus rattus* | GRAND FOND TAKAMAKA | NA | 0 | NA |
| **GLM0184** | *Rattus rattus* | RIVIERE DES ROCHES | M | 0 | NA |
| **GLM0185** | *Rattus rattus* | RIVIERE DES ROCHES | M | 1 | OR752570 |
| **GLM0186** | *Rattus rattus* | RIVIERE DES ROCHES | F | 0 | NA |
| **GLM0189** | *Rattus rattus* | RIVIERE DES ROCHES | M | 0 | NA |
| **GLM0190** | *Rattus rattus* | RIVIERE DES ROCHES | F | 0 | NA |
| **GLM0191** | *Rattus rattus* | RIVIERE DES ROCHES | F | 0 | NA |
| **GLM0199** | *Rattus rattus* | RAVINE 3 BASSINS | M | 0 | NA |
| **GLM0200** | *Rattus rattus* | RAVINE 3 BASSINS | NA | 0 | NA |
| **GLM0201** | *Rattus rattus* | RAVINE 3 BASSINS | M | 0 | NA |
| **GLM0202** | *Rattus rattus* | RAVINE 3 BASSINS | F | 0 | NA |
| **GLM0203** | *Rattus rattus* | RAVINE 3 BASSINS | M | 0 | NA |
| **GLM0204** | *Rattus rattus* | RAVINE 3 BASSINS | F | 0 | NA |
| **GLM0207** | *Rattus rattus* | RAVINE 3 BASSINS | M | 0 | NA |
| **GLM0210** | *Rattus rattus* | PORT EST | M | 0 | NA |
| **GLM0212** | *Rattus rattus* | PORT EST | F | 0 | NA |
| **GLM0222** | *Rattus rattus* | PORT EST | M | 0 | NA |
| **GLM0224** | *Rattus rattus* | PORT EST | M | 0 | NA |
| **GLM0226** | *Rattus rattus* | PORT EST | F | 0 | NA |
| **GLM0227** | *Rattus rattus* | PORT EST | M | 0 | NA |
| **GLM0229** | *Rattus rattus* | PORT EST | F | 0 | NA |
| **GLM0230** | *Rattus rattus* | PORT EST | M | 0 | NA |
| **GLM0231** | *Rattus rattus* | PORT EST | F | 0 | NA |
| **GLM0232** | *Rattus rattus* | PORT EST | F | 0 | NA |
| **GLM0237** | *Rattus rattus* | PORT EST | F | 0 | NA |
| **GLM0289** | *Rattus rattus* | PLAINE DES PALMISTES | M | 1 | OR752573 |
| **GLM0291** | *Rattus rattus* | PLAINE DES PALMISTES | F | 1 | OR752574 |
| **GLM0293** | *Rattus rattus* | PLAINE DES PALMISTES | M | 0 | NA |
| **GLM0296** | *Rattus rattus* | PLAINE DES PALMISTES | F | 0 | NA |
| **GLM0297** | *Rattus rattus* | PLAINE DES PALMISTES | M | 0 | NA |
| **GLM0298** | *Rattus rattus* | PLAINE DES PALMISTES | M | 0 | NA |
| **GLM0300** | *Rattus rattus* | PLAINE DES PALMISTES | M | 1 | OR752576 |
| **GLM0302** | *Rattus rattus* | PLAINE DES PALMISTES | F | 1 | OR752578 |
| **GLM0304** | *Rattus rattus* | PLAINE DES PALMISTES | M | 1 | OR752579 |
| **GLM0305** | *Rattus rattus* | PLAINE DES PALMISTES | F | 0 | NA |
| **GLM0306** | *Rattus rattus* | PLAINE DES PALMISTES | F | 1 | OR752580 |
| **GLM0308** | *Rattus rattus* | PLAINE DES PALMISTES | F | 0 | NA |
| **GLM0309** | *Rattus rattus* | CHEMIN FEOGA | F | 0 | NA |
| **GLM0311** | *Rattus rattus* | CHEMIN FEOGA | M | 1 | OR752582 |
| **GLM0312** | *Rattus rattus* | CHEMIN FEOGA | F | 1 | OR752583 |
| **GLM0313** | *Rattus rattus* | CHEMIN FEOGA | F | 0 | NA |
| **GLM0317** | *Rattus rattus* | CHEMIN FEOGA | F | 0 | NA |
| **GLM0319** | *Rattus rattus* | CHEMIN FEOGA | F | 0 | NA |
| **GLM0320** | *Rattus rattus* | CHEMIN FEOGA | F | 0 | NA |
| **GLM0321** | *Rattus rattus* | CHEMIN FEOGA | M | 0 | NA |
| **GLM0331** | *Rattus rattus* | CHEMIN FEOGA | F | 1 | OR752586 |
| **GLM0342** | *Rattus rattus* | COL DE BELLEVUE | F | 0 | NA |
| **GLM0346** | *Rattus rattus* | COL DE BELLEVUE | F | 0 | NA |
| **GLM0347** | *Rattus rattus* | COL DE BELLEVUE | F | 0 | NA |
| **GLM0362** | *Rattus rattus* | FORET TAMARINS MAIDO | F | 0 | NA |
| **GLM0376** | *Rattus rattus* | FORET TAMARINS MAIDO | M | 0 | NA |
| **GLM0404** | *Rattus rattus* | GRAND ETANG | F | 0 | NA |
| **GLM0406** | *Rattus rattus* | GRAND ETANG | F | 0 | NA |
| **GLM0409** | *Rattus rattus* | GRAND ETANG | M | 1 | OR752598 |
| **GLM0412** | *Rattus rattus* | GRAND ETANG | F | 1 | OR752601 |
| **GLM0414** | *Rattus rattus* | GRAND ETANG | M | 0 | NA |
| **GLM0418** | *Rattus rattus* | GRAND ETANG | M | 0 | NA |
| **GLM0432** | *Rattus rattus* | RF BOIS DE NEFLES | M | 1 | OR752603 |
| **GLM0433** | *Rattus rattus* | RF BOIS DE NEFLES | F | 0 | NA |
| **GLM0434** | *Rattus rattus* | RF BOIS DE NEFLES | F | 0 | NA |
| **GLM0435** | *Rattus rattus* | RF BOIS DE NEFLES | M | 0 | NA |
| **GLM0438** | *Rattus rattus* | RF BOIS DE NEFLES | F | 0 | NA |
| **GLM0439** | *Rattus rattus* | RF BOIS DE NEFLES | M | 0 | NA |
| **GLM0454** | *Rattus rattus* | RF BOIS DE NEFLES | F | 0 | NA |
| **GLM0456** | *Rattus rattus* | PLANTATION MELISSA | F | 0 | NA |
| **GLM0458** | *Rattus rattus* | PLANTATION MELISSA | M | 1 | OR752608 |
| **GLM0459** | *Rattus rattus* | PLANTATION MELISSA | F | 1 | OR752609 |
| **GLM0461** | *Rattus rattus* | PLANTATION MELISSA | M | 1 | OR752610 |

| | | | | | |
|---|---|---|---|---|---|
| **GLM0462** | *Rattus rattus* | PLANTATION MELISSA | F | 0 | NA |
| **GLM0468** | *Rattus rattus* | PLANTATION MELISSA | M | 1 | OR752611 |
| **GLM0469** | *Rattus rattus* | PLANTATION MELISSA | M | 0 | NA |
| **GLM0470** | *Rattus rattus* | PLANTATION MELISSA | M | 1 | OR752612 |
| **GLM0477** | *Rattus rattus* | PLANTATION MELISSA | F | 1 | OR752613 |
| **GLM0487** | *Rattus rattus* | BRAS DES CALUMETS | M | 0 | NA |
| **GLM0488** | *Rattus rattus* | BRAS DES CALUMETS | F | 0 | NA |
| **GLM0490** | *Rattus rattus* | BRAS DES CALUMETS | M | 0 | NA |
| **GLM0492** | *Rattus rattus* | BRAS DES CALUMETS | M | 1 | OR752614 |
| **GLM0550** | *Rattus rattus* | RIVIÈRE DES ROCHES FRONT DE MER | M | 0 | NA |
| **GLM0552** | *Rattus rattus* | RIVIÈRE DES ROCHES FRONT DE MER | M | 0 | NA |
| **GLM0553** | *Rattus rattus* | RIVIÈRE DES ROCHES FRONT DE MER | F | 0 | NA |
| **GLM0554** | *Rattus rattus* | RIVIÈRE DES ROCHES FRONT DE MER | M | 0 | NA |
| **GLM0555** | *Rattus rattus* | RIVIÈRE DES ROCHES FRONT DE MER | M | 0 | NA |
| **GLM0599** | *Rattus rattus* | PORT EST | M | 0 | NA |
| **GLM0601** | *Rattus rattus* | PORT EST | M | 0 | NA |
| **GLM0603** | *Rattus rattus* | PORT EST | M | 0 | NA |
| **GLM0606** | *Rattus rattus* | PORT EST | M | 0 | NA |
| **GLM0716** | *Rattus rattus* | PLAIN DES PALMISTES | F | 1 | OR752620 |
| **GLM0717** | *Rattus rattus* | PLAIN DES PALMISTES | M | 1 | OR752621 |
| **GLM0718** | *Rattus rattus* | PLAIN DES PALMISTES | M | 0 | NA |
| **GLM0725** | *Rattus rattus* | COL DE BELLEVUE | F | 1 | OR752622 |
| **GLM0726** | *Rattus rattus* | COL DE BELLEVUE | F | 0 | NA |
| **GLM0727** | *Rattus rattus* | COL DE BELLEVUE | F | 0 | NA |
| **GLM0731** | *Rattus rattus* | COL DE BELLEVUE | F | 0 | NA |
| **GLM0939** | *Rattus rattus* | ETANG ST PAUL | F | 0 | NA |
| **GLM0022** | *Suncus murinus* | PIC ADAM | M | 0 | NA |
| **GLM0023** | *Suncus murinus* | PIC ADAM | M | 0 | NA |
| **GLM0024** | *Suncus murinus* | PIC ADAM | F | 0 | NA |
| **GLM0032** | *Suncus murinus* | PIC ADAM | M | 0 | NA |
| **GLM0033** | *Suncus murinus* | PIC ADAM | M | 0 | NA |
| **GLM0034** | *Suncus murinus* | ETANG ST PAUL | M | 0 | NA |
| **GLM0035** | *Suncus murinus* | ETANG ST PAUL | F | 0 | NA |
| **GLM0036** | *Suncus murinus* | ETANG ST PAUL | M | 0 | NA |
| **GLM0070** | *Suncus murinus* | ETANG ST PAUL | M | 0 | NA |
| **GLM0071** | *Suncus murinus* | ETANG ST PAUL | M | 0 | NA |
| **GLM0072** | *Suncus murinus* | ETANG ST PAUL | M | 0 | NA |
| **GLM0073** | *Suncus murinus* | RIVIERE DES PLUIES | F | 0 | NA |
| **GLM0074** | *Suncus murinus* | RIVIERE DES PLUIES | M | 0 | NA |
| **GLM0075** | *Suncus murinus* | RIVIERE DES PLUIES | F | 0 | NA |
| **GLM0076** | *Suncus murinus* | RIVIERE DES PLUIES | M | 0 | NA |
| **GLM0077** | *Suncus murinus* | RIVIERE DES PLUIES | F | 0 | NA |
| **GLM0078** | *Suncus murinus* | RIVIERE DES PLUIES | F | 0 | NA |
| **GLM0079** | *Suncus murinus* | RIVIERE DES PLUIES | F | 0 | NA |
| **GLM0081** | *Suncus murinus* | RIVIERE DES PLUIES | M | 0 | NA |
| **GLM0082** | *Suncus murinus* | RIVIERE DES PLUIES | M | 0 | NA |
| **GLM0083** | *Suncus murinus* | RIVIERE DES PLUIES | M | 0 | NA |
| **GLM0084** | *Suncus murinus* | RIVIERE DES PLUIES | F | 0 | NA |
| **GLM0085** | *Suncus murinus* | RIVIERE DES PLUIES | M | 0 | NA |
| **GLM0086** | *Suncus murinus* | RIVIERE DES PLUIES | F | 0 | NA |
| **GLM0087** | *Suncus murinus* | RIVIERE DES PLUIES | M | 0 | NA |
| **GLM0088** | *Suncus murinus* | RIVIERE DES PLUIES | M | 0 | NA |
| **GLM0089** | *Suncus murinus* | RIVIERE DES PLUIES | F | 0 | NA |
| **GLM0090** | *Suncus murinus* | RIVIERE DES PLUIES | F | 0 | NA |
| **GLM0091** | *Suncus murinus* | RIVIERE DES PLUIES | M | 0 | NA |
| **GLM0092** | *Suncus murinus* | RIVIERE DES PLUIES | M | 0 | NA |
| **GLM0098** | *Suncus murinus* | ILET COCO | F | 0 | NA |
| **GLM0099** | *Suncus murinus* | ILET COCO | F | 0 | NA |
| **GLM0100** | *Suncus murinus* | ILET COCO | M | 0 | NA |
| **GLM0101** | *Suncus murinus* | ILET COCO | M | 0 | NA |
| **GLM0103** | *Suncus murinus* | ILET COCO | M | 0 | NA |
| **GLM0113** | *Suncus murinus* | SANS SOUCIS | F | 0 | NA |
| **GLM0147** | *Suncus murinus* | GRAND FOND TAKAMAKA | M | 0 | NA |
| **GLM0158** | *Suncus murinus* | GRAND FOND TAKAMAKA | M | 0 | NA |
| **GLM0160** | *Suncus murinus* | GRAND FOND TAKAMAKA | M | 0 | NA |
| **GLM0161** | *Suncus murinus* | GRAND FOND TAKAMAKA | F | 0 | NA |
| **GLM0161** | *Suncus murinus* | GRAND FOND TAKAMAKA | F | 0 | NA |
| **GLM0168** | *Suncus murinus* | RIVIÈRE DES ROCHES FRONT DE MER | M | 0 | NA |
| **GLM0169** | *Suncus murinus* | RIVIÈRE DES ROCHES FRONT DE MER | F | 0 | NA |
| **GLM0170** | *Suncus murinus* | RIVIÈRE DES ROCHES FRONT DE MER | F | 0 | NA |
| **GLM0171** | *Suncus murinus* | RIVIÈRE DES ROCHES FRONT DE MER | M | 0 | NA |
| **GLM0172** | *Suncus murinus* | RIVIÈRE DES ROCHES FRONT DE MER | M | 0 | NA |

| | | | | | |
|---|---|---|---|---|---|
| **GLM0187** | *Suncus murinus* | RIVIERE DES ROCHES | M | 0 | NA |
| **GLM0208** | *Suncus murinus* | PORT EST | F | 0 | NA |
| **GLM0209** | *Suncus murinus* | PORT EST | F | 0 | NA |
| **GLM0213** | *Suncus murinus* | PORT EST | M | 0 | NA |
| **GLM0215** | *Suncus murinus* | PORT EST | F | 0 | NA |
| **GLM0216** | *Suncus murinus* | PORT EST | M | 0 | NA |
| **GLM0217** | *Suncus murinus* | PORT EST | F | 0 | NA |
| **GLM0218** | *Suncus murinus* | PORT EST | M | 0 | NA |
| **GLM0219** | *Suncus murinus* | PORT EST | M | 0 | NA |
| **GLM0220** | *Suncus murinus* | PORT EST | M | 0 | NA |
| **GLM0221** | *Suncus murinus* | PORT EST | M | 0 | NA |
| **GLM0233** | *Suncus murinus* | PORT EST | F | 0 | NA |
| **GLM0270** | *Suncus murinus* | PLAINE DES PALMISTES | M | 0 | NA |
| **GLM0271** | *Suncus murinus* | PLAINE DES PALMISTES | F | 0 | NA |
| **GLM0285** | *Suncus murinus* | PLAINE DES PALMISTES | M | 0 | NA |
| **GLM0286** | *Suncus murinus* | PLAINE DES PALMISTES | F | 0 | NA |
| **GLM0315** | *Suncus murinus* | CHEMIN FEOGA | M | 0 | NA |
| **GLM0316** | *Suncus murinus* | CHEMIN FEOGA | M | 0 | NA |
| **GLM0323** | *Suncus murinus* | CHEMIN FEOGA | M | 0 | NA |
| **GLM0350** | *Suncus murinus* | CASE KOUSSAY | F | 0 | NA |
| **GLM0364** | *Suncus murinus* | CASE KOUSSAY | F | 0 | NA |
| **GLM0386** | *Suncus murinus* | MAIDO | M | 0 | NA |
| **GLM0391** | *Suncus murinus* | MAIDO | M | 0 | NA |
| **GLM0396** | *Suncus murinus* | MAIDO | M | 0 | NA |
| **GLM0402** | *Suncus murinus* | GRAND ETANG | M | 0 | NA |
| **GLM0403** | *Suncus murinus* | GRAND ETANG | F | 1 | OR752595 |
| **GLM0415** | *Suncus murinus* | GRAND ETANG | M | 0 | NA |
| **GLM0416** | *Suncus murinus* | GRAND ETANG | F | 0 | NA |
| **GLM0421** | *Suncus murinus* | GRAND ETANG | F | 0 | NA |
| **GLM0427** | *Suncus murinus* | RF BOIS DE NEFLES | M | 0 | NA |
| **GLM0474** | *Suncus murinus* | PLANTATION MELISSA | M | 0 | NA |
| **GLM0475** | *Suncus murinus* | PLANTATION MELISSA | M | 0 | NA |
| **GLM0485** | *Suncus murinus* | BRAS DES CALUMETS | F | 0 | NA |
| **GLM0486** | *Suncus murinus* | BRAS DES CALUMETS | F | 0 | NA |
| **GLM0509** | *Suncus murinus* | PLAINE DES CAFRES | M | 0 | NA |
| **GLM0546** | *Suncus murinus* | RIVIÈRE DES ROCHES FRONT DE MER | M | 0 | NA |
| **GLM0547** | *Suncus murinus* | RIVIÈRE DES ROCHES FRONT DE MER | F | 0 | NA |
| **GLM0548** | *Suncus murinus* | RIVIÈRE DES ROCHES FRONT DE MER | M | 0 | NA |
| **GLM0549** | *Suncus murinus* | RIVIÈRE DES ROCHES FRONT DE MER | M | 0 | NA |
| **GLM0564** | *Suncus murinus* | RIVIÈRE DES ROCHES FRONT DE MER | M | 0 | NA |
| **GLM0565** | *Suncus murinus* | RIVIÈRE DES ROCHES FRONT DE MER | M | 0 | NA |
| **GLM0574** | *Suncus murinus* | RIVIÈRE DES ROCHES FRONT DE MER | F | 0 | NA |
| **GLM0575** | *Suncus murinus* | RIVIÈRE DES ROCHES FRONT DE MER | F | 0 | NA |
| **GLM0576** | *Suncus murinus* | RIVIÈRE DES ROCHES FRONT DE MER | F | 0 | NA |
| **GLM0608** | *Suncus murinus* | PORT EST | F | 0 | NA |
| **GLM0904** | *Suncus murinus* | ETANG ST PAUL | M | 0 | NA |
| **GLM0905** | *Suncus murinus* | ETANG ST PAUL | F | 0 | NA |
| **GLM0906** | *Suncus murinus* | ETANG ST PAUL | F | 0 | NA |
| **GLM0907** | *Suncus murinus* | ETANG ST PAUL | F | 0 | NA |
| **GLM0908** | *Suncus murinus* | ETANG ST PAUL | M | 0 | NA |
| **GLM0909** | *Suncus murinus* | ETANG ST PAUL | M | 0 | NA |
| **GLM0910** | *Suncus murinus* | ETANG ST PAUL | M | 0 | NA |
| **GLM0952** | *Suncus murinus* | PLAINE DES CAFRES | M | 0 | NA |
